# From cellular regeneration toward functional integration: a 15-year bibliometric analysis of inner ear hair cell regeneration (2011–2025)

**DOI:** 10.3389/fncel.2026.1888383

**Published:** 2026-08-18

**Authors:** Xiaoyi Zhang, Lintao Tang, Dongmei Guan, Xinghong Liu, Hui Xie

**Affiliations:** 1 Chengdu University of Traditional Chinese Medicine, Chengdu, China; 2 Hospital of Chengdu University of Traditional Chinese Medicine, Chengdu, China; 3 Sichuan Academy of Medical Sciences and Sichuan Provincial People's Hospital, Chengdu, China

**Keywords:** bibliometric analysis, functional integration, inner ear hair cells, regeneration, supporting cells

## Abstract

Sensorineural hearing loss, affecting over 1.5 billion people worldwide, results largely from irreversible damage to inner ear hair cells—specialized mechanosensory receptor cells that mammals cannot spontaneously regenerate. While recent advances in gene therapy and stem cell biology have raised hopes for biological repair, the rapid expansion of research has made it difficult to identify overarching trends, knowledge gaps, and translational bottlenecks. This study provides the first bibliometric analysis focusing on inner ear hair cell regeneration, covering 1,035 publications identified from the Web of Science Core Collection after systematic screening. To ensure the robustness of the findings and assess the sensitivity of the findings to database choice, we performed cross-database validation using Scopus and PubMed, which revealed high overlap rates (82.65% for PubMed and 75.76% for Scopus) and consistent patterns in the non-overlapping subset, suggesting that the primary WoSCC-based findings are not qualitatively altered by the inclusion of literature from other major databases. Using VOSviewer and CiteSpace, we analyzed publication trajectories, country/institution contributions, collaboration networks, and keyword dynamics. The results reveal a sustained increase in annual output, with the United States and China emerging as the two most productive and collaboratively central countries in the global research network. Keyword burst analysis shows a temporal shift from structural characterization (early 2010s) to signaling pathways (mid-late 2010s), and more recently to pathological mechanisms and protective strategies (late 2010s-present), including synaptopathy, transplantation, protection, ototoxicity, replacement, autophagy, and disease. VOSviewer co-occurrence analysis further reveals thematic clusters centered on hair cell regeneration and signaling, ototoxic injury and protection, and gene therapy and delivery, suggesting a field expanding from basic mechanisms toward translational research. These findings demonstrate that the field has diversified from a predominant emphasis on cellular regeneration toward broader attention to functional reconstruction of the hair cell–spiral ganglion neuron synapse. This transition underscores a growing emphasis on multi-target synaptic integration and highlights an unmet need for immune-compatible, subtype-specific gene delivery systems. Overall, this analysis, supplemented by cross-database consistency checks, provides a quantitative roadmap.

## 1 Introduction

The World Report on Hearing indicates that over 1.5 billion people worldwide (one-fifth of the global population) currently experience some degree of hearing loss, a figure projected to approach 2.5 billion by 2050 (Chadha et al., 2021; Li et al., 2024). Sensorineural hearing loss (SNHL), the most prevalent type of hearing impairment, is pathologically characterized by irreversible damage to cochlear sensory hair cells and spiral ganglion neurons, commonly induced by ototoxic drugs (e.g., cisplatin, aminoglycosides), aging, noise exposure, and inflammation (Zhang et al., 2025). Unlike fish and birds, mammalian cochlear hair cells progressively lose their regenerative capacity after birth; once damaged in the mature cochlea—whether by noise, ototoxic agents, or aging—they typically cannot self-repair or regenerate, resulting in permanent hearing loss (Smith-Cortinez et al., 2023). Current clinical interventions for SNHL primarily rely on assistive devices such as hearing aids and cochlear implants (Matsunaga and Nakagawa, 2023). However, hearing aids often provide suboptimal sound perception in noisy environments, while cochlear implants require invasive surgery and provide limited sound perception in challenging listening environments. Thus, there is an urgent clinical need for more effective therapeutic strategies. Consequently, exploring approaches to restore inner ear hair cells—including outer hair cells responsible for sound amplification and inner hair cells responsible for signal encoding—has become a fundamental question in cellular neuroscience: how do specialized mechanosensory receptor cells regenerate after injury, and what cellular and molecular mechanisms govern this process? The present bibliometric analysis focuses specifically on the regeneration aspect of this broader question, based on a literature corpus identified through a regeneration-focused search strategy. While protection and synaptic repair are recognized as important adjacent domains, the present corpus does not comprehensively cover the full bodies of literature in those areas.

Although the mammalian inner ear has evolutionarily lost the capacity for spontaneous regeneration, multiple therapeutic strategies have emerged to address this challenge. These strategies can be conceptually categorized into three broad paradigms: (1) hair cell protection, aimed at preventing or mitigating damage to existing hair cells through pharmacological agents or genetic modulation of stress-response pathways; (2) *de novo* hair cell regeneration, achieved via supporting cell reprogramming (e.g., Atoh1-mediated transdifferentiation), stem cell-derived hair cell differentiation, or gene editing to restore endogenous regenerative capacity; and (3) synaptic and neural repair, focused on reconstructing ribbon synapses between regenerated or surviving hair cells and spiral ganglion neurons, or preserving spiral ganglion neuron integrity. Advances in gene editing, directed stem cell differentiation, small-molecule drug screening, and biomaterial engineering (Hosseinkhani et al., 2023; Matsunaga and Nakagawa, 2023; Qi et al., 2024; Smith-Cortinez et al., 2023; Zhang et al., 2024)—have progressively moved the goal of repairing and functionally regenerating mature cochlear hair cells from theoretical concept toward applied exploration. Notably, the inner ear exhibits a high degree of homology: both auditory and vestibular hair cells originate from the otic placode, depend on the key transcription factor Atoh1 for hair cell fate determination during terminal differentiation (You et al., 2023), share consistent epigenetic regulatory mechanisms (Balendran et al., 2022), and display co-localized expression of several key proteins including RBM24 (Shi et al., 2022). Postnatally, both cell types rely on supporting cells as potential progenitor sources (Berlingeri et al., 2022; Janesick et al., 2022), and their regenerative potential is coordinately regulated by conserved signaling pathways such as Wnt and Notch (Carpena et al., 2025; Noda et al., 2025). Based on this biological commonality, research on the regeneration and repair of these two hair cell types has long proceeded in parallel with mutual reinforcement, with vestibular organs (e.g., the saccule) frequently serving as ideal models for studying hair cell regeneration and providing important insights for auditory hair cell regeneration strategies. Thus, conducting a macro-level analysis that integrates both systems into a cohesive knowledge framework is not only grounded in solid biological rationale but also facilitates the extraction of generalizable principles applicable to auditory repair. From a cellular neuroscience perspective, understanding how these two hair cell types respond to injury and regeneration cues provides a unique window into the plasticity and repair capacity of sensory receptor cells and their synaptic reconnection with primary auditory neurons in the mammalian nervous system.

Given the rapid accumulation of research output in this field, the present study employs bibliometric methods to conduct a macro-level analysis of inner ear hair cell regeneration and repair as an integrated knowledge system. By systematically examining annual publication volumes, citation frequencies, country/regional distributions, and keyword co-occurrence networks, this study aims to delineate the research frontiers, core contributors, and collaborative networks within this interdisciplinary field, thereby elucidating the evolutionary trends of its research themes. Importantly, by identifying evolutionary trends in cellular and molecular approaches—from structural characterization to signaling pathway modulation and gene therapy—this analysis provides a quantitative roadmap for future investigations in the cellular neuroscience of sensory hair cell regeneration and synaptic repair.

## 2 Historical landscape of inner ear hair cell regeneration research

**Figure 1:**
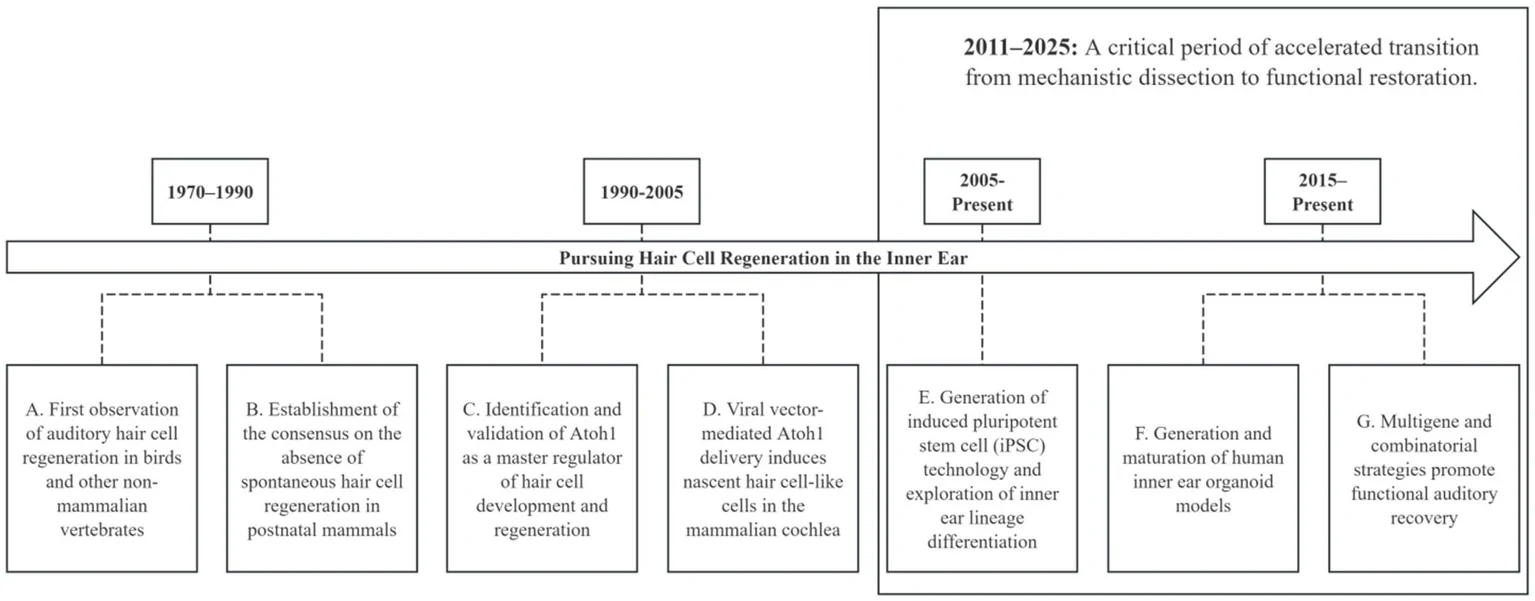
Pursuing hair cell regeneration in the inner ear.

Several historical milestones have shaped this field. The first observation of auditory hair cell regeneration in birds and other non-mammalian vertebrates was reported in the late 1980s (Cruz et al., 1987; Ryals and Rubel, 1988), establishing that hair cell regeneration is possible in vertebrates. Subsequently, a consensus emerged that postnatal mammals lack spontaneous hair cell regeneration. A major breakthrough came with the identification and validation of Atoh1 (also known as Math1) as a master regulator of hair cell development and regeneration (Bermingham et al., 1999; Izumikawa et al., 2005). Viral vector-mediated Atoh1 delivery was shown to induce nascent hair cell-like cells in the mammalian cochlea (Izumikawa et al., 2005). The generation of induced pluripotent stem cell (iPSC) technology opened new avenues for inner ear lineage differentiation (Jeong et al., 2024; Takahashi and Yamanaka, 2006). More recently, human inner ear organoid models have been generated and matured (Koehler et al., 2017; van der Valk et al., 2026), and multigene or combinatorial strategies have been developed to promote functional auditory recovery (Costa et al., 2015; McGovern et al., 2024; Zhang et al., 2024) (Figure 1).

## 3 Quantitative analysis of research landscape based on Bibliometrics

Since the first observation of hair cell regeneration in birds in the late 1980s (Cruz et al., 1987; Ryals and Rubel, 1988), research in this field has undergone a paradigm shift from mechanistic exploration to technological breakthrough. The identification of the Atoh1 gene in the 1990s laid the foundation for gene therapy, while the emergence of stem cell technologies and CRISPR editing after 2010 further expanded regenerative strategies (Figure 1). However, traditional reviews are limited in their ability to quantify the developmental trajectories of different technological pathways and their collaborative patterns. Therefore, this study employs bibliometric analysis to elucidate the evolutionary dynamics of research interest in gene therapy and stem cell technologies (via keyword co-occurrence networks), the intensity of transnational collaborations (via institutional collaboration maps), and future directions (via burst detection), aiming to provide data-driven insights to support technological translation.

### 3.1 Materials and methods

#### 3.1.1 Data sources and search strategy

The Web of Science Core Collection (WoSCC) database was searched to retrieve relevant literature. To ensure the quality of English-language publications, the search was restricted to documents indexed in the Social Sciences Citation Index (SSCI) (2020–present) and the Science Citation Index Expanded (SCI-EXPANDED) (2011–present). An advanced search was conducted using the following query: [TS = (“hair cells” OR “inner ear hair cell” OR “inner ear hair cells” OR “cochlea inner ear hair cells”)] AND TS = (regeneration OR regenerated OR regenerate OR recycling OR recycle OR rebirth OR regeneratively OR recuperation OR reproduction OR recovery OR regrowth OR reuse OR restore OR regenerating OR renewable OR regenerative OR recyclable OR reclaiming OR revivify OR reborn OR rehabilitation OR reusability OR rejuvenation OR revive OR renew OR regenesis OR renovation OR reviviscence OR revivifying OR retroaction OR retroact OR resurgence OR restoring OR rendition OR renascence OR recuperate OR recreate OR reconstitution OR rewrite OR renaturation OR resurgent OR restoration OR resurrection OR revitalization OR shoot regeneration OR refresh OR reproducibility OR regeneration system OR reprocess OR reutilization). The search was refined by excluding the following document types: Proceeding Paper, Editorial Material, Book Chapters, Correction, Data Paper, Meeting Abstract, and Retracted Publication. Publications in languages other than English (German, Portuguese, Chinese, Estonian, French, and Russian) were also excluded. The timespan was set from January 1, 2010, to December 31, 2025 (publication date), yielding a total of 1,667 records. After full-text screening against the predefined inclusion/exclusion criteria (see Section 3.2), 1,035 publications were finally included as the analytical dataset for all bibliometric analyses (publication trends, citation analysis, collaboration networks, and keyword co-occurrence).

To maximize sensitivity and capture all potentially relevant literature in this rapidly evolving field, a broad search strategy using title, abstract, and keyword fields was employed with extensive regeneration-related synonyms. This approach prioritized recall over precision, with subsequent rigorous manual screening (see Section 3.2) applied to remove noise. While this strategy introduced non-specific terms (e.g., “recycling,” “reuse”), it was intentionally designed to improve sensitivity and recall for the heterogeneous and evolving terminology in hair cell regeneration research. We acknowledge that field-specific terms such as “Atoh1” and “supporting cell reprogramming” were not directly included in the search syntax; however, the strategy captured the selected known items from the retrieved records, and the conclusions of this study apply to the resulting regeneration-focused corpus. Importantly, the resulting corpus is best characterized as regeneration-focused: it was identified through the search strategy described above and is not intended to comprehensively represent the full bodies of literature on hair cell protection, cochlear synaptopathy, or neural/synaptic repair, which are recognized as important adjacent domains. The search syntaxes were adapted for each database to account for differences in field codes and indexing conventions. The complete search strings for WoSCC, PubMed, and Scopus are provided in Supplementary material S1 (Search strategy), including all filters and date restrictions applied.

The final literature search and data extraction were performed on February 6, 2026. Citation counts and journal metrics were retrieved on the same date. All searches were conducted on title, abstract, and keyword fields. The search timespan was set from January 1, 2010, to December 31, 2025; however, bibliometric analyses were carried out from 2011 onward. This was because the initial retrieval for the 2010–2025 period yielded 1,667 records, none of which were from 2010 (Supplement: Retrieve screenshots of records and statistics of articles). The exclusion of 2010 was implemented to avoid potential inaccuracies that could arise if the absence of 2010 records were attributable to incomplete indexing in the Web of Science Core Collection for that year, rather than to a genuine absence of relevant publications. This ensured that the analytical dataset aligned with the complete annual coverage of the Science Citation Index Expanded (SCI-EXPANDED) during the period under systematic investigation.

#### 3.1.2 Search strategy validation against sentinel publications

To assess whether our search strategy adequately captured the core literature in the field, we tested it against a pre-defined set of 11 seminal publications that are universally recognized as foundational to inner ear hair cell regeneration research. These sentinel publications were selected based on their demonstrated impact and citation frequency in the field, covering key milestones including:

Notch/Wnt signal regulation (Mizutari et al., 2013; Slowik and Bermingham-McDonogh, 2013).

Inner ear organoids (Koehler et al., 2017).

AAV-mediated gene delivery (Tan et al., 2019).

Multi-gene combinatorial reprogramming (McGovern et al., 2024; Zhang et al., 2024).

Pharmacological regeneration (Lahlou et al., 2024).

This validation confirms that, despite the broad and non-specific nature of some search terms, the strategy achieved adequate sensitivity to capture the foundational and high-impact literature central to the field of inner ear hair cell regeneration Supplementary material (Sentinel publications).

#### 3.1.3 PICOS framework

This systematic bibliometric review was structured according to the PICOS framework as follows:

*Population (P)*: Inner ear hair cells (including outer hair cells, inner hair cells, and vestibular hair cells), supporting cells, ribbon synapses, and spiral ganglion neurons, as represented in published literature.

*Intervention (I)*: Any experimental intervention aimed at hair cell regeneration, repair, protection, or functional recovery, including but not limited to gene therapy (e.g., Atoh1, AAV vectors), stem cell/organoid approaches, small-molecule modulators (e.g., Notch/Wnt inhibitors), biomaterials, and pharmacological agents. For protection and synaptic-repair studies, these were included only when explicitly connected to regeneration or functional recovery within the regeneration-focused literature corpus.

*Comparator (C)*: For bibliometric and comparative analyses, comparisons were made across: (1) different research themes and technological approaches (e.g., gene therapy vs. stem cell approaches; Wnt vs. Notch signaling); (2) different time periods (early 2010s, mid-2010s, and late 2010s onward) to examine thematic shifts; and (3) different countries/regions and institutions to analyze productivity, citation impact, and collaboration networks. No experimental intervention–control group comparison was applicable given the methodological nature of this study.

*Outcome (O)*: (1) Hair cell regeneration or supporting cell transdifferentiation; (2) ribbon synapse formation or repair; (3) measurable improvement in auditory or vestibular function; (4) molecular or cellular evidence of regeneration-associated pathways; and, at the bibliometric level, (5) publication trends, citation impact, keyword co-occurrence patterns, and collaborative network structures.

*Study design (S)*: Original research articles (*in vivo*, *in vitro*, or ex vivo) and peer-reviewed review articles that explicitly report primary data or synthesize evidence on inner ear hair cell regeneration and repair. Conference abstracts, editorials, book chapters, case reports, and non-English publications were excluded.

### 3.2 Data processing and bibliometric analysis

To ensure the precision of included publications, the literature was required to meet the following criteria: the research subject must be inner ear hair cells (or their key components, such as ribbon synapses), and the research objective must involve active intervention or exploration of repair, protection, regeneration, or functional recovery processes following injury. Publications that merely described normal physiology, developmental mechanisms, or injury pathology per se, or that only provided general research tools without addressing specific regenerative/reparative interventions, were excluded. English-language publications retrieved from the Web of Science were systematically screened and cleaned. Based on these principles, the following criteria were established for manual screening.

#### 3.2.1 Inclusion criteria

The research subject must be inner ear hair cells, their progenitor cells (e.g., supporting cells), or their connections with auditory nerves (ribbon synapses). The research objective must directly involve at least one of the following aspects, a primary emphasis on regeneration:

a) Demonstration of *de novo* hair cell regeneration or supporting cell transdifferentiation (e.g., via Atoh1-mediated reprogramming, stem cell differentiation, or gene editing).
b) Investigation of key molecular or cellular mechanisms underlying hair cell regeneration (e.g., Notch and Wnt signaling, cell cycle regulation, or epigenetic modulation).
c) Demonstration of protective effects on hair cells or synapses in injury models, where protection is framed as a strategy to preserve cellular substrates for potential regeneration.
d) Demonstration of synaptic or neural repair following hair cell regeneration or as a complementary strategy for functional recovery.

#### 3.2.2 Exclusion criteria

Studies were excluded if they met any of the following criteria:

a) Research focus solely on normal development, physiological or biochemical characteristics, or ultrastructural description of hair cells without addressing injury and repair.
b) Research focus solely on the methodological establishment of injury or disease models without subsequent application to regeneration/repair interventions.
c) Research focus solely on the development and characterization of general technologies, tools, or materials (e.g., novel microscopes, fluorescent dyes, nanocarriers), even if illustrated using inner ear cells, where the scientific objective is not directed toward solving regeneration biology problems.
d) Research focus on compensatory mechanisms in central auditory pathways or alternative devices such as cochlear implants, rather than biological repair.

For borderline cases (e.g., fundamental studies characterizing potential progenitor cells, or development of tissue engineering scaffolds), individual assessment was conducted based on whether the research explicitly served regenerative medicine objectives or whether its application potential was validated in regeneration models. Representative case assessments were documented in the Appendix to enhance the transparency of the screening process.

**Figure 2:**
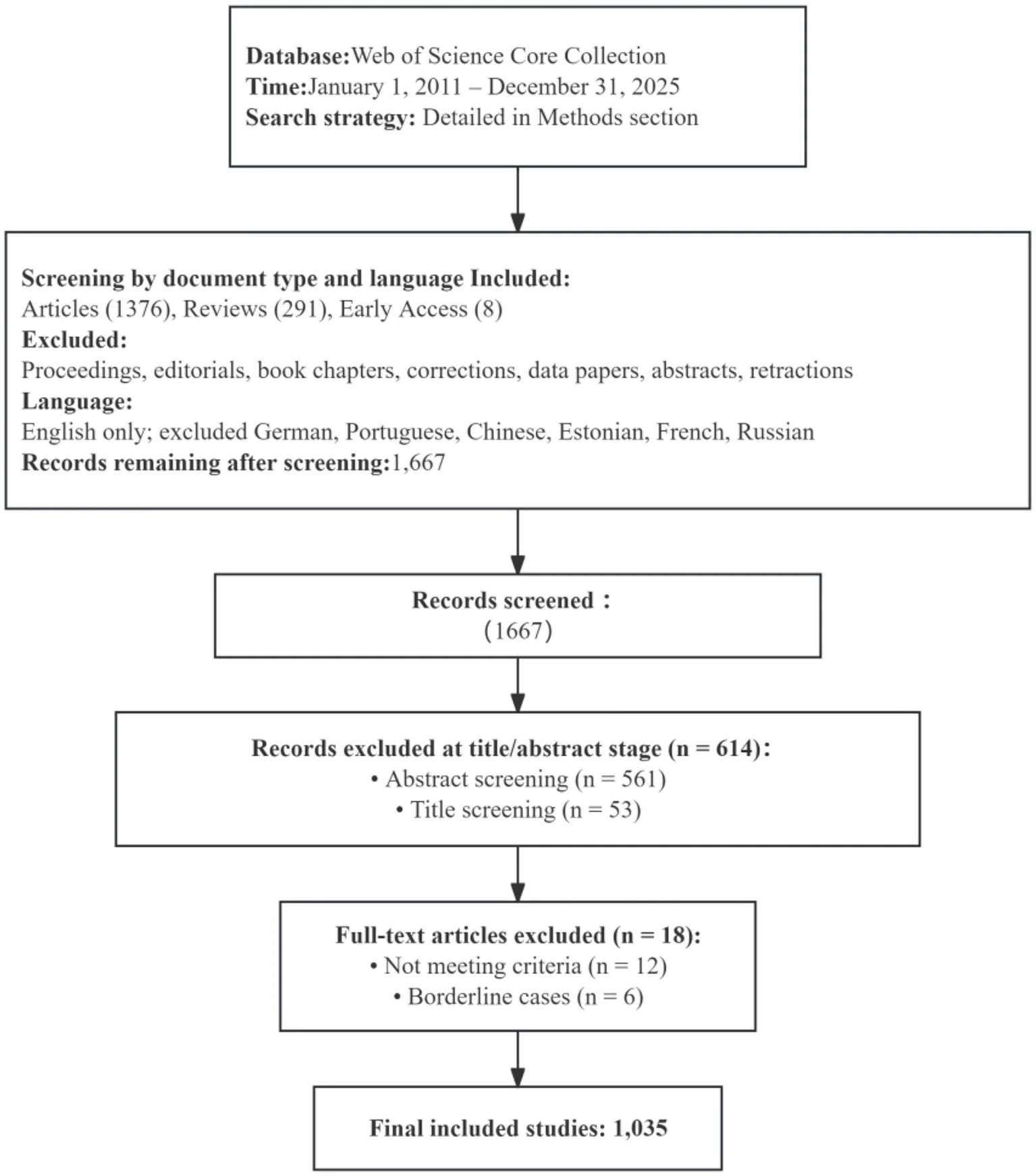
PRISMA flow diagram of the literature selection process. (Early access records overlap with the article/review categories, as they represent publication status rather than an independent document type; the sum of these categories exceeds the total unique records due to this overlap).

Selection Process: Screening was independently performed by two researchers. Disagreements were resolved through discussion or arbitration by a third researcher. The final process of forming the included literature set is illustrated in the literature search flowchart (Figure 2), [Supplementary material S2 (Reasons for exclusion)].

### 3.3 Assessment of risk of Bias

Given the bibliometric nature of this study, conventional risk-of-bias tools for interventional studies (e.g., Cochrane RoB 2) are not directly applicable. Instead, we assessed potential biases at four levels.

1. Database and search bias. To minimize single-source bias, we cross-validated findings using three independent databases: Web of Science Core Collection (primary), Scopus, and PubMed. Overlap rates calculated based on the initial retrieval sets before manual screening, were 82.65% for PubMed and 75.76% for Scopus relative to WoSCC. Independent analysis of non-overlapping records (166 unique publications) suggested that no single database qualitatively altered the main trends observed in the WoSCC-only analysis regarding publication patterns and keyword dynamics (see Section 5. Cross-Database Validation).
2. Publication and language bias. The search was restricted to English-language peer-reviewed articles, excluding conference abstracts, editorials, and non-English publications. This may introduce a language bias and may underrepresent regional or non-English research contributions. However, this is standard practice in bibliometric analyses of this field and ensures consistency across the three databases.
3. Citation and time-accumulation bias. Citation-based metrics (e.g., average citations per paper) inherently favor older, foundational studies (e.g., early Atoh1 research) and may underestimate recent high-impact advances (2022–2025). To mitigate this, we supplemented citation analysis with keyword burst detection using CiteSpace, both of which are less sensitive to accumulation time and better capture emerging trends.
4. Selective outcome reporting bias. Bibliometric analyses rely exclusively on published literature. Negative or null results that remain unpublished are systematically omitted. This limitation is inherent to all bibliometric studies and is acknowledged as part of this review’s limitations.

### 3.4 Statistical analysis and visualization tools

Formal Bradford zone partitioning analysis was performed to quantify journal distribution patterns based on the final dataset of 1,035 eligible publications. All source journals were sorted in descending order according to the number of included articles. The total volume of publications (1,035 papers) was equally divided into three segments of 345 papers each to demarcate core, related, and peripheral zones. We sequentially accumulated article counts from the highest-yield journal to determine the boundary of each zone, then counted the number of journals within each zone to verify whether the distribution conformed to Bradford’s geometric progression rule. Two visualization software tools were employed in this study: CiteSpace (version 7.0. R0) (Chen et al., 2009) and VOSviewer (version 1.6.20) (van Eck and Waltman, 2010). VOSviewer: author, country, and keyword co-occurrence networks; clustering; and overlay visualization. CiteSpace: keyword burst detection reported in Table 1. The combined use of these two tools allows for a more comprehensive exploration of hot topics, emerging trends, and research evolution in the field of inner ear hair cell regeneration and repair.

**Table 1:** Top 20 keywords with the strongest citation bursts, 2011–2025.

Keywords	Year	Strength	Begin	End	2011–2025
Guinea pig	2011	11.96	2011	2013	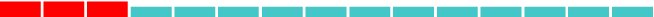
In vivo	2011	5.63	2011	2017	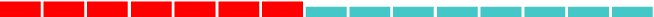
Sensory epithelium	2011	4.84	2011	2013	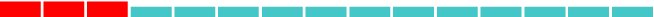
Retinoblastoma protein	2012	4.93	2012	2014	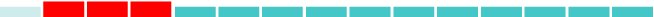
System	2012	3.2	2012	2013	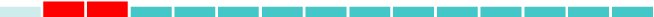
Avian inner ear	2012	3.16	2012	2014	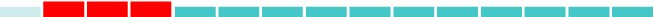
Zebrafish lateral line	2011	3.4	2015	2019	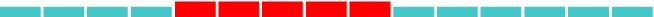
Inner ear development	2015	3.06	2015	2018	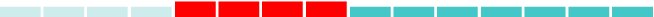
Pathway	2016	5.66	2016	2021	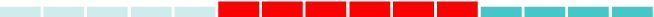
Oxidative stress	2012	3.44	2017	2019	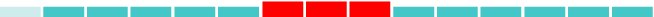
Receptor	2018	3.17	2018	2019	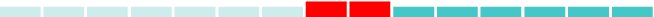
Sox2	2012	3.1	2018	2020	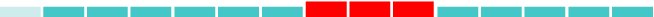
Sensory hair cells	2013	3.04	2018	2019	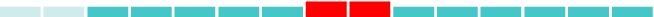
Synaptopathy	2019	3.75	2019	2023	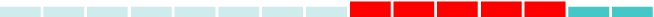
Transplantation	2012	2.98	2019	2021	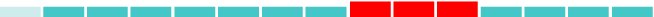
Protection	2014	3.28	2020	2025	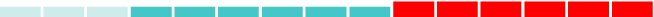
Cisplatin induced ototoxicity	2011	3.58	2021	2022	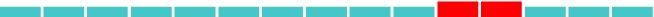
Replacement	2021	3.16	2021	2022	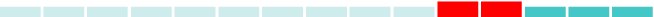
Autophagy	2022	3.04	2022	2025	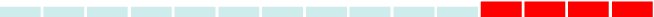
Disease	2023	3.66	2023	2025	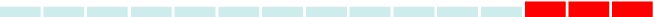

[The number of states:2; (0,1):1.0; minium duration:2; burst items found:29].

#### 3.4.1 Parameter settings for bibliometric analyses

All bibliometric analyses were performed using VOSviewer (version 1.6.20) and CiteSpace (version 7.0. R0). The specific parameter configurations for each software are detailed below to ensure reproducibility.

##### 3.4.1.1 VOSviewer settings

**Figure 3:**
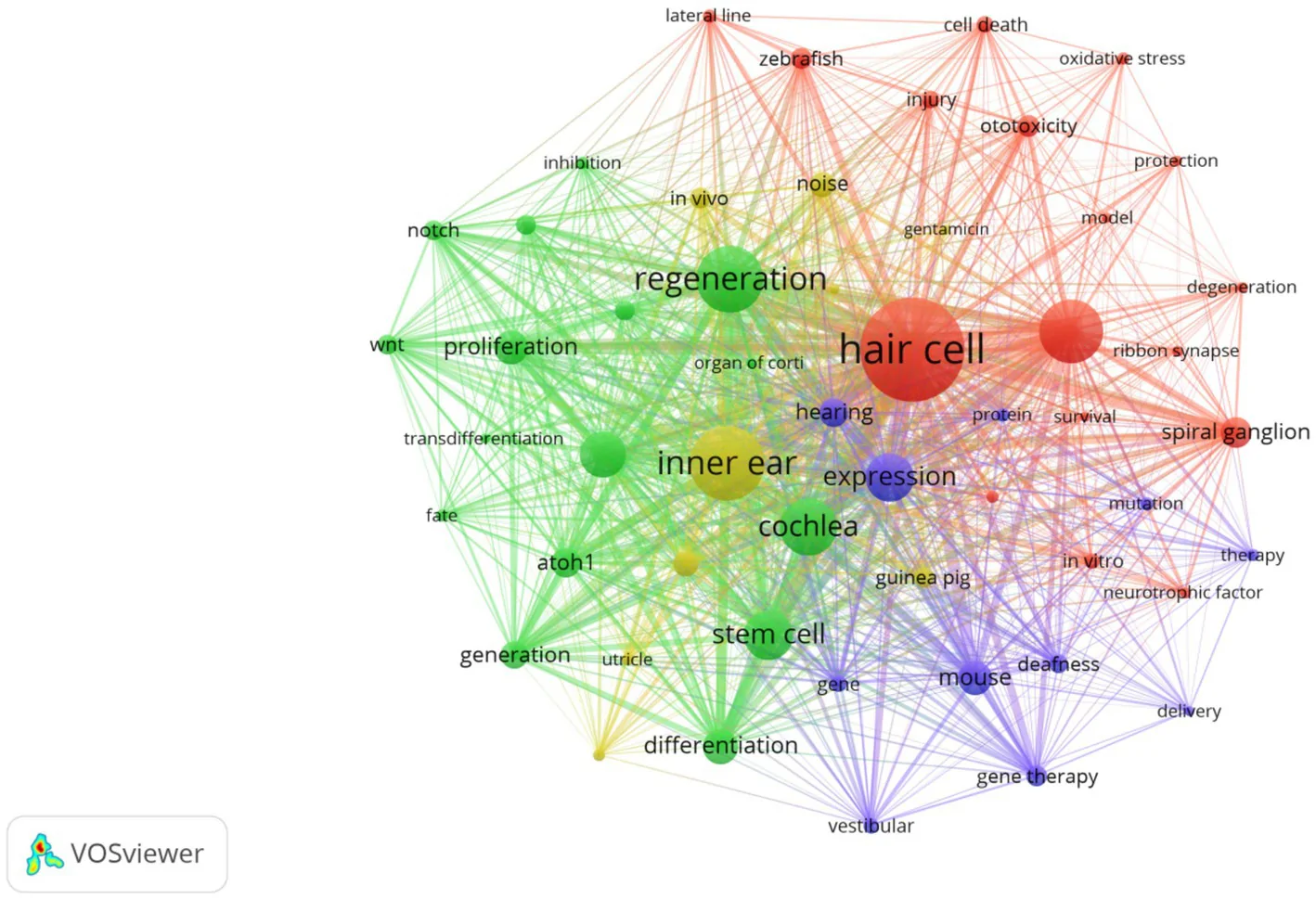
Keyword co-occurrence network visualization (Keywords co-occurrence network. Node size represents keyword frequency; edge thickness represents co-occurrence intensity. Color represents the topic clustering identified by VOSviewer clustering algorithm. Keyword co-occurrence network visualization. This static network map illustrates the core knowledge architecture. Node size represents keyword frequency, and edge thickness represents co-occurrence intensity. Colors denote distinct thematic clusters identified by the VOSviewer algorithm: the red cluster (regeneration and signaling), the blue cluster (stem cell and gene regulation), and the green cluster (translational otology and pathology). This visualization helps to identify the main research modules and their interconnections. Scale: 1.52, weights: occurrences, size variation: 0.79, max.length: 30, font: Open Sans, size variation: 0.91, min.strength: 0, max.lines: 1000).

For author co-authorship analysis, country collaboration analysis, and keyword co-occurrence analysis, the following parameters were applied. The counting method was set to full counting. For the author co-authorship analysis, documents with more than 25 authors were excluded to avoid distortion from hyper-authored publications. The minimum number of documents for an author to be included was set to 5, and the minimum number of documents for a country to be included was set to 5. For keyword co-occurrence analysis, the minimum number of occurrences for a keyword to be included was set to 30. The normalization method used was the default association strength measure. Detailed parameter configurations are provided in the legend of Figure 3. Visualization maps were generated with the default layout settings.

##### 3.4.1.2 CiteSpace settings

For keyword burst detection (reported in Table 1), the following parameters were configured in CiteSpace. The time slicing was set from 2011 to 2025, with 1 year per slice. Term sources included titles, abstracts, author keywords, and Keywords Plus. The node type was selected as keyword. The selection criterion was set to Top *N* = 50 per slice. For burst detection, the default gamma parameter (*γ* = 1.0) was used (Chen, 2006). The keyword merging strategy is consistent with that used in VOSviewer.

##### 3.4.1.3 Data cleaning and normalization

Country names were standardized using a thesaurus file in VOSviewer. Regional designations (e.g., Taiwan, Hong Kong, and Macau) were consolidated under China, and United Kingdom variants (England, Scotland, Wales, Northern Ireland, UK, Great Britain) were unified as United Kingdom. Keyword variants were harmonized using a thesaurus file in VOSviewer. Plural and hyphenated forms (e.g., hair cells, hair-cell, hair-cells → hair cell; hearing-loss → hearing loss; inner-ear → inner ear), species terms (mice、mouse model → mouse), and plural forms of cell types (supporting cells → supporting cell) were consolidated to the singular or standard form. The complete thesaurus files are provided in Supplementary material S3 (Thesaurus).

##### 3.4.1.4 Counting method and interpretation

All bibliometric analyses, including country-level and institutional productivity assessments, were performed using full counting. Under this approach, a publication with authors from multiple countries is counted once for each participating country. Consequently, country-level counts reflect the total number of country-affiliated publication occurrences rather than mutually exclusive unique publications. This methodological choice is standard for collaboration network analysis and was applied consistently across all productivity and network metrics.

## 4 Results

**Figure 4:**
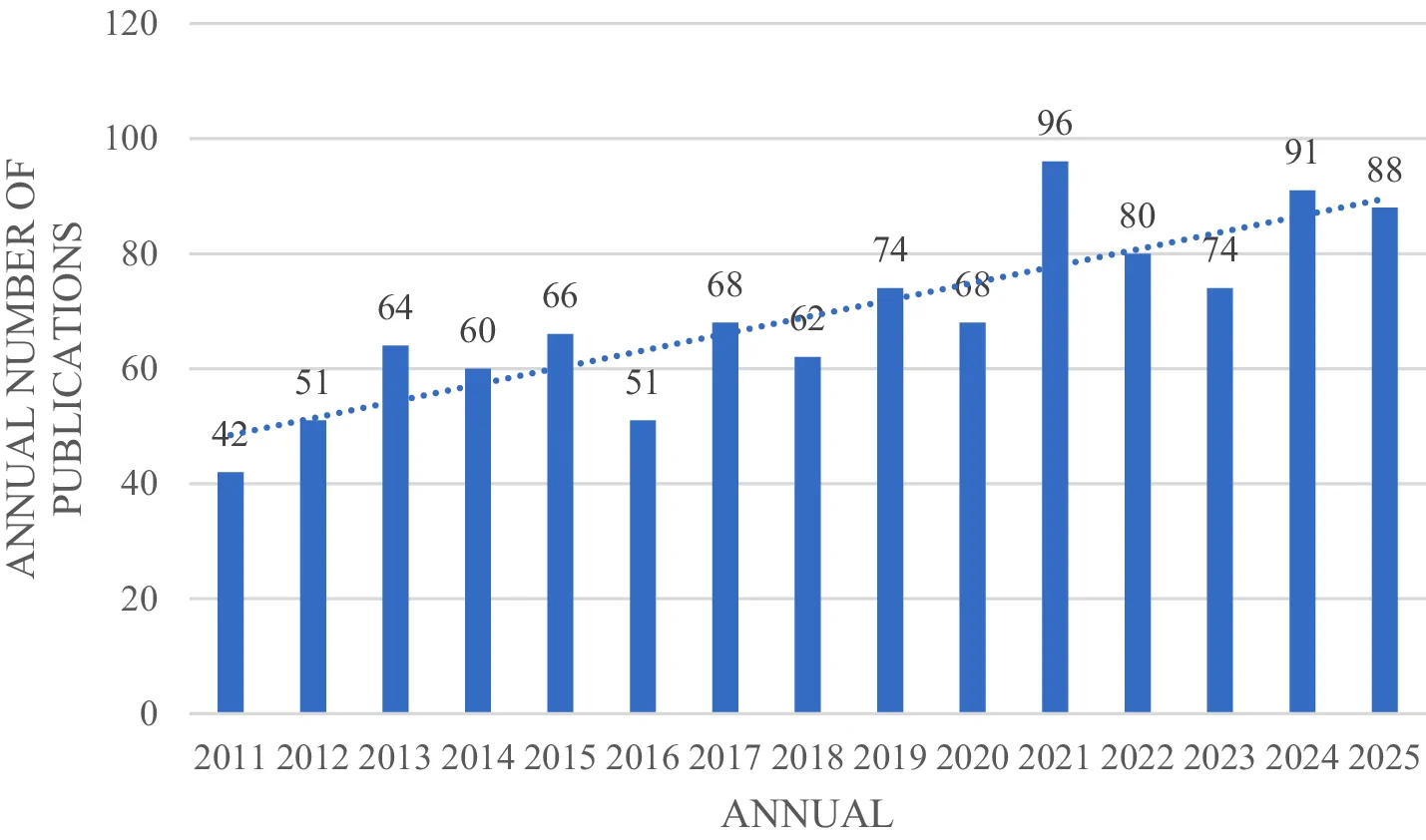
Annual publication trends in the field of inner ear hair cell regeneration and repair based on the final analytical dataset (*n* = 1,035).

**Figure 5:**
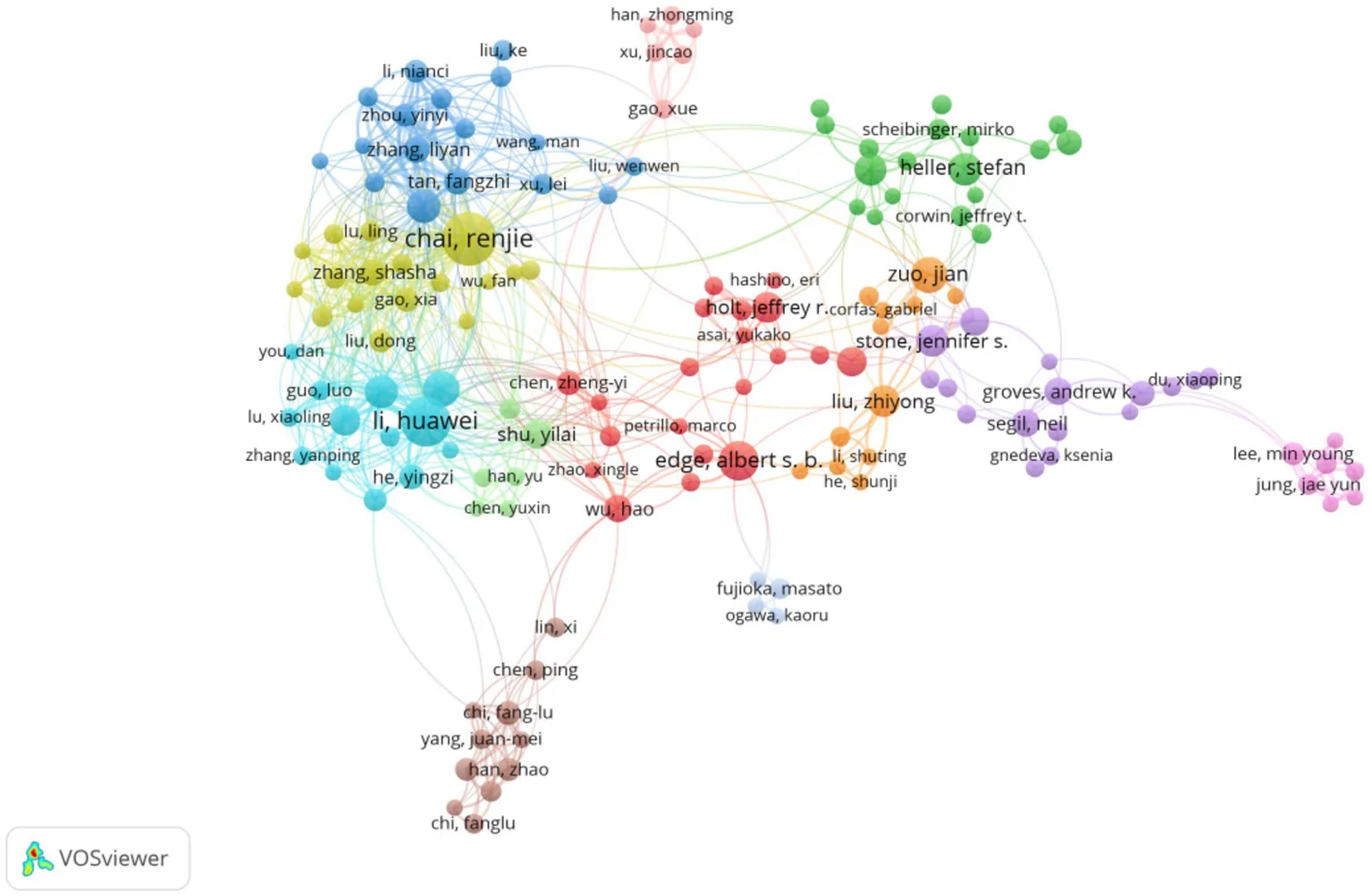
Author collaboration network (Author cooperation network based on co-author analysis. Node size represents the publication count; Edge thickness represents cooperation strength. Only authors who have published more than 5 papers are included. Visualization generated by VOSviewer).

**Figure 6:**
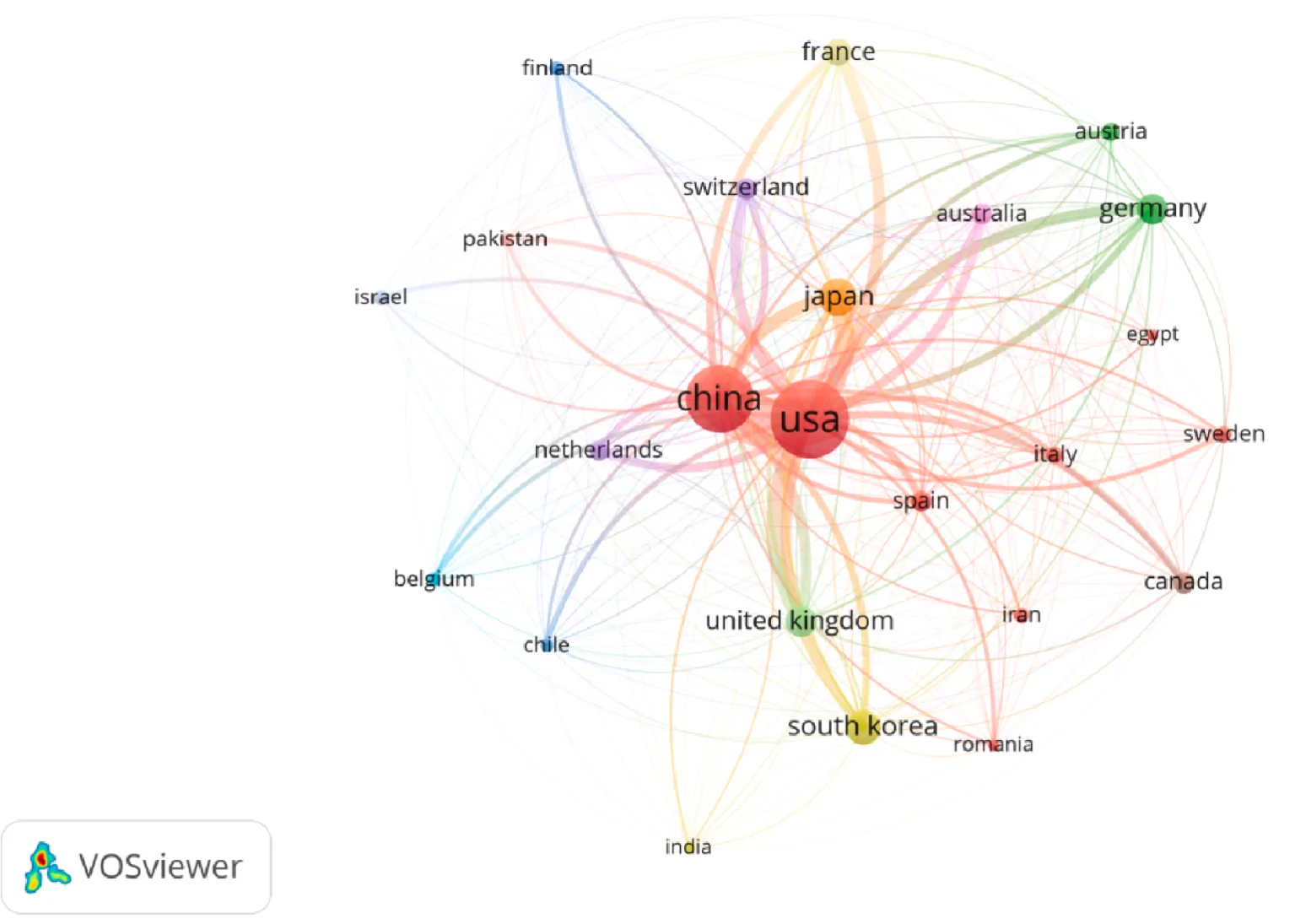
Country collaboration network (original network visualization) (National cooperation network based on co-author analysis. Node size represents the publication count; Edge thickness represents cooperation strength. Only countries with publication number ≥ 5 are included. Visualization generated by VOSviewer).

**Figure 7:**
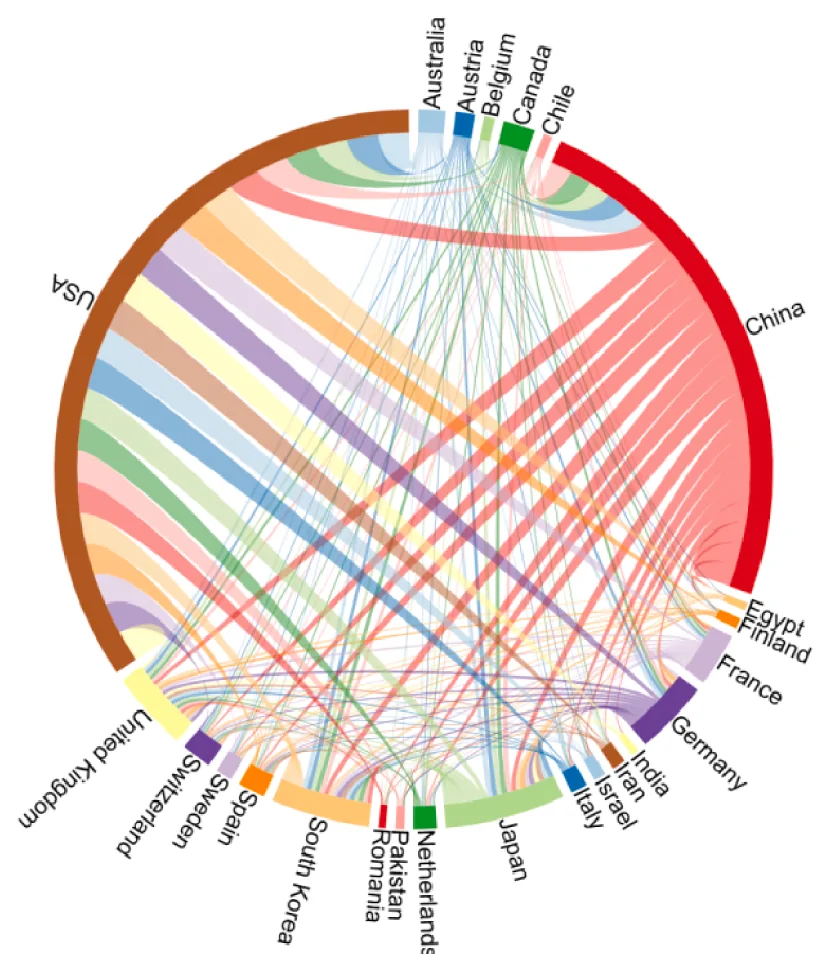
Country chord diagram (circular flow visualization of collaborations). Figure 7 World map visualization of countries with more than five publications (Supplementary material).

**Figure 8:**
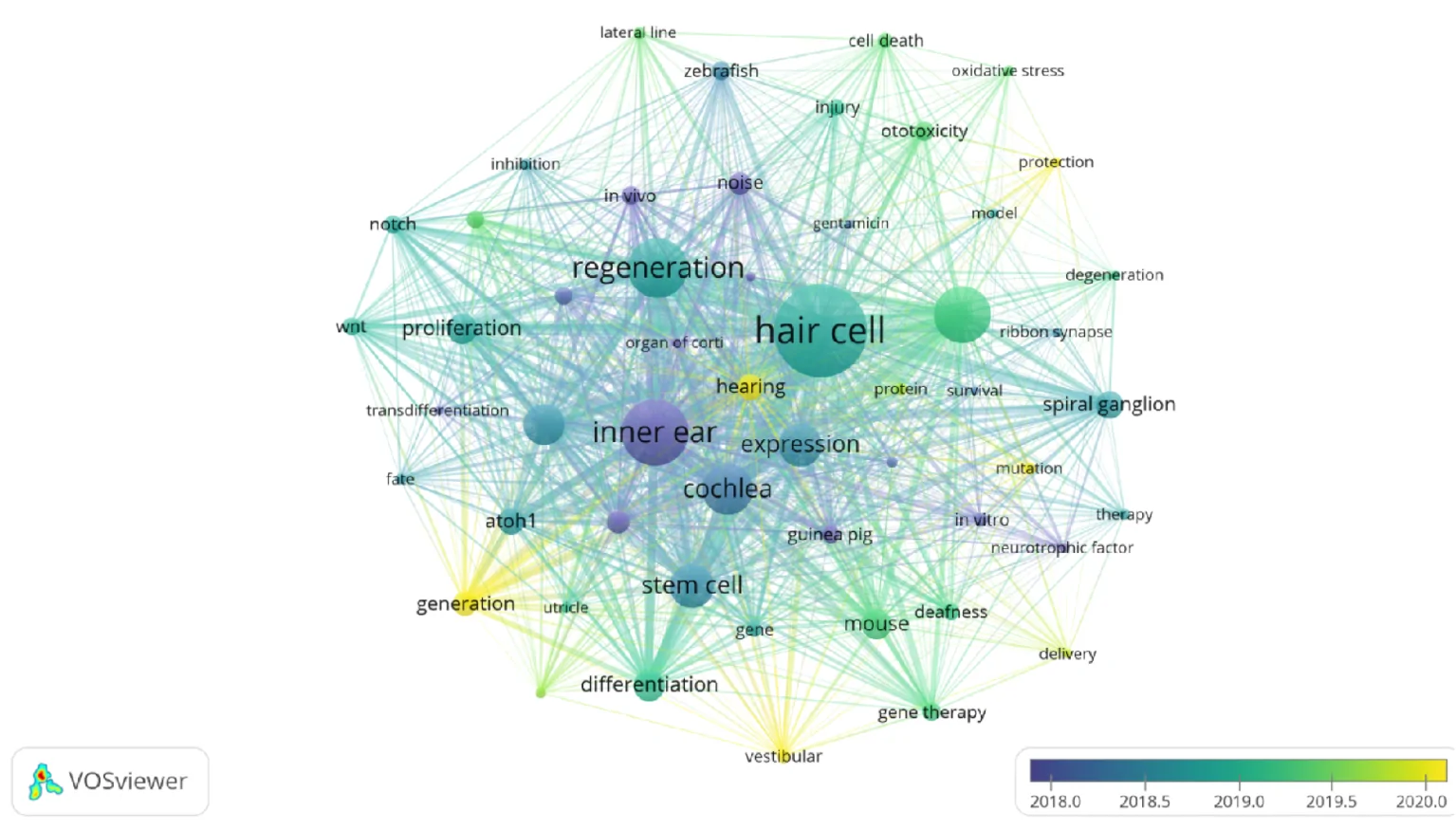
Keyword co-occurrence overlay visualization (keyword co-occurrence overlay visualization, This overlay network visualizes the temporal dynamics of the same keyword co-occurrence data. Node color represents the average publication year of the keyword (see gradient legend), with blue/purple indicating earlier emergence and yellow/red indicating more recent research focus. This visualization tracks the evolution of research interests over time, from foundational mechanisms towards translational applications. Consistent with the parameters in Figure 3).

PRISMA flow diagram of the literature selection process. After initial screening by document type and language, 1,667 records remained. Following full-text screening against the predefined inclusion/exclusion criteria, 1,035 publications were finally included. All subsequent bibliometric analyses (Figures 4–8, Tables 1–5) were performed on this final dataset of 1,035 publications [Supplementary material S4 (PRISMA_2020_checklist)].

### 4.1 Annual publication trends

After initial screening by document type and language, 1,667 records remained. These comprised 1,376 Articles, 291 Reviews, and 8 Early Access records (note: Early Access records overlap with the Article/Review categories, as they represent publication status rather than an independent document type; the sum of these categories exceeds the total unique records due to this overlap). Following full-text screening against the predefined inclusion/exclusion criteria, 1,035 publications were finally included. Figure 4 illustrates a sustained increase in research activity in the field of inner ear hair cell regeneration and repair from 2011 to 2025 based on the 1,035 included publications, with a particularly notable surge around 2021. Annual publications rose substantially from 68 to 96 during this period and subsequently maintained a high level exceeding 80 papers per year. This marked growth coincides temporally with the increasing applications of gene editing technologies (e.g., CRISPR-Cas9) and stem cell/organoid culture systems in this field (Razavi et al., 2024; Zhou et al., 2024). However, publication trends alone cannot establish a direct causal attribution; this increase may also reflect broader funding availability, growing awareness of hearing loss burden, and the maturation of foundational knowledge in the preceding decade. Concurrently, the expanding global population with hearing impairment and aging trends have prompted increased attention from both academia and the medical community toward translational research in this direction, establishing it as a frontier hotspot at the intersection of auditory medicine and regenerative medicine. (Figure 4).

### 4.2 Author analysis

Author-level analysis (Table 2) reveals distinct profiles of academic impact among the top five authors. Chai R ranks first in publication volume (48 papers) and total citations (2,225), indicating sustained high productivity across multiple research avenues. Edge ASB and Zuo J have fewer publications (26 and 23, respectively) but substantially higher average citations per paper (56.65 and 62.91), suggesting that their work has achieved high per-study influence. Li H (40 papers, 1,565 citations) and Li W (23 papers, 952 citations) occupy an intermediate position, with both substantial productivity and strong total citation counts. These complementary profiles illustrate the coexistence of high-productivity and high-impact research strategies within the core author community, reflecting a diversified landscape of research contributions.

**Table 2:** Top 5 authors ranked by number of publications.

Rank	Author	Documents	Citations	Average citation/publication
1	Chai R	48	2,225	46.35
2	Li H	40	1,565	39.13
3	Edge ASB	26	1,473	56.65
4	Li W	23	952	41.39
5	Zuo J	23	1,447	62.91

### 4.3 Journal analysis

Journal-level analysis reveals a three-tiered structure in knowledge dissemination. The first tier comprises journals with relatively low publication volumes but high average citations per paper, including the Journal of Neuroscience (66.25 citations/paper), PNAS (44.41), and Nature Communications (54.60). These journals are frequently selected for studies achieving high per-paper scholarly impact. The second tier consists of specialized otolaryngology and hearing science journals, with Hearing Research (66 papers, 25.42 citations/paper) as the most prolific journal in the field, followed by JARO (16 papers, 24.25 citations/paper). These journals serve as the primary venues for systematic investigation and in-depth disciplinary exchange. The third tier comprises broad open-access journals including PLOS ONE (49 papers, 26.78 citations/paper), Scientific Reports (28 papers, 29.75 citations/paper), and multiple Frontiers series journals. These platforms, through their broad scope and accessible publication models, accommodate a substantial volume of confirmatory, technical, and exploratory studies.

Bradford’s law zone partitioning was conducted based on the full dataset of 1,035 eligible publications. All journals were sorted in descending order of publication output, and the total papers were equally divided into three segments of 345 articles each to define core, related, and peripheral zones. The core zone contained 10 high-yield journals contributing 331 papers, accounting for 32.0% of all publications. The related zone included 60 medium-output journals with 357 articles (34.5% of total records). The remaining low-volume journals formed the peripheral zone, publishing 347 papers (33.5% of all literature). The three zones contained journals at an approximate ratio of 1:6:36, which broadly approximates the geometric progression (1: n:n^2^) described by Bradford’s law of scattering. The core zone (10 journals) is slightly larger than the classical Bradford core typically observed in more mature, consolidated fields, suggesting that the literature on inner ear hair cell regeneration remains relatively dispersed across a diverse range of journals—a pattern consistent with an active, interdisciplinary research area at a translational stage. The coexistence of these three tiers—low-volume/high-impact specialist and generalist journals, high-volume specialized journals, and high-volume open-access platforms—collectively covers a broad spectrum of knowledge dissemination functions in the field (Table 3).

**Table 3:** High-output journals in inner ear hair cell regeneration research (Tied ranks indicate equal publication counts) journal impact factors and JCR quartiles are based on the 2025 journal citation reports (clarivate analytics).

Rank	Source	Documents	Citations	Average citation/publication	IF	JCR
1	Hearing research	66	1,678	25.42	2.5	Q1
2	PLOS ONE	49	1,312	26.78	2.6	Q2
3	Journal of neuroscience	44	2,915	66.25	4.0	Q1
4	Frontiers in cellular neuroscience	41	988	24.1	4.0	Q1
5	Scientific reports	28	833	29.75	3.9	Q1
6	Proceedings of the national academy of sciences of the United States of America	27	1,199	44.41	9.1	Q1
7	Frontiers in molecular neuroscience	22	464	21.09	3.8	Q2
8	Nature communications	20	1,092	54.6	15.7	Q1
9	International journal of molecular sciences	18	152	8.44	4.9	Q1
10	Development	16	824	51.5	3.6	Q1
10	Frontiers in cell and developmental biology	16	246	15.38	4.3	Q1
10	JARO: journal of the association for research in otolaryngology	16	388	24.25	2.3	Q1

### 4.4 Institutional analysis

Institution-level analysis (Table 4) reveals a concentration of research output among leading academic centers. After thesaurus-based normalization and DOI deduplication, Harvard University ranks first with 88 unique publications and the highest average citations per paper (50.61), reflecting a broad and highly influential research portfolio. Fudan University ranks second with 87 publications (25.25 citations/paper), followed by Southeast University with 55 publications (44.42 citations/paper) and Nantong University with 51 publications (35.16 citations/paper). The presence of three Chinese institutions among the top five suggests substantial research capacity in this field within China’s academic system.

**Table 4:** Top 10 institutions ranked by number of publications (Tied ranks indicate equal publication counts).

Rank	Organization	Documents	Citations	Average citation/publication
1	Harvard University	88	4,454	50.61
2	Fudan University	87	2,197	25.25
3	Southeast University	55	2,443	44.42
4	Nantong University	51	1793	35.16
5	Stanford University	47	1,410	30.00
6	Shanghai Jiao Tong University	41	881	21.49
7	Chinese Academy of Sciences	40	1,144	28.60
8	University of Washington	32	1,472	46.00
9	Capital Medical University	27	709	26.26
10	Washington University in St. Louis	24	772	32.17
10	Kyoto University	24	563	23.46

[Institutional affiliations were normalized using a thesaurus-based consolidation of variant name forms (e.g., all Harvard-affiliated entities including Harvard Medical School, Massachusetts Eye and Ear, and Harvard Stem Cell Institute were grouped into “Harvard University”), and publication counts for each consolidated institution were calculated at the unique-document level based on DOI deduplication to avoid double-counting multi-affiliated publications. Raw affiliation strings were retained for institutions without significant name heterogeneity].

The University of Washington (32 publications, 46.00 citations/paper) and Stanford University (47 publications, 30.00 citations/paper) represent U.S. institutions with high per-paper impact. The Chinese Academy of Sciences (40 publications, 28.60 citations/paper) and Capital Medical University (27 publications, 26.26 citations/paper) also appear among the top 10, indicating diverse institutional participation. Overall, the institutional landscape shows a mix of high-volume Chinese institutions and high-impact U.S. institutions. Harvard University and Southeast University demonstrate both high volume and high impact, with average citations per paper of 50.61 and 44.42, respectively, among the top five institutions.

### 4.5 Country analysis

Country-level analysis (Table 5) reveals a markedly skewed distribution of research output and citation impact across nations. The United States leads with 504 publications and also achieves the highest total citations (19,304) and a high average of 38.3 citations per paper. China ranks second with 351 publications but exhibits a lower citation average of 18.75 per paper. Japan ranks third with 79 publications and an average of 26.46 citations per paper. South Korea ranks fourth with 65 publications and an average of 14.43 citations per paper. The United Kingdom ranks fifth with 53 publications but achieves the highest average citations per paper (39.34) among the top five countries, demonstrating that moderate publication volume can nonetheless yield high per-paper scholarly influence.

**Table 5:** Top 5 countries ranked by number of publications.

Rank	Country	Documents	Citations	Average citation/publication
1	USA	504	19,304	38.3
2	China	351	6,583	18.75
3	Japan	79	2090	26.46
4	South Korea	65	938	14.43
5	United Kingdom	53	2085	39.34

This skewed distribution—where a small number of countries dominate both productivity and citation impact, while the majority contribute marginally—is consistent with the general pattern of scientific concentration observed across many research fields. Although the United Kingdom ranks fifth in the number of publications with only 53 papers, it achieves the highest average citations per paper among the top five countries, suggesting that scholarly influence is determined by a combination of factors, not merely by productivity.

(Figure 6) and (Figure 7) collectively depict the international collaboration landscape from two complementary perspectives. Both visualizations consistently show the United States and China as the most prominent nodes in terms of publication volume and network centrality. In Figure 6, the U.S. exhibits extensive collaborative links; in Figure 7, the U.S.–China chord is visibly thicker than all others, visually demonstrating an exceptionally robust bilateral partnership between these two nations. Both figures also reveal a pronounced core–periphery structure in the collaboration network. A small group of countries (United States, China, United Kingdom, Germany, and Japan) exhibits high publication volumes and extensive collaborative links, while most nations occupy peripheral positions. In Figure 7, the top five countries by publication volume occupy the majority of the circumferential arc, with lower-publishing countries compressed into narrow segments.

Geographically, both visualizations show two major regional clusters. Europe exhibits a dense, multidirectional sub-network. The Asia-Pacific cluster, centered on China, Japan, and South Korea, shows a more hierarchical structure with China as the dominant hub. Together, Figures 6 and 7 illustrate the concentration of collaborative activity within a small group of high-output countries.

Analysis of national publication data reveals a markedly uneven distribution of research output in this field. The United States ranks first with 504 publications, substantially ahead of other countries, followed by China (351 publications), Japan (79 publications), the United Kingdom (53 publications), and South Korea (65 publications). In contrast, the majority of countries have contributed few publications, with some nations producing only one or two papers. When countries are ranked in descending order of publication count, a small group of high-output countries (United States, China, Japan, UK, South Korea) accounts for the vast majority of research output, while most countries fall within peripheral positions with limited publications. This concentration pattern is consistent with the general bibliometric principle that a small number of core entities contribute the majority of resources in a given field (Bradford, 1934).

Further analysis incorporating the global scientific collaboration flow map [Supplementary material S5 (Supplementary image)] reveals that this uneven pattern extends beyond publication output to shape the spatial structure of collaborative networks. Collaborative flows are highly concentrated along the trans-Pacific axis centered on the United States, China, Japan, and South Korea, forming a hierarchical structure in which a small number of core countries dominate collaborative connections. Although emerging economies are gradually integrating into global networks, their collaboration patterns remain predominantly characterized by participation in networks led by core countries, rather than independently led regional collaborative clusters.

### 4.6 Keywords analysis

Keyword analysis the keyword co-occurrence network (Figure 3) reveals the core knowledge architecture of this field. The largest nodes—hair cell, regeneration, and inner ear—along with prominent terms including cochlea, stem cell, Atoh1, and spiral ganglion, confirm that hair cell regeneration, cochlear biology, and spiral ganglion neuroprotection constitute the central research themes. Color-coded clustering partitions the landscape into three thematic modules: regenerative mechanisms (e.g., Wnt/Notch, proliferation), stem cell and gene regulation (e.g., Atoh1, expression), and translational pathology (e.g., ototoxicity, gene therapy). The network exhibits strong agglomeration, with a small set of high-frequency keywords dominating the knowledge framework.

The overlay visualization (Figure 8) tracks the temporal evolution of these themes. Persistent foundational hotspots—hair cell and regeneration—maintain stable co-occurrence across all years. Clusters related to cellular regeneration (proliferation, Atoh1, Wnt/Notch) concentrate in earlier periods, whereas nodes linked to translational therapy (gene therapy, neurotrophic factor, delivery) and pathological injury (oxidative stress, ototoxicity) shift toward later years. This gradient reveals a clear trajectory: from single-pathway mechanistic studies toward integrated network regulation and clinical translation, with model organisms (zebrafish, mouse, and guinea pig) serving as consistent experimental carriers throughout.

Table 1 summarizes the top 20 keywords with the strongest citation bursts, systematically outlining the phased evolution of inner ear hair cell regeneration research from fundamental morphological observation to clinical translational treatment. In the early research phase (2011–2017), burst keywords mainly centered on preclinical animal models and basic inner ear structural research: guinea pig possessed the maximum burst strength (11.96, 2011–2013), while *in vivo* maintained a long burst duration spanning 2011 to 2017, alongside sensory epithelium and avian inner ear. These items demonstrate that early work relied heavily on in vivo animal models to characterize the structure and physiology of inner ear sensory epithelia, laying the fundamental research foundation of the field. Intermediate-stage research (2015–2019) shifted focus to molecular signaling and injury pathological mechanisms: keywords such as inner ear development, pathway, and oxidative stress successively generated citation bursts, mirroring rising investigations into developmental regulatory pathways and ototoxic injury-induced cell death mechanisms. Meanwhile, the burst of zebrafish lateral line (2015–2019) highlighted the rising utility of zebrafish as a high-throughput regenerative model during this period. Since 2019, the research paradigm has undergone a clear shift toward therapeutic translation and disease intervention. Synaptopathy (2019–2023) captured sustained attention to auditory synaptic damage mechanisms, followed by emerging therapeutic keywords including transplantation, gene therapy-associated targets sox2 and sensory hair cells. After 2020, burst keywords focused on clinical hearing loss treatment: protection sustained its citation burst through 2025, covering otoprotective strategies against acoustic and drug-induced damage; cisplatin induced ototoxicity and replacement reflected growing research on chemotherapeutic drug-induced hearing impairment and hair cell replacement strategies. Most recently (2022–2025), autophagy and disease exhibited prominent late-stage bursts, indicating that current frontier research integrates autophagic regulatory mechanisms into therapeutic schemes for clinical deafness disorders. Overall, the keyword co-occurrence Overlay Visualization depicts a complete research evolution: from establishing animal experimental models and characterizing basic inner ear structures, to dissecting molecular pathways and injury pathology, and ultimately advancing toward disease-targeted otoprotection, hair cell regeneration, and gene therapeutic translation.

**Figure 9:**
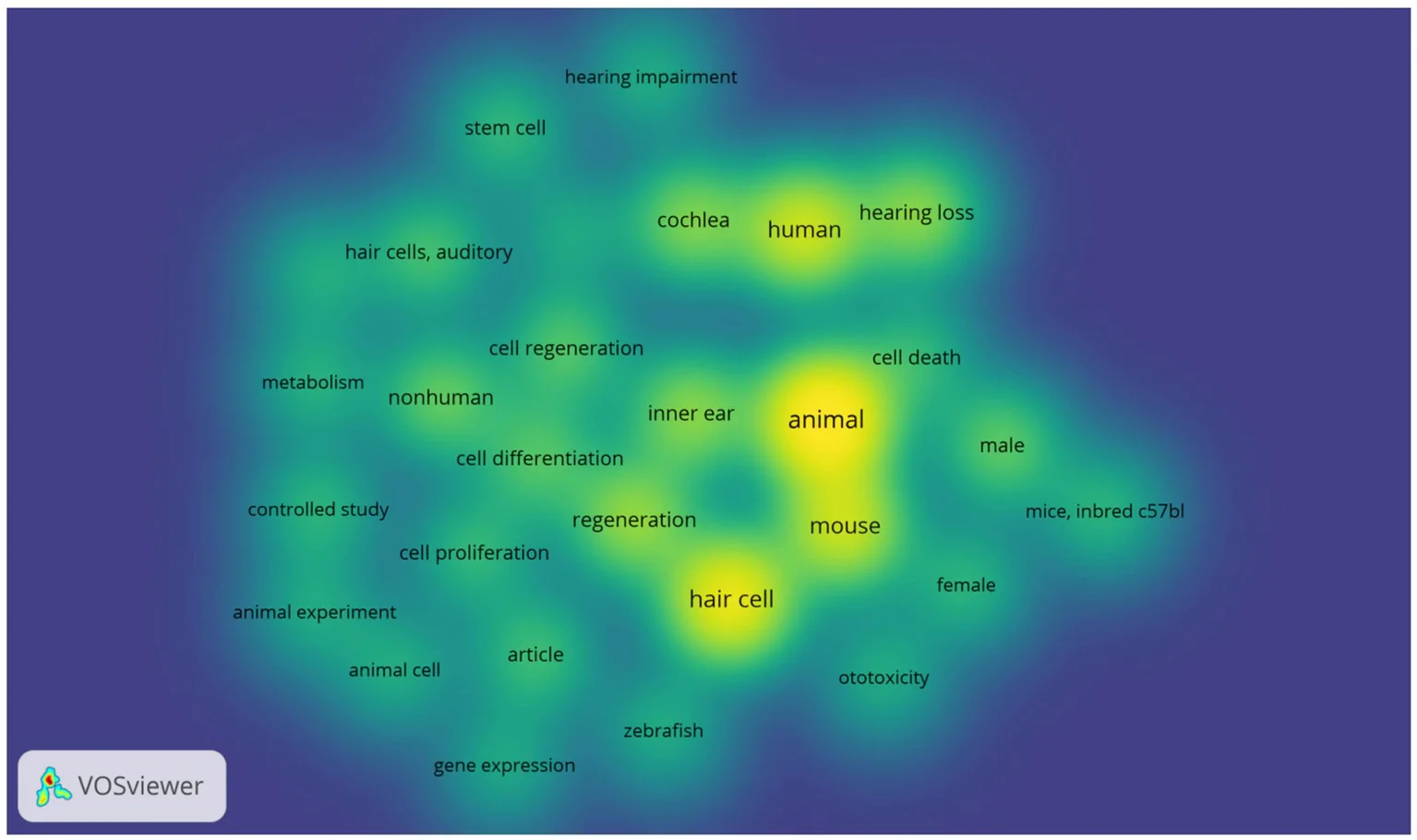
Topic density overview of the 166 unique records from PubMed and Scopus (non-overlapping with WoSCC). This visualization is generated using database-specific indexing fields (e.g., MeSH terms for PubMed), which differ substantially from the author-declared keywords and Keywords Plus used for the main WoSCC analysis (Figures 3, 8). As such, this figure serves as a supplementary high-level thematic overview of the non-overlapping literature subset, rather than a directly comparable keyword co-occurrence network. Minimum number of occurrences of a keyword: 30; density visualization generated by VOSviewer).

A critical caveat is that the keyword visualizations for the unique subsets (Figure 9) were generated using the available metadata fields from each database. For PubMed, this includes substantial MeSH (Medical Subject Headings) terms such as “animals,” “humans,” and “male/female,” which are not directly equivalent to the author-declared keywords or Keywords Plus from the WoSCC dataset (Figures 3, 8). Consequently, Figure 9 is intended to provide a rough high-level thematic overview of the non-overlapping literature, rather than a direct thematic equivalence test against the primary WoSCC network. The observed presence of core scientific terms (e.g., “hair cells,” “regeneration,”) in both datasets, however, suggests broad thematic consistency.

## 5 Cross-database consistency check

### 5.1 Validation rationale and retrieval strategy

To assess the robustness of the findings derived from the Web of Science Core Collection (WoSCC) and to exclude potential single-database bias, we performed confirmatory retrieval using Scopus and PubMed over the same period (2011–2025) with equivalent search strategies. All searches were conducted on title, abstract, and keyword fields using a broad set of regeneration-related synonyms combined with inner ear hair cell terms, restricted to English-language articles and reviews.

### 5.2 Retrieval outcomes and overlap analysis

The initial yields before deduplication were 1,667 records from WoSCC, 1,078 from PubMed, and 232 from Scopus. After intra-database deduplication based on DOI matching (supplemented by title and author matching for records without DOIs), 1,667 WoSCC records, 1,072 PubMed records, and 231 Scopus records remained for cross-database comparison.

The lower Scopus yield (232 vs. 1,078 for PubMed) warrants careful interpretation. Including database-specific indexing practices, differences in field definitions and phrase handling between databases, and the exclusion of conference abstracts (which constitute a substantial proportion of Scopus records in this subject area) by our document type filter (Article/Review only). The precise cause of this yield difference was not determined in this study. The exact Scopus query, including all field restrictions, filters, and search date, is provided in Supplementary material S1 (Search strategy) for full transparency. Despite this lower yield, the Scopus-derived unique records (*n* = 24) were included in the supplementary consistency check to ensure coverage as much as possible.

### 5.3 Overlap analysis revealed the following rates

PubMed overlap rate: 82.65% of PubMed records (886/1,072) were also indexed in WoSCC; Scopus overlap rate: 75.76% of Scopus records (175/231) were also indexed in WoSCC; WoSCC coverage of PubMed: 53.14% of WoSCC records (886/1,667) were also found in PubMed; WoSCC coverage of Scopus: 10.50% of WoSCC records (175/1,667) were also found in Scopus.

These overlap rates indicate that WoSCC alone captures the majority of peer-reviewed literature indexed in PubMed, while the substantially lower WoSCC–Scopus overlap reflects Scopus’s narrower scope in this field rather than a limitation of WoSCC.

### 5.4 Screening and comparative analysis of unique literature

To further examine the core conclusions from WoSCC—particularly regarding keyword trends and author impact patterns—the identical inclusion and exclusion criteria (see Methods, Section 3.2) were applied to the non-overlapping portions of PubMed and Scopus. This process identified 142 unique PubMed records and 24 unique Scopus records, totaling 166 supplementary publications not indexed in WoSCC. The 142 PubMed-unique and 24 Scopus-unique records were cross-deduplicated against each other using DOI matching (supplemented by title and author matching for records without DOIs), resulting in a final set of 166 unique supplementary publications that were used for subsequent analysis. Independent visualization analysis using VOSviewer was performed on this subset, focusing on keyword co-occurrence and author publication distributions.

#### 5.4.1 Publication trends

**Figure 10:**
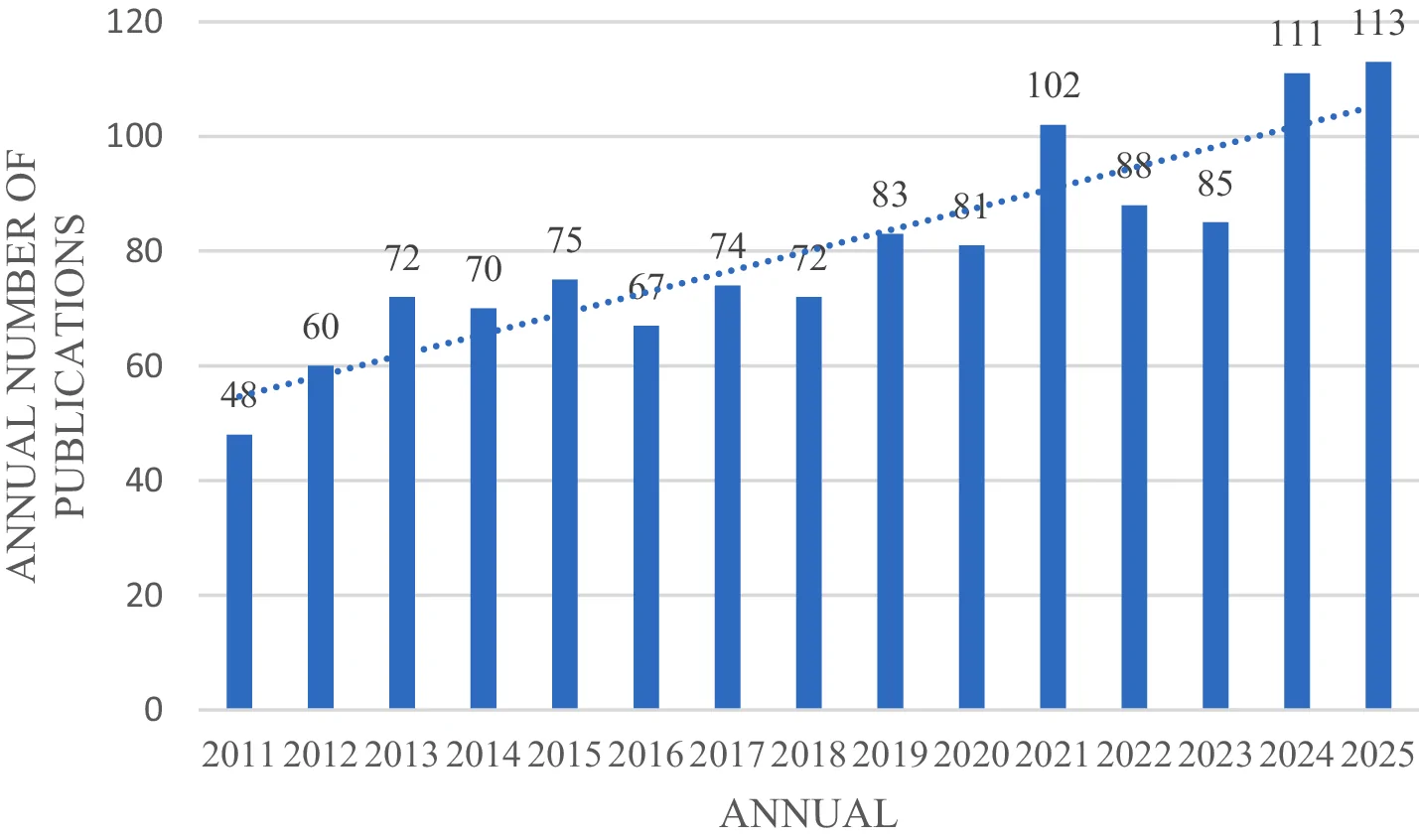
Annual publication trends in the field of inner ear hair cell regeneration and repair: Comparison using three databases (WoSCC, Scopus, and PubMed combined). The description is based on 1,201 merged records and is only used for trend verification.

After merging the 166 unique publications with the WoSCC dataset (total *n* = 1,201), the overall annual publication trend remained consistent with the WoSCC-only analysis (Figure 10). A direct comparison between the merged dataset trend (new Figure 10) and the WoSCC-only trend (Figure 4) reveals a near-identical growth trajectory, including the pronounced surge around 2021, confirming that the primary findings are robust and not qualitatively altered by including literature from other major databases.

#### 5.4.2 Keyword analysis

High-frequency terms in the 166 unique publications were centered on hair cells inner ear cochlea hearing loss and cell regeneration (Figure 9). Other frequent terms included stem cell signal transduction cell differentiation/proliferation gene expression cell death and ototoxicity. Experimental models such as mouse mice inbred C57BL zebrafish and nonhuman animal were also prominent. Notably emerging translational keywords like gene therapy or synaptic reconstruction were absent in this subset likely due to its restricted scope and small sample size. The WoSCC dataset does not exhibit contradictory trends identified within the limited scope of this subset.

#### 5.4.3 Author analysis

Of the 166 distinct publications identified, Chai R (*n* = 9) and Li H (*n* = 6) were the two leading authors with over five publications each. This ranking aligns with the primary WoSCC analysis presented in Table 2. Given the small sample size, this finding should be interpreted as limited descriptive consistency rather than formal validation of the overall author landscape [Supplementary material S6 (Dataset)].

### 5.5 Validation conclusions

Collectively, these supplementary cross-database analyses provide a coverage and trend-consistency check for the primary WoSCC-based findings. The high overlap between WoSCC and PubMed, combined with the absence of overtly contradictory trends in the non-overlapping subsets, suggests that database selection is unlikely to have introduced a systematic bias that would challenge the main conclusions of this study regarding publication trajectories and keyword dynamics. Collaboration network conclusions, however, are derived from WoSCC data and were not independently assessed in the cross-database consistency check. However, it is important to recognize that this analysis is based on initial retrieval sets rather than the final screened corpus, and therefore does not constitute formal validation of the analytical dataset’s completeness. The primary conclusions of this study should be interpreted as derived from WoSCC data, with supplementary support from cross-database consistency checks rather than definitive consistency check.

## 6 Discussion

The following discussion is based on WoSCC data (2011–2025), with supplementary consistency checks using PubMed and Scopus. It should be noted that recent clinical advances in OTOF gene replacement for congenital deafness—published after our search window—have demonstrated that gene replacement in surviving inner hair cells can restore auditory signaling in appropriately selected patients. These developments underscore the growing clinical momentum in inner ear gene therapy, while also highlighting that *de novo* hair cell regeneration and functional integration of regenerated cells remain distinct and clinically unproven challenges. The analysis identifies a temporal evolution of research emphases—foundational structural characterization (early 2010s), followed by signaling pathways (mid-to-late 2010s), and more recently pathological mechanisms and protective/regenerative strategies (late 2010s to present)—reflecting thematic diversification rather than discrete paradigm shifts. The early emphasis (approximately 2011–2014) centered on structural characterization, with bursts of guinea pig, sensory epithelium, *in vivo*, retinoblastoma protein, and avian inner ear establishing the foundational architecture of the mammalian inner ear. The mid-to-late 2010s shifted toward molecular signaling and injury pathology, with sustained bursts of pathway (2016–2021), oxidative stress (2017–2019), zebrafish lateral line (2015–2019), and inner ear development (2015–2018) (Liberman and Kujawa, 2017). More recently (late 2010s to present), an emerging emphasis has turned to pathological mechanisms and protective/regenerative strategies, with synaptopathy (2019–2023), transplantation (2019–2021), protection (2020–2025), cisplatin-induced ototoxicity (2021–2022), replacement (2021–2022), autophagy (2022–2025), and disease (2023–2025)—together reflecting a broadening from basic mechanisms toward translational applications. The progression from early gene therapy studies (Izumikawa et al., 2005) to recent multigene cocktails (McGovern et al., 2024; Zhang et al., 2024) exemplifies the advancement from single-factor induction toward network regulation. Foundational structural research persisted alongside these emerging approaches. Collectively, these overlapping emphases document the field’s maturation and diversification, with multiple themes progressing in parallel rather than in strict succession.

Regarding global research power, the United States leads in volume and total citations (504 papers, 38.3 citations/paper), exemplified by work showing pharmacologically regenerated hair cells restore afferent innervation and vestibular function—representing progress in the synaptic and neural repair paradigm (Lahlou et al., 2024). China ranks second (351 papers), with notable contributions spanning multiple paradigms: the development of AAV-ie vectors has addressed key delivery bottlenecks for inner ear gene therapy (Tan et al., 2019; Wu et al., 2025), while AAV-regulated Serpine2 overexpression has been successfully employed to promote *de novo* hair cell regeneration from supporting cells (Sun et al., 2024). From a cellular neuroscience perspective, the prominence of “oxidative stress” (2017–2019) and “ribbon synapse” (2014–2021) underscores that hair cell damage mechanisms converge on common cellular pathways—including mitochondrial dysfunction and synaptic degeneration—regardless of the initial insult. These pathways represent promising targets for therapeutic interventions aimed at protecting hair cells and their synaptic connections.

From a cellular neuroscience standpoint, translating hair cell regeneration into functional recovery requires addressing several fundamental biological challenges. These challenges can be mapped onto the three paradigms outlined above—hair cell protection, *de novo* regeneration, and synaptic/neural repair—each with its own translational bottlenecks. Despite the promising advances identified by our bibliometric analysis, the field has yet to establish consistent, broadly translatable clinical outcomes. Clinical translation has begun for narrowly defined genetic forms of auditory neuropathy through gene replacement in surviving inner hair cells. The 2025 first-in-human DB-OTO study reported that 9 of 12 children with OTOF-related deafness met the primary and key secondary hearing endpoints, with three achieving average normal hearing sensitivity (Valayannopoulos et al., 2026); a 2026 multicentre AAV1-hOTOF trial involving 42 participants reported hearing recovery in 90% of treated participants with follow-up extending to 2.5 years (Jiang et al., 2026); and on April 23, 2026, the FDA granted accelerated approval to Otarmeni for molecularly confirmed biallelic OTOF-related hearing loss. In contrast, clinically validated de novo hair cell regeneration and durable neural integration of regenerated hair cells into the auditory neural circuit remain unresolved. The gap between preclinical breakthroughs and therapeutic application remains wide, constrained by a combination of biological, technical, and safety-related barriers. The predominance of neonatal animal models and non-mammalian species in the literature (Cruz et al., 1987; Ryals and Rubel, 1988; Smith-Cortinez et al., 2023) further complicates extrapolation to the aged or injured human cochlea, which presents a uniquely challenging environment with limited access, poor vascular supply, and quiescent supporting cells (Berlingeri et al., 2022). The field currently confronts three core challenges that cut across these paradigms. First, functional integration of regenerated or repaired hair cells with existing neural circuitry (synaptic/neural repair paradigm): the recognition of synaptic pathology as a core feature of sensorineural hearing loss (Moser and Starr, 2016) aligns with the growing interest in synaptic repair, and MXene hydrogels that promote both hair cell and synapse formation via mTOR signaling offer a potential pathway (Zhang et al., 2022). However, the functional quality of regenerated synapses—their number, morphology, neurotransmitter release properties, and stability over time—remains largely unexplored. Long-term functional stability beyond short-term endpoints (typically 1–4 weeks post-treatment) has likewise received limited attention, despite the progressive and age-related nature of human hearing loss. Second, safety and delivery limitations across all paradigms: AAV-mediated gene delivery, whether used for gene replacement (protection paradigm), Atoh1 overexpression (regeneration paradigm), or synaptic repair, elicits host immune responses (Ishibashi et al., 2023; Ishibashi et al., 2025); while targeted microRNA editing has shown promise in restoring auditory function (Zhu et al., 2024), long-term safety and cell-type-specific delivery data remain insufficient. Off-target mutagenesis in post-mitotic hair cells, which cannot be replaced if damaged, raises additional safety concerns for gene editing approaches. Third, subtype-specific biology of auditory versus vestibular hair cells (relevant to both protection and regeneration paradigms): Atoh1 expression timing is crucial for proper subtype differentiation (You et al., 2023), epigenetic mechanisms regulate inner ear development (Balendran et al., 2022), and RBM24 is co-localized in both cell types (Shi et al., 2022), suggesting that distinct molecular networks govern each lineage and may require tailored intervention strategies. The heterogeneity of hearing loss etiologies—noise-induced, drug-induced, age-related, and genetic—adds further complexity, as the optimal regenerative strategy likely differs by etiology and the field lacks clear guidance on patient stratification.

Beyond these three core challenges, additional translational barriers warrant acknowledgment. Consistent with the hair cell protection and *de novo* regeneration paradigms, our bibliometric analysis did not systematically separate auditory and vestibular hair cell research, as both lineages share developmental origins and are frequently investigated together in the literature. However, the occurrence of vestibular-focused studies suggests a distinct wave of research in this area, while the overall corpus remains heavily dominated by cochlear (auditory) investigations. This conflation may obscure lineage-specific regulatory mechanisms and translational barriers—particularly given that vestibular hair cells retain greater regenerative capacity in adult mammals than their auditory counterparts, which has implications for both protection and regeneration strategies. Future bibliometric studies with dedicated vestibular filters, or stratified analyses by inner ear compartment, would complement and refine our findings (McGovern and Cox, 2025).

Synthesizing bibliometric trends, future work may increasingly focus on precision gene editing, endogenous supporting cell reprogramming, functional synaptic reconstruction, and efficient delivery systems. Tbx2 regulates inner versus outer hair cell differentiation (García-Añoveros et al., 2022), gene editing restores function in Myo6 models (Xue et al., 2022), and single-cell sequencing dissects transcriptional networks underlying Atoh1-mediated trans differentiation (Tu and Zuo, 2023)—presaging multi-target synergy as core pathways. The bibliometric signals suggest these potential priorities. The field appears to be broadening from a predominant emphasis on cellular regeneration toward increasing attention to functional reconstruction—a trend supported by the growing interest in synaptic mechanism and the more recent emergence of gene therapy-related clusters, although the term ‘synaptic reconstruction’ itself was not detected as a burst keyword in our analysis. Future investigations in cellular neuroscience should focus on three interconnected priorities: (1) deciphering the transcriptional and epigenetic codes that govern hair cell subtype specification; (2) reconstructing functional ribbon synapses between regenerated hair cells and spiral ganglion neurons; and (3) ensuring long-term stability and immune compatibility of regenerated hair cells and their synaptic reconnection with spiral ganglion neurons. Acknowledging these barriers—and the negative or confirmatory studies that are systematically underrepresented in the publication record—is essential for designing the next generation of studies that can bridge the gap between animal model breakthroughs and human therapeutic solutions. Addressing these priorities will propel mechanistic discoveries toward therapeutic solutions for sensorineural hearing loss.

## 7 Limitations

This study has five main limitations. First, although we performed cross-database validation using Scopus and PubMed to minimize database bias, the primary bibliometric analysis was still based on the Web of Science Core Collection. Non-English and regional journals remain underrepresented across all major databases, which may affect the generalizability of the findings. Second, bibliometric analysis quantifies publication activity and citations, not scientific validity; therefore, rigorous negative results and well-controlled studies that did not achieve significance are systematically omitted. Third, citation-based metrics are subject to time accumulation effects, artificially elevating older foundational work (e.g., early Atoh1 studies) while underestimating recent high-impact advances (e.g., gene editing from 2022–2025). Fourth, the final literature search was performed on February 6, 2026. Due to the inherent delay in database indexing, late-2025 publications may not yet have been completely indexed in the Web of Science Core Collection at the time of retrieval. Consequently, the 2025 publication count should not be interpreted as necessarily complete, and the analytical dataset reflects the state of WoSCC indexing as of the search date. Fifth, keyword burst detection identifies statistical surges that may occasionally reflect transient trends rather than genuine paradigm shifts. Additionally, the search strategy was designed to be highly sensitive for regeneration-related literature. While this approach successfully captured the core literature on inner ear hair cell regeneration—as validated by the sentinel publication test (see Section 3.1.1)—it may not have fully captured all publications specifically focused on hair cell protection or synaptic repair that do not use regeneration-related terminology. Therefore, our findings and conclusions are most directly applicable to the regeneration-focused literature corpus and should be interpreted with this scope in mind. Nevertheless, these limitations do not invalidate the descriptive results reported in this study; however, they do constrain the strength and generalizability of the thematic inferences drawn from the bibliometric data. Multiple lines of convergent evidence—including publication trajectories, collaboration networks, and keyword co-occurrence clustering—support the observed prominence of the United States and China in publication volume and network centrality, the three overlapping yet chronologically prominent thematic phases, and the emerging focus on synaptic reconstruction. This convergence makes it highly unlikely that these patterns arise solely from database bias, citation accumulation, or transient keyword surges.

## 8 Conclusion

Over the past 15 years, research centered on inner ear hair cell regeneration has undergone a progressive evolution from mechanistic dissection toward multi-pathway intervention, establishing a global landscape characterized by the United States and China as the two dominant contributors in terms of publication volume and collaborative centrality, along with synergistic collaboration among multiple countries. Keyword burst detection and cluster analysis jointly reveal that the field has broadened substantially: while *de novo* hair cell regeneration remains a core theme, our integrative interpretation of these bibliometric signals points toward increasing attention to functional reconstruction—encompassing synaptic repair, neural preservation, and the integration of regenerated hair cells with existing auditory circuitry—as an emerging translational objective. Within this evolving landscape, gene-based therapeutic strategies have become central across multiple paradigms: as tools for hair cell protection (e.g., gene replacement to preserve existing cells), for supporting cell reprogramming (e.g., Atoh1-mediated trans differentiation), and for synaptic repair (e.g., restoring ribbon synapse function). Novel delivery systems have addressed key barriers to inner ear drug administration, though safety and subtype-specificity remain critical challenges. From a cellular neuroscience perspective, an increasingly important translational question is how regenerated hair cells can establish functional connectivity with spiral ganglion neurons.” However, the delayed accumulation of immunological safety evidence and insufficient investigation into the subtype specificity of auditory versus vestibular hair cells remain weak links in the current landscape. The focus of the next phase should center on addressing the functional differences between these two hair cell types by overcoming fundamental bottlenecks in synaptic reconstruction, neuronal integration, and immune compatibility, thereby transforming mechanistic breakthroughs into a comprehensive cellular neuroscience framework for sensory repair.

## Data Availability

Publicly available datasets were analyzed in this study. This data can be found here: the data that support the findings of this study are openly available in Fig share at https://doi.org/10.6084/m9.figshare.32251974, reference number 32251974.
