# Supplementary material for: From cellular regeneration toward functional integration: a 15-year bibliometric analysis of inner ear hair cell regeneration (2011–2025)
Source: Front Cell Neurosci. 2026 Aug 18;20:1888383. doi: 10.3389/fncel.2026.1888383 (PMC13529467; doi:10.3389/fncel.2026.1888383)
Supplement: Supplementary file 1 [file Supplementary_file_3.docx]

label	replace by

台湾	中国

Taiwan	China

台湾地区	中国

Taiwan	China

中国台北	中国

Chinese Taipei	China

Formosa	China

England	United Kingdom

Scotland	United Kingdom

Wales	United Kingdom

Northern Ireland	United Kingdom

UK	United Kingdom

Great Britain	United Kingdom

香港	中国

Hong Kong	China

澳门	中国

Macau	China

Macao	China

香港地区	中国

澳门地区	中国

Hong Kong SAR	China

Macau SAR	China

peoples r china	China
