# Supplementary material for: From cellular regeneration toward functional integration: a 15-year bibliometric analysis of inner ear hair cell regeneration (2011–2025)
Source: Front Cell Neurosci. 2026 Aug 18;20:1888383. doi: 10.3389/fncel.2026.1888383 (PMC13529467; doi:10.3389/fncel.2026.1888383)
Supplement: Supplementary file 2 [file Supplementary_file_4.docx]

Label	Replace by

math1	atoh1

Math1	atoh1

atoh1 expression	atoh1

math1 gene-transfer	atoh1

hair-cells	hair cell

hair cells	hair cell

hair cell regeneration	hair cell

auditory hair-cells	hair cell

sensory hair-cells	hair cell

inner hair-cells	hair cell

inner hair cell	hair cell

outer hair-cells	hair cell

cochlear hair cells	hair cell

auditory hair cells	hair cell

supporting cells	supporting cell

cochlear supporting cells	supporting cell

hearing-loss	hearing loss

sensorineural hearing loss	hearing loss

sensorineural hearing-loss	hearing loss

noise-induced hearing loss	hearing loss

induced hearing-loss	hearing loss

age-related hearing loss	hearing loss

inner-ear	inner ear

mammalian inner-ear	inner ear

mouse inner-ear	inner ear

inner-ear development	inner ear

mammalian inner ear	inner ear

mouse inner ear	inner ear

stem-cells	stem cell

stem cells	stem cell

pluripotent stem-cells	stem cell

embryonic stem-cells	stem cell

mesenchymal stem-cells	stem cell

neural progenitor cells	stem cell

progenitor cells	stem cell

progenitors	stem cell

gene-therapy	gene therapy

gene-transfer	gene therapy

gene-expression	gene expression

ribbon synapses	ribbon synapse

guinea-pigs	guinea pig

guinea-pig	guinea pig

mature guinea-pigs	guinea pig

guinea-pig cochlea	guinea pig

in-vivo	in vivo

in-vitro	in vitro

lateral-line	lateral line

mice	mouse

mouse model	mouse

animals	animal

humans	human

tooferlin	otoferlin

notch signaling pathway	notch

notch inhibition	notch

beta-catenin	wnt

cisplatin-induced ototoxicity	ototoxicity

induced ototoxicity	ototoxicity

noise exposure	noise

acoustic trauma	noise

spiral ganglion neurons	spiral ganglion

spiral ganglion neuron	spiral ganglion

auditory neurons	spiral ganglion

auditory-nerve	spiral ganglion

corti	organ of corti

sensory epithelia	sensory epithelium

ear	sensory epithelium

mammalian cochlea	cochlea

cochlear	cochlea

mouse cochlea	cochlea

zebrafish lateral-line	zebrafish

danio-rerio	zebrafish

direct transdifferentiation	transdifferentiation

direct conversion	transdifferentiation

death	cell death

apoptosis	cell death

damage	injury

ablation	injury

laser-ablation	injury

drug-delivery	drug delivery

adenoassociated virus	aav

vestibular function	vestibular

balance	vestibular

neurotrophins	neurotrophic factor

transcription factors	transcription factor

proteins	protein

signaling pathway	pathway

p27(kip1)	p27kip1

p27Kip1	p27kip1

rescues hearing	rescue

protects	protection

regenerative	regeneration

postnatal-development	development

mouse utricle	utricle

neuromasts	neuromast

mutations	mutation

transgene expression	expression
