# Supplementary material for: From cellular regeneration toward functional integration: a 15-year bibliometric analysis of inner ear hair cell regeneration (2011–2025)
Source: Front Cell Neurosci. 2026 Aug 18;20:1888383. doi: 10.3389/fncel.2026.1888383 (PMC13529467; doi:10.3389/fncel.2026.1888383)
Supplement: Supplementary file 3 [file Supplementary_file_6.docx]

TY - JOUR

AB - The synapse between inner hair cells (IHCs) and type I spiral ganglion neurons (SGNs) has been identified as a sensitive structure to noise-induced damage in the mammalian cochlea. Since this synapse provides the major information pathway from the cochlea to the auditory brain, it is important to maintain its integrity. Neurotrophin-3 (NT-3) has been known to play an important role in the development and the functional maintenance of this synapse. Application of exogenous NT-3, or overexpression of this gene in a transgenic animal model, have shown the value to protect this synapse from noise-induced damage. In the present study, NT-3 overexpression was induced by cochlear gene transfection before noise exposure via the use of an adeno-associated viral (AAV) vector. We found that such an overexpression provided a significant synaptic protection against a noise exposure that caused massive damage to the synapses, likely due to it promoting the repair of the synapse after the initial damage.

AD - Otolaryngology Research Institute, 6th Affiliated Hospital, Shanghai Jiao Tong University, Shanghai, China.

Otolaryngology Research Institute, 6th Affiliated Hospital, Shanghai Jiao Tong University, Shanghai, China. yinshankai@china.com.

Otolaryngology Research Institute, 6th Affiliated Hospital, Shanghai Jiao Tong University, Shanghai, China. Jian.Wang@dal.ca.

School of Human Communication Disorders, Dalhousie University, Halifax, NS, Canada. Jian.Wang@dal.ca.

AN - 29535374

AU - Chen, H.

AU - Xing, Y.

AU - Xia, L.

AU - Chen, Z.

AU - Yin, S.

AU - Wang, J.

C1 - PubMed

C2 - PMC6062503

DA - Jul

DO - 10.1038/s41434-018-0012-0

DP - NLM

ET - 2018/03/15

IS - 4

KW - Animals

Cochlea/*metabolism

Dependovirus/genetics/metabolism

Guinea Pigs

Hair Cells, Auditory/metabolism

Hair Cells, Auditory, Inner/metabolism

Hearing Loss, Noise-Induced/genetics/metabolism/*prevention & control

Male

Neurons/metabolism

Neurotrophin 3/administration & dosage/*biosynthesis/genetics

Noise/adverse effects

Synapses/metabolism

Transfection

LA - eng

N1 - 1476-5462

Chen, Hengchao

Orcid: 0000-0001-8881-8744

Xing, Yazhi

Xia, Li

Chen, Zhengnong

Yin, Shankai

Wang, Jian

Orcid: 0000-0003-3633-6799

MOP-136835/CIHR/Canada

Journal Article

Research Support, Non-U.S. Gov't

England

Gene Ther. 2018 Jul;25(4):251-259. doi: 10.1038/s41434-018-0012-0. Epub 2018 Mar 13.

PY - 2018

SN - 0969-7128 (Print)

0969-7128

SP - 251-259

ST - AAV-mediated NT-3 overexpression protects cochleae against noise-induced synaptopathy

T2 - Gene Ther

TI - AAV-mediated NT-3 overexpression protects cochleae against noise-induced synaptopathy

VL - 25

ID - 39629

ER -

TY - JOUR

AB - Inner ear hair cell (HC) damage is irreversible in mammals, but it has been shown that supporting cells (SCs) have the potential to differentiate into HCs. Serpine2, a serine protease inhibitor, encodes protease nexin 1, and this has been suggested to be a factor that promotes HC regeneration. In this study, we overexpressed Serpine2 in inner ear SCs cultured in two- and three-dimensional systems using the adeno-associated virus-inner ear (AAV-ie) vector, which promoted organoid expansion and HC differentiation. Overexpression of Serpine2 in the mouse cochlea through the round window membrane (RWM) injection promoted SC proliferation and HC regeneration, and the regenerated HCs were found to be derived from Lgr5+ SCs. Regenerated HCs have electrophysiological properties that are similar to those of native HCs. Notably, Serpine2 overexpression promoted HC survival and restored hearing of neomycin-damaged mice. In conclusion, our findings indicate that Serpine2 overexpression promotes HC regeneration and suggests that the utilization of inner ear progenitor cells in combination with AAVs might be a promising therapeutic target for hearing restoration. © 2024 The Authors

AD - State Key Laboratory of Digital Medical Engineering, Department of Otolaryngology Head and Neck Surgery, Zhongda Hospital, School of Life Sciences and Technology, School of Medicine, Advanced Institute for Life and Health, Jiangsu Province High-Tech Key Laboratory for Bio-Medical Research, Southeast University, Nanjing, 210096, China

Co-Innovation Center of Neuroregeneration, Nantong University, Nantong, 226001, China

Department of Otolaryngology-Head and Neck Surgery, the Affiliated Drum Tower Hospital of Nanjing University Medical School, Jiangsu Provincial Key Medical Discipline (Laboratory), Nanjing, 210008, China

Department of Neurology, Aerospace Center Hospital, School of Life Science, Beijing Institute of Technology, Beijing, 100081, China

Department of Otolaryngology, Head and Neck Surgery, the First Affiliated Hospital of Anhui Medical University, Hefei, 230022, China

Advanced Technology Research Institute, Beijing Institute of Technology, Jinan, 250300, China

Department of Otolaryngology Head and Neck Surgery, Sichuan Provincial People's Hospital, School of Medicine, University of Electronic Science and Technology of China, Chengdu, 610072, China

Southeast University Shenzhen Research Institute, Shenzhen, 518063, China

AU - Sun, Q.

AU - Tan, F.

AU - Wang, X.

AU - Gu, X.

AU - Chen, X.

AU - Lu, Y.

AU - Li, N.

AU - Qian, X.

AU - Zhou, Y.

AU - Zhang, Z.

AU - Wang, M.

AU - Zhang, L.

AU - Tong, B.

AU - Qi, J.

AU - Chai, R.

C1 - Scopus

C7 - 102396

DB - Scopus

DO - 10.1016/j.omtn.2024.102396

IS - 4

J2 - Mol. Ther. Nucl. Acids

KW - AAV-ie

cochlea

hair cell regeneration

inner ear stem cell

MT: Delivery Strategies

Serpine2

adeno associated virus vector

complementary DNA

neomycin

protease nexin I

RNA

serine proteinase inhibitor

tamoxifen

tissue adhesive

transcriptome

unclassified drug

Adeno associated virus

animal cell

animal tissue

Article

cell differentiation

cell maturation

cell proliferation

cell regeneration

cell survival

cochlea fenestra

controlled study

differential expression analysis

embryo

evoked brain stem auditory response

gene therapy

genome packaging

hair cell (inner ear)

hearing impairment

HEK293T cell line

human

human cell

immunofluorescence

in vitro study

injection

inner ear

Lgr5+ stem cell line

male

mouse

newborn

nonhuman

organoid

real time polymerase chain reaction

RNA sequencing

stem cell

sustentacular cell

three dimensional cell culture

two dimensional cell culture

viral gene therapy

Wnt signaling

LA - English

M3 - Article

N1 - Export Date: 21 May 2026; Cited By: 5; Correspondence Address: L. Zhang; State Key Laboratory of Digital Medical Engineering, Department of Otolaryngology Head and Neck Surgery, Zhongda Hospital, School of Life Sciences and Technology, School of Medicine, Advanced Institute for Life and Health, Jiangsu Province High-Tech Key Laboratory for Bio-Medical Research, Southeast University, Nanjing, 210096, China; email: liyanzhang@seu.edu.cn; B. Tong; Department of Otolaryngology, Head and Neck Surgery, the First Affiliated Hospital of Anhui Medical University, Hefei, 230022, China; email: tongbusheng@ahmu.edu.cn; J. Qi; Department of Neurology, Aerospace Center Hospital, School of Life Science, Beijing Institute of Technology, Beijing, 100081, China; email: qijieyu@bit.edu.cn; R. Chai; State Key Laboratory of Digital Medical Engineering, Department of Otolaryngology Head and Neck Surgery, Zhongda Hospital, School of Life Sciences and Technology, School of Medicine, Advanced Institute for Life and Health, Jiangsu Province High-Tech Key Laboratory for Bio-Medical Research, Southeast University, Nanjing, 210096, China; email: renjiec@seu.edu.cn

PY - 2024

SN - 21622531 (ISSN)

ST - AAV-regulated Serpine2 overexpression promotes hair cell regeneration

T2 - Molecular Therapy Nucleic Acids

TI - AAV-regulated Serpine2 overexpression promotes hair cell regeneration

UR - https://www.scopus.com/pages/publications/85210365949?origin=resultslist

VL - 35

ID - 39860

ER -

TY - JOUR

AB - Sensorineural hearing deficiency caused by hair cell damage represents a prevalent sensory deficit disorder. In mammals, age-related reduction in plasticity of inner ear supporting cells (recognized as hair cell precursors) compromises their trans-differentiation capacity, resulting in impaired spontaneous hair cell regeneration post-injury. Therapeutic reprogramming of supporting cells to functionally replace damaged hair cells has emerged as a promising strategy for sensorineural hearing loss treatment. In this study, we demonstrate that the secretory protein Sparcl1 enhances supporting cell reprogramming and hair cell regeneration in both in vitro and in vivo models. Through the adeno-associated virus (AAV)-mediated overexpression system, we successfully achieved in vivo expansion of inner ear organoids accompanied by hair cell differentiation. RNA-seq analysis revealed that Sparcl1 overexpression stimulates supporting cell proliferation via follistatin (Fst) activation and extracellular matrix (ECM) remodeling. Notably, both AAV-ie-Sparcl1 delivery and recombinant Sparcl1 protein administration effectively induced supporting cell differentiation into hair cells in vivo. Collectively, our findings establish Sparcl1 as a potent positive regulator of hair cell regeneration and elucidate mechanisms by which secretory proteins regulate supporting cell plasticity. © 2025 The American Society of Gene and Cell Therapy

AD - Department of Otolaryngology-Head and Neck Surgery, Zhongda Hospital, State Key Laboratory of Digital Medical Engineering, Jiangsu Provincial Key Laboratory of Critical Care Medicine, School of Life Sciences and Technology, School of Medicine, Advanced Institute for Life and Health, Southeast University, Nanjing, 210096, China

Co-Innovation Center of Neuroregeneration, Nantong University, Nantong, 226001, China

Department of Otorhinolaryngology-Head and Neck Surgery, Zhongda Hospital, Southeast University, Nanjing, 210009, China

Department of Neurology, Aerospace Center Hospital, School of Life Science, Beijing Institute of Technology, Beijing, 100081, China

Department of Otolaryngology-Head and Neck Surgery, the Affiliated Drum Tower Hospital of Nanjing University Medical School, Jiangsu Provincial Key Medical Discipline (Laboratory), Nanjing, 210008, China

Department of Otology, The First Affiliated Hospital of Zhengzhou University, Henan, Zhengzhou, 450000, China

State Key Laboratory of Hearing and Balance Science, Beijing Institute of Technology, Beijing, 100081, China

School of Medical Engineering, Affiliated Zhuhai People's Hospital, Beijing Institute of Technology, Zhuhai, 519088, China

Advanced Technology Research Institute, Beijing Institute of Technology, Jinan, 250300, China

Department of Otolaryngology-Head and Neck Surgery, Sichuan Provincial People's Hospital, School of Medicine, University of Electronic Science and Technology of China, Chengdu, 610072, China

Southeast University Shenzhen Research Institute, Shenzhen, 518063, China

AU - Li, N.

AU - Tan, F.

AU - Zhang, L.

AU - Ding, X.

AU - Sun, Q.

AU - Wang, M.

AU - Zhang, Z.

AU - Lu, Y.

AU - Zhou, Y.

AU - Qian, X.

AU - Ye, F.

AU - Qi, J.

AU - Chai, R.

C1 - Scopus

C2 - 40181541

DB - Scopus

DO - 10.1016/j.ymthe.2025.03.054

IS - 7

J2 - Mol. Ther.

KW - AAV

hair cell regeneration

secretory protein

Sparcl1

supporting cell reprogramming

Animals

Calcium-Binding Proteins

Cell Differentiation

Cell Plasticity

Cell Proliferation

Dependovirus

Disease Models, Animal

Extracellular Matrix Proteins

Genetic Vectors

Hair Cells, Auditory

Humans

Mice

Regeneration

follistatin

secretory protein Sparcl1

tamoxifen

unclassified drug

calcium binding protein

scleroprotein

Adeno associated virus

animal cell

animal experiment

animal model

Article

cell culture

cell regeneration

controlled study

dynamics

extracellular matrix remodeling

Gapdh gene

gene

HEK293T cell line

human

human cell

immunofluorescence

in vitro study

in vivo study

inner ear

inner ear organoid

mouse

nonhuman

organoid

plasmid

polymerase chain reaction

real time polymerase chain reaction

RNA sequencing

animal

cochlear hair cell

cytology

Dependoparvovirus

disease model

gene vector

genetics

metabolism

LA - English

M3 - Article

N1 - Export Date: 21 May 2026; Cited By: 3; Correspondence Address: F. Ye; Department of Otology, The First Affiliated Hospital of Zhengzhou University, Zhengzhou, Henan, 450000, China; email: yefanglei000@sina.com; J. Qi; Department of Neurology, Aerospace Center Hospital, School of Life Science, Beijing Institute of Technology, Beijing, 100081, China; email: qijieyu@bit.edu.cn; R. Chai; Department of Otolaryngology-Head and Neck Surgery, Zhongda Hospital, State Key Laboratory of Digital Medical Engineering, Jiangsu Provincial Key Laboratory of Critical Care Medicine, School of Life Sciences and Technology, School of Medicine, Advanced Institute for Life and Health, Southeast University, Nanjing, 210096, China; email: renjiec@seu.edu.cn; CODEN: MTOHC

PY - 2025

SN - 15250016 (ISSN)

SP - 3022-3035

ST - AAV-Sparcl1 promotes hair cell regeneration by increasing supporting cell plasticity

T2 - Molecular Therapy

TI - AAV-Sparcl1 promotes hair cell regeneration by increasing supporting cell plasticity

UR - https://www.scopus.com/pages/publications/105002667946?origin=resultslist

VL - 33

ID - 39851

ER -

TY - JOUR

AB - OBJECTIVES: To examine the inner ear functions of mice that lack the WD domain (δWD) of autophagy protein ATG16L1. DESIGN: Mice were assessed with vestibular evoked potentials (VsEPs), auditory brainstem responses (ABRs) and four δWD mice were observed with electron microscopy to assess otolith otoconia loss, and cochlear hair cell loss. STUDY SAMPLE: Seven δWD mice and four control mice. RESULTS: Compared to control mice, VsEP thresholds were elevated, latencies increased and amplitudes decreased in the δWD mice. ABR thresholds were elevated for the 32 kHz tonebursts (but not 8 or 16 kHz) in the δWD mice compared to controls. Electron microscopy in four δWD mice revealed a complete absence of otoconia in three of four otolith organs, with the remaining mouse (perhaps a failed mutant mouse, due to incomplete penetrance of this phenotype) showing a normal complement of otoconia. In the cochlea, abnormal hair cells were observed, typically showing modest hair cell loss or damage of hair cells or stereocilia. CONCLUSIONS: Three confirmed δWD mice showed missing otoconia, and changes in VsEPs and ABRs suggest that non-canonical autophagy pathways involved in endomembrane repair and removal are important for maintaining vestibular (and perhaps auditory) function in the inner ear.

AD - Department of Rehabilitation Science, University at Buffalo, Buffalo, NY, USA.

Department of Special Education and Communication Disorders, University of Nebraska-Lincoln, Lincoln, NE, USA.

Vestibular Neurogenics Laboratory, Boys Town National Research Hospital, Omaha, NE, USA.

Quadram Institute of Bioscience & Norwich Medical School, University of East Anglia, Norwich, UK.

AN - 40773284

AU - Burkard, R.

AU - Jones, S.

AU - Jones, T.

AU - Lundberg, Y. Y.

AU - Wileman, T.

C1 - PubMed

DA - Aug 7

DO - 10.1080/14992027.2025.2539824

DP - NLM

ET - 2025/08/07

KW - Atg16l1

Non-canonical autophagy

auditory brainstem response

cochlear hair cells

otoconia

vestibular sensory evoked potential

LA - eng

N1 - 1708-8186

Burkard, Robert

Jones, Sherri

Jones, Timothy

Lundberg, Yunxia Yesha

Wileman, Thomas

Journal Article

England

Int J Audiol. 2025 Aug 7:1-13. doi: 10.1080/14992027.2025.2539824.

PY - 2025

SN - 1499-2027

SP - 1-13

ST - An absent WD domain in the autophagy protein ATG16L1 leads to auditory and vestibular dysfunction and otoconial deficits in mice

T2 - Int J Audiol

TI - An absent WD domain in the autophagy protein ATG16L1 leads to auditory and vestibular dysfunction and otoconial deficits in mice

ID - 39261

ER -

TY - JOUR

AB - BACKGROUND: The posterior lateral line in zebrafish develops from a migrating primordium that deposits clusters of cells that differentiate into neuromasts at regular intervals along the trunk. The deposition of these neuromasts is known to be coordinated by Wnt and FGF signals that control the proliferation, migration, and organization of the primordium. However, little is known about the control of proliferation in the neuromasts following their deposition. RESULTS: We show that pharmacological activation of the Wnt/β-catenin signaling pathway with 1-azakenpaullone upregulates proliferation in neuromasts post-deposition. This results in increased size of the neuromasts and overproduction of sensory hair cells. We also show that activation of Wnt signaling returns already quiescent supporting cells to a proliferative state in mature neuromasts. Additionally, activation of Wnt signaling increases the number of supporting cells that return to the cell cycle in response to hair cell damage and the number of regenerated hair cells. Finally, we show that inhibition of Wnt signaling by overexpression of dkk1b suppresses proliferation during both differentiation and regeneration. CONCLUSIONS: These data suggest that Wnt/β-catenin signaling is both necessary and sufficient for the control of proliferation of lateral line progenitors during development, ongoing growth of the neuromasts, and hair cell regeneration.

AD - Neuroscience Program, Colgate University, Hamilton, New York, USA.

AN - 23606225

AU - Head, J. R.

AU - Gacioch, L.

AU - Pennisi, M.

AU - Meyers, J. R.

C1 - PubMed

DA - Jul

DO - 10.1002/dvdy.23973

DP - NLM

ET - 2013/04/23

IS - 7

KW - Animals

Benzazepines/pharmacology

Cell Differentiation/genetics/physiology

Cell Proliferation

Hair Cells, Auditory/cytology/drug effects

Indoles/pharmacology

Wnt Signaling Pathway/genetics/*physiology

Zebrafish/*embryology/*metabolism

Zebrafish Proteins/genetics/*metabolism

LA - eng

N1 - 1097-0177

Head, Jeffery R

Gacioch, Leah

Pennisi, Matthew

Meyers, Jason R

Journal Article

Research Support, U.S. Gov't, Non-P.H.S.

United States

Dev Dyn. 2013 Jul;242(7):832-46. doi: 10.1002/dvdy.23973. Epub 2013 Jun 5.

PY - 2013

SN - 1058-8388

SP - 832-46

ST - Activation of canonical Wnt/β-catenin signaling stimulates proliferation in neuromasts in the zebrafish posterior lateral line

T2 - Dev Dyn

TI - Activation of canonical Wnt/β-catenin signaling stimulates proliferation in neuromasts in the zebrafish posterior lateral line

VL - 242

ID - 39223

ER -

TY - JOUR

AB - A loss of sensory hair cells or spiral ganglion neurons from the inner ear causes deafness, affecting millions of people. Currently, there is no effective therapy to repair the inner ear sensory structures in humans. Cochlear implantation can restore input, but only if auditory neurons remain intact. Efforts to develop stem cell-based treatments for deafness have demonstrated progress, most notably utilizing embryonic-derived cells. In an effort to bypass limitations of embryonic or induced pluripotent stem cells that may impede the translation to clinical applications, we sought to utilize an alternative cell source. Here, we show that adult human mesenchymal-like stem cells (MSCs) obtained from nasal tissue can repair spiral ganglion loss in experimentally lesioned cochlear cultures from neonatal rats. Stem cells engraft into gentamicin-lesioned organotypic cultures and orchestrate the restoration of the spiral ganglion neuronal population, involving both direct neuronal differentiation and secondary effects on endogenous cells. As a physiologic assay, nasal MSC-derived cells engrafted into lesioned spiral ganglia demonstrate responses to infrared laser stimulus that are consistent with those typical of excitable cells. The addition of a pharmacologic activator of the canonical Wnt/β-catenin pathway concurrent with stem cell treatment promoted robust neuronal differentiation. The availability of an effective adult autologous cell source for inner ear tissue repair should contribute to efforts to translate cell-based strategies to the clinic.

AD - 1 Department of Otolaryngology, University of Miami Miller School of Medicine , Miami, Florida.

AN - 24172073

AU - Bas, E.

AU - Van De Water, T. R.

AU - Lumbreras, V.

AU - Rajguru, S.

AU - Goss, G.

AU - Hare, J. M.

AU - Goldstein, B. J.

C1 - PubMed

C2 - PMC3928683

DA - Mar 1

DO - 10.1089/scd.2013.0274

DP - NLM

ET - 2013/11/01

IS - 5

KW - Adult

Animals

Cochlea/growth & development/injuries/pathology

Ear, Inner/growth & development/pathology

Humans

*Mesenchymal Stem Cell Transplantation

Mesenchymal Stem Cells/*cytology

*Nerve Regeneration

Neurons/pathology

Rats

Spiral Ganglion/growth & development/injuries/pathology

Wnt Signaling Pathway/genetics

LA - eng

N1 - 1557-8534

Bas, Esperanza

Van De Water, Thomas R

Lumbreras, Vicente

Rajguru, Suhrud

Goss, Garrett

Hare, Joshua M

Goldstein, Bradley J

NIH/NIDCD R01DC011481-02A2/PHS HHS/United States

Journal Article

Research Support, N.I.H., Extramural

Research Support, Non-U.S. Gov't

United States

Stem Cells Dev. 2014 Mar 1;23(5):502-14. doi: 10.1089/scd.2013.0274. Epub 2013 Dec 4.

PY - 2014

SN - 1547-3287 (Print)

1547-3287

SP - 502-14

ST - Adult human nasal mesenchymal-like stem cells restore cochlear spiral ganglion neurons after experimental lesion

T2 - Stem Cells Dev

TI - Adult human nasal mesenchymal-like stem cells restore cochlear spiral ganglion neurons after experimental lesion

VL - 23

ID - 39093

ER -

TY - JOUR

AB - The phospholipid- and Ca(2+)-binding protein annexin A5 (ANXA5) is the most abundant membrane-associated protein of ~P23 mouse vestibular hair bundles, the inner ear's sensory organelle. Using quantitative mass spectrometry, we estimated that ANXA5 accounts for ~15,000 copies per stereocilium, or ~2% of the total protein there. Although seven other annexin genes are expressed in mouse utricles, mass spectrometry showed that none were present at levels near ANXA5 in bundles and none were upregulated in stereocilia of Anxa5(-/-) mice. Annexins have been proposed to mediate Ca(2+)-dependent repair of membrane lesions, which could be part of the repair mechanism in hair cells after noise damage. Nevertheless, mature Anxa5(-/-) mice not only have normal hearing and balance function, but following noise exposure, they are identical to wild-type mice in their temporary or permanent changes in hearing sensitivity. We suggest that despite the unusually high levels of ANXA5 in bundles, it does not play a role in the bundle's key function, mechanotransduction, at least until after two months of age in the cochlea and six months of age in the vestibular system. These results reinforce the lack of correlation between abundance of a protein in a specific compartment or cellular structure and its functional significance.

AD - Oregon Hearing Research Center, Oregon Health &Science University, Portland, OR 97239, USA.

Vollum Institute, Oregon Health &Science University, Portland, OR 97239, USA.

Department of Microbiology &Molecular Genetics, Department of Pediatrics &Human Development, Michigan State University, East Lansing, MI 48824, USA.

Department of Special Education &Communication Disorders, University of Nebraska-Lincoln, Lincoln, NE 68583, USA.

School of Public Health, Oregon Health &Science University, Portland, OR 97239, USA.

AN - 27251877

AU - Krey, J. F.

AU - Drummond, M.

AU - Foster, S.

AU - Porsov, E.

AU - Vijayakumar, S.

AU - Choi, D.

AU - Friderici, K.

AU - Jones, S. M.

AU - Nuttall, A. L.

AU - Barr-Gillespie, P. G.

C1 - PubMed

C2 - PMC4890179

DA - Jun 2

DO - 10.1038/srep27221

DP - NLM

ET - 2016/06/03

KW - Animals

Annexin A5/*genetics/metabolism

Calcium/metabolism

Evoked Potentials, Auditory

Female

Gene Knockout Techniques

Hair Cells, Auditory/*physiology

Male

Mass Spectrometry

Mechanotransduction, Cellular

Mice

Stereocilia/*metabolism

Vestibule, Labyrinth/*growth & development/physiology

LA - eng

N1 - 2045-2322

Krey, Jocelyn F

Drummond, Meghan

Foster, Sarah

Porsov, Edward

Vijayakumar, Sarath

Choi, Dongseok

Friderici, Karen

Jones, Sherri M

Nuttall, Alfred L

Barr-Gillespie, Peter G

P30 EY010572/EY/NEI NIH HHS/United States

R01 DC000141/DC/NIDCD NIH HHS/United States

S10 RR025512/RR/NCRR NIH HHS/United States

F32 DC012455/DC/NIDCD NIH HHS/United States

S10 OD012246/OD/NIH HHS/United States

P30 DC005983/DC/NIDCD NIH HHS/United States

R01 DC002368/DC/NIDCD NIH HHS/United States

R01 DC011034/DC/NIDCD NIH HHS/United States

R01 DC004568/DC/NIDCD NIH HHS/United States

P30 CA069533/CA/NCI NIH HHS/United States

Journal Article

Research Support, N.I.H., Extramural

Research Support, Non-U.S. Gov't

England

Sci Rep. 2016 Jun 2;6:27221. doi: 10.1038/srep27221.

PY - 2016

SN - 2045-2322

SP - 27221

ST - Annexin A5 is the Most Abundant Membrane-Associated Protein in Stereocilia but is Dispensable for Hair-Bundle Development and Function

T2 - Sci Rep

TI - Annexin A5 is the Most Abundant Membrane-Associated Protein in Stereocilia but is Dispensable for Hair-Bundle Development and Function

VL - 6

ID - 39631

ER -

TY - JOUR

AB - Cochlear sensory hair cells (HCs) are crucial for hearing as mechanoreceptors of the auditory systems. Clarification of transcriptional regulation for the cochlear sensory HC development is crucial for the improvement of cell replacement therapies for hearing loss. Transcription factor Atoh1 is the key player during HC development and regeneration. In this review, we will focus on Atoh1 and its related signaling pathways (Notch, fibroblast growth factor, and Wnt/β-catenin signaling) involved in the development of cochlear sensory HCs. We will also discuss the potential applicability of these signals for the induction of HC regeneration.

AD - Department of Otolaryngology-Head and Neck Surgery, Chonnam National University Hospital, Chonnam National University Medical School, Gwangju, Korea.; Research Institute of Medical Sciences, Chonnam National University, Gwangju, Korea.

Research Institute of Medical Sciences, Chonnam National University, Gwangju, Korea.; Department of Physiology, Chonnam National University Medical School, Gwangju, Korea.

AN - 28184337

AU - Lee, S.

AU - Jeong, H. S.

AU - Cho, H. H.

C1 - PubMed

C2 - PMC5299128

DA - Jan

DO - 10.4068/cmj.2017.53.1.37

DP - NLM

ET - 2017/02/12

IS - 1

KW - Hair Cells, Auditory

Hearing Loss

Regeneration

Transcription Factors

LA - eng

N1 - 2233-7393

Lee, Sungsu

Jeong, Han-Seong

Cho, Hyong-Ho

Journal Article

Korea (South)

Chonnam Med J. 2017 Jan;53(1):37-46. doi: 10.4068/cmj.2017.53.1.37. Epub 2017 Jan 25.

PY - 2017

SN - 2233-7385 (Print)

2233-7393

SP - 37-46

ST - Atoh1 as a Coordinator of Sensory Hair Cell Development and Regeneration in the Cochlea

T2 - Chonnam Med J

TI - Atoh1 as a Coordinator of Sensory Hair Cell Development and Regeneration in the Cochlea

VL - 53

ID - 39059

ER -

TY - JOUR

AB - Atonal homolog 1 (Atoh1) is a basic helix-loop-helix (bHLH) transcription factor that is essential for the genesis, survival, and maturation of a variety of neuronal and non-neuronal cell populations, including those involved in proprioception, interoception, balance, respiration, and hearing. Such diverse functions require fine regulation at the transcriptional and protein levels. Here, we show that serine 193 (S193) is phosphorylated in Atoh1's bHLH domain in vivo Knock-in mice of both sexes bearing a GFP-tagged phospho-dead S193A allele on a null background (Atoh1(S193A/lacZ)) exhibit mild cerebellar foliation defects, motor impairments, partial pontine nucleus migration defects, cochlear hair cell degeneration, and profound hearing loss. We also found that Atoh1 heterozygous mice of both sexes (Atoh1(lacZ/+)) have adult-onset deafness. These data indicate that different cell types have different degrees of vulnerability to loss of Atoh1 function and that hypomorphic Atoh1 alleles should be considered in human hearing loss.SIGNIFICANCE STATEMENT The discovery that Atonal homolog 1 (Atoh1) governs the development of the sensory hair cells in the inner ear led to therapeutic efforts to restore these cells in cases of human deafness. Because prior studies of Atoh1-heterozygous mice did not examine or report on hearing loss in mature animals, it has not been clinical practice to sequence ATOH1 in people with deafness. Here, in seeking to understand how phosphorylation of Atoh1 modulates its effects in vivo, we discovered that inner ear hair cells are much more vulnerable to loss of Atoh1 function than other Atoh1-positive cell types and that heterozygous mice actually develop hearing loss late in life. This opens up the possibility that missense mutations in ATOH1 could increase human vulnerability to loss of hair cells because of aging or trauma.

AD - Jan and Dan Duncan Neurological Research Institute, Texas Children's Hospital, Houston, Texas 77030.

Program in Integrative Molecular and Biomedical Sciences.

Department of Molecular and Human Genetics.

Program in Developmental Biology.

Huffington Center on Aging.

Department of Molecular and Cellular Biology.

Bobby R Alford Department of Otolaryngology, Head and Neck Surgery, and.

Department of Neuroscience, and.

Jan and Dan Duncan Neurological Research Institute, Texas Children's Hospital, Houston, Texas 77030, hzoghbi@bcm.edu klisch@bcm.edu.

Howard Hughes Medical Institute, Baylor College of Medicine, Houston, Texas 77030.

AN - 28729444

AU - Xie, W. R.

AU - Jen, H. I.

AU - Seymour, M. L.

AU - Yeh, S. Y.

AU - Pereira, F. A.

AU - Groves, A. K.

AU - Klisch, T. J.

AU - Zoghbi, H. Y.

C1 - PubMed

C2 - PMC6596675

DA - Sep 6

DO - 10.1523/jneurosci.0295-17.2017

DP - NLM

ET - 2017/07/22

IS - 36

KW - Aging/*genetics/pathology

*Alleles

Animals

Basic Helix-Loop-Helix Proteins/*genetics

Female

Gene Knock-In Techniques

Genetic Predisposition to Disease/*genetics

Hair Cells, Auditory/*pathology

Hearing Loss/*genetics/pathology

Male

Mice

Mice, Inbred C57BL

Mice, Transgenic

Movement Disorders/*genetics/pathology

Mutation, Missense/genetics

Serine/genetics

Atoh1

Math1

hearing loss

inner ear

phosphorylation

ponti nucleus

LA - eng

N1 - 1529-2401

Xie, Wei Rose

Orcid: 0000-0002-6011-4435

Jen, Hsin-I

Seymour, Michelle L

Yeh, Szu-Ying

Orcid: 0000-0003-4506-5652

Pereira, Fred A

Orcid: 0000-0003-2100-0280

Groves, Andrew K

Orcid: 0000-0002-0784-7998

Klisch, Tiemo J

Orcid: 0000-0001-8182-384x

Zoghbi, Huda Y

Orcid: 0000-0002-0700-3349

S10 OD016167/OD/NIH HHS/United States

U54 HD083092/HD/NICHD NIH HHS/United States

R25 GM056929/GM/NIGMS NIH HHS/United States

R01 DC014832/DC/NIDCD NIH HHS/United States

R01 DC006185/DC/NIDCD NIH HHS/United States

Journal Article

Research Support, N.I.H., Extramural

United States

J Neurosci. 2017 Sep 6;37(36):8583-8594. doi: 10.1523/JNEUROSCI.0295-17.2017. Epub 2017 Jul 20.

PY - 2017

SN - 0270-6474 (Print)

0270-6474

SP - 8583-8594

ST - An Atoh1-S193A Phospho-Mutant Allele Causes Hearing Deficits and Motor Impairment

T2 - J Neurosci

TI - An Atoh1-S193A Phospho-Mutant Allele Causes Hearing Deficits and Motor Impairment

VL - 37

ID - 39272

ER -

TY - JOUR

AB - Hearing in mammals relies upon the transduction of sound by hair cells (HCs) in the organ of Corti within the cochlea of the inner ear. Sensorineural hearing loss is a widespread and permanent disability due largely to a lack of HC regeneration in mammals. Recent studies suggest that targeting the retinoblastoma (Rb)/E2F pathway can elicit proliferation of auditory HCs. However, previous attempts to induce HC proliferation in this manner have resulted in abnormal cochlear morphology, HC death, and hearing loss. Here we show that cochlear HCs readily proliferate and survive following neonatal, HC-specific, conditional knock-out of p27Kip1 (p27CKO), a tumor suppressor upstream of Rb. Indeed, HC-specific p27CKO results in proliferation of these cells without the upregulation of the supporting cell or progenitor cell proteins, Prox1 or Sox2, suggesting that they remain HCs. Furthermore, p27CKO leads to a significant addition of postnatally derived HCs that express characteristic synaptic and stereociliary markers and survive to adulthood, although a portion of the newly derived inner HCs exhibit cytocauds and lack VGlut3 expression. Despite this, p27CKO mice exhibit normal hearing as measured by evoked auditory brainstem responses, which suggests that the newly generated HCs may contribute to, or at least do not greatly detract from, function. These results show that p27Kip1 actively maintains HC quiescence in postnatal mice, and suggest that inhibition of p27Kip1 in residual HCs represents a potential strategy for cell-autonomous auditory HC regeneration. © 2014 the authors.

AD - Department of Developmental Neurobiology, St. Jude Children's Research Hospital, Memphis, 38105, TN, United States

AU - Walters, B. J.

AU - Liu, Z.

AU - Crabtree, M.

AU - Coak, E.

AU - Cox, B. C.

AU - Zuo, J.

C1 - Scopus

C2 - 25411503

DB - Scopus

DO - 10.1523/JNEUROSCI.3200-14.2014

IS - 47

J2 - J. Neurosci.

KW - Cdkn1b

Cip/Kip

Cochlea

Cyclins

Regeneration

Retinoblastoma

Aging

Animals

Animals, Newborn

Cell Proliferation

Cyclin-Dependent Kinase Inhibitor p27

Gene Deletion

Hair Cells, Auditory

Hearing

Mice

Mice, Knockout

cyclin dependent kinase inhibitor 1B

Cdkn1b protein, mouse

animal cell

animal experiment

animal model

animal tissue

Article

autonomic nervous system

cell cycle

cell regeneration

confocal microscopy

controlled study

evoked brain stem auditory response

hair cell

immunohistochemistry

mouse

nonhuman

postnatal development

protein expression

signal transduction

synaptic transmission

animal

cochlear hair cell

cytology

genetics

growth, development and aging

knockout mouse

newborn

physiology

LA - English

M3 - Article

N1 - Export Date: 21 May 2026; Cited By: 40; CODEN: JNRSD

PY - 2014

SN - 02706474 (ISSN)

SP - 15751-15763

ST - Auditory hair cell-specific deletion of p27Kip1 in postnatal mice promotes cell-autonomous generation of new hair cells and normal hearing

T2 - Journal of Neuroscience

TI - Auditory hair cell-specific deletion of p27Kip1 in postnatal mice promotes cell-autonomous generation of new hair cells and normal hearing

UR - https://www.scopus.com/pages/publications/84911447118?origin=resultslist

VL - 34

ID - 39846

ER -

TY - JOUR

AB - Ribbon synapses between inner hair cells (IHCs) and type I spiral ganglion neurons (SGNs) in the inner ear are damaged by noise trauma and with aging, causing 'synaptopathy 'and hearing loss. Co-cultures of neonatal denervated organs of Corti and newly introduced SGNs have been developed to find strategies for improving IHC synapse regeneration, but evidence of the physiological normality of regenerated synapses is missing. This study utilizes IHC optogenetic stimulation and SGN recordings, showing that newly formed IHC synapses are indeed functional, exhibiting glutamatergic excitatory postsynaptic currents. When older organs of Corti were plated, synaptic activity probed by deconvolution, showed more mature release properties, closer to the highly specialized mode of IHC synaptic transmission that is crucial for coding the sound signal. This newly developed functional assessment of regenerated IHC synapses provides a powerful tool for testing approaches to improve synapse regeneration.

AD - The Center for Hearing and Balance, The Johns Hopkins School of Medicine, Baltimore, Maryland.

Department of Otolaryngology Head and Neck Surgery, The Johns Hopkins School of Medicine, Baltimore, Maryland.

Department of Neuroscience, The Johns Hopkins School of Medicine, Baltimore, Maryland.

Department of Biomedical Engineering, The Johns Hopkins School of Medicine, Baltimore, Maryland.

Department of Otolaryngology, Harvard Medical School, Boston, Massachusetts, USA.

Eaton-Peabody Laboratory, Massachusetts Eye and Ear, Boston, Massachusetts, USA.

Program in Speech and Hearing Bioscience and Technology, Harvard Medical School, Boston, Massachusetts, USA.

Harvard Stem Cell Institute, Cambridge, Massachusetts, USA.

AN - 38076928

AU - Vincent, P. F. Y.

AU - Young, E. D.

AU - Edge, A. S. B.

AU - Glowatzki, E.

C1 - PubMed

C2 - PMC10705289

DA - Dec 2

DO - 10.1101/2023.10.05.561095

DP - NLM

ET - 2023/12/11

LA - eng

N1 - 2692-8205

Vincent, Philippe F Y

Orcid: 0000-0001-7712-7198

Young, Eric D

Edge, Albert S B

Orcid: 0000-0001-8641-755x

Glowatzki, Elisabeth

Orcid: 0000-0003-3135-658x

R01 DC006476/DC/NIDCD NIH HHS/United States

R01 DC007174/DC/NIDCD NIH HHS/United States

Journal Article

Preprint

United States

bioRxiv [Preprint]. 2023 Dec 2:2023.10.05.561095. doi: 10.1101/2023.10.05.561095.

PY - 2023

SN - 2692-8205

ST - Auditory Hair Cells and Spiral Ganglion Neurons Regenerate Synapses with Refined Release Properties In Vitro

T2 - bioRxiv

TI - Auditory Hair Cells and Spiral Ganglion Neurons Regenerate Synapses with Refined Release Properties In Vitro

ID - 38917

ER -

TY - JOUR

AB - INTRODUCTION: Vestibular hair cells (HCs) are essential for maintaining balance and detecting head movements. In mammals, following vestibular damage HC regeneration derives from epithelial non-hair cells (ENHCs), which possess limited capacity for proliferation and reprogramming. OBJECTIVES: To examine the role of BMP4 in reprogramming utricular HCs following ototoxic injury in postnatal mice. METHODS: The study utilized both wild-type mice and transgenic strains on a C57BL/6J background, including Notch1(flox/flox), Pou4f3(+/DTR), ROSA26(tdTomato), Atoh1-eGFP; Sox9-CreER(T2), and Fos-CreER(T2), to investigate and lineage-trace the reprogramming of new HCs initiated by BMP4 protein. Advanced sequencing techniques, including single-cell RNA sequencing, bulk RNA sequencing, and CUT&Tag sequencing, were employed for transcriptomic and epigenomic analyses. To induce vestibular dysfunction, intraperitoneal injections of IDPN were administered to postnatal day 30 (P30) mice. Vestibular function was assessed through behavioral tests, including vestibulo-ocular reflex, off-vertical axis rotation, and gait analysis, to evaluate the functional outcomes of HC regeneration. For cellular studies, sphere or explant tissues from P2 mice, with gentamicin or small molecule cocktail in vitro, were used to evaluate proliferation and differentiation of ENHCs through EdU labelling or tdTomato lineage tracing, histological analyses, immunofluorescence staining, and Western blot analysis. RESULTS: We found that increased BMP4 expression enhances ENHC reprogramming, accompanied by elevated levels of key HC transcription factors, including Gfi1, Pou4f3, and Atoh1, via c-Fos activation. Moreover, exogenous BMP4 further sensitized ENHCs to Notch inhibition and Wnt pathway activation, thus amplifying the regenerative outcomes. Conversely, inhibition of c-Fos or BMP4 diminished these effects, demonstrating that BMP4 is essential for both Notch inhibition and Wnt activation. Notably, the use of a combination of small molecules targeting these pathways successfully restored vestibular function and promoted HC regeneration in adult mice. CONCLUSION: Our findings suggest that BMP4 and its associated signaling pathways represent promising therapeutic targets for the restoration of hearing and balance.

AD - ENT Institute and Otorhinolaryngology, Department of Affiliated Eye and ENT Hospital, Key Laboratory of Hearing Medicine of NHFPC, Shanghai Key Laboratory of Gene Editing and Cell Therapy for Rare Diseases, State Key Laboratory of Medical Neurobiology, Fudan University, Shanghai 200031, China.

ENT Institute and Otorhinolaryngology, Department of Affiliated Eye and ENT Hospital, Key Laboratory of Hearing Medicine of NHFPC, Shanghai Key Laboratory of Gene Editing and Cell Therapy for Rare Diseases, State Key Laboratory of Medical Neurobiology, Fudan University, Shanghai 200031, China. Electronic address: hwli@shmu.edu.cn.

ENT Institute and Otorhinolaryngology, Department of Affiliated Eye and ENT Hospital, Key Laboratory of Hearing Medicine of NHFPC, Shanghai Key Laboratory of Gene Editing and Cell Therapy for Rare Diseases, State Key Laboratory of Medical Neurobiology, Fudan University, Shanghai 200031, China. Electronic address: Shansun@fudan.edu.cn.

AN - 41325835

AU - You, D.

AU - Zhang, Y.

AU - Wang, K.

AU - Guo, L.

AU - Guo, J.

AU - Che, C.

AU - Li, W.

AU - Zhao, L.

AU - Li, H.

AU - Sun, S.

C1 - PubMed

DA - Nov 29

DO - 10.1016/j.jare.2025.11.065

DP - NLM

ET - 2025/12/02

KW - Adult mice

BMP4 signaling

Progenitor cells

Vestibular function recovery

c-Fos

competing financial interests or personal relationships that could have appeared

to influence the work reported in this paper.

LA - eng

N1 - 2090-1224

You, Dan

Zhang, Yunzhong

Wang, Kunkun

Guo, Luo

Guo, Jin

Che, Chenhao

Li, Wen

Zhao, Liping

Li, Huawei

Sun, Shan

Journal Article

Egypt

J Adv Res. 2025 Nov 29:S2090-1232(25)00960-9. doi: 10.1016/j.jare.2025.11.065.

PY - 2025

SN - 2090-1224

ST - BMP4 cocktail promotes utricular progenitor reprogramming and vestibular functional recovery in adult mice

T2 - J Adv Res

TI - BMP4 cocktail promotes utricular progenitor reprogramming and vestibular functional recovery in adult mice

ID - 39366

ER -

TY - JOUR

AB - Platinum-based chemotherapy drugs, including cisplatin (CDDP), are known to cause irreversible hearing loss. We recently discovered that BRCA1 facilitates the repair of CDDP-induced DNA double-strand breaks (DSBs) in cochlear hair cells (HCs) and prevents hearing loss. However, the checkpoint pathways activated in response to DSBs in HCs, and the mechanisms by which Brca1 regulates these pathways, remain unclear. In this study, we demonstrate that CDDP induces significant DSBs in outer HCs (OHCs), accompanied by phosphorylation of ATM, CHK2, and p53 at serine‑15 (S15) and S20, leading to apoptosis. In contrast, CDDP induces fewer DSBs in inner HCs (IHCs), with phosphorylation of ATM and p53 at S20, but not CHK2, promoting cell survival. Brca1 deficiency further increases phosphorylation of ATM and p53 at S15 in CDDP-treated OHCs. In response to CDDP, phosphorylation of CHK2 is not detected, but phosphorylation of p53 at S20 is enhanced in Brca1-deficient OHCs, whereas, phosphorylation of p53 at S20 is significantly increased in both Brca1-deficient and proficient IHCs. The expression patterns of phosphorylated ATM, CHK2, and p53 in response to CDDP in cultured explants and cell lines differ markedly from those observed in Brca1-deficient and proficient mice in vivo. These findings suggest that CDDP induces extensive DSBs in OHCs, activating the ATM-CHK2-p53 pathway to promote cell death, while Brca1 deficiency exacerbates CDDP-induced DSBs and activates ATM-p53 signaling independent of CHK2, accelerating OHC loss. Additionally, these results demonstrate that CDDP induces modest DSBs in IHCs with activation of the ATM-p53 pathway, independent of CHK2 and BRCA1, leading to cell survival.

AD - International Cancer Center, Marshall Laboratory of Biomedical Engineering, Department of Otolaryngology, The First Affiliated Hospital, Department of Anatomy and Histology, Shenzhen University Medical School, Shenzhen 518060, China.

Department of Pathology, Shenzhen University Medical School, Shenzhen 518060, China.

Guangdong Key Laboratory for Genome Stability & Disease Prevention and International Cancer Center, Shenzhen University Medical School, Shenzhen 518060, China.

Department of Otolaryngology, The First Affiliated Hospital of Shenzhen University, Shenzhen Second People's Hospital, Shenzhen 518035, China.

Department of Biochemistry and Molecular Biology, International Cancer Center, Shenzhen University Medical School, Shenzhen 518060, China.

Department of Otolaryngology, The First Affiliated Hospital of Shenzhen University, Shenzhen Second People's Hospital, Shenzhen 518035, China. Electronic address: szshent@email.szu.edu.cn.

International Cancer Center, Marshall Laboratory of Biomedical Engineering, Department of Otolaryngology, The First Affiliated Hospital, Department of Anatomy and Histology, Shenzhen University Medical School, Shenzhen 518060, China. Electronic address: peixinhai@szu.edu.cn.

AN - 40684720

AU - Jiang, W.

AU - Bai, F.

AU - Wang, G.

AU - Liu, X.

AU - Peng, B.

AU - Xu, X.

AU - Nie, G.

AU - Zhu, W. G.

AU - Hu, B.

AU - Pei, X. H.

C1 - PubMed

DA - Sep

DO - 10.1016/j.heares.2025.109369

DP - NLM

ET - 2025/07/21

KW - Animals

*Ataxia Telangiectasia Mutated Proteins/metabolism

*Checkpoint Kinase 2/metabolism

*Tumor Suppressor Protein p53/metabolism

*BRCA1 Protein/deficiency/genetics

Cisplatin/toxicity

Phosphorylation

Signal Transduction/drug effects

DNA Breaks, Double-Stranded

Apoptosis/drug effects

Mice, Knockout

Mice

*DNA Damage

*Hair Cells, Auditory, Outer/drug effects/pathology/enzymology

Mice, Inbred C57BL

Cell Survival

*Hair Cells, Auditory/drug effects/pathology/enzymology

Antineoplastic Agents/toxicity

Brca1

Checkpoint pathway

Cisplatin

DNA damage

Hair cells

interest exists.

LA - eng

N1 - 1878-5891

Jiang, Weitao

Bai, Feng

Wang, Guanrun

Liu, Xiong

Peng, Bin

Xu, Xingzhi

Nie, Guohui

Zhu, Wei-Guo

Hu, Bing

Pei, Xin-Hai

Journal Article

Netherlands

Hear Res. 2025 Sep;465:109369. doi: 10.1016/j.heares.2025.109369. Epub 2025 Jul 14.

PY - 2025

SN - 0378-5955

SP - 109369

ST - BRCA1 deficiency promotes DNA damage in cochlear hair cells with activation of ATM-p53 pathway independent of CHK2

T2 - Hear Res

TI - BRCA1 deficiency promotes DNA damage in cochlear hair cells with activation of ATM-p53 pathway independent of CHK2

VL - 465

ID - 38937

ER -

TY - JOUR

AB - Cochlear hair cells (HCs) sense sound waves and allow us to hear. Loss of HCs will cause irreversible sensorineural hearing loss. It is well known that DNA damage repair plays a critical role in protecting cells in many organs. However, how HCs respond to DNA damage and how defective DNA damage repair contributes to hearing loss remain elusive. In this study, we showed that cisplatin induced DNA damage in outer hair cells (OHCs) and promoted OHC loss, leading to hearing loss in mice of either sex. Cisplatin induced the expression of Brca1, a DNA damage repair factor, in OHCs. Deficiency of Brca1 induced OHC and hearing loss, and further promoted cisplatin-induced DNA damage in OHCs, accelerating OHC loss. This study provides the first in vivo evidence demonstrating that cisplatin mainly induces DNA damage in OHCs and that BRCA1 promotes repair of DNA damage in OHCs and prevents hearing loss. Our findings not only demonstrate that DNA damage-inducing agent generates DNA damage in postmitotic HCs but also suggest that DNA repair factors, like BRCA1, protect postmitotic HCs from DNA damage-induced cell death and hearing loss.

AD - International Cancer Center, Marshall Laboratory of Biomedical Engineering, Department of Otolaryngology, The First Affiliated Hospital, Department of Anatomy and Histology, Shenzhen University Medical School, Shenzhen 518060, China.

Department of Pathology, Shenzhen University Medical School, Shenzhen 518060, China.

Shenzhen Key Laboratory of Nanozymes and Translational Cancer Research, Department of Otolaryngology, Shenzhen Institute of Translational Medicine, The First Affiliated Hospital of Shenzhen University, Shenzhen Second People's Hospital, Shenzhen 518035, China.

Department of Biomedical Engineering, Southern University of Science and Technology, Shenzhen 518055, China.

Guangdong Key Laboratory for Genome Stability & Disease Prevention and International Cancer Center, Shenzhen University Medical School, Shenzhen, 518060, China.

Department of Biochemistry and Molecular Biology, International Cancer Center, Shenzhen University Medical School, Shenzhen 518060, China.

International Cancer Center, Marshall Laboratory of Biomedical Engineering, Department of Otolaryngology, The First Affiliated Hospital, Department of Anatomy and Histology, Shenzhen University Medical School, Shenzhen 518060, China peixinhai@szu.edu.cn.

AN - 39227158

AU - Jiang, W.

AU - Wang, G.

AU - Bai, F.

AU - Hu, B.

AU - Xu, Y.

AU - Xu, X.

AU - Nie, G.

AU - Zhu, W. G.

AU - Chen, F.

AU - Pei, X. H.

C1 - PubMed

C2 - PMC11484548

DA - Oct 16

DO - 10.1523/jneurosci.0132-24.2024

DP - NLM

ET - 2024/09/04

IS - 42

KW - Animals

*BRCA1 Protein/metabolism/genetics

*DNA Damage

Mice

*Cisplatin/toxicity

Female

Male

*Hearing Loss/prevention & control/genetics/metabolism

*DNA Repair/drug effects

Hair Cells, Auditory/drug effects/metabolism

Hair Cells, Auditory, Outer/drug effects/metabolism

Mice, Inbred C57BL

Mice, Knockout

Brca1

DNA damage

cisplatin

hair cells

hearing loss

LA - eng

N1 - 1529-2401

Jiang, Weitao

Orcid: 0009-0006-5726-2847

Wang, Guanrun

Bai, Feng

Hu, Bing

Xu, Yang

Orcid: 0009-0005-2210-2424

Xu, Xingzhi

Nie, Guohui

Zhu, Wei-Guo

Chen, Fangyi

Pei, Xin-Hai

Orcid: 0000-0001-6441-1691

Journal Article

Research Support, Non-U.S. Gov't

United States

J Neurosci. 2024 Oct 16;44(42):e0132242024. doi: 10.1523/JNEUROSCI.0132-24.2024.

PY - 2024

SN - 0270-6474 (Print)

0270-6474

ST - BRCA1 Promotes Repair of DNA Damage in Cochlear Hair Cells and Prevents Hearing Loss

T2 - J Neurosci

TI - BRCA1 Promotes Repair of DNA Damage in Cochlear Hair Cells and Prevents Hearing Loss

VL - 44

ID - 38890

ER -

TY - JOUR

AB - Millions of people worldwide suffer from hearing and balance disorders caused by loss of the sensory hair cells that convert sound vibrations and head movements into electrical signals that are conveyed to the brain. In mammals, the great majority of hair cells are produced during embryogenesis. Hair cells that are lost after birth are virtually irreplaceable, leading to permanent disability. Other vertebrates, such as fish and amphibians, produce hair cells throughout life. However, hair cell replacement after damage to the mature inner ear was either not investigated or assumed to be impossible until studies in the late 1980s proved this to be false. Adult birds were shown to regenerate lost hair cells in the auditory sensory epithelium after noise- and ototoxic drug-induced damage. Since then, the field of hair cell regeneration has continued to investigate the capacity of the auditory and vestibular epithelia in vertebrates (fishes, birds, reptiles, and mammals) to regenerate hair cells and to recover function, the molecular mechanisms governing these regenerative capabilities, and the prospect of designing biologically-based treatments for hearing loss and balance disorders. Here, we review the major findings of the field during the past 25 years and speculate how future inner ear repair may one day be achieved. © 2013 Elsevier B.V.

AD - Virginia Merrill Bloedel Hearing Research Center, Dept. of Otolaryngology and Head and Neck Surgery, University of Washington, Seattle, WA 98195, United States

Department of Physiology and Biophysics, University of Washington, Seattle, WA 98195, United States

AU - Rubel, E. W.

AU - Furrer, S. A.

AU - Stone, J. S.

C1 - Scopus

C2 - 23321648

DB - Scopus

DO - 10.1016/j.heares.2012.12.014

J2 - Hear. Res.

KW - Animals

Cell Transplantation

Ear, Inner

Epithelium

Gene Expression Profiling

Gene Expression Regulation, Developmental

Hair Cells, Auditory

Hearing Loss, Noise-Induced

Humans

Mitosis

Regeneration

Stem Cells

auditory system function

bird

cell cycle regulation

cell death

cell differentiation

cell division

cell maturation

cell population

cell regeneration

cell ultrastructure

hair cell

medical research

nonhuman

priority journal

review

signal transduction

synaptic potential

vestibular function

LA - English

M3 - Review

N1 - Export Date: 21 May 2026; Cited By: 152; Correspondence Address: E.W. Rubel; Virginia Merrill Bloedel Hearing Research Center, University of Washington, School of Medicine, Seattle, WA 98195-7923, Box 357923 CHDD Building, United States; email: rubel@uw.edu; CODEN: HERED

PY - 2013

SN - 18785891 (ISSN)

SP - 42-51

ST - A brief history of hair cell regeneration research and speculations on the future

T2 - Hearing Research

TI - A brief history of hair cell regeneration research and speculations on the future

UR - https://www.scopus.com/pages/publications/84875380031?origin=resultslist

VL - 297

ID - 39850

ER -

TY - JOUR

AB - To encode continuous sound stimuli, the inner hair cell (IHC) ribbon synapses utilize calcium-binding proteins (CaBPs), which reduce the inactivation of their Ca(V)1.3 calcium channels. Mutations in the CABP2 gene underlie non-syndromic autosomal recessive hearing loss DFNB93. Besides CaBP2, the structurally related CaBP1 is highly abundant in the IHCs. Here, we investigated how the two CaBPs cooperatively regulate IHC synaptic function. In Cabp1/2 double-knockout mice, we find strongly enhanced Ca(V)1.3 inactivation, slowed recovery from inactivation and impaired sustained exocytosis. Already mild IHC activation further reduces the availability of channels to trigger synaptic transmission and may effectively silence synapses. Spontaneous and sound-evoked responses of spiral ganglion neurons in vivo are strikingly reduced and strongly depend on stimulation rates. Transgenic expression of CaBP2 leads to substantial recovery of IHC synaptic function and hearing sensitivity. We conclude that CaBP1 and 2 act together to suppress voltage- and calcium-dependent inactivation of IHC Ca(V)1.3 channels in order to support sufficient rate of exocytosis and enable fast, temporally precise and indefatigable sound encoding.

AD - Experimental Otology Group, InnerEarLab, Department of Otolaryngology, University Medical Center Göttingen, Göttingen, Germany.

Auditory Neuroscience Group, Max Planck Institute for Multidisciplinary Sciences, Göttingen, Germany.

Auditory Systems Physiology Group, Institute for Auditory Neuroscience, InnerEarLab, University Medical Center Göttingen, Göttingen, Germany.

Collaborative Research Center 889, University of Göttingen, Göttingen, Germany.

Functional Auditory Genomics, Institute for Auditory Neuroscience, University Medical Center Göttingen, Göttingen, Germany.

Institute for Auditory Neuroscience and InnerEarLab, University Medical Center Göttingen, Göttingen, Germany.

Restorative Cochlear Genomics Group, Auditory Neuroscience and Optogenetics Laboratory, German Primate Cente, Göttingen, Germany.

Multiscale Bioimaging Cluster of Excellence (MBExC), University of Göttingen, Göttingen, Germany.

AN - 39718549

AU - Oestreicher, D.

AU - Chepurwar, S.

AU - Kusch, K.

AU - Rankovic, V.

AU - Jung, S.

AU - Strenzke, N.

AU - Pangrsic, T.

C1 - PubMed

C2 - PMC11668525

DA - Dec 24

DO - 10.7554/eLife.93646

DP - NLM

ET - 2024/12/24

KW - Animals

*Hair Cells, Auditory, Inner/metabolism/physiology

*Synaptic Transmission/physiology

Mice

*Mice, Knockout

*Calcium Channels, L-Type/metabolism/genetics

*Calcium-Binding Proteins/metabolism/genetics

Calcium/metabolism

Cacna1d

Dfnb93

calcium channel

hearing impairment

mouse

neuroscience

ribbon synapse

LA - eng

N1 - 2050-084x

Oestreicher, David

Orcid: 0000-0002-4541-6398

Chepurwar, Shashank

Kusch, Kathrin

Rankovic, Vladan

Jung, Sangyong

Strenzke, Nicola

Orcid: 0000-0003-1673-1046

Pangrsic, Tina

Orcid: 0000-0002-7313-1648

PP1608: PA 2769/1-1/Deutsche Forschungsgemeinschaft/

413501650/Deutsche Forschungsgemeinschaft/

CRC889: B09/Deutsche Forschungsgemeinschaft/

CRC889: A06/Deutsche Forschungsgemeinschaft/

EXC 2067/1-390729940/Multiscale Bioimaging Cluster of Excellence/

Journal Article

England

Elife. 2024 Dec 24;13:RP93646. doi: 10.7554/eLife.93646.

PY - 2024

SN - 2050-084x

ST - CaBP1 and 2 enable sustained Ca(V)1.3 calcium currents and synaptic transmission in inner hair cells

T2 - Elife

TI - CaBP1 and 2 enable sustained Ca(V)1.3 calcium currents and synaptic transmission in inner hair cells

VL - 13

ID - 39620

ER -

TY - JOUR

AB - The auditory sensory epithelium, composed of mechano-sensory hair cells (HCs) and highly specialized glial-like supporting cells (SCs), is critical for our ability to detect sound. SCs provide structural and functional support to HCs and play an essential role in cochlear development, homeostasis and repair. Despite their importance, however, surprisingly little is known about the molecular mechanisms guiding SC differentiation. Here, we provide evidence that in addition to its well-characterized inhibitory function, canonical Notch signaling plays a positive, instructive role in the differentiation of SCs. Using γ-secretase inhibitor DAPT to acutely block canonical Notch signaling, we identified a cohort of Notch-regulated SC-specific genes, with diverse functions in cell signaling, cell differentiation, neuronal innervation and synaptogenesis. We validated the newly identified Notch-regulated genes in vivo using genetic gain (Emx2(Cre/+); Rosa26(N1ICD/+)) and loss-of-function approaches (Emx2(Cre/+); Rosa26(DnMAML1/+)). Furthermore, we demonstrate that Notch over-activation in the differentiating murine cochlea (Emx2(Cre/+); Rosa26(N1ICD/+)) actively promotes a SC-specific gene expression program. Finally, we show that outer SCs -so called Deiters' cells are selectively lost by prolonged reduction (Emx2(Cre/+); Rosa26(DnMAML1/+/+)) or abolishment of canonical Notch signaling (Fgfr3-iCreER; Rbpj(-/Δ)), indicating a critical role for Notch signaling in Deiters' cell development.

AD - Solomon H. Snyder Department of Neuroscience, Johns Hopkins University, School of Medicine Baltimore, MD 21205, USA.

Center for Sensory Biology, Johns Hopkins University, School of Medicine Baltimore, MD 21205, USA.

AN - 26786414

AU - Campbell, D. P.

AU - Chrysostomou, E.

AU - Doetzlhofer, A.

C1 - PubMed

C2 - PMC4726253

DA - Jan 20

DO - 10.1038/srep19484

DP - NLM

ET - 2016/01/21

KW - Animals

Cell Count

Cell Death

Cell Differentiation/genetics

Cochlea/cytology/embryology/metabolism

Gene Expression Profiling

Gene Expression Regulation, Developmental

Labyrinth Supporting Cells/cytology/*metabolism

Mice

Mice, Transgenic

Nuclear Proteins/genetics/metabolism

Organ Specificity/genetics

Phenotype

Receptors, Notch/*metabolism

*Signal Transduction

Transcription Factors/genetics/metabolism

LA - eng

N1 - 2045-2322

Campbell, Dean P

Chrysostomou, Elena

Doetzlhofer, Angelika

DC011571/DC/NIDCD NIH HHS/United States

DC005211/DC/NIDCD NIH HHS/United States

T32GM007445/GM/NIGMS NIH HHS/United States

T32 GM007445/GM/NIGMS NIH HHS/United States

R01 DC011571/DC/NIDCD NIH HHS/United States

P30 DC005211/DC/NIDCD NIH HHS/United States

Journal Article

Research Support, N.I.H., Extramural

England

Sci Rep. 2016 Jan 20;6:19484. doi: 10.1038/srep19484.

PY - 2016

SN - 2045-2322

SP - 19484

ST - Canonical Notch signaling plays an instructive role in auditory supporting cell development

T2 - Sci Rep

TI - Canonical Notch signaling plays an instructive role in auditory supporting cell development

VL - 6

ID - 39703

ER -

TY - JOUR

AB - Sensory hair cells are the mechanotransductive receptors that detect gravity, sound, and vibration in all vertebrates. Damage to these sensitive receptors often results in deficits in vestibular function and hearing. There are currently two main reasons for studying the process of hair cell loss in fishes. First, fishes, like other non-mammalian vertebrates, have the ability to regenerate hair cells that have been damaged or lost via exposure to ototoxic chemicals or acoustic overstimulation. Thus, they are used as a biomedical model to understand the process of hair cell death and regeneration and find therapeutics that treat or prevent human hearing loss. Secondly, scientists and governmental natural resource managers are concerned about the potential effects of intense anthropogenic sounds on aquatic organisms, including fishes. Dr. Arthur N. Popper and his students, postdocs and research associates have performed pioneering experiments in both of these lines of fish hearing research. This review will discuss the current knowledge regarding the causes and consequences of both lateral line and inner ear hair cell damage in teleost fishes.

AD - Department of Biology, Western Kentucky University, Bowling Green, KY, 42101, USA. michael.smith1@wku.edu.

Department of Biology, Western Kentucky University, Bowling Green, KY, 42101, USA. jerry.monroe@wku.edu.

AN - 26515323

AU - Smith, M. E.

AU - Monroe, J. D.

C1 - PubMed

DO - 10.1007/978-3-319-21059-9_17

DP - NLM

ET - 2015/10/31

KW - Animals

Fishes/classification/*physiology

Hair Cells, Auditory/*pathology

Hearing/*physiology

Hearing Loss/*physiopathology

Humans

Lateral Line System/pathology/*physiopathology

Recovery of Function/physiology

Regeneration/physiology

Vestibule, Labyrinth/pathology/*physiopathology

Acoustic trauma

Anthropogenic sound

Fish

Hair cell

Hearing loss

Inner ear

Lateral line

Ototoxicity

Regeneration

LA - eng

N1 - Smith, Michael E

Monroe, J David

2 P20 GM103436-14/GM/NIGMS NIH HHS/United States

8 P20 GM103436-12/GM/NIGMS NIH HHS/United States

P20 RR-16481/RR/NCRR NIH HHS/United States

Journal Article

Research Support, N.I.H., Extramural

Research Support, Non-U.S. Gov't

Research Support, U.S. Gov't, Non-P.H.S.

Review

United States

Adv Exp Med Biol. 2016;877:393-417. doi: 10.1007/978-3-319-21059-9_17.

PY - 2016

SN - 0065-2598 (Print)

0065-2598

SP - 393-417

ST - Causes and Consequences of Sensory Hair Cell Damage and Recovery in Fishes

T2 - Adv Exp Med Biol

TI - Causes and Consequences of Sensory Hair Cell Damage and Recovery in Fishes

VL - 877

ID - 38878

ER -

TY - JOUR

AB - Sensory hair cell loss in the vestibular organs of the inner ear causes balance disorders which are essentially irreversible due to the lack of hair cell regeneration. Here, we administered a γ-secretase inhibitor to an adult mouse model of vestibular hair cell loss. The treatment regenerated type I and type II hair cells and restored canal and otolith afferent innervation, resulting in a complete recovery of rotational and translational vestibulo-ocular reflexes across all frequencies. Genetic deletion of Notch1 in supporting cells identified Notch1 as the target of the drug. The results demonstrate that a single injection of a γ-secretase inhibitor is a viable therapy for functional restoration of the vestibular system in patients with balance disorders.

AN - 41040353

AU - Lahlou, H.

AU - Zhu, H.

AU - Zhou, W.

AU - Edge, A. S. B.

C1 - PubMed

C2 - PMC12485952

DA - Sep 26

DO - 10.1101/2024.10.02.616053

DP - NLM

ET - 2025/10/03

LA - eng

N1 - 2692-8205

Lahlou, Hanae

Zhu, Hong

Zhou, Wu

Edge, Albert S B

Orcid: 0000-0001-8641-755x

Journal Article

Preprint

United States

bioRxiv [Preprint]. 2025 Sep 26:2024.10.02.616053. doi: 10.1101/2024.10.02.616053.

PY - 2025

SN - 2692-8205

ST - Cellular Reprogramming by Notch Inhibition Regenerates Hair Cells and Afferent Neurons to Restore the Sense of Balance

T2 - bioRxiv

TI - Cellular Reprogramming by Notch Inhibition Regenerates Hair Cells and Afferent Neurons to Restore the Sense of Balance

ID - 38961

ER -

TY - JOUR

AB - The inner ear contains six distinct sensory organs that each maintains some ability to regenerate hair cells into adulthood. In the postnatal cochlea, there appears to be a relationship between the developmental maturity of a region and its ability to regenerate as postnatal regeneration largely occurs in the apical turn, which is the last region to differentiate and mature during development. In the mature cristae there are also regional differences in regenerative ability, which led us to hypothesize that there may be a general relationship between the relative maturity of a region and the regenerative competence of that region in all of the inner ear sensory organs. By analyzing adult mouse cristae labeled embryonically with BrdU, we found that hair cell birth starts in the central region and progresses to the periphery with age. Since the peripheral region of the adult cristae also maintains active Notch signaling and some regenerative competence, these results are consistent with the hypothesis that the last regions to develop retain some of their regenerative ability into adulthood. Further, by analyzing embryonic day 14.5 inner ears we provide evidence for a wave of hair cell birth along the longitudinal axis of the cristae from the central regions to the outer edges. Together with the data from the adult inner ears labeled with BrdU as embryos, these results suggest that hair cell differentiation closely follows cell cycle exit in the cristae, unlike in the cochlea where they are uncoupled. © 2016 Elsevier Inc.

AD - Department of Biological Structure, University of Washington, Seattle, 98195, WA, United States

Graduate Program in Neuroscience, University of Washington, Seattle, 98195, WA, United States

Institute for Stem Cells and Regenerative Medicine, University of Washington, Seattle, 98109, WA, United States

AU - Slowik, A. D.

AU - Bermingham-McDonogh, O.

C1 - Scopus

C2 - 26826497

DB - Scopus

DO - 10.1016/j.ydbio.2016.01.033

IS - 1

J2 - Dev. Biol.

KW - Animals

Bromodeoxyuridine

Cell Cycle

Cell Differentiation

Cochlea

Female

Hair Cells, Ampulla

Hair Cells, Auditory

Hearing

Mice

Organogenesis

Receptors, Notch

Regeneration

Signal Transduction

Spatial Analysis

broxuridine

Notch receptor

adult

animal experiment

Article

auditory system

cell cycle progression

cell regeneration

central cell cycle progression

cochlea fenestra

competence

controlled study

embryo

hair cell

inner ear

male

mouse

nonhuman

perinatal period

peripheral cell cycle progression

priority journal

sense organ

animal

cochlear hair cell

cytology

embryology

metabolism

physiology

LA - English

M3 - Article

N1 - Export Date: 21 May 2026; Cited By: 4; Correspondence Address: O. Bermingham-McDonogh; Department of Biological Structure, University of Washington, Seattle, 98195, United States; email: oliviab@uw.edu; CODEN: DEBIA

PY - 2016

SN - 00121606 (ISSN)

SP - 1-14

ST - A central to peripheral progression of cell cycle exit and hair cell differentiation in the developing mouse cristae

T2 - Developmental Biology

TI - A central to peripheral progression of cell cycle exit and hair cell differentiation in the developing mouse cristae

UR - https://www.scopus.com/pages/publications/84959145880?origin=resultslist

VL - 411

ID - 39852

ER -

TY - JOUR

AB - Postnatal mammalian cochlear hair cells (HCs) can be regenerated by direct transdifferentiation or by mitotic regeneration from supporting cells through many pathways, including Atoh1, Wnt, Hedgehog and Notch signaling. However, most new HCs are immature HCs. In this study we used RNA-Seq analysis to compare the differences between the transcriptomes of Atoh1 overexpression-induced new HCs and the native HCs, and to define the factors that might help to promote the maturation of new HCs. As expected, we found Atoh1-induced new HCs had obvious HC characteristics as demonstrated by the expression of HC markers such as Pou4f3 and Myosin VIIA (Myo7a). However, Atoh1-induced new HCs had significantly lower expression of genes that are related to HC function such as Slc26a5 (Prestin), Slc17a8 and Otof. We found that genes related to HC cell differentiation and maturation (Kcnma1, Myo6, Myo7a, Grxcr1, Gfi1, Wnt5a, Fgfr1, Gfi1, Fgf8 etc.) had significantly lower expression levels in new HCs compared to native HCs. In conclusion, we found a set of genes that might regulate the differentiation and maturation of new HCs, and these genes might serve as potential new therapeutic targets for functional HC regeneration and hearing recovery.

AD - ENT Institute and Otorhinolaryngology Department of Affiliated Eye and ENT Hospital, Fudan University Shanghai 200031, China.

NHC Key Laboratory of Hearing Medicine (Fudan University) Shanghai 200031, China.

AN - 32211215

AU - Liu, L. M.

AU - Zhao, L. P.

AU - Wu, L. J.

AU - Guo, L.

AU - Li, W. Y.

AU - Chen, Y.

C1 - PubMed

C2 - PMC7076321

DP - NLM

ET - 2020/03/27

IS - 1

KW - Inner ears

RNA-Seq

cochlea

gene expression

hair cell maturation

hair cell regeneration

supporting cells

LA - eng

N1 - 2160-4150

Liu, Li-Man

Zhao, Li-Ping

Wu, Ling-Jie

Guo, Luo

Li, Wen-Yan

Chen, Yan

Journal Article

United States

Am J Stem Cells. 2020 Feb 15;9(1):1-15. eCollection 2020.

PY - 2020

SN - 2160-4150 (Print)

2160-4150

SP - 1-15

ST - Characterization of the transcriptomes of Atoh1-induced hair cells in the mouse cochlea

T2 - Am J Stem Cells

TI - Characterization of the transcriptomes of Atoh1-induced hair cells in the mouse cochlea

VL - 9

ID - 39115

ER -

TY - JOUR

AB - Hair cell-driven mechanosensory systems are crucial for successful execution of a number of behaviors in fishes, and have emerged as good models for exploring questions relevant to human hearing. This review focuses on ototoxic effects in the inner ear and lateral line system of fishes. We specifically examine studies where chemical ototoxins such as aminoglycoside antibiotics have been employed as tools to disable the lateral line. Lateral line ablation results in alterations to feeding behavior and orientation to water current in a variety of species. However, neither behavior is abolished in the presence of additional sensory cues, supporting the hypothesis that many fish behaviors are driven by multisensory integration. Within biomedical research, the larval zebrafish lateral line has become an important model system for understanding signaling mechanisms that contribute to hair cell death and for developing novel pharmacological therapies that protect hair cells from ototoxic damage. Furthermore, given that fishes robustly regenerate damaged hair cells, ototoxin studies in fishes have broadened our understanding of the molecular and genetic events in an innately regenerative system, offering potential targets for mammalian hair cell regeneration. Collectively, studies of fish mechanosensory systems have yielded insight into fish behavior and in mechanisms of hair cell death, protection, and regeneration.

AD - Department of Integrative Physiology and Neuroscience, Washington State University, Vancouver, WA, USA. Allison.coffin@wsu.edu.

Department of Biology, St. Mary's College of Maryland, St Marys City, MD, USA. jramcharitar@iau.edu.lc.

College of Medicine, International American University, Vieux Fort, St. Lucia, West-Indies. jramcharitar@iau.edu.lc.

AN - 26515324

AU - Coffin, A. B.

AU - Ramcharitar, J.

C1 - PubMed

DO - 10.1007/978-3-319-21059-9_18

DP - NLM

ET - 2015/10/31

KW - Aminoglycosides/toxicity

Animals

Anti-Bacterial Agents/toxicity

Ear, Inner/drug effects/pathology/*physiopathology

Fishes/classification/*physiology

Hair Cells, Auditory/drug effects/physiology

Humans

Larva/drug effects/physiology

Lateral Line System/*drug effects/pathology/*physiopathology

Zebrafish/*physiology

Auditory

Ear

Fish

Hair cell

Lateral line

LA - eng

N1 - Coffin, Allison B

Ramcharitar, John

DC011344/DC/NIDCD NIH HHS/United States

DC013900/DC/NIDCD NIH HHS/United States

Howard Hughes Medical Institute/United States

Intramural NIH HHS/United States

Journal Article

Research Support, N.I.H., Intramural

Research Support, Non-U.S. Gov't

Research Support, U.S. Gov't, Non-P.H.S.

Review

United States

Adv Exp Med Biol. 2016;877:419-37. doi: 10.1007/978-3-319-21059-9_18.

PY - 2016

SN - 0065-2598 (Print)

0065-2598

SP - 419-37

ST - Chemical Ototoxicity of the Fish Inner Ear and Lateral Line

T2 - Adv Exp Med Biol

TI - Chemical Ototoxicity of the Fish Inner Ear and Lateral Line

VL - 877

ID - 38818

ER -

TY - JOUR

AB - BACKGROUND: The autosomal recessive deafness 9 (DFNB9) is caused by mutations in the otoferlin gene that accounts for 2-8% of all inherited deafness cases. In a previous study, we demonstrated that Adeno-associated virus (AAV) gene therapy restored hearing in a preclinical mouse model of profound DFNB9 deafness caused by a frameshift mutation leading to a complete loss of otoferlin expression. However, it remains to be demonstrated that it can address the full spectrum of DFNB9 deafness severity, while also restoring central auditory processing essential for speech understanding. METHODS: Using homologous recombination in mouse embryonic stem cells, we created a knock-in mouse model carrying the E1799del otoferlin mutation, which mirrors the human E1804del variant linked to DFNB9 deafness, characterized by moderate-to-profound deafness during febrile episodes in affected individuals. A mixture of male and female mice was used at P2, P8, and P30. Some were followed for up to 4 months for longevity monitoring and behavioral tests. RESULTS: The mouse model exhibits abnormal otoferlin distribution, failure of synaptic transmission in inner hair cells, and profound hearing loss, all of which is restored to normal through AAV gene therapy. Notably, we conduct objective behavioral testing to provide the first evidence that gene therapy administered to the cochlea, which is part of the peripheral auditory system, can restore frequency discrimination, indicating the recovery of central auditory processing. This is achieved even when treatment is administered late at the end of the critical period. CONCLUSIONS: These findings indicate that gene therapy can address the entire spectrum of DFNB9 hearing loss, and that profound deafness during critical period may not impede the restoration of central auditory processing.

We investigated gene therapy, a technique that introduces a healthy copy of a gene to restore normal cell function, as a potential treatment for an unusual form of inherited deafness caused by mutations in the otoferlin gene. These mutations result in the production of a defective protein, leading to hearing loss during fever episodes. We developed and studied a mouse model with the same genetic alteration observed in affected individuals and discovered that the deafness resulted from the abnormal protein distribution in the inner ear. We use gene therapy to correct this mislocalization and restored normal hearing. Our findings indicate that gene therapy may be an effective approach for treating all forms of otoferlin-related hearing loss.

eng

AD - Université Paris Cité, Institut Pasteur, AP-HP, INSERM, CNRS, Fondation Pour l'Audition, Institut de l'Audition, IHU reConnect, Paris, F-75012, France.

Sorbonne Université, Collège Doctoral, Paris, F-75005, France.

Unité Fonctionnelle Implants Auditifs, ORL, GH Pitié-Salpêtrière, AP-HP Sorbonne Université, 75013, Paris, France.

Collège de France, Paris, F-75005, France.

University of California San Francisco, San Francisco, CA, 94115-1330, USA.

Université Paris Cité, Institut Pasteur, AP-HP, INSERM, CNRS, Fondation Pour l'Audition, Institut de l'Audition, IHU reConnect, Paris, F-75012, France. saaid.safieddine@pasteur.fr.

AN - 40506483

AU - Benamer, N.

AU - Le Ribeuz, H.

AU - Felgerolle, C.

AU - Calvet, C.

AU - Postal, O.

AU - Plion, B.

AU - Saenz-Roldan, M.

AU - Giorgi, M.

AU - Lecomte, M. J.

AU - Nguyen, Y.

AU - Petit, C.

AU - Michalski, N.

AU - Gourévitch, B.

AU - Akil, O.

AU - Safieddine, S.

C1 - PubMed

C2 - PMC12162826

DA - Jun 12

DO - 10.1038/s43856-025-00926-3

DP - NLM

ET - 2025/06/13

IS - 1

LA - eng

N1 - 2730-664x

Benamer, Najate

Orcid: 0000-0003-1646-1509

Le Ribeuz, Hélène

Felgerolle, Chloé

Orcid: 0000-0001-8824-375x

Calvet, Charlotte

Postal, Olivier

Plion, Baptiste

Saenz-Roldan, Mauricio

Orcid: 0009-0004-6240-5967

Giorgi, Marie

Orcid: 0000-0002-5505-7165

Lecomte, Marie-José

Orcid: 0000-0001-5097-6798

Nguyen, Yann

Petit, Christine

Orcid: 0000-0002-9069-002x

Michalski, Nicolas

Orcid: 0000-0002-1287-2709

Gourévitch, Boris

Akil, Omar

Safieddine, Saaid

Orcid: 0000-0002-6159-0572

Journal Article

England

Commun Med (Lond). 2025 Jun 12;5(1):229. doi: 10.1038/s43856-025-00926-3.

PY - 2025

SN - 2730-664x

SP - 229

ST - Cochlear gene therapy restores hearing and auditory processing in an atypical DFNB9 mouse model

T2 - Commun Med (Lond)

TI - Cochlear gene therapy restores hearing and auditory processing in an atypical DFNB9 mouse model

VL - 5

ID - 39490

ER -

TY - JOUR

AB - A role for connexin (Cx)30 in epithelial repair following injury was examined in the organ of Corti, the sensory epithelium of the cochlea. In this tissue, lesions caused by loss of the sensory hair cells are closed by the supporting cells that surround each one. Gap junctions in which Cx30 is the predominant connexin are large and numerous between supporting cells. In mice carrying a deletion in the gene (Gjb6) that encodes Cx30, the size and number of gap junction plaques, and the extent of dye transfer, between supporting cells was greatly reduced compared with normal animals. This corresponded with unique peculiarities of the lesion closure events during the progressive hair cell loss that occurs in these animals in comparison with other models of hair cell loss, whether acquired or as a result of a mutation. Only one, rather than all, of the supporting cells that contacted an individual dying hair closed the lesion, indicating disturbance of the co-ordination of cellular responses. The cell shape changes that the supporting cells normally undergo during repair of the organ of Corti did not occur. Also, there was disruption of the migratory activities that normally lead to the replacement of a columnar epithelium with a squamous-like one. These observations demonstrate a role for Cx30 and intercellular communication in regulating repair responses in an epithelial tissue.

AD - Centre for Auditory Research, UCL Ear Institute, London WC1X 8EE, UK. a.forge@ucl.ac.uk

AN - 23424196

AU - Forge, A.

AU - Jagger, D. J.

AU - Kelly, J. J.

AU - Taylor, R. R.

C1 - PubMed

DA - Apr 1

DO - 10.1242/jcs.125476

DP - NLM

ET - 2013/02/21

IS - Pt 7

KW - Animals

Cell Communication/genetics/physiology

Cochlea/*metabolism/ultrastructure

Connexin 30

Connexins/genetics/*metabolism

Gap Junctions/metabolism/ultrastructure

In Vitro Techniques

Mice

Mice, Knockout

Microscopy, Electron

Wound Healing/physiology

Cochlea

Connexin

Cx30

Gap junction

Wound healing

LA - eng

N1 - 1477-9137

Forge, Andrew

Jagger, Daniel J

Kelly, John J

Taylor, Ruth R

G1000068/MRC_/Medical Research Council/United Kingdom

BB/D009669/1/BB_/Biotechnology and Biological Sciences Research Council/United Kingdom

Journal Article

Research Support, Non-U.S. Gov't

England

J Cell Sci. 2013 Apr 1;126(Pt 7):1703-12. doi: 10.1242/jcs.125476. Epub 2013 Feb 19.

PY - 2013

SN - 0021-9533

SP - 1703-12

ST - Connexin30-mediated intercellular communication plays an essential role in epithelial repair in the cochlea

T2 - J Cell Sci

TI - Connexin30-mediated intercellular communication plays an essential role in epithelial repair in the cochlea

VL - 126

ID - 39484

ER -

TY - JOUR

AB - &lt;b&gt;Introduction:&lt;/b&gt; More than 5% of the world's population experience hearing impairment. The most common form is presbycusis (age-related hearing loss; ARHL). It affects almost one in three people over the age of 65. The hair cells of the cochlea play an important role in the process of sound registration. Genetic mutations, aging and environmental factors can cause damage that contributes to the hearing loss.&lt;b&gt;Methods and results:&lt;/b&gt; The currently explored research directions include drug treatments, gene therapies, and stem cell therapies. To date, no significant differences in the therapeutic effect depending on the route of corticosteroid administration have been demonstrated in patients with moderate to severe hearing loss. New dexamethasone-containing hydrogel formulations, as well as lipid formulations, thermosensitive polymers, and nanoparticles, have been developed to achieve high drug concentrations in the inner ear structures. Otoprotective effects of antioxidants or substances that modify the toxic effects of e.g. cisplatin, are also being studied. Attempts at auditory cells' regeneration seem promising in hearing loss research. Substances that regulate the central mechanisms of the Notch and Wnt pathways are being explored to this end. The genetic determinants of presbycusis suggest that interference at the level of specific genes may be a promising option for the treatment of this condition. With the CRISPR/Cas9 technology, the functions of inner ear genes can be effectively studied by disrupting normal gene alleles. The CRISPR/Cas9 complexes developed to target specific genes are delivered using cationic lipids, proteins, and viral vectors. They are then transported through the round window membrane by diffusion, without the need to surgically disrupt the inner ear. The potential of using antisense oligonucleotides to treat hereditary deafness caused by hair cell degeneration has also been established. Another research direction is related to stem cells being used for the development of in vitro 3D models of the human inner ear. Studies are also pursued to identify the mechanisms underlying the formation of cochlear organoids from pluripotent cells as well as determine the critical time points and events for cochlear sensory epithelial development and targeted hair cell differentiation.&lt;b&gt;Conclusions:&lt;/b&gt; In summary, significant progress has been made over the past decade in the search for novel therapies for sensory hearing loss. This line of research remains an ambitious and important area for further exploration.

AD - Chair and Department of Otorhinolaryngology, Head and Neck Surgery, Medical University of Warsaw, Poland.

Scientific Research Group at the Department of Otorhinolaryngology Head and Neck Surgery, Medical University of Warsaw, Poland.

Department of Otolaryngology, Pomeranian Medical University, Szczecin, Poland.

AN - 39051644

AU - Rzepakowska, A.

AU - Borowy, A.

AU - Siedlecki, E.

AU - Wolszczak, M.

AU - Radomska, K.

C1 - PubMed

DA - Jul 25

DP - NLM

ET - 2024/07/26

IS - 4

KW - Humans

*Genetic Therapy

Presbycusis/therapy

Hearing Loss, Sensorineural/therapy

Stem Cell Transplantation

Aged

gene therapy

hearing loss

new treatments

presbycusis

stem cells

LA - eng

N1 - 2300-8423

Rzepakowska, Anna

Borowy, Aleksandra

Siedlecki, Eryk

Wolszczak, Marta

Radomska, Katarzyna

Journal Article

Review

Poland

Otolaryngol Pol. 2024 Jul 25;78(4):29-38.

PY - 2024

SN - 0030-6657

SP - 29-38

ST - Contemporary directions in the therapy of sensory hearing loss

T2 - Otolaryngol Pol

TI - Contemporary directions in the therapy of sensory hearing loss

VL - 78

ID - 39679

ER -

TY - JOUR

AB - Clinical translation of gene therapy has been challenging, due to limitations in current delivery vehicles such as traditional viral vectors. Herein, we report the use of gRNA:Cas9 ribonucleoprotein (RNP) complexes engineered extracellular vesicles (EVs) for in vivo gene therapy. By leveraging a novel high-throughput microfluidic droplet-based electroporation system (μDES), we achieved 10-fold enhancement of loading efficiency and more than 1000-fold increase in processing throughput on loading RNP complexes into EVs (RNP-EVs), compared with conventional bulk electroporation. The flow-through droplets serve as enormous bioreactors for offering millisecond pulsed, low-voltage electroporation in a continuous-flow and scalable manner, which minimizes the Joule heating influence and surface alteration to retain natural EV stability and integrity. In the Shaker-1 mouse model of dominant progressive hearing loss, we demonstrated the effective delivery of RNP-EVs into inner ear hair cells, with a clear reduction of Myo7a(sh1) mRNA expression compared to RNP-loaded lipid-like nanoparticles (RNP-LNPs), leading to significant hearing recovery measured by auditory brainstem responses (ABR).

AD - College of Pharmacy, Department of Pharmaceutics, University of Florida, Gainesville, Florida 32611, United States.

Department of Otolaryngology, Head and Neck Surgery, University of Kansas School of Medicine, Kansas City, Kansas 66160, United States.

Department of Medicinal Chemistry, Center for Natural Products, Drug Discovery and Development, University of Florida, Gainesville, Florida, 32610, United States.

Department of Otolaryngology, Hannover Medical School, 30625 Hannover, Germany.

AN - 38168224

AU - Pan, X.

AU - Huang, P.

AU - Ali, S. S.

AU - Renslo, B.

AU - Hutchinson, T. E.

AU - Erwin, N.

AU - Greenberg, Z.

AU - Ding, Z.

AU - Li, Y.

AU - Warnecke, A.

AU - Fernandez, N. E.

AU - Staecker, H.

AU - He, M.

C1 - PubMed

C2 - PMC10760051

DA - Dec 15

DO - 10.1101/2023.09.14.557853

DP - NLM

ET - 2024/01/04

LA - eng

N1 - 2692-8205

Pan, Xiaoshu

Huang, Peixin

Ali, Samantha S

Renslo, Bryan

Hutchinson, Tarun E

Erwin, Nina

Greenberg, Zachary

Ding, Zuo

Li, Yanjun

Warnecke, Athanasia

Fernandez, Natalia E

Staecker, Hinrich

He, Mei

R35 GM133794/GM/NIGMS NIH HHS/United States

Journal Article

Preprint

United States

bioRxiv [Preprint]. 2023 Dec 15:2023.09.14.557853. doi: 10.1101/2023.09.14.557853.

PY - 2023

SN - 2692-8205

ST - CRISPR-Cas9 Engineered Extracellular Vesicles for the Treatment of Dominant Progressive Hearing Loss

T2 - bioRxiv

TI - CRISPR-Cas9 Engineered Extracellular Vesicles for the Treatment of Dominant Progressive Hearing Loss

ID - 39212

ER -

TY - JOUR

AB - The number of patients with hearing loss is on the rise due to congenital abnormalities, degenerative changes in old age, and acquired injuries such as virus or ototoxic drug-induced diseases. Hearing loss is a refractory and disabling disease that has serious negative effects on quality of life. The pathology of hearing loss in the inner ear is characterized by varying degrees of damage to the cochlear sensory epithelium cells (such as hair cells and supporting cells), stria vascularis (including marginal, intermediate and basal cells) and spiral ganglion neurons. Regeneration or direct repair of damaged cells in the inner ear is an effective way to treat sensorineural deafness. It is currently possible to regenerate hair cells to treat sensorineural hearing loss by FX-322, a small molecule drug in clinical trials. With the development of genetic engineering technology, gene therapy has brought a promising treatment strategy for many previously intractable diseases. Gene therapy has been regarded as a promising method in the treatment and rehabilitation of sensorineural hearing loss, and recombinant adeno-associated virus gene therapy has been widely used in fundamental research into hearing loss treatments. At present, gene therapy for hearing loss is transitioning from feasibility studies to explorations of its safety and its therapeutic potential. The present article reviews the concepts, strategies, and applications of gene therapy mediated by recombinant adeno-associated viruses in the field of hearing loss treatment.

AD - State Key Laboratory of Digital Medical Engineering, Department of Otolaryngology Head and Neck Surgery, Zhongda Hospital, School of Life Sciences and Technology, School of Medicine, Advanced Institute for Life and Health, Jiangsu Province High-Tech Key Laboratory for Bio-Medical Research, Southeast University, Nanjing 210096, China.

Department of Neurology, Aerospace Center Hospital, School of Life Science, Beijing Institute of Technology, Beijing 100081, China.

State Key Laboratory of Hearing and Balance Science, Beijing Institute of Technology, Beijing 100081, China.

Advanced Technology Research Institute, Beijing Institute of Technology, Jinan 250300, China.

Department of Otorhinolaryngology-Head and Neck Surgery, Zhongnan Hospital of Wuhan University, Wuhan 430071, China.

Department of Otolaryngology, Shandong Provincial Hospital Affiliated to Shandong First Medical University, Jinan 250021, China.

Medical Science and Technology Innovation Center, Shandong First Medical University & Shandong Academy of Medical Sciences, Jinan 250021, China.

Co-Innovation Center of Neuroregeneration, Nantong University, Nantong 226001, China.

Department of Otolaryngology Head and Neck Surgery, Sichuan Provincial People's Hospital, University of Electronic Science and Technology of China, Chengdu 610072, China.

Southeast University Shenzhen Research Institute, Shenzhen 518063, China.

AN - 40166123

AU - Qi, J.

AU - Fu, X.

AU - Zhang, L.

AU - Tan, F.

AU - Li, N.

AU - Sun, Q.

AU - Hu, X.

AU - He, Z.

AU - Xia, M.

AU - Chai, R.

C1 - PubMed

C2 - PMC11955060

DA - Jan

DO - 10.1016/j.fmre.2022.08.015

DP - NLM

ET - 2022/09/07

IS - 1

KW - Adeno-associated virus

Auditory repair

Cochlea

Gene therapy

Hearing loss

LA - eng

N1 - 2667-3258

Qi, Jieyu

Fu, Xiaolong

Zhang, Liyan

Tan, Fangzhi

Li, Nianci

Sun, Qiuhan

Hu, Xiaojie

He, Zuhong

Xia, Ming

Chai, Renjie

Journal Article

Review

Netherlands

Fundam Res. 2022 Sep 7;5(1):192-202. doi: 10.1016/j.fmre.2022.08.015. eCollection 2025 Jan.

PY - 2025

SN - 2096-9457 (Print)

2667-3258

SP - 192-202

ST - Current AAV-mediated gene therapy in sensorineural hearing loss

T2 - Fundam Res

TI - Current AAV-mediated gene therapy in sensorineural hearing loss

VL - 5

ID - 38998

ER -

TY - JOUR

AB - Hair cells (HCs) are mechanosensors that play crucial roles in perceiving sound, acceleration, and fluid motion. The precise architecture of the auditory epithelium and its repair after HC loss is indispensable to the function of organ of Corti (OC). In this study, we showed that Brg1 was highly expressed in auditory HCs. Specific deletion of Brg1 in postnatal HCs resulted in rapid HC degeneration and profound deafness in mice. Further experiments showed that cell-intrinsic polarity of HCs was abolished, docking of outer hair cells (OHCs) by Deiter's cells (DCs) failed, and scar formation in the reticular lamina was deficient. We demonstrated that Brg1 ablation disrupted the Gαi/Insc/LGN and aPKC asymmetric distributions, without overt effects on the core planer cell polarity (PCP) pathway. We also demonstrated that Brg1-deficient HCs underwent apoptosis, and that leakage in the reticular lamina caused by deficient scar formation shifted the mode of OHC death from apoptosis to necrosis. Together, these data demonstrated a requirement for Brg1 activity in HC development and suggested a role for Brg1 in the proper cellular structure formation of HCs.

AD - School of Life Science and Key Laboratory of the Ministry of Education for Experimental Teratology, Shandong University, Jinan 250100, China.

Department of Otolaryngology-Head and Neck Surgery, The Second Hospital of Shandong University, Jinan 250033, China.

AN - 27255603

AU - Jin, Y.

AU - Ren, N.

AU - Li, S.

AU - Fu, X.

AU - Sun, X.

AU - Men, Y.

AU - Xu, Z.

AU - Zhang, J.

AU - Xie, Y.

AU - Xia, M.

AU - Gao, J.

C1 - PubMed

C2 - PMC4891731

DA - Jun 3

DO - 10.1038/srep27124

DP - NLM

ET - 2016/06/04

KW - Animals

Animals, Newborn

Apoptosis

Cell Polarity

Cicatrix/*genetics/metabolism

Cochlea/*injuries

DNA Helicases/*genetics/metabolism

Deafness/*genetics

*Gene Deletion

Hair Cells, Auditory/*cytology/metabolism/pathology

Hair Cells, Auditory, Outer/cytology/metabolism

Mice

Necrosis

Nuclear Proteins/*genetics/metabolism

Signal Transduction

Transcription Factors/*genetics/metabolism

LA - eng

N1 - 2045-2322

Jin, Yecheng

Ren, Naixia

Li, Shiwei

Fu, Xiaolong

Sun, Xiaoyang

Men, Yuqin

Xu, Zhigang

Zhang, Jian

Xie, Yue

Xia, Ming

Gao, Jiangang

Journal Article

Research Support, Non-U.S. Gov't

England

Sci Rep. 2016 Jun 3;6:27124. doi: 10.1038/srep27124.

PY - 2016

SN - 2045-2322

SP - 27124

ST - Deletion of Brg1 causes abnormal hair cell planer polarity, hair cell anchorage, and scar formation in mouse cochlea

T2 - Sci Rep

TI - Deletion of Brg1 causes abnormal hair cell planer polarity, hair cell anchorage, and scar formation in mouse cochlea

VL - 6

ID - 39622

ER -

TY - JOUR

AB - Despite the significant impact of hearing and balance disorders on the general population there are currently no dedicated pharmaceuticals that target the inner ear. Advances in molecular biology and neuroscience have improved our understanding of the inner ear allowing the development of a range of molecular targets that have the potential to treat both hearing and balance disorders. One of the principal advantages of the inner ear is that it is accessible through a variety of approaches that would allow a potential to be delivered locally rather than systemically. This significantly broadens the potential medications that can be developed and opens the possibility of local gene delivery as a therapeutic intervention. Several potential clinical targets have been identified including delivery of neurotrophin expressing genes as an adjunct to cochlear implantation, delivery of protective genes to prevent trauma and the development of strategies for regenerating inner ear sensory cells. In order to translate these potential therapeutics into humans we will want to optimize the gene delivery methodology, dosing and activity of the drug for therapeutic value. To this end we have developed a series of adenovectors that efficiently transduce the inner ear. The use of these gene delivery approaches are attractive for the potential of hair cell regeneration after loss induced by trauma or ototoxins. This approach is particularly suited for the development of molecular therapies targeted at the vestibular system given that no device based therapeutic such a cochlear implant available for vestibular loss. © 2011 Elsevier B.V.

AD - Dept. Otolaryngology Head and Neck Surgery, University of Kansas School of Medicine, Kansas City, KS 66209, MS 3010, 3901 Rainbow Blvd., United States

Dept. Otolaryngology, University of Heidelberg, Heidelberg, Germany

GenVec Inc., Gaithersburg, MD, United States

AU - Staecker, H.

AU - Praetorius, M.

AU - Brough, D. E.

C1 - Scopus

C2 - 21251965

DB - Scopus

DO - 10.1016/j.heares.2011.01.006

IS - 1-2

J2 - Hear. Res.

KW - Adenoviridae

Animals

Basic Helix-Loop-Helix Transcription Factors

Ear, Inner

Gene Targeting

Gene Therapy

Gene Transfer Techniques

Genetic Vectors

Hair Cells, Auditory

Humans

Labyrinth Diseases

Models, Animal

Regeneration

Translational Research

Vestibular Diseases

basic helix loop helix transcription factor

parvovirus vector

protein atoh1

protein math1

unclassified drug

cell regeneration

disease model

gene vector

hair cell

human

immune response

inner ear disease

molecularly targeted therapy

nonhuman

priority journal

review

vestibular disorder

viral gene delivery system

viral gene therapy

LA - English

M3 - Review

N1 - Export Date: 21 May 2026; Cited By: 27; Correspondence Address: H. Staecker; Dept. Otolaryngology Head and Neck Surgery, University of Kansas School of Medicine, Kansas City, KS 66209, MS 3010, 3901 Rainbow Blvd., United States; email: hstaecker@kumc.edu; CODEN: HERED

PY - 2011

SN - 18785891 (ISSN)

SP - 44-51

ST - Development of gene therapy for inner ear disease: Using bilateral vestibular hypofunction as a vehicle for translational research

T2 - Hearing Research

TI - Development of gene therapy for inner ear disease: Using bilateral vestibular hypofunction as a vehicle for translational research

UR - https://www.scopus.com/pages/publications/79957942610?origin=resultslist

VL - 276

ID - 39855

ER -

TY - JOUR

AB - Hearing loss, as a sensory disorder, is the most common occurrence among humans, which has received increasing attention from society. It is mainly caused by the damage of inner ear hair cells (HCs) or the degeneration of spiral ganglion neurons. In mammals, cochlear HCs cannot regenerate naturally after injury, leading to irreversible hearing loss. Therefore, HCs are essential for hearing protection. In recent years, the protection of drug-related ototoxicity, inner ear stem cells, gene therapy, new materials, and signal regulation have become important ways to develop regeneration strategies of HCs. An in-depth study of the causes of the occurrence and development of hearing impairment and the regeneration of hearing loss for effective prevention, discovery, and treatment of deafness has great significance. This review aimed to analyze the development of the inner ear and summarize the related factors leading to HCs injury and the research progress of regeneration after injury.

AD - Department of Otolaryngology, Shandong Provincial Hospital, Shandong University, Jinan 250021, China.

Department of Central Laboratory, Shandong Provincial Hospital Affiliated to Shandong First Medical University, Jinan 250021, China.

Medical Science and Technology Innovation Center, Shandong First Medical University & Shandong Academy of Medical Sciences, Jinan 271000, China.

Department of Otolaryngology, Shandong Provincial Hospital Affiliated to Shandong First Medical University, Jinan 250021, China.

NHC Key Laboratory of Otorhinolaryngology, Jinan 250021, China.

Department of Pathology, Shandong Cancer Hospital and Institute, Shandong First Medical University and Shandong Academy of Medical Sciences, Jinan 250117, China.

AN - 40166090

AU - Bing, X.

AU - Liu, C.

AU - Cao, X.

AU - Li, C.

AU - Gao, X.

AU - Zhu, F.

AU - Wu, X.

AU - Guo, N.

AU - Hu, H.

AU - Xia, M.

AU - Zhao, M.

C1 - PubMed

C2 - PMC11955025

DA - Jan

DO - 10.1016/j.fmre.2023.09.005

DP - NLM

ET - 2023/11/21

IS - 1

KW - Development

Hearing impairment

Hearing protection

Inner ear hair cells

Regeneration

LA - eng

N1 - 2667-3258

Bing, Xin

Liu, Chengcheng

Cao, Xue

Li, Chengzhilin

Gao, Xiaochen

Zhu, Fangyuan

Wu, Xinhao

Guo, Na

Hu, Houyang

Xia, Ming

Zhao, Miaoqing

Journal Article

Review

Netherlands

Fundam Res. 2023 Nov 21;5(1):203-214. doi: 10.1016/j.fmre.2023.09.005. eCollection 2025 Jan.

PY - 2025

SN - 2096-9457 (Print)

2667-3258

SP - 203-214

ST - Development of the inner ear and regeneration of hair cells after hearing impairment

T2 - Fundam Res

TI - Development of the inner ear and regeneration of hair cells after hearing impairment

VL - 5

ID - 38930

ER -

TY - JOUR

AB - Precise and efficient endocytosis is critical for sustained neurotransmission during continuous neuronal activity. Endocytosis is a prerequisite for maintaining the auditory function. However, the differences between the patterns of endocytosis in cochlear inner hair cells (IHCs) and outer hair cells (OHCs) remain unclear. Both IHCs and OHCs were obtained from adult C57 mice. Patterns of endocytosis in cells were estimated by analyzing the uptake of FM1-43, a fluorescent. The observations were made using live confocal imaging, fluorescence intensities were calculated statistically. Results revealed the details about following phenomenon, i) sites of entry: the FM1-43 dye was found to enter IHC at the apical area initially, the additional sites of entry were then found at basolateral membrane of the cells, The entry of the dye into OHCs initially appeared to be occurring around whole apical membranes area, which then diffused towards the other membrane surface of the cells, ii) capacity of endocytosis: fluorescence intensity in IHCs showed significantly higher than that of OHCs (P<0.01). We have found different patterns of endocytosis between IHCs and OHCs, this indicated functional distinctions between them. Moreover, FM1-43 dye can be potentially used as an indicator of the functional loss or repair of cochlear hair cells.

AD - Department of Otolaryngology-Head and Neck Surgery, Affiliated Hospital of North Sichuan Medical College, Nanchong, Sichuan, China.

AN - 31177790

AU - Li, S.

AU - Yu, S.

AU - Ding, T.

AU - Yan, A.

AU - Qi, Y.

AU - Gong, S.

AU - Tang, S.

AU - Liu, K.

C1 - PubMed

DA - Aug 29

DO - 10.33549/physiolres.934009

DP - NLM

ET - 2019/06/11

IS - 4

KW - Animals

Biological Transport/physiology

Endocytosis/*physiology

Evoked Potentials, Auditory/physiology

Fluorescent Dyes/analysis/metabolism

Hair Cells, Auditory, Inner/chemistry/*metabolism

Hair Cells, Auditory, Outer/chemistry/*metabolism

Mice

Mice, Inbred C57BL

Organ Culture Techniques

Pyridinium Compounds/analysis/metabolism

Quaternary Ammonium Compounds/analysis/metabolism

LA - eng

N1 - 1802-9973

Li, Sijun

Yu, Shukui

Ding, Tonghui

Yan, Aihui

Qi, Yue

Gong, Shusheng

Tang, Siquan

Liu, Ke

Journal Article

Czech Republic

Physiol Res. 2019 Aug 29;68(4):659-665. doi: 10.33549/physiolres.934009. Epub 2019 Jun 6.

PY - 2019

SN - 0862-8408

SP - 659-665

ST - Different patterns of endocytosis in cochlear inner and outer hair cells of mice

T2 - Physiol Res

TI - Different patterns of endocytosis in cochlear inner and outer hair cells of mice

VL - 68

ID - 39027

ER -

TY - JOUR

AB - Dysferlin direct protein-protein interactions (PPI) previously have been elucidated with surface plasmon resonance (SPR) and predicted to underlie membrane repair in mechanotransducing myofibrils. In mechanotransducing inner ear hair cells, dysferlin is detected with Z-stack confocal immunofluorescence in the stereocilia and their inserts in the tectorial membrane (TM) co-localizing with FKBP8, consistent with the SPR determination of tight, positively Ca(2+)-dependent interaction. FKBP8, a direct binding partner of mechanotransducing TMC1, when overexpressed, evokes an elevation in anti-apoptotic BCL2, inhibition of ryanodine receptor (RYR) activity, and a consequent reduction in Ca(2+) release. RYR3 has now been immunolocalized to the tip of the TM in close association with a third-row outer hair cell (OHC) stereociliary BCL2-positive insertion. Dysferlin, annexin A2, and Alzheimer's proteins BACE1 and amyloid precursor protein (APP) are also accumulated in these stereociliary insertions. RYR2 and RYR1 have been immunolocalized to the TM core, in position to influence TM Ca(2+). Dysferlin PPI pathways also intersect with AD protein pathways in the spiral ganglion (SG). Dysferlin segregates with FKBP8, BACE1, and RYR3 in the interiors of SG type I cell bodies. RYR1, RYR2, PSEN1, BCL2, and caspase 3 are primarily confined to plasma membrane sites. RYR3 pathways traverse the plasma membrane to the cell body interior. Western analysis of dysferlinopathy proteins links FKBP8 and BCL2 overexpression with RYR inhibition, indicative of dysferlin targets that are ameliorative in AD.

AD - Laboratory of Bio-Otology, Department of Otolaryngology, Wayne State University School of Medicine, Detroit, MI 48201, USA.

Department of Biochemistry, Microbiology and Immunology, Wayne State University School of Medicine, Detroit, MI 48201, USA.

AN - 41096823

AU - Drescher, M. J.

AU - Drescher, D. G.

AU - Khan, K. M.

AU - Hatfield, J. S.

AU - Hemani, D.

C1 - PubMed

C2 - PMC12524998

DA - Sep 30

DO - 10.3390/ijms26199559

DP - NLM

ET - 2025/10/16

IS - 19

KW - Humans

*Dysferlin/metabolism

Animals

*Spiral Ganglion/metabolism

*Alzheimer Disease/metabolism

*Organ of Corti/metabolism

*Protein Interaction Maps

Calcium/metabolism

Signal Transduction

Alzheimer’s disease

Bcl2

Fkbp8

dysferlin protein–protein interactions

ryanodine receptors

surface plasmon resonance

tectorial membrane

LA - eng

N1 - 1422-0067

Drescher, Marian J

Drescher, Dennis G

Khan, Khalid M

Hatfield, James S

Hemani, Darshi

DC004076, DC000156/Jain Foundation and NIH Grants/

Journal Article

Switzerland

Int J Mol Sci. 2025 Sep 30;26(19):9559. doi: 10.3390/ijms26199559.

PY - 2025

SN - 1422-0067

ST - Dysferlin Protein-Protein Interaction Pathways in the Organ of Corti and Spiral Ganglion Intersect with Alzheimer's Protein Pathways

T2 - Int J Mol Sci

TI - Dysferlin Protein-Protein Interaction Pathways in the Organ of Corti and Spiral Ganglion Intersect with Alzheimer's Protein Pathways

VL - 26

ID - 39168

ER -

TY - JOUR

AB - Aminoglycoside antibiotics are "the drug of choice" for treating many bacterial infections, but their administration results in hearing loss in up to one fourth of the patients who receive them. Several biochemical pathways have been implicated in aminoglycoside antibiotic ototoxicity; however, little is known about how hair cells respond to aminoglycoside antibiotics at the transcriptome level. Here we have investigated the genome-wide response to the aminoglycoside antibiotic gentamicin. Using organotypic cultures of the perinatal organ of Corti, we performed RNA sequencing using cDNA libraries obtained from FACS-purified hair cells. Within 3 h of gentamicin treatment, the messenger RNA level of more than three thousand genes in hair cells changed significantly. Bioinformatic analysis of these changes highlighted several known signal transduction pathways, including the JNK pathway and the NF-κB pathway, in addition to genes involved in the stress response, apoptosis, cell cycle control, and DNA damage repair. In contrast, only 698 genes, mainly involved in cell cycle and metabolite biosynthetic processes, were significantly affected in the non-hair cell population. The gene expression profiles of hair cells in response to gentamicin share a considerable similarity with those previously observed in gentamicin-induced nephrotoxicity. Our findings suggest that previously observed early responses to gentamicin in hair cells in specific signaling pathways are reflected in changes in gene expression. Additionally, the observed changes in gene expression of cell cycle regulatory genes indicate a disruption of the postmitotic state, which may suggest an alternate pathway regulating gentamicin-induced apoptotic hair cell death. This work provides a more comprehensive view of aminoglycoside antibiotic ototoxicity, and thus contributes to identifying potential pathways or therapeutic targets to alleviate this important side effect of aminoglycoside antibiotics.

AD - Genetic, Molecular and Cellular Biology Program, University of Southern California Los Angeles, CA, USA ; Department of Stem Cell Biology and Regenerative Medicine, University of Southern California Los Angeles, CA, USA.

Genetic, Molecular and Cellular Biology Program, University of Southern California Los Angeles, CA, USA ; Department of Stem Cell Biology and Regenerative Medicine, University of Southern California Los Angeles, CA, USA ; Department of Otolaryngology, University of Southern California Los Angeles, CA, USA.

AN - 26052268

AU - Tao, L.

AU - Segil, N.

C1 - PubMed

C2 - PMC4439550

DO - 10.3389/fncel.2015.00190

DP - NLM

ET - 2015/06/09

KW - RNA sequencing

aminoglycoside antibiotics

differential gene expression

gentamicin

ototoxicity

sensory hair cells

LA - eng

N1 - 1662-5102

Tao, Litao

Segil, Neil

R01 DC007173/DC/NIDCD NIH HHS/United States

Journal Article

Switzerland

Front Cell Neurosci. 2015 May 21;9:190. doi: 10.3389/fncel.2015.00190. eCollection 2015.

PY - 2015

SN - 1662-5102 (Print)

1662-5102

SP - 190

ST - Early transcriptional response to aminoglycoside antibiotic suggests alternate pathways leading to apoptosis in sensory hair cells in the mouse inner ear

T2 - Front Cell Neurosci

TI - Early transcriptional response to aminoglycoside antibiotic suggests alternate pathways leading to apoptosis in sensory hair cells in the mouse inner ear

VL - 9

ID - 39525

ER -

TY - JOUR

AB - BACKGROUND AND PURPOSE: Cisplatin-induced sensorineural hearing loss is a significant clinical challenge. Although the potential effects of brain-derived neurotrophic factor (BDNF) have previously been investigated in some ototoxicity models, its efficacy in cisplatin-induced hearing loss remains uncertain. This study aimed to investigate the therapeutic potential of recombinant human BDNF (rhBDNF) in protecting cells against cisplatin-induced ototoxicity. EXPERIMENTAL APPROACH: Using an in vivo model of cisplatin-induced hearing loss, we investigated the beneficial effects of transtympanic administration of rhBDNF in a thermogel solution on hearing function and cochlear injury, using electrophysiological, morphological, immunofluorescence and molecular analyses. KEY RESULTS: Our data showed that local rhBDNF treatment counteracted hearing loss in rats receiving cisplatin by preserving synaptic connections in the cochlear epithelium and protecting hair cells (HCs) and spiral ganglion neurons (SGNs) against cisplatin-induced cell death. Specifically, rhBDNF maintains the balance of its receptor levels (pTrkB and p75), boosting TrkB-CREB pro-survival signalling and reducing caspase 3-dependent apoptosis in the cochlea. Additionally, it activates antioxidant mechanisms while inhibiting inflammation and promoting vascular repair. CONCLUSION AND IMPLICATIONS: Collectively, we demonstrated that early transtympanic treatment with rhBDNF plays a multifaceted protective role against cisplatin-induced ototoxicity, thus holding promise as a novel potential approach to preserve hearing in adult and paediatric patients undergoing cisplatin-based chemotherapy.

AD - Department of Neuroscience, Unit of Audiology, Università degli Studi di Napoli Federico II, Naples, Italy.

Department of Head and Neck Surgery, Università Cattolica del Sacro Cuore, Rome, Italy.

Research & Early Development, Dompé Farmaceutici S.p.A., L'Aquila, Italy.

Department of Neuroscience, Università Cattolica del Sacro Cuore, Rome, Italy.

Fondazione Policlinico Universitario A. Gemelli IRCCS, Rome, Italy.

AN - 39390645

AU - Pisani, A.

AU - Rolesi, R.

AU - Mohamed-Hizam, V.

AU - Montuoro, R.

AU - Paludetti, G.

AU - Giorgio, C.

AU - Cocchiaro, P.

AU - Brandolini, L.

AU - Detta, N.

AU - Sirico, A.

AU - Amendola, P. G.

AU - Novelli, R.

AU - Aramini, A.

AU - Allegretti, M.

AU - Paciello, F.

AU - Grassi, C.

AU - Fetoni, A. R.

C1 - PubMed

DA - Feb

DO - 10.1111/bph.17359

DP - NLM

ET - 2024/10/11

IS - 3

KW - *Cisplatin/administration & dosage/toxicity

Animals

*Brain-Derived Neurotrophic Factor/administration & dosage/metabolism

*Neuroprotective Agents/administration & dosage/pharmacology

Rats

*Recombinant Proteins/administration & dosage

Male

*Rats, Sprague-Dawley

Humans

Hearing Loss/chemically induced/prevention & control/drug therapy

Cochlea/drug effects/metabolism/pathology

Receptor, trkB/metabolism

cisplatin

ototoxicity

rhBDNF

sensorineural hearing loss

LA - eng

N1 - 1476-5381

Pisani, Anna

Rolesi, Rolando

Mohamed-Hizam, Veronica

Montuoro, Raffaele

Paludetti, Gaetano

Giorgio, Cristina

Cocchiaro, Pasquale

Brandolini, Laura

Detta, Nicola

Sirico, Anna

Amendola, Pier Giorgio

Novelli, Rubina

Aramini, Andrea

Allegretti, Marcello

Paciello, Fabiola

Grassi, Claudio

Fetoni, Anna Rita

NEXTGENERATIONEU (NGEU) Ministry of University and Research/

#nextgenerationeu (ngeu)/

PE0000006/project MNESYS/

D3.1 2024/Università Cattolica del Sacro Cuore/

Journal Article

England

Br J Pharmacol. 2025 Feb;182(3):546-563. doi: 10.1111/bph.17359. Epub 2024 Oct 10.

PY - 2025

SN - 0007-1188

SP - 546-563

ST - Early transtympanic administration of rhBDNF exerts a multifaceted neuroprotective effect against cisplatin-induced hearing loss

T2 - Br J Pharmacol

TI - Early transtympanic administration of rhBDNF exerts a multifaceted neuroprotective effect against cisplatin-induced hearing loss

VL - 182

ID - 39757

ER -

TY - JOUR

AB - There is considerable interest in whether sensory deficiency is associated with the development of Alzheimer's disease (AD). Notably, the relationship between hearing impairment and AD is of high relevance but still poorly understood. In this study, we found early-onset hearing loss in two AD mouse models, 3xTgAD and 3xTgAD/Polβ(+/-). The 3xTgAD/Polβ(+/-) mouse is DNA repair deficient and has more humanized AD features than the 3xTgAD. Both AD mouse models showed increased auditory brainstem response (ABR) thresholds between 16 and 32 kHz at 4 weeks of age, much earlier than any AD cognitive and behavioral changes. The ABR thresholds were significantly higher in 3xTgAD/Polβ(+/-) mice than in 3xTgAD mice at 16 kHz, and distortion product otoacoustic emission signals were reduced, indicating that DNA damage may be a factor underlying early hearing impairment in AD. Poly ADP-ribosylation and protein expression levels of DNA damage markers increased significantly in the cochlea of the AD mice but not in the adjacent auditory cortex. Phosphoglycerate mutase 2 levels and the number of synaptic ribbons in the presynaptic zones of inner hair cells were decreased in the cochlea of the AD mice. Furthermore, the activity of sirtuin 3 was downregulated in the cochlea of these mice, indicative of impaired mitochondrial function. Taken together, these findings provide new insights into potential mechanisms for hearing dysfunction in AD and suggest that DNA damage in the cochlea might contribute to the development of early hearing loss in AD.

AD - DNA repair Section, National Institute on Aging, National Institutes of Health, Baltimore, MD, USA.

National Institute on Deafness and Other Communication Disorders, National Institutes of Health, Bethesda, MD, USA.

Department of Otolaryngology-Head & Neck Surgery, Johns Hopkins School of Medicine, Baltimore, MD, USA.

Waitt Advanced Biophotonics Center, Salk Institute for Biological Studies, La Jolla, CA, USA.

Department of Cell & Developmental Biology School of Biological Sciences University of California, San Diego, La Jolla, CA, USA.

Computational Biology & Genomics Core, National Institute on Aging, National Institutes of Health, Baltimore, MD, USA.

Danish Center for Healthy Aging, University of Copenhagen, Copenhagen N, Denmark.

AN - 38500536

AU - Park, J. H.

AU - Sahbaz, B. D.

AU - Pekhale, K.

AU - Chu, X.

AU - Okur, M. N.

AU - Grati, M.

AU - Isgrig, K.

AU - Chien, W.

AU - Chrysostomou, E.

AU - Sullivan, L.

AU - Croteau, D. L.

AU - Manor, U.

AU - Bohr, V. A.

C1 - PubMed

C2 - PMC10948084

C6 - NIHMS1971152 ChromaDex, Inc.

DO - 10.59368/agingbio.20240025

DP - NLM

ET - 2024/03/19

LA - eng

N1 - 2994-2578

Park, Jae-Hyeon

Sahbaz, Burcin Duan

Pekhale, Komal

Chu, Xixia

Okur, Mustafa N

Grati, Mhamed

Isgrig, Kevin

Chien, Wade

Chrysostomou, Elena

Sullivan, Lauren

Croteau, Deborah L

Manor, Uri

Bohr, Vilhelm A

P30 AG068635/AG/NIA NIH HHS/United States

P30 CA014195/CA/NCI NIH HHS/United States

R01 DC021075/DC/NIDCD NIH HHS/United States

ZIA AG000790/ImNIH/Intramural NIH HHS/United States

Journal Article

United States

Aging Biol. 2024;1:20240025. doi: 10.59368/agingbio.20240025. Epub 2024 Feb 20.

PY - 2024

SN - 2994-2578

ST - Early-Onset Hearing Loss in Mouse Models of Alzheimer's Disease and Increased DNA Damage in the Cochlea

T2 - Aging Biol

TI - Early-Onset Hearing Loss in Mouse Models of Alzheimer's Disease and Increased DNA Damage in the Cochlea

VL - 1

ID - 39782

ER -

TY - JOUR

AB - In humans, auditory hair cells are not replaced when injured. Thus, cochlear hair cell loss causes progressive and permanent hearing loss. Conversely, non-mammalian vertebrates are capable of regenerating lost sensory hair cells. The zebrafish lateral line has numerous qualities that make it well-suited for studying hair cell development and regeneration. Histone deacetylase (HDAC) activity has been shown to have an important role in regenerative processes in vertebrates, but its function in hair cell regeneration in vivo is not fully understood. Here, we have examined the role of HDAC activity in hair cell regeneration in the zebrafish lateral line. We eliminated lateral line hair cells of 5-day post-fertilization larvae using neomycin and then treated the larvae with HDAC inhibitors. To assess hair cell regeneration, we used 5-bromo-2-deoxyuridine (BrdU) incorporation in zebrafish larvae to label mitotic cells after hair cell loss. We found that pharmacological inhibition of HDACs using trichostatin A (TSA) or valproic acid (VPA) increased histone acetylation in the regenerated neuromasts following neomycin-induced damage. We also showed that treatment with TSA or VPA decreased the number of supporting cells and regenerated hair cells in response to hair cell damage. Additionally, BrdU immunostaining and western blot analysis showed that TSA or VPA treatment caused a significant decrease in the percentage of S-phase cells and induced p21(Cip1) and p27(Kip1) expression, both of which are likely to explain the decrease in the amount of newly regenerated hair cells in treated embryos. Finally, we showed that HDAC inhibitors induced no observable cell death in neuromasts as measured by cleaved caspase-3 immunohistochemistry and western blot analysis. Taken together, our results demonstrate that HDAC activity has an important role in the regeneration of hair cells in the lateral line.

AD - Department of Otorhinolaryngology, Affiliated Eye and ENT Hospital of Fudan University Shanghai, China.

Department of Otolaryngology Head and Neck Surgery, The First Affiliated Hospital of Xiamen University Xiamen, Fujian, China.

Department of Otorhinolaryngology, Affiliated Eye and ENT Hospital of Fudan University Shanghai, China ; State Key Laboratory of Medical Neurobiology, Fudan University Shanghai, China ; Institute of Stem Cell and Regeneration Medicine, Institutions of Biomedical Science, Fudan University Shanghai, China ; Key Laboratory of Hearing Science, Ministry of Health, EENT Hospital, Fudan University Shanghai, China.

AN - 25431550

AU - He, Y.

AU - Cai, C.

AU - Tang, D.

AU - Sun, S.

AU - Li, H.

C1 - PubMed

C2 - PMC4230041

DO - 10.3389/fncel.2014.00382

DP - NLM

ET - 2014/11/29

KW - hair cell

histone deacetylase inhibitors

regeneration

trichostatin A

valproic acid

zebrafish

commercial or financial relationships that could be construed as a potential

conflict of interest.

LA - eng

N1 - 1662-5102

He, Yingzi

Cai, Chengfu

Tang, Dongmei

Sun, Shan

Li, Huawei

Journal Article

Retracted Publication

Switzerland

Front Cell Neurosci. 2014 Nov 13;8:382. doi: 10.3389/fncel.2014.00382. eCollection 2014.

PY - 2014

SN - 1662-5102 (Print)

1662-5102

SP - 382

ST - Effect of histone deacetylase inhibitors trichostatin A and valproic acid on hair cell regeneration in zebrafish lateral line neuromasts

T2 - Front Cell Neurosci

TI - Effect of histone deacetylase inhibitors trichostatin A and valproic acid on hair cell regeneration in zebrafish lateral line neuromasts

VL - 8

ID - 39225

ER -

TY - JOUR

AB - BACKGROUND: Hair cells are important for maintaining our sense of hearing and balance. However, they are difficult to regenerate in mammals once they are lost. Clarification of the molecular mechanisms underlying inner ear disorders is also impeded by the anatomical limitation of experimental access to the human inner ear. Therefore, the generation of hair cells, possibly from induced pluripotent stem (iPS) cells, is important for regenerative therapy and studies of inner ear diseases. RESULTS: We generated hair cells from mouse iPS cells using an established stepwise induction protocol. First, iPS cells were differentiated into the ectodermal lineage by floating culture. Next, they were treated with basic fibroblast growth factor to induce otic progenitor cells. Finally, the cells were co-cultured with three kinds of mouse utricle tissues: stromal tissue, stromal tissue + sensory epithelium, and the extracellular matrix of stromal tissue. Hair cell-like cells were successfully generated from iPS cells using mouse utricle stromal tissues. However, no hair cell-like cells with hair bundle-like structures were formed using other tissues. CONCLUSIONS: Hair cell-like cells were induced from mouse iPS cells using mouse utricle stromal tissues. Certain soluble factors from mouse utricle stromal cells might be important for induction of hair cells from iPS cells.

AN - 25373336

AU - Taura, A.

AU - Ohnishi, H.

AU - Ochi, S.

AU - Ebisu, F.

AU - Nakagawa, T.

AU - Ito, J.

C1 - PubMed

C2 - PMC4226914

DA - Nov 6

DO - 10.1186/s12868-014-0121-7

DP - NLM

ET - 2014/11/07

KW - Animals

Cell Culture Techniques

Cell Line

Coculture Techniques

Epithelium/physiology

Extracellular Matrix/physiology

Hair Cells, Auditory/*physiology

Immunohistochemistry

Induced Pluripotent Stem Cells/*physiology

Mice

Saccule and Utricle/cytology/*physiology

Stromal Cells/physiology

LA - eng

N1 - 1471-2202

Taura, Akiko

Ohnishi, Hiroe

Ochi, Shohei

Ebisu, Fumi

Nakagawa, Takayuki

Ito, Juichi

Journal Article

Research Support, Non-U.S. Gov't

England

BMC Neurosci. 2014 Nov 6;15:121. doi: 10.1186/s12868-014-0121-7.

PY - 2014

SN - 1471-2202

SP - 121

ST - Effects of mouse utricle stromal tissues on hair cell induction from induced pluripotent stem cells

T2 - BMC Neurosci

TI - Effects of mouse utricle stromal tissues on hair cell induction from induced pluripotent stem cells

VL - 15

ID - 39464

ER -

TY - JOUR

AB - Sensory hair cell losses underlie the vast majority of permanent hearing and balance deficits in humans, but many nonmammalian vertebrates can fully recover from hearing impairments and balance dysfunctions because supporting cells (SCs) in their ears retain lifelong regenerative capacities that depend on proliferation and differentiation as replacement hair cells. Most SCs in vertebrate ears stop dividing during embryogenesis; and soon after birth, vestibular SCs in mammals transition to lasting quiescence as they develop massively thickened circumferential F-actin bands at their E-cadherin-rich adherens junctions. Here, we report that treatment with EGF and a GSK3 inhibitor thinned the circumferential F-actin bands throughout the sensory epithelium of cultured utricles that were isolated from adult mice of either sex. That treatment also caused decreases in E-cadherin, β-catenin, and YAP in the striola, and stimulated robust proliferation of mature, normally quiescent striolar SCs. The findings suggest that E-cadherin-rich junctions, which are not present in the SCs of the fish, amphibians, and birds which readily regenerate hair cells, are responsible in part for the mammalian ear's vulnerability to permanent balance and hearing deficits.SIGNIFICANCE STATEMENT Millions of people are affected by hearing and balance deficits that arise when loud sounds, ototoxic drugs, infections, and aging cause hair cell losses. Such deficits are permanent for humans and other mammals, but nonmammals can recover hearing and balance after supporting cells regenerate replacement hair cells. Mammalian supporting cells lose the capacity to proliferate around the time they develop unique, exceptionally reinforced, E-cadherin-rich intercellular junctions. Here, we report the discovery of a pharmacological treatment that thins F-actin bands, depletes E-cadherin, and stimulates proliferation in long-quiescent supporting cells within a balance epithelium from adult mice. The findings suggest that high E-cadherin in those supporting cell junctions may be responsible, in part, for the permanence of hair cell loss in mammals.

AD - Department of Neuroscience, University of Virginia, School of Medicine, Charlottesville, Virginia 22908, and.

Department of Neuroscience, University of Virginia, School of Medicine, Charlottesville, Virginia 22908, and corwin@virginia.edu.

Department of Cell Biology, University of Virginia, Charlottesville, Virginia 22908.

AN - 32079647

AU - Kozlowski, M. M.

AU - Rudolf, M. A.

AU - Corwin, J. T.

C1 - PubMed

C2 - PMC7096146

DA - Mar 25

DO - 10.1523/jneurosci.2630-19.2020

DP - NLM

ET - 2020/02/23

IS - 13

KW - Actins/metabolism

Animals

Cadherins/genetics/*metabolism

Cell Proliferation/*drug effects

Epidermal Growth Factor/*pharmacology

Glycogen Synthase Kinase 3/*antagonists & inhibitors

Hair Cells, Auditory/*drug effects/metabolism

Mice

Pyridines/pharmacology

Pyrimidines/pharmacology

Saccule and Utricle/*drug effects/metabolism

beta Catenin/metabolism

actin

e-cadherin

hair cell

inner ear

regeneration

vestibular

LA - eng

N1 - 1529-2401

Kozlowski, Mikolaj M

Orcid: 0000-0002-7546-2985

Rudolf, Mark A

Orcid: 0000-0003-4314-5667

Corwin, Jeffrey T

Orcid: 0000-0001-9180-7894

F30 DC016806/DC/NIDCD NIH HHS/United States

R01 DC000200/DC/NIDCD NIH HHS/United States

T32 GM007267/GM/NIGMS NIH HHS/United States

Journal Article

Research Support, N.I.H., Extramural

United States

J Neurosci. 2020 Mar 25;40(13):2618-2632. doi: 10.1523/JNEUROSCI.2630-19.2020. Epub 2020 Feb 20.

PY - 2020

SN - 0270-6474 (Print)

0270-6474

SP - 2618-2632

ST - EGF and a GSK3 Inhibitor Deplete Junctional E-cadherin and Stimulate Proliferation in the Mature Mammalian Ear

T2 - J Neurosci

TI - EGF and a GSK3 Inhibitor Deplete Junctional E-cadherin and Stimulate Proliferation in the Mature Mammalian Ear

VL - 40

ID - 39116

ER -

TY - JOUR

AB - Hearing impairment is a global health problem that affects social communications and the economy. The damage and loss of cochlear hair cells and spiral ganglion neurons (SGNs) as well as the degeneration of neurites of SGNs are the core causes of sensorineural hearing loss. Biotechnologies and biomedical engineering technologies provide new hope for the treatment of auditory diseases, which utilizes biological strategies or tissue engineering methods to achieve drug delivery and the regeneration of cells, tissues, and even organs. Here, the advancements in the applications of biotechnologies (including gene therapy and cochlear organoids) and biomedical engineering technologies (including drug delivery, electrode coating, electrical stimulation and bionic scaffolds) in the field of hearing reconstruction are presented. Moreover, we summarize the challenges and provide a perspective on this field.

AD - State Key Laboratory of Digital Medical Engineering Department of Otolaryngology Head and Neck Surgery Zhongda Hospital School of Life Sciences and Technology Advanced Institute for Life and Health Jiangsu Province High-Tech Key Laboratory for Bio-Medical Research Southeast University Nanjing China.

Co-Innovation Center of Neuroregeneration Nantong University Nantong China.

Department of Neurology The China-Japan Union Hospital of Jilin University Changchun Jilin China.

Department of Otolaryngology Head and Neck Surgery Affiliated Drum Tower Hospital of Nanjing University Medical School Jiangsu Provincial Key Medical Discipline Nanjing China.

The Eighth Affiliated Hospital Sun Yat-Sen University Shenzhen China.

Department of Neurosurgery The First Affiliated Hospital of Shandong First Medical University & Shandong Provincial Qianfoshan Hospital Jinan China.

Medical Science and Technology Innovation Center Shandong First Medical University and Shandong Academy of Medical Sciences Jinan China.

Department of Otolaryngology-Head and Neck Surgery Stanford University School of Medicine Stanford California USA.

Department of Otolaryngology-Head and Neck Surgery The Second Xiangya Hospital Central South University Changsha Hunan Province China.

Department of Otolaryngology Head and Neck Surgery Sichuan Provincial People's Hospital University of Electronic Science and Technology of China Chengdu China.

Institute for Stem Cell and Regeneration Chinese Academy of Sciences Beijing China.

Beijing Key Laboratory of Neural Regeneration and Repair Capital Medical University Beijing China.

AN - 39188297

AU - Hu, Y.

AU - Fang, L.

AU - Zhang, H.

AU - Zheng, S.

AU - Liao, M.

AU - Cui, Q.

AU - Wei, H.

AU - Wu, D.

AU - Cheng, H.

AU - Qi, Y.

AU - Wang, H.

AU - Xin, T.

AU - Wang, T.

AU - Chai, R.

C1 - PubMed

C2 - PMC11235852

DA - Nov

DO - 10.1002/smmd.20230021

DP - NLM

ET - 2024/08/27

IS - 4

KW - drug delivery

electrical stimulation

gene therapy

hearing reconstruction

organoid

LA - eng

N1 - 2751-1871

Hu, Yangnan

Fang, Le

Zhang, Hui

Orcid: 0000-0001-5462-2479

Zheng, Shasha

Liao, Menghui

Cui, Qingyue

Wei, Hao

Wu, Danqi

Cheng, Hong

Qi, Yanru

Wang, Huan

Orcid: 0000-0001-5065-3702

Xin, Tao

Wang, Tian

Chai, Renjie

Orcid: 0000-0002-3885-543x

Journal Article

Review

Germany

Smart Med. 2023 Oct 27;2(4):e20230021. doi: 10.1002/SMMD.20230021. eCollection 2023 Nov.

PY - 2023

SN - 2751-1863 (Print)

2751-1871

SP - e20230021

ST - Emerging biotechnologies and biomedical engineering technologies for hearing reconstruction

T2 - Smart Med

TI - Emerging biotechnologies and biomedical engineering technologies for hearing reconstruction

VL - 2

ID - 38858

ER -

TY - JOUR

AB - Photobiomodulation (PBM) stimulates different types of stem cells to migrate, proliferate, and differentiate in vitro and in vivo. However, little is known about the effects of PBM on the differentiation of embryonic stem cells (ESCs) toward the otic lineage. Only a few reports have documented the in vitro differentiation of ESCs into inner-ear hair cells (HCs) due to the complexity of HCs compared with other target cell types. In this study, we determined the optimal condition to differentiate the ESCs into the otic organoid using different culture techniques and PBM parameters. The efficiency of organoid formation within the embryoid body (EB) was dependent on the cell density of the hanging drop. PBM, using 630 nm wavelength light-emitting diodes (LEDs), further improved the differentiation of inner-ear hair cell-like cells coupled with reactive oxygen species (ROS) overexpression. Transcriptome analysis showed the factors that are responsible for the effect of PBM in the formation of otic organoids, notably, the downregulation of neural development-associated genes and the hairy and enhancer of split 5 (Hes5) gene, which inhibits the differentiation of prosensory cells to hair cells. These data enrich the current differentiation protocols for generating inner-ear hair cells.

AD - Beckman Laser Institute Korea, Dankook University, 119 Dandae-ro, Cheonan 31116, Republic of Korea.

Department of Otolaryngology-Head & Neck Surgery, College of Medicine, Dankook University, 119 Dandae-ro, Cheonan 31116, Republic of Korea.

Department of Nanobiomedical Science and BK21 PLUS NBM Global Research Center for Regenerative Medicine, Dankook University, 119 Dandae-ro, Cheonan 31116, Republic of Korea.

DKU-Theragen Institute for NGS Analysis (DTiNa), 119 Dandae-ro, Cheonan 31116, Republic of Korea.

Institute of Tissue Regeneration Engineering (ITREN), Dankook University, 119 Dandae-ro, Cheonan 31116, Republic of Korea.

Department of Biomedical Science, Dankook University, 119 Dandae-ro, Cheonan 31116, Republic of Korea.

AN - 32258218

AU - Chang, S. Y.

AU - Carpena, N. T.

AU - Mun, S.

AU - Jung, J. Y.

AU - Chung, P. S.

AU - Shim, H.

AU - Han, K.

AU - Ahn, J. C.

AU - Lee, M. Y.

C1 - PubMed

C2 - PMC7118273

DA - Jun 12

DO - 10.1016/j.omtm.2020.03.010

DP - NLM

ET - 2020/04/08

KW - Notch

embryonic stem cell

genomics

inner ear

photobiomodulation

tissue regeneration

LA - eng

N1 - 2329-0501

Chang, So-Young

Carpena, Nathaniel T

Mun, Seyoung

Jung, Jae Yun

Chung, Phil-Sang

Shim, Hosup

Han, Kyudong

Ahn, Jin-Chul

Lee, Min Young

Journal Article

United States

Mol Ther Methods Clin Dev. 2020 Mar 13;17:556-567. doi: 10.1016/j.omtm.2020.03.010. eCollection 2020 Jun 12.

PY - 2020

SN - 2329-0501 (Print)

2329-0501

SP - 556-567

ST - Enhanced Inner-Ear Organoid Formation from Mouse Embryonic Stem Cells by Photobiomodulation

T2 - Mol Ther Methods Clin Dev

TI - Enhanced Inner-Ear Organoid Formation from Mouse Embryonic Stem Cells by Photobiomodulation

VL - 17

ID - 38933

ER -

TY - JOUR

AB - The zebrafish lateral line mechanosensory system shares considerable morphological and molecular similarities with the inner ear. In particular, mechanosensory hair cells are responsible for transducing sensory stimuli in both structures. The epithelia cell adhesion molecule (EpCAM) is expressed in the cells of the inner ear of mammals and in the lateral lines system of fish. EpCAM regulates the many cellular functions including adhesion, migration, proliferation, and differentiation. In this study, we use the epcam (jh79) mutant zebrafish line to determine that EpCAM function is required for proper development and regeneration of posterior lateral line hair cells.

AD - Division of Biology and Biomedical Systems, University of Missouri-Kansas City, Kansas City, Missouri, United States.

Division of Biological and Biomedical Systems, University of Missouri-Kansas City, Kansas City, Missouri, United States.

AN - 38800699

AU - Bufalo, A. J.

AU - Vanderbeck, M. M.

AU - Schmitt, J.

AU - McGraw, H. F.

C1 - PubMed

C2 - PMC11117072

DO - 10.17912/micropub.biology.001219

DP - NLM

ET - 2024/05/27

LA - eng

N1 - 2578-9430

Bufalo, Andrew J

Vanderbeck, Madison M

Schmitt, Jodie

McGraw, Hillary F

R16 GM146690/GM/NIGMS NIH HHS/United States

Journal Article

United States

MicroPubl Biol. 2024 May 8;2024:10.17912/micropub.biology.001219. doi: 10.17912/micropub.biology.001219. eCollection 2024.

PY - 2024

SN - 2578-9430

ST - EpCAM regulates hair cell development and regeneration in the zebrafish lateral line

T2 - MicroPubl Biol

TI - EpCAM regulates hair cell development and regeneration in the zebrafish lateral line

VL - 2024

ID - 39350

ER -

TY - JOUR

AB - The burgeoning field of epigenetics is beginning to make a significant impact on our understanding of tissue development, maintenance, and function. Epigenetic mechanisms regulate the structure and activity of the genome in response to intracellular and environmental cues that direct cell-type specific gene networks. The inner ear is comprised of highly specialized cell types with identical genomes that originate from a single totipotent zygote. During inner ear development specific combinations of transcription factors and epigenetic modifiers must function in a coordinated manner to establish and maintain cellular identity. These epigenetic regulatory mechanisms contribute to the maintenance of distinct chromatin states and cell-type specific gene expression patterns. In this review, we highlight emerging paradigms for epigenetic modifications related to inner ear development, and how epigenetics may have a significant role in hearing loss, protection, and regeneration.

AD - Department of Developmental Neurobiology, St. Jude Children's Research Hospital Memphis, TN, USA.

AN - 25750614

AU - Layman, W. S.

AU - Zuo, J.

C1 - PubMed

C2 - PMC4285911

DO - 10.3389/fncel.2014.00446

DP - NLM

ET - 2014/01/01

KW - DNA methylation

auditory

cellular reprogramming

hair cells

histone acetylation

histone deacetylase inhibitors

histone methylation

ototoxicity

LA - eng

N1 - 1662-5102

Layman, Wanda S

Zuo, Jian

R21 DC013879/DC/NIDCD NIH HHS/United States

Journal Article

Review

Switzerland

Front Cell Neurosci. 2015 Jan 7;8:446. doi: 10.3389/fncel.2014.00446. eCollection 2014.

PY - 2014

SN - 1662-5102 (Print)

1662-5102

SP - 446

ST - Epigenetic regulation in the inner ear and its potential roles in development, protection, and regeneration

T2 - Front Cell Neurosci

TI - Epigenetic regulation in the inner ear and its potential roles in development, protection, and regeneration

VL - 8

ID - 39305

ER -

TY - JOUR

AB - Sensorineural deafness mainly occurs due to damage to hair cells, and advances in stem cell technology, especially the application of induced pluripotent stem cells (iPSCs) and adult stem cells, provides new possibilities for hair cell regeneration. This review describes the basic knowledge of stem cells and their important applications in regenerative medicine, as well as recent progress in stem cell research in the field of hair cell regeneration, especially the induced differentiation of hair-like cells. At the same time, we also point out the challenges facing current research, including differentiation efficiency, cell stability issues, and treatment safety and long-term efficacy considerations. Finally, we look forward to the direction of future research, and emphasize the importance of the cell differentiation mechanism, simulation of the inner ear microenvironment, safety assessment, and personalized treatment strategies. In conclusion, despite many challenges, stem cell technology has shown great potential in the field of hearing research and is expected to bring revolutionary treatment options for patients with sensorineural hearing loss in the future.

AD - Otorhinolaryngology Department, First Affiliated Hospital of Kunming Medical University Kunming, Yunnan, China.

Nephrology Department, First Affiliated Hospital of Kunming Medical University Kunming, Yunnan, China.

AN - 39308766

AU - Li, M.

AU - Ma, R.

AU - Li, Q.

AU - Guo, M.

C1 - PubMed

C2 - PMC11411250

DO - 10.62347/aeiv5813

DP - NLM

ET - 2024/09/23

IS - 4

KW - Sensorineural hearing loss

hair cell regeneration

inner ear microenvironment

regenerative medicine

stem cell technology

LA - eng

N1 - 2160-4150

Li, Min

Ma, Rongyue

Li, Qing

Guo, Min

Journal Article

Review

United States

Am J Stem Cells. 2024 Aug 25;13(4):212-221. doi: 10.62347/AEIV5813. eCollection 2024.

PY - 2024

SN - 2160-4150 (Print)

2160-4150

SP - 212-221

ST - Exploring the application of stem cell technology in treating sensorineural hearing loss

T2 - Am J Stem Cells

TI - Exploring the application of stem cell technology in treating sensorineural hearing loss

VL - 13

ID - 39298

ER -

TY - JOUR

AB - Ferlin proteins participate in such diverse biological events as vesicle fusion in C. elegans, fusion of myoblast membranes to form myotubes, Ca(2+)-sensing during exocytosis in the hair cells of the inner ear, and Ca(2+)-dependent membrane repair in skeletal muscle cells. Ferlins are Ca(2+)-dependent, phospholipid-binding, multi-C2 domain-containing proteins with a single transmembrane helix that spans a vesicle membrane. The overall domain composition of the ferlins resembles the proteins involved in exocytosis; therefore, it is thought that they participate in membrane fusion at some level. But if ferlins do fuse membranes, then they are distinct from other known fusion proteins. Here we show that the central FerA domain from dysferlin, myoferlin, and otoferlin is a novel four-helix bundle fold with its own Ca(2+)-dependent phospholipid-binding activity. Small-angle X-ray scattering (SAXS), spectroscopic, and thermodynamic analysis of the dysferlin, myoferlin, and otoferlin FerA domains, in addition to clinically-defined dysferlin FerA mutations, suggests that the FerA domain interacts with the membrane and that this interaction is enhanced by the presence of Ca(2+).

AD - Department of Cell Physiology and Molecular Biophysics, Texas Tech University Health Sciences Center, Lubbock, TX, 79430-6551, USA.

Department of Biochemistry and Molecular Biology, The University of Texas Medical Branch, Galveston, TX, 77555, USA.

Department of Biophysics, Johns Hopkins University, Baltimore, MD, 21205, USA.

Department of Cell Physiology and Molecular Biophysics, Texas Tech University Health Sciences Center, Lubbock, TX, 79430-6551, USA. roger.b.sutton@ttuhsc.edu.

Center for Membrane Protein Research, Texas Tech University Health Sciences Center, Lubbock, TX, 79430-6551, USA. roger.b.sutton@ttuhsc.edu.

AN - 30026467

AU - Harsini, F. M.

AU - Chebrolu, S.

AU - Fuson, K. L.

AU - White, M. A.

AU - Rice, A. M.

AU - Sutton, R. B.

C1 - PubMed

C2 - PMC6053371

DA - Jul 19

DO - 10.1038/s41598-018-29184-1

DP - NLM

ET - 2018/07/22

IS - 1

KW - Calcium/*metabolism

Calcium-Binding Proteins/*chemistry/genetics/metabolism

Cell Membrane/*metabolism

Circular Dichroism

Dysferlin/*chemistry/genetics/metabolism

Humans

Membrane Fusion

Membrane Proteins/*chemistry/genetics/metabolism

Models, Molecular

Muscle Proteins/*chemistry/genetics/metabolism

Mutation

Protein Domains

Protein Structure, Secondary

Scattering, Small Angle

Thermodynamics

X-Ray Diffraction

LA - eng

N1 - 2045-2322

Harsini, Faraz M

Chebrolu, Sukanya

Fuson, Kerry L

White, Mark A

Orcid: 0000-0003-1057-4203

Rice, Anne M

Sutton, R Bryan

Orcid: 0000-0001-8393-5239

R01 AR063634/AR/NIAMS NIH HHS/United States

Journal Article

Research Support, N.I.H., Extramural

Research Support, Non-U.S. Gov't

England

Sci Rep. 2018 Jul 19;8(1):10949. doi: 10.1038/s41598-018-29184-1.

PY - 2018

SN - 2045-2322

SP - 10949

ST - FerA is a Membrane-Associating Four-Helix Bundle Domain in the Ferlin Family of Membrane-Fusion Proteins

T2 - Sci Rep

TI - FerA is a Membrane-Associating Four-Helix Bundle Domain in the Ferlin Family of Membrane-Fusion Proteins

VL - 8

ID - 39675

ER -

TY - JOUR

AB - Damage to inner hair cells (IHCs) is a leading cause of hearing loss, typically initiating at the base region of the basilar membrane. However, the mechanisms and preventative strategies for IHC damage remain to be elucidated. This study revealed that IHCs in the low-frequency region exhibit a significantly faster calcium clearance rate than high-frequency IHCs. This difference is associated with different PMCA1 expression. We then generated an IHC-specific Pmca1 knockout mouse model (Pmca1 CKO) exhibiting profound hearing loss and IHC death. Using single-cell RNA-seq analysis, we found that the differentially expressed genes (DEGs) were related to tetrahydrofolate biosynthesis, DNA damage, and DNA repair dysfunction. We therefore treated Pmca1 CKO mice with folic acid and found that it protected IHCs by reducing γ-H2A.X levels. In addition, we found that folic acid protected IHCs from noise-induced damage. Overall, our findings suggest that disrupted calcium homeostasis plays a role in IHC damage and that folic acid may be a promising therapeutic agent for protecting hair cells.

AD - Department of Otolaryngology-Head and Neck Surgery, Shanghai Ninth People's Hospital, Shanghai Jiao Tong University School of Medicine, Shanghai, China.

Ear Institute, Shanghai Jiao Tong University School of Medicine, Shanghai, China.

Shanghai Key Laboratory of Translational Medicine on Ear and Nose Diseases, Shanghai, China.

Shanghai Jiao Tong University School of Medicine, Shanghai, China.

Department of Otolaryngology-Head and Neck Surgery, Shanghai Ninth People's Hospital, Shanghai Jiao Tong University School of Medicine, Shanghai, China. wuhao@shsmu.edu.cn.

Ear Institute, Shanghai Jiao Tong University School of Medicine, Shanghai, China. wuhao@shsmu.edu.cn.

Shanghai Key Laboratory of Translational Medicine on Ear and Nose Diseases, Shanghai, China. wuhao@shsmu.edu.cn.

Department of Otolaryngology-Head and Neck Surgery, Shanghai Ninth People's Hospital, Shanghai Jiao Tong University School of Medicine, Shanghai, China. 13761638443@163.com.

Ear Institute, Shanghai Jiao Tong University School of Medicine, Shanghai, China. 13761638443@163.com.

Shanghai Key Laboratory of Translational Medicine on Ear and Nose Diseases, Shanghai, China. 13761638443@163.com.

Department of Otolaryngology-Head and Neck Surgery, Shanghai Ninth People's Hospital, Shanghai Jiao Tong University School of Medicine, Shanghai, China. liuhent9h@163.com.

Ear Institute, Shanghai Jiao Tong University School of Medicine, Shanghai, China. liuhent9h@163.com.

Shanghai Key Laboratory of Translational Medicine on Ear and Nose Diseases, Shanghai, China. liuhent9h@163.com.

AN - 41330911

AU - Cai, R.

AU - Ma, X.

AU - Lu, J.

AU - Liu, H.

AU - Wang, M.

AU - Wang, Z.

AU - Xi, Q.

AU - Wu, H.

AU - Zhang, Z.

AU - Liu, H.

C1 - PubMed

C2 - PMC12811298

DA - Dec 2

DO - 10.1038/s41420-025-02880-4

DP - NLM

ET - 2025/12/03

IS - 1

LA - eng

N1 - 2058-7716

Cai, Ruijie

Ma, Xiaotong

Lu, Jiawen

Liu, Hongchao

Wang, Meijian

Wang, Ziquan

Xi, Qinyan

Wu, Hao

Orcid: 0000-0002-5317-902x

Zhang, Zhihua

Liu, Huihui

Orcid: 0000-0002-2854-2480

82101211/National Natural Science Foundation of China (National Science Foundation of China)/

Journal Article

United States

Cell Death Discov. 2025 Dec 2;12(1):31. doi: 10.1038/s41420-025-02880-4.

PY - 2025

SN - 2058-7716 (Print)

2058-7716

SP - 31

ST - Folic acid prevents inner hair cell degeneration via genomic stability

T2 - Cell Death Discov

TI - Folic acid prevents inner hair cell degeneration via genomic stability

VL - 12

ID - 39672

ER -

TY - JOUR

AB - In the vestibular inner ear, multiple hair-cell organs decompose head movements into angular or linear components with distinct planes of action, time course, frequencies, and amplitudes. The hair cell responses are transmitted to vestibular afferents and propagated to the brain, where the signals contribute to orientation and heading perception and drive reflexes that stabilize vision and posture during movement. Mammalian and other amniote vestibular epithelia feature two hair cell types (I and II) with distinctive afferent synaptic contacts (calyx and bouton) and transmission mechanisms (nonquantal versus quantal), and are organized into central and peripheral zones that generate afferent populations with fundamentally different encoding properties. In altricial rodents like mice, physiological differences between hair cells, afferents and zones emerge prenatally and develop over several postnatal weeks. The mouse utricle is a model system for investigating developmental differentiation of afferent signals by virtue of its highly organized epithelium, accessibility of immature stages, and genetic tractability. Physiological studies demonstrate that selective acquisition of low-voltage-activated potassium channels from the K(V)1 (Kcna) and K(V)7 (Kcnq) families profoundly shapes the maturing sensory signal at multiple stages: hair cell receptor potentials become faster, synaptic transmission from type I hair cells becomes nonquantal, and afferent spike patterns become more irregular. Targeted genetic manipulations coupled with behavioral assessments have revealed transcription factors that regulate the physiological differentiation of hair cell and afferent types. Developmental mechanisms to create new hair cells, hair bundles and functional synapses persist at low levels in mature vestibular epithelia, allowing some regeneration and repair to sustain transduction and transmission for years.

AD - Department of Neurobiology, University of Chicago, Chicago, United States. Electronic address: eatock@uchicago.edu.

AN - 40973231

AU - Eatock, R. A.

C1 - PubMed

DO - 10.1016/bs.ctdb.2025.07.001

DP - NLM

ET - 2025/09/20

KW - Animals

*Hair Cells, Vestibular/cytology/physiology

*Synapses/physiology

*Cell Differentiation/physiology

Humans

Mice

Mammals

Extrastriola

Hair bundle polarity

Hair cell receptor potential

Mechanoelectrical transduction

Nonquantal transmission

Quantal transmission

Ribbon synapses

Striola

Type I hair cells

Type II hair cells

Vestibular afferent neurons

Vestibular efferent neurons

Voltage-gated ion channels

LA - eng

N1 - 1557-8933

Eatock, Ruth Anne

Journal Article

Review

United States

Curr Top Dev Biol. 2025;165:235-306. doi: 10.1016/bs.ctdb.2025.07.001. Epub 2025 Aug 25.

PY - 2025

SN - 0070-2153

SP - 235-306

ST - Functional development and differentiation of mammalian vestibular hair cells and their synapses

T2 - Curr Top Dev Biol

TI - Functional development and differentiation of mammalian vestibular hair cells and their synapses

VL - 165

ID - 38827

ER -

TY - JOUR

AB - DNA damage to cochlear hair cells caused by 8-oxoguanine (8-oxoG) is essential for the development of noise-induced hearing loss (NIHL). Human 8-oxoG DNA glycosylase1 (hOGG1) is a key enzyme in the base excision repair (BER) pathway that eliminates 8-oxoG. Many epidemiological and functional studies have suggested that the hOGG1 Ser326Cys polymorphism (rs1052133) is associated with many diseases. The purpose of this investigation was to investigate whether the hOGG1 Ser326Cys polymorphism in the human BER pathway is associated with genetic susceptibility to NIHL in a Chinese population. This polymorphism was genotyped among 612 workers with NIHL and 615 workers with normal hearing. We found that individuals with the hOGG1 Cys/Cys genotype had a statistically significantly increased risk of NIHL compared with those who carried the hOGG1 Ser/Ser genotype (adjusted OR=1.59, 95% CI=1.13-2.25) and this increased risk was more pronounced among the workers in the 15- to 25- and >25-year noise exposure time, 85-92 dB(A) noise exposure level, ever smoking, and ever drinking groups, similar effects were also observed in a recessive model. In summary, our data suggested that the hOGG1 Cys/Cys genotype may be a genetic susceptibility marker for NIHL in the Chinese Han population.

AD - Kunshan Municipal Center for Disease Prevention and Control, Kunshan, China; Department of Environmental Genomics, Jiangsu Key Laboratory of Cancer Biomarkers, Prevention and Treatment, Cancer Center, Nanjing Medical University, Nanjing, China.

Department of Environmental Genomics, Jiangsu Key Laboratory of Cancer Biomarkers, Prevention and Treatment, Cancer Center, Nanjing Medical University, Nanjing, China; Institute of Occupational Disease Prevention, Jiangsu Provincial Center for Disease Prevention and Control, Nanjing, China.

Kunshan Municipal Center for Disease Prevention and Control, Kunshan, China.

Department of Disease Prevention and Control of Yizheng Hospital, Drum Tower Hospital Group of Nanjing, Yizheng, China.

Institute of Occupational Disease Prevention, Jiangsu Provincial Center for Disease Prevention and Control, Nanjing, China.

The First People's Hospital of Kunshan, Kunshan, China.

Department of Environmental Genomics, Jiangsu Key Laboratory of Cancer Biomarkers, Prevention and Treatment, Cancer Center, Nanjing Medical University, Nanjing, China; Department of Genetic Toxicology, the Key Laboratory of Modern Toxicology of Ministry of Education, School of Public Health, Nanjing Medical University, Nanjing, China.

AN - 24599382

AU - Shen, H.

AU - Cao, J.

AU - Hong, Z.

AU - Liu, K.

AU - Shi, J.

AU - Ding, L.

AU - Zhang, H.

AU - Du, C.

AU - Li, Q.

AU - Zhang, Z.

AU - Zhu, B.

C1 - PubMed

C2 - PMC3943766

DO - 10.1371/journal.pone.0089662

DP - NLM

ET - 2014/03/07

IS - 3

KW - Adult

Amino Acid Substitution

Case-Control Studies

DNA Glycosylases/*genetics

Female

Gene Frequency

Genetic Association Studies

Genetic Predisposition to Disease

Hearing Loss, Noise-Induced/enzymology/*genetics

Humans

Male

Middle Aged

Polymorphism, Single Nucleotide

LA - eng

N1 - 1932-6203

Shen, Huanxi

Cao, Jinglian

Hong, Zhiqiang

Liu, Kai

Shi, Jian

Ding, Lu

Zhang, Hengdong

Du, Cheng

Li, Qian

Zhang, Zhengdong

Zhu, Baoli

Journal Article

Research Support, Non-U.S. Gov't

United States

PLoS One. 2014 Mar 5;9(3):e89662. doi: 10.1371/journal.pone.0089662. eCollection 2014.

PY - 2014

SN - 1932-6203

SP - e89662

ST - A functional Ser326Cys polymorphism in hOGG1 is associated with noise-induced hearing loss in a Chinese population

T2 - PLoS One

TI - A functional Ser326Cys polymorphism in hOGG1 is associated with noise-induced hearing loss in a Chinese population

VL - 9

ID - 39284

ER -

TY - JOUR

AB - Recent advances in cochlear implantology are exemplified by novel functional strategies such as bimodal electroacoustic stimulation, in which the patient has intact low-frequency hearing and profound high-frequency hearing pre-operatively. Therefore, the synergistic restoration of dysfunctional cochlear hair cells and the protection of hair cells from ototoxic insults have become a persistent target pursued for this hybrid system. In this study, we developed a composite GelMA/PEDOT:PSS conductive hydrogel that is suitable as a coating for the cochlear implant electrode for the potential local delivery of otoregenerative and otoprotective drugs. Various material characterization methods (e.g., 1H NMR spectroscopy, FT-IR, EIS, and SEM), experimental models (e.g., murine cochlear organoid and aminoglycoside-induced ototoxic HEI-OC1 cellular model), and biological analyses (e.g., confocal laser scanning microscopy, real time qPCR, flow cytometry, and bioinformatic sequencing) were used. The results demonstrated decent material properties of the hydrogel, such as mechanical (e.g., high tensile stress and Young’s modulus), electrochemical (e.g., low impedance and high conductivity), biocompatibility (e.g., satisfactory cochlear cell interaction and free of systemic toxicity), and biosafety (e.g., minimal hemolysis and cell death) features. In addition, the CDR medicinal cocktail sustainably released by the hydrogel not only promoted the expansion of the cochlear stem cells but also boosted the trans-differentiation from cochlear supporting cells into hair cells. Furthermore, hydrogel-based drug delivery protected the hair cells from oxidative stress and various forms of programmed cell death (e.g., apoptosis and ferroptosis). Finally, using large-scale sequencing, we enriched a complex network of signaling pathways that are potentially downstream to various metabolic processes and abundant metabolites. In conclusion, we present a conductive hydrogel-based local delivery of bifunctional drug cocktails, thereby serving as a potential solution to intracochlear therapy of bimodal auditory rehabilitation and diseases beyond. © 2024 by the authors.

AD - Department of ORL-HNS, Shanghai Fourth People’s Hospital, School of Medicine, Tongji University, Shanghai, 200070, China

Plasma Medicine and Surgical Implants Center, School of Medicine, Tongji University, Shanghai, 200070, China

Department of ORL-HNS, The Royal College of Surgeons in Ireland, Dublin, D02 YN77, Ireland

Department of ORL-HNS, The Royal College of Surgeons of England, London, WC2A 3PE, United Kingdom

State Key Laboratory for Modification of Chemical Fibers and Polymer Materials, College of Chemistry, Chemical Engineering and Biotechnology, Center for Advanced Low-Dimension Materials, Donghua University, Shanghai, 200051, China

AU - Tan, F.

AU - Li, X.

AU - Li, X.

AU - Xu, M.

AU - Shahzad, K. A.

AU - Hou, L.

C1 - Scopus

C2 - 38254695

C7 - 95

DB - Scopus

DO - 10.3390/biom14010095

IS - 1

J2 - Biomolecules

KW - apoptosis

cochlear implant

conductive hydrogel

electroacoustic stimulation

ferroptosis

GelMA

hair cell

hair cell regeneration

PEDOT:PSS

Animals

Cell Communication

Hair Cells, Auditory

Humans

Hydrogels

Mice

Signal Transduction

Spectroscopy, Fourier Transform Infrared

aminoglycoside

glyceraldehyde 3 phosphate dehydrogenase

hydrogel

hydroxylysine

neomycin

photoinitiator

polystyrene

reactive oxygen metabolite

aminoacylation

arachidonic acid metabolism

Article

auditory rehabilitation

biocompatibility

biodegradation

bioinformatics

biosafety

cell death

cell interaction

cell proliferation

cell regeneration

cell transdifferentiation

cell viability

central nervous system

cochlear hair cell

cochlear nerve

conductance

confocal laser scanning microscopy

differential gene expression

electrochemical analysis

electrostimulation

flow cytometry

fluorescence activated cell sorting

gene expression

gene ontology

hair cell (inner ear)

hearing

HEI-OC1 cell line

hemolysis

human

hybrid

immunofluorescence

impedance spectroscopy

infrared spectroscopy

lipid peroxidation

mitochondrial membrane potential

nonhuman

nuclear magnetic resonance spectroscopy

organoid

ototoxicity

oxidative stress

physiological stress

polymerization

proof of concept

proton nuclear magnetic resonance

real time polymerase chain reaction

scanning electron microscopy

stem cell expansion

synergistic effect

upregulation

Young modulus

animal

Fourier transform infrared spectroscopy

mouse

LA - English

M3 - Article

N1 - Export Date: 21 May 2026; Cited By: 22; Correspondence Address: F. Tan; Department of ORL-HNS, Shanghai Fourth People’s Hospital, School of Medicine, Tongji University, Shanghai, 200070, China; email: feitan@tongji.edu.cn

PY - 2024

SN - 2218273X (ISSN)

ST - GelMA/PEDOT:PSS Composite Conductive Hydrogel-Based Generation and Protection of Cochlear Hair Cells through Multiple Signaling Pathways

T2 - Biomolecules

TI - GelMA/PEDOT:PSS Composite Conductive Hydrogel-Based Generation and Protection of Cochlear Hair Cells through Multiple Signaling Pathways

UR - https://www.scopus.com/pages/publications/85183149445?origin=resultslist

VL - 14

ID - 39861

ER -

TY - JOUR

AB - HYPOTHESIS: The present study assessed how to inject a gene into the mouse vestibule and which is the optimum gene to the mouse vestibule adenovirus (AdV) vector or adeno-associated virus (AAV) vector. BACKGROUND: Loss of vestibular hair cell is seen in various balance disorder diseases. There have been some reports concerning gene delivery to the mouse vestibule in recent years. To effectively induce transgene expression at the vestibule, we assessed the efficiency of inoculating the mouse inner ear using various methods. METHODS: We employed an AdV- and AAV-carrying green fluorescent protein using a semicircular canal approach (via a canalostomy) and round window approach. RESULTS: AAV injection via canalostomy induced gene expression at the hair cells, supporting cells, and fibrocytes at the vestibular organs without auditory or balance dysfunction, suggesting it was the most suitable transfection method. This method is thus considered to be a promising strategy to prevent balance dysfunction. CONCLUSION: AAV injection via canalostomy to the vestibule is the noninvasive and highly efficient transfection method, and this study may have the potential to repair balance disorders in human in the future.

AD - Department of Otorhinolaryngology, Juntendo University Faculty of Medicine, Tokyo, Japan.

AN - 22525215

AU - Okada, H.

AU - Iizuka, T.

AU - Mochizuki, H.

AU - Nihira, T.

AU - Kamiya, K.

AU - Inoshita, A.

AU - Kasagi, H.

AU - Kasai, M.

AU - Ikeda, K.

C1 - PubMed

DA - Jun

DO - 10.1097/MAO.0b013e31825368d1

DP - NLM

ET - 2012/04/25

IS - 4

KW - Animals

Dependovirus/*genetics

*Gene Transfer Techniques

Genetic Therapy/methods

*Genetic Vectors/administration & dosage

Hair Cells, Auditory/*cytology/metabolism

Humans

Male

Mice

Mice, Inbred C57BL

*Vestibule, Labyrinth/metabolism/pathology/physiology

LA - eng

N1 - 1537-4505

Okada, Hiroko

Iizuka, Takashi

Mochizuki, Hideki

Nihira, Tomoko

Kamiya, Kazusaku

Inoshita, Ayako

Kasagi, Hiromi

Kasai, Misato

Ikeda, Katsuhisa

Journal Article

United States

Otol Neurotol. 2012 Jun;33(4):655-9. doi: 10.1097/MAO.0b013e31825368d1.

PY - 2012

SN - 1531-7129

SP - 655-9

ST - Gene transfer targeting mouse vestibule using adenovirus and adeno-associated virus vectors

T2 - Otol Neurotol

TI - Gene transfer targeting mouse vestibule using adenovirus and adeno-associated virus vectors

VL - 33

ID - 39639

ER -

TY - JOUR

AB - Most degenerative diseases begin with a gradual loss of specific cell types before reaching a threshold for symptomatic onset. However, the endogenous regenerative capacities of different tissues are difficult to study, because of the limitations of models for early stages of cell loss. Therefore, we generated a transgenic mouse line (Mos-iCsp3) in which a lox-mismatched Cre/lox cassette can be activated to produce a drug-regulated dimerizable caspase-3. Tissue-restricted Cre expression yielded stochastic Casp3 expression, randomly ablating a subset of specific cell types in a defined domain. The limited and mosaic cell loss led to distinct responses in 3 different tissues targeted using respective Cre mice: reversible, impaired glucose tolerance with normoglycemia in pancreatic β cells; wound healing and irreversible hair loss in the skin; and permanent moderate deafness due to the loss of auditory hair cells in the inner ear. These mice will be important for assessing the repair capacities of tissues and the potential effectiveness of new regenerative therapies.

AD - Department of Otology and Laryngology, Harvard Medical School, Boston, Massachusetts, USA.

AN - 21576819

AU - Fujioka, M.

AU - Tokano, H.

AU - Fujioka, K. S.

AU - Okano, H.

AU - Edge, A. S.

C1 - PubMed

C2 - PMC3104751

DA - Jun

DO - 10.1172/jci45081

DP - NLM

ET - 2011/05/18

IS - 6

KW - Alopecia/genetics/pathology

Animals

Apoptosis/genetics

Caspase 3/deficiency/*genetics/physiology

Cell Lineage

Dimerization

*Disease Models, Animal

Epidermis/pathology

Gene Expression Regulation/drug effects

Gene Knockdown Techniques/*methods

*Genes, Transgenic, Suicide

Glucose Intolerance/genetics/pathology

Hair Cells, Auditory, Inner/pathology

Hearing Loss, Bilateral/*genetics

Hearing Loss, Sensorineural/*genetics

Homeodomain Proteins/*genetics

Insulin/deficiency/*genetics

Islets of Langerhans/pathology

Keratin-14/*genetics

Mice

Mice, Inbred C57BL

Mice, Transgenic/*genetics

*Mosaicism

Organ Specificity

Phenotype

Tacrolimus/analogs & derivatives/pharmacology

Transcription Factor Brn-3C/deficiency/*genetics

Transgenes/drug effects

Wound Healing/genetics

LA - eng

N1 - 1558-8238

Fujioka, Masato

Tokano, Hisashi

Fujioka, Keiko Shiina

Okano, Hideyuki

Edge, Albert S B

R01 DC007174/DC/NIDCD NIH HHS/United States

P30 DC05209/DC/NIDCD NIH HHS/United States

R21 DC010440-01/DC/NIDCD NIH HHS/United States

R21 DC010440/DC/NIDCD NIH HHS/United States

P30 DC005209/DC/NIDCD NIH HHS/United States

Journal Article

Research Support, N.I.H., Extramural

Research Support, Non-U.S. Gov't

United States

J Clin Invest. 2011 Jun;121(6):2462-9. doi: 10.1172/JCI45081. Epub 2011 May 16.

PY - 2011

SN - 0021-9738 (Print)

0021-9738

SP - 2462-9

ST - Generating mouse models of degenerative diseases using Cre/lox-mediated in vivo mosaic cell ablation

T2 - J Clin Invest

TI - Generating mouse models of degenerative diseases using Cre/lox-mediated in vivo mosaic cell ablation

VL - 121

ID - 39638

ER -

TY - JOUR

AB - CRISPR/Cas9 system is a promising method for the generation of human disease models by genome editing in non-conventional experimental animals. Medium/large-sized animals like sheep have several advantages to study human diseases and medicine. Here, we present a protocol that describes the generation of an otoferlin edited sheep model via CRISPR-assisted single-stranded oligodinucleotide-mediated Homology-Directed Repair (HDR), through direct cytoplasmic microinjection in in vitro produced zygotes.Otoferlin is a protein expressed in the cochlear inner hair cells, with different mutations at the OTOF gene being the major cause of nonsyndromic recessive auditory neuropathy spectrum disorder in humans. By using this protocol, we reported for the first time an OTOF KI model in sheep with 17.8% edited lambs showing indel mutations, and 61.5% of them bearing knock-in mutations by HDR . The reported method establishes the bases to produce a deafness model to test novel therapies in human disorders related to OTOF mutations.

AD - Laboratory Animal Biotechnology Unit, Institut Pasteur de Montevideo, Montevideo, Uruguay.

INSERM Centre de Recherche en Transplantation et Immunologie UMR 1064, Transgenesis Rat ImmunoPhenomic Facility (TRIP), Nantes, France.

Instituto de Reproducción Animal Uruguay, Fundación IRAUy, Montevideo, Uruguay.

Instituto de Reproducción Animal Uruguay, Fundación IRAUy, Montevideo, Uruguay. menchaca.alejo@gmail.com.

Instituto Nacional de Investigación Agropecuaria (INIA), Montevideo, Uruguay. menchaca.alejo@gmail.com.

AN - 35696036

AU - Crispo, M.

AU - Chenouard, V.

AU - Dos Santos-Neto, P.

AU - Tesson, L.

AU - Souza-Neves, M.

AU - Heslan, J. M.

AU - Cuadro, F.

AU - Anegón, I.

AU - Menchaca, A.

C1 - PubMed

DO - 10.1007/978-1-0716-2301-5_12

DP - NLM

ET - 2022/06/14

KW - Animals

*CRISPR-Cas Systems

*Deafness/genetics

Gene Editing/methods

Humans

Mutation

Recombinational DNA Repair

Sheep

Embryo

Genome editing

In vitro fertilization

Otof

ssODNs

LA - eng

N1 - 1940-6029

Crispo, Martina

Chenouard, Vanessa

Dos Santos-Neto, Pedro

Tesson, Laurent

Souza-Neves, Marcela

Heslan, Jean-Marie

Cuadro, Federico

Anegón, Ignacio

Menchaca, Alejo

Journal Article

Research Support, Non-U.S. Gov't

United States

Methods Mol Biol. 2022;2495:233-244. doi: 10.1007/978-1-0716-2301-5_12.

PY - 2022

SN - 1064-3745

SP - 233-244

ST - Generation of a Human Deafness Sheep Model Using the CRISPR/Cas System

T2 - Methods Mol Biol

TI - Generation of a Human Deafness Sheep Model Using the CRISPR/Cas System

VL - 2495

ID - 39674

ER -

TY - JOUR

AB - Screening for small molecules or drugs that can protect or restore mechanosensory hair cells has been hampered by limited cell numbers. In Nature Biotechnology, Koehler et al. (2017) have developed a human organoid-based approach using basic developmental principles to generate large numbers of bonafide hair cells in vitro.

AD - Laboratory of Cochlear Development, Porter Neuroscience Research Center, National Institute on Deafness and Other Communication Disorders, National Institutes of Health, Bethesda, MD 20892, USA. Electronic address: kelleymt@nidcd.nih.gov.

AN - 28686861

AU - Kelley, M. W.

C1 - PubMed

DA - Jul 6

DO - 10.1016/j.stem.2017.06.007

DP - NLM

ET - 2017/07/08

IS - 1

KW - Hair Cells, Auditory/cytology/*metabolism

Humans

Mechanotransduction, Cellular/*physiology

Wnt Signaling Pathway/*physiology

LA - eng

N1 - 1875-9777

Kelley, Matthew W

Comment

Journal Article

United States

Cell Stem Cell. 2017 Jul 6;21(1):11-12. doi: 10.1016/j.stem.2017.06.007.

PY - 2017

SN - 1875-9777

SP - 11-12

ST - Generation of Human Hair Cells In Vitro: Is It All about How the Wnt Blows?

T2 - Cell Stem Cell

TI - Generation of Human Hair Cells In Vitro: Is It All about How the Wnt Blows?

VL - 21

ID - 39157

ER -

TY - JOUR

AB - Sensory hair cells are exquisitely sensitive vertebrate mechanoreceptors that mediate the senses of hearing and balance. Understanding the factors that regulate the development of these cells is important, not only to increase our understanding of ear development and its functional physiology but also to shed light on how these cells may be replaced therapeutically. In this review, we describe the signals and molecular mechanisms that initiate hair cell development in vertebrates, with particular emphasis on the transcription factor Atoh1, which is both necessary and sufficient for hair cell development. We then discuss recent findings on how microRNAs may modulate the formation and maturation of hair cells. Last, we review recent work on how hair cells are regenerated in many vertebrate groups and the factors that conspire to prevent this regeneration in mammals.

AD - Department of Neuroscience, Baylor College of Medicine, Houston, TX 77030, USA. akgroves@bcm.edu

AN - 23724999

AU - Groves, A. K.

AU - Zhang, K. D.

AU - Fekete, D. M.

C1 - PubMed

C2 - PMC3773239

C6 - NIHMS507004

DA - Jul 8

DO - 10.1146/annurev-neuro-062012-170309

DP - NLM

ET - 2013/06/04

KW - Animals

Basic Helix-Loop-Helix Proteins/*genetics/metabolism

Hair Cells, Auditory/*physiology

Humans

Mechanoreceptors/*physiology

Regeneration/*genetics

LA - eng

N1 - 1545-4126

Groves, Andrew K

Zhang, Kaidi D

Fekete, Donna M

R01 DC006185/DC/NIDCD NIH HHS/United States

Journal Article

Review

United States

Annu Rev Neurosci. 2013 Jul 8;36:361-81. doi: 10.1146/annurev-neuro-062012-170309. Epub 2013 May 29.

PY - 2013

SN - 0147-006X (Print)

0147-006x

SP - 361-81

ST - The genetics of hair cell development and regeneration

T2 - Annu Rev Neurosci

TI - The genetics of hair cell development and regeneration

VL - 36

ID - 38826

ER -

TY - JOUR

AB - Inner ear hair cells are mechanoreceptors critical for hearing and balance. Damage to vestibular hair cells causes balance impairment, yet it is unclear whether hair cell loss correlates with vestibular dysfunction. In this study, we ablated hair cells in Pou4f3 (DTR/+) mice with diphtheria toxin (DT), and found a dose-dependent decrease in hair cell survival in both the macula and crista ampullaris, including loss of type I and II hair cells in the striolar/central and extrastriolar/peripheral regions. Responses to linear acceleration, measured by the translational vestibulo-ocular reflex (tVOR) and vestibular sensory evoked potential (VsEP), were intact with 25% or more hair cell survival in the macula, and diminished only when hair cell survival decreased further. By contrast, rotational vestibulo-ocular reflex (rVOR) responses were significantly reduced with ∼31% hair cell survival in the cristae. Further, single-unit recordings of vestibular afferents from cristae and maculae showed more irregular and reduced firing rates, but only those corresponding to cristae displayed reduced sensitivity to head rotation. Limited hair cell regeneration was observed in the extrastriolar/peripheral regions of both maculae and cristae 6 months post ablation, although no significant recovery of the VORs was observed. Thus, the adult mouse vestibular end organs display different degrees of redundancy, demonstrating robust responses to head rotation and linear acceleration despite loss of most hair cells.

AN - 41383774

AU - Wang, T.

AU - Hosseini, D. K.

AU - Mahhoudi, A.

AU - Sayyid, Z. N.

AU - He, J.

AU - Sit, C.

AU - Iriarte Oporto, Z. G.

AU - Zhu, H.

AU - Zhou, W.

AU - Cheng, A. G.

C1 - PubMed

C2 - PMC12694599

DA - Dec 2

DO - 10.1101/2025.11.27.691044

DP - NLM

ET - 2025/12/12

LA - eng

N1 - 2692-8205

Wang, Tian

Hosseini, Davood K

Mahhoudi, Ahmad

Sayyid, Zahra N

He, Jun

Sit, Caroline

Iriarte Oporto, Zelma G

Zhu, Hong

Zhou, Wu

Cheng, Alan G

Orcid: 0000-0003-3193-3818

Journal Article

Preprint

United States

bioRxiv [Preprint]. 2025 Dec 2:2025.11.27.691044. doi: 10.1101/2025.11.27.691044.

PY - 2025

SN - 2692-8205

ST - Graded Hair Cell Ablation Reveals Functional Redundancy in the Mature Mouse Vestibular System

T2 - bioRxiv

TI - Graded Hair Cell Ablation Reveals Functional Redundancy in the Mature Mouse Vestibular System

ID - 39137

ER -

TY - JOUR

AB - In the inner ear, cyclic guanosine monophosphate (cGMP) signaling has been described as facilitating otoprotection, which was previously observed through elevated cGMP levels achieved by phosphodiesterase 5 inhibition. However, to date, the upstream guanylyl cyclase (GC) subtype eliciting cGMP production is unknown. Here, we show that mice with a genetic disruption of the gene encoding the cGMP generator GC-A, the receptor for atrial and B-type natriuretic peptides, display a greater vulnerability of hair cells to hidden hearing loss and noise- and age-dependent hearing loss. This vulnerability was associated with GC-A expression in spiral ganglia and outer hair cells (OHCs) but not in inner hair cells (IHCs). GC-A knockout mice exhibited elevated hearing thresholds, most pronounced for the detection of high-frequency tones. Deficits in OHC input-output functions in high-frequency regions were already present in young GC-A-deficient mice, with no signs of an accelerated progression of age-related hearing loss or higher vulnerability to acoustic trauma. OHCs in these frequency regions in young GC-A knockout mice exhibited diminished levels of KCNQ4 expression, which is the dominant K(+) channel in OHCs, and decreased activation of poly (ADP-ribose) polymerase-1, an enzyme involved in DNA repair. Further, GC-A knockout mice had IHC synapse impairments and reduced amplitudes of auditory brainstem responses that progressed with age and with acoustic trauma, in contrast to OHCs, when compared to GC-A wild-type littermates. We conclude that GC-A/cGMP-dependent signaling pathways have otoprotective functions and GC-A gene disruption differentially contributes to hair-cell damage in a healthy, aged, or injured system. Thus, augmentation of natriuretic peptide GC-A signaling likely has potential to overcome hidden and noise-induced hearing loss, as well as presbycusis.

AD - Molecular Physiology of Hearing, Tübingen Hearing Research Centre, Department of Otolaryngology, University of Tübingen, Tübingen, Germany.

Department of Anatomy and Cell Biology, Schulich School of Medicine and Dentistry, University of Western Ontario, London, ON, Canada.

Cell Death Mechanisms Group, Institute for Ophthalmic Research, Centre for Ophthalmology, University of Tübingen, Tübingen, Germany.

Department of Biophysics, Center for Integrative Physiology and Molecular Medicine, Hearing Research, Saarland University, Homburg, Germany.

Signal Transduction and Transgenic Models, Interfaculty Institute of Biochemistry, University of Tübingen, Tübingen, Germany.

Institute of Physiology, University of Würzburg, Würzburg, Germany.

AN - 32327991

AU - Marchetta, P.

AU - Möhrle, D.

AU - Eckert, P.

AU - Reimann, K.

AU - Wolter, S.

AU - Tolone, A.

AU - Lang, I.

AU - Wolters, M.

AU - Feil, R.

AU - Engel, J.

AU - Paquet-Durand, F.

AU - Kuhn, M.

AU - Knipper, M.

AU - Rüttiger, L.

C1 - PubMed

C2 - PMC7160671

DO - 10.3389/fnagi.2020.00083

DP - NLM

ET - 2020/04/25

KW - Kcnq4

Parp-1

aging

cGMP

guanylyl cyclase A

hidden hearing loss

inner ear

otoprotection

LA - eng

N1 - 1663-4365

Marchetta, Philine

Möhrle, Dorit

Eckert, Philipp

Reimann, Katrin

Wolter, Steffen

Tolone, Arianna

Lang, Isabelle

Wolters, Markus

Feil, Robert

Engel, Jutta

Paquet-Durand, François

Kuhn, Michaela

Knipper, Marlies

Rüttiger, Lukas

Journal Article

Switzerland

Front Aging Neurosci. 2020 Apr 9;12:83. doi: 10.3389/fnagi.2020.00083. eCollection 2020.

PY - 2020

SN - 1663-4365 (Print)

1663-4365

SP - 83

ST - Guanylyl Cyclase A/cGMP Signaling Slows Hidden, Age- and Acoustic Trauma-Induced Hearing Loss

T2 - Front Aging Neurosci

TI - Guanylyl Cyclase A/cGMP Signaling Slows Hidden, Age- and Acoustic Trauma-Induced Hearing Loss

VL - 12

ID - 39833

ER -

TY - JOUR

AB - Cochlear hair cells are mechanoreceptors of the auditory system and cannot spontaneously regenerate in adult mammals; thus hearing loss due to hair cell damage is permanent. In contrast, hair cells in nonmammalian vertebrates such as birds and in the zebrafish lateral line have the ability to regenerate after hair cell loss. Many regulatory factors, including signaling pathways, transcription factors, and epigenetic regulators, play roles in hair cell regeneration in various species. In this chapter, we review the history of hair cell regeneration research, the methods used in the study of hair cell regeneration, the properties and modulating factors of inner ear stem cells, and the re-formation of cochlear ribbon synapses and hearing function recovery.

AD - ENT Institute and Otorhinolaryngology Department, Affiliated Eye and ENT Hospital, Fudan University, Shanghai, China.

NHC Key Laboratory of Hearing Medicine (Fudan University), Shanghai Engineering Research Centre of Cochlear Implant, Shanghai, China.

Key Laboratory for Developmental Genes and Human Disease, Ministry of Education, Jiangsu Province High-Tech Key Laboratory for Bio-Medical Research, Institute of Life Sciences, Southeast University, Nanjing, China.

MOE Key Laboratory for Developmental Genes and Human Disease, Institute of Life Sciences, Jiangsu Province High-Tech Key Laboratory for Bio-Medical Research, Southeast University, Nanjing, China.

Key Laboratory of Hearing Medicine of NHFPC, ENT Institute and Otorhinolaryngology Department, Shanghai Engineering Research Centre of Cochlear Implant, Affiliated Eye and ENT Hospital, State Key Laboratory of Medical Neurobiology, Fudan University, Shanghai, China.

Key Laboratory of Hearing Medicine of NHFPC, ENT Institute and Otorhinolaryngology Department, Shanghai Engineering Research Centre of Cochlear Implant, Affiliated Eye and ENT Hospital, State Key Laboratory of Medical Neurobiology, Fudan University, Shanghai, China. hwli@shmu.edu.cn.

AN - 30915698

AU - Chen, Y.

AU - Zhang, S.

AU - Chai, R.

AU - Li, H.

C1 - PubMed

DO - 10.1007/978-981-13-6123-4_1

DP - NLM

ET - 2019/03/28

KW - Animals

Birds

*Ear, Inner

Hair Cells, Auditory/*cytology

*Regeneration

Stem Cells/cytology

Zebrafish

Auditory system

Cochlea

Epigenetic regulation

Hair cell regeneration

Signaling pathways

Transcription factors

LA - eng

N1 - Chen, Yan

Zhang, Shasha

Chai, Renjie

Li, Huawei

Journal Article

Review

United States

Adv Exp Med Biol. 2019;1130:1-16. doi: 10.1007/978-981-13-6123-4_1.

PY - 2019

SN - 0065-2598 (Print)

0065-2598

SP - 1-16

ST - Hair Cell Regeneration

T2 - Adv Exp Med Biol

TI - Hair Cell Regeneration

VL - 1130

ID - 38768

ER -

TY - JOUR

AB - Cochlear hair cells (HCs) are the mechanoreceptors of the auditory system, and because these cells cannot be spontaneously regenerated in adult mammals, hearing loss due to HC damage is permanent. However, cochleae of neonatal mice harbor some progenitor cells that retain limited ability to give rise to new HCs in vivo. Here we review the regulatory factors, signaling pathways, and epigenetic factors that have been reported to play roles in HC regeneration in the neonatal mammalian cochlea.

AD - Key Laboratory for Developmental Genes and Human Disease, Ministry of Education, Institute of Life Sciences, Southeast University Nanjing 210096, China.

Department of Otolaryngology Head and Neck Surgery, Affiliated Drum Tower Hospital of Nanjing University Medical School, Jiangsu Provincial Key Medical Discipline (Laboratory) Nanjing 210008, China.

Co-Innovation Center of Neuroregeneration, Nantong University Nantong 226001, China.

Institute for Stem Cell and Regeneration, Chinese Academy of Science Beijing, China.

Jiangsu Province High-Tech Key Laboratory for Bio-Medical Research, Southeast University Nanjing 211189, China.

AN - 32699655

AU - Zhang, S.

AU - Qiang, R.

AU - Dong, Y.

AU - Zhang, Y.

AU - Chen, Y.

AU - Zhou, H.

AU - Gao, X.

AU - Chai, R.

C1 - PubMed

C2 - PMC7364385

DP - NLM

ET - 2020/07/24

IS - 3

KW - Cochlea

hair cell regeneration

inner ear progenitor

signaling pathway

transcription factor

LA - eng

N1 - 2160-4150

Zhang, Shasha

Qiang, Ruiying

Dong, Ying

Zhang, Yuan

Chen, Yin

Zhou, Han

Gao, Xia

Chai, Renjie

Journal Article

Review

United States

Am J Stem Cells. 2020 Jun 15;9(3):25-35. eCollection 2020.

PY - 2020

SN - 2160-4150 (Print)

2160-4150

SP - 25-35

ST - Hair cell regeneration from inner ear progenitors in the mammalian cochlea

T2 - Am J Stem Cells

TI - Hair cell regeneration from inner ear progenitors in the mammalian cochlea

VL - 9

ID - 38833

ER -

TY - JOUR

AB - Noise-induced hearing loss (NIHL) is a major public health problem caused by damage to cochlear hair cells, synapses, and spiral ganglion neurons (SGNs). Since effective treatments are lacking, we investigated cellular stress responses induced by moderate and loud noise in a mouse model of cochlear synaptopathy. RNA sequencing and spatial transcriptomics revealed that noise exposure elicited a robust but transient upregulation of endoplasmic reticulum chaperones and proteasome subunits in SGNs and their supporting cells. To target this response, we administered TRC051384, a small-molecule activator of the heat shock transcription factor Hsf1, prior to noise exposure. TRC051384 crossed the blood-labyrinth barrier and reached the cochlea, induced heat shock protein gene expression, and restored ubiquitin-proteasome function in SGNs. Notably, TRC051384 treatment enhanced auditory brainstem response threshold recovery, preserved Wave I amplitudes, and maintained ribbon synapse density. These findings establish proteotoxic stress in SGNs as a key driver of NIHL and identify HSF1 activation as a promising therapeutic strategy.

AD - Department of Neurology, Northwestern University Feinberg School of Medicine, Chicago, Illinois, USA.

Present address: Department of Otorhinolaryngology, Union Hospital, Tongji Medical College, Huazhong University of Science and Technology, Wuhan 430022, Hubei, China.

Department of Psychiatry and Behavioral Sciences, Northwestern University Feinberg School of Medicine, Chicago, Illinois, USA.

Department of Preventive Medicine, Northwestern University Feinberg School of Medicine, Chicago, Illinois, USA.

Department of Communication Sciences and Disorders, Northwestern University, Evanston, Illinois, USA.

The Hugh Knowles Center for Clinical and Basic Science in Hearing and Its Disorders, Northwestern University, Evanston, Illinois, USA.

Department of Otolaryngology, Washington University in St. Louis School of Medicine, St. Louis, MO, USA.

AN - 41415422

AU - Yu, J.

AU - Ramirez, M. A.

AU - Wang, Y. Z.

AU - Edassery, S.

AU - Shramuk, M.

AU - Cheatham, M. A.

AU - Rutherford, M. A.

AU - Welty, L. J.

AU - Savas, J. N.

C1 - PubMed

C2 - PMC12709475 exists.

DA - Dec 11

DO - 10.64898/2025.12.08.693048

DP - NLM

ET - 2025/12/19

LA - eng

N1 - 2692-8205

Yu, Jintao

Ramirez, Miguel A

Wang, Yi-Zhi

Edassery, Seby

Shramuk, Maxwell

Cheatham, Mary Ann

Rutherford, Mark A

Welty, Leah J

Savas, Jeffrey N

R01 DC014712/DC/NIDCD NIH HHS/United States

Journal Article

Preprint

United States

bioRxiv [Preprint]. 2025 Dec 11:2025.12.08.693048. doi: 10.64898/2025.12.08.693048.

PY - 2025

SN - 2692-8205

ST - Heat-shock pathway activation by TRC051384 protects spiral ganglion neurons from noise-induced hearing loss

T2 - bioRxiv

TI - Heat-shock pathway activation by TRC051384 protects spiral ganglion neurons from noise-induced hearing loss

ID - 39202

ER -

TY - JOUR

AB - The histamine H3 receptor (H3R) is a crucial regulator of synaptic plasticity, neurotransmitter release, and neural signaling within the central nervous system. However, its role in the cochlea remains poorly understood, even though mast cells, a rich endogenous source of histamine, have recently been documented in the mammalian cochlea. This study examined H3R expression and localization in the postnatal day 4-5 (P4-5) C57BL/6 mouse cochlea and evaluated its functional consequences under antagonist treatment. RT-qPCR analysis showed significantly higher H3R mRNA levels in the modiolus compared to the organ of Corti and the lateral wall. Immunofluorescence staining confirmed H3R localization in hair cells (HCs) and spiral ganglion neurons (SGNs). Dissected cochlear explants exposed to two distinct H3R antagonists-ciproxifan and pitolisant-at concentrations of 10µM, 50µM, and 100µM, displayed different responses: ciproxifan induced dose-dependent HC loss. In contrast, pitolisant caused no loss of HC but led to stereociliary abnormalities at higher concentrations. Both antagonists disrupted type II SGN neurite projections, redirecting their normal basal-directed trajectory toward the apical region. These findings implicate H3R in maintaining cochlear structural integrity and guiding SGN neurite development during early postnatal maturation. Further investigation into H3R-mediated mechanisms may reveal new therapeutic targets for hearing preservation and repair.

AD - Department of Otorhinolaryngology, Head and Neck Surgery, Charité- Universitätsmedizin Berlin, Corporate Member of Freie Universität Berlin and Humboldt-Universität zu Berlin, 10117, Berlin, Germany.

Department of Otorhinolaryngology, Head and Neck Surgery, Charité- Universitätsmedizin Berlin, Corporate Member of Freie Universität Berlin and Humboldt-Universität zu Berlin, 10117, Berlin, Germany. agnes.szczepek@charite.de.

Faculty of Medicine and Health Sciences, University of Zielona Góra, Zielona Góra, 65-046, Poland. agnes.szczepek@charite.de.

AN - 40801998

AU - Kong, L.

AU - Olze, H.

AU - Szczepek, A. J.

C1 - PubMed

C2 - PMC12350512

DA - Aug 13

DO - 10.1007/s11064-025-04521-9

DP - NLM

ET - 2025/08/13

IS - 4

KW - Animals

*Spiral Ganglion/drug effects/growth & development/metabolism

Mice, Inbred C57BL

*Cochlea/drug effects/growth & development/metabolism

*Neurites/drug effects/metabolism

*Histamine H3 Antagonists/pharmacology

*Receptors, Histamine H3/metabolism

Mice

Imidazoles

Antagonists

Cochlea

Hair cells

Histamine H3 receptor

Spiral ganglion neurons

LA - eng

N1 - 1573-6903

Kong, Lingyi

Orcid: 0000-0002-5398-0327

Olze, Heidi

Orcid: 0000-0001-9987-6392

Szczepek, Agnieszka J

Orcid: 0000-0002-9292-6606

Journal Article

United States

Neurochem Res. 2025 Aug 13;50(4):266. doi: 10.1007/s11064-025-04521-9.

PY - 2025

SN - 0364-3190 (Print)

0364-3190

SP - 266

ST - Histamine H3 Receptor Antagonists Influence the Directional Growth of Type II Spiral Ganglion Neurites Within the Developing Cochlea of C57BL/6 Mice

T2 - Neurochem Res

TI - Histamine H3 Receptor Antagonists Influence the Directional Growth of Type II Spiral Ganglion Neurites Within the Developing Cochlea of C57BL/6 Mice

VL - 50

ID - 39557

ER -

TY - JOUR

AB - High altitude is characterized by low oxygen, low pressure, and high radiation. When migrates from low to high altitudes, the body's tissues and organs experience hypoxic stress and will present acoustic adaptation as the protective response. However, the mechanisms of acoustic adaptation at high altitudes remain unclear. In this study, cochlear tissues from Wistar rats were collected at 15, 30, 60, 120, and 180 days after high-altitude migration. Transcriptome sequencing was conducted and DESeq algorithm revealed expression patterns of Differentially Expressed Genes(DEGs) after high altitude migration. Day 60 is a critical stage for cochlear tissue "damage" and "repair" in high-altitude conditions. Transmission Electron Microscopy (TEM) observations of structures also support the findings. A time-series gene co-expression network algorithm was used to investigate gene regulatory patterns and key genes after migration. Immunofluorescence, immunohistochemistry, and qPCR were per-formed for key gene validation and localization. At Day 60, the peak DEG count occurs in rats migrating to high altitude, aligning with the critical phase for cochlear tissue damage and repair at high altitudes. Repair hinges on synaptic plasticity and myelination-linked processes, influencing modules M4 to M6. Module M4's activation gradually diminishes from its peak. However, the 'damage' effect is orchestrated by inflammation-related processes in modules M3 to M5, with module M3's activation also waning. Key gene module M4, pivotal for repair during this pivotal phase, encompasses Sptbn5, Cldn1, Gfra2, and Lims2 as its core genes. Immunohistochemistry reveals Sptbn5's presence in cochlear neurons, hair cells, Schwann cells and stria vascularis tissue. Cldn1 and Gfra2 predominantly localize within the cochlear neuron region. These results may suggest new directions for future research on acoustic acclimatization to high altitude.

AD - Department of Regenerative Medicine, College of Pharmacy, Jilin University, Changchun, Jilin, 130021, China.

Laboratory of Cancer Precision Medicine, The First Hospital of Jilin University, Changchun, Jilin, 130021, China.

Tibet University Medical College, Lhasa, Tibet, 850000, China.

Department of Otolaryngology-Head and Neck Surgery, The First Hospital of Jilin University, Changchun, Jilin, 130021, China.

Department of Neurosurgery, The Affiliated Ganzhou Hospital of Nanchang University, Ganzhou, Jiangxi, 341000, China.

Department of Regenerative Medicine, College of Pharmacy, Jilin University, Changchun, Jilin, 130021, China. suxy@jlu.edu.cn.

Department of Otolaryngology-Head and Neck Surgery, The First Hospital of Jilin University, Changchun, Jilin, 130021, China. wang_ping@jlu.edu.cn.

AN - 39505982

AU - Wang, L.

AU - Yi, X.

AU - Zhou, Y.

AU - Gongga, L.

AU - Yu, S.

AU - Guo, X.

AU - Pan, X.

AU - Su, X.

AU - Wang, P.

C1 - PubMed

C2 - PMC11541580

DA - Nov 6

DO - 10.1038/s41598-024-78169-w

DP - NLM

ET - 2024/11/07

IS - 1

KW - Animals

Rats

*Altitude

*Rats, Wistar

*Hypoxia/metabolism/genetics

*Gene Expression Profiling

Transcriptome

Cochlea/metabolism

Male

Adaptation, Physiological/genetics

Gene Regulatory Networks

Acclimatization

Acclimatation

BFS algorithm

Hearing loss

High altitudes hypoxia

TO-GCN analysis

LA - eng

N1 - 2045-2322

Wang, Luoying

Yi, Xingcheng

Zhou, Yulai

Gongga, Lanzi

Yu, Shuyuan

Guo, Xinyi

Pan, Xiaoqiang

Su, Xiaoyun

Wang, Ping

No. 20220204037YY/Jilin Provincial Science&amp; Technology Development Program/

No.20200201493JC/Jilin Provincial Science&amp; Technology Development Program/

No. 81860567/National Natural Science Foundation of China/

Journal Article

England

Sci Rep. 2024 Nov 6;14(1):26909. doi: 10.1038/s41598-024-78169-w.

PY - 2024

SN - 2045-2322

SP - 26909

ST - Hypoxia adaptation mechanism in rats' peripheral auditory system in high altitude migration: a time series transcriptome analysis

T2 - Sci Rep

TI - Hypoxia adaptation mechanism in rats' peripheral auditory system in high altitude migration: a time series transcriptome analysis

VL - 14

ID - 39591

ER -

TY - JOUR

AB - Noise exposure is a health hazard in the textile industry. In cochlear hair cells, DNA damage caused by 8-oxoguanine (8-oxo G) can result in noise-induced hearing loss. Human 8-hydroxyguanine glycosylase (hOGG1) is a DNA repair enzyme that excises (8-oxo G) in the DNA and repairs DNA damage. Glutathione peroxidase-1 (GPx) is a crucial antioxidant enzyme that aids in limiting cochlear damages. Heme oxygenase-1 (HO-1) is a stress-inducible protein with a high fold change in the hair cells of the cochlea. The study aimed to investigate the association of either hOGG1 and GPx-1 polymorphisms with audiometric notches and HO-1 protein among textile workers. hOGG1 and GPx genotypes were analyzed by PCR-RFLP, and HO-1 levels were measured by ELISA in 115 male textile workers. Blood pressure and audiogram were performed. Results recorded the relation between audiometric notches and ear complaints among workers. Older age workers showed audiometric notches at > 25 dB with a significant decrease in HO-1 levels and higher levels in workers with normal audiogram. Ser/Cys genotype of hOGG1 gene was associated with age and work duration while CC genotype of GPx is associated with HO-1 levels and diastolic pressure. Ser/Cys genotype of hOGG1 gene was associated with age while Cys/Cys genotype was associated with work duration among workers. CC genotype of GPx gene was associated with higher HO-1 levels and TT genotype was associated with high diastolic pressure. Finally, hearing impairment was dependent on the duration of exposure to noise, older age, and the presence of heterozygote TC genotype of GPx gene among textile workers.

AD - Department of Environmental and Occupational Medicine, Environment and Climate Change Research Institute, National Research Centre, Giza, Egypt. monataha45@yahoo.com.

Department of Environmental and Occupational Medicine, Environment and Climate Change Research Institute, National Research Centre, Giza, Egypt.

AN - 38147255

AU - Taha, M. M.

AU - Samir Ellaithy, L.

AU - Abd El-Aziz, N. S.

AU - Mahdy-Abdallah, H.

AU - Adel Helmy, M.

C1 - PubMed

C2 - PMC10798917

DA - Jan

DO - 10.1007/s11356-023-31590-6

DP - NLM

ET - 2023/12/26

IS - 4

KW - Humans

Male

Case-Control Studies

Genotype

*Hearing

*Heme Oxygenase-1/genetics

Polymorphism, Genetic

Textiles

*Noise, Occupational

*Occupational Exposure

*DNA Glycosylases/genetics

*Glutathione Peroxidase GPX1/genetics

Audiometric notches

GPx genes

HO-1 protein

Hearing impairment

Noise

hOGG1

LA - eng

N1 - 1614-7499

Taha, Mona Mohamed

Orcid: 0000-0002-2546-4763

Samir Ellaithy, Lamia

Abd El-Aziz, Nermeeen Said

Mahdy-Abdallah, Heba

Adel Helmy, Mona

11010180./National research centre/

Journal Article

Germany

Environ Sci Pollut Res Int. 2024 Jan;31(4):6176-6185. doi: 10.1007/s11356-023-31590-6. Epub 2023 Dec 26.

PY - 2024

SN - 0944-1344 (Print)

0944-1344

SP - 6176-6185

ST - Implication of noise exposure on hearing with emphasis to hOGG1 and GPx-1 polymorphisms and HO-1 protein among textile workers

T2 - Environ Sci Pollut Res Int

TI - Implication of noise exposure on hearing with emphasis to hOGG1 and GPx-1 polymorphisms and HO-1 protein among textile workers

VL - 31

ID - 39342

ER -

TY - JOUR

AB - The sensory "hair" cells of the inner ear transform sound energy into electrical signals, but are readily lost through aging, excessive noise, and ototoxic agents. The newt provides an excellent model in which to explore regeneration and whilst loss of hair cells from inner ear epithelia does not require whole organ regeneration, new hair cells are generated from differentiated supporting cells that transdifferentiate without an intervening mitotic event. Here we describe the methods for maintaining the sensory epithelia in long term culture; for the use of the aminoglycoside, gentamicin, to kill the hair cells; and for the examination of the tissue by electron microscopy or fluorescence microscopy. Demembranation of the epithelium reveals the underlying ultrastructure of the tissue for examination by scanning electron microscopy (SEM) and is a technique that can be utilized with immunogold labelling.

AD - UCL Ear Institute, University College London, 332 Gray's Inn Road, London, WC1X 8EE, UK, Ruth.r.taylor@ucl.ac.uk.

AN - 25740492

AU - Taylor, R. R.

C1 - PubMed

DO - 10.1007/978-1-4939-2495-0_20

DP - NLM

ET - 2015/03/06

KW - Animals

Cell Culture Techniques/*methods

Epithelium/metabolism

Fluorescent Antibody Technique

Gold/metabolism

Hair Cells, Auditory/*cytology/ultrastructure

Microscopy, Electron, Scanning

Microscopy, Electron, Transmission

*Regeneration

Salamandridae/*physiology

Staining and Labeling

Time Factors

LA - eng

N1 - 1940-6029

Taylor, Ruth R

Journal Article

Research Support, Non-U.S. Gov't

United States

Methods Mol Biol. 2015;1290:253-65. doi: 10.1007/978-1-4939-2495-0_20.

PY - 2015

SN - 1064-3745

SP - 253-65

ST - In vitro preparation of newt inner ear sensory epithelia as a model for repair and regeneration

T2 - Methods Mol Biol

TI - In vitro preparation of newt inner ear sensory epithelia as a model for repair and regeneration

VL - 1290

ID - 39158

ER -

TY - JOUR

AB - While mesenchymal stem cells (MSCs) are promising candidates for inner ear hair cell regeneration, to date, there have been no convincing reports indicating whether MSCs can survive in the cochlea for more than a few weeks, as the high levels of potassium (K(+)) in the endolymph (EL) are thought to be toxic to transplanted stem cells. For conditioning the EL for MSC transplantation, we conducted this in vitro study to examine the effects of artificial EL with altered K(+) concentration levels, in the range of 5-153.8 mM, on proliferation, apoptosis, and morphological changes in MSCs derived from various human tissues. Our findings demonstrate that altering the K(+) concentration in artificial EL could significantly influence the survival of MSCs in vitro. We discovered that K(+) concentrations of 55-130 mM in artificial EL could enhance the survival of MSCs in vitro. However, MSCs exhibited reduced proliferation regardless of K(+) concentration.

AD - 1 Department of Otolaryngology, Peking Union Medical College Hospital , Chinese Academy of Medical Sciences and Peking Union Medical College, Beijing, People's Republic of China .

2 Department of Otolaryngology Research, Translational Medicine Center , Peking Union Medical College Hospital, Chinese Academy of Medical Sciences and Peking Union Medical College, Beijing, People's Republic of China .

3 Department of Cell Biology, Center of Excellence in Tissue Engineering, Key Laboratory of Beijing, Institute of Basic Medical Sciences and School of Basic Medicine , Chinese Academy of Medical Sciences and Peking Union Medical College, Beijing, People's Republic of China .

AN - 29631482

AU - Gao, J.

AU - Wang, S.

AU - Tang, Q.

AU - Li, X.

AU - Zhang, Y.

AU - Liu, W.

AU - Gao, Z.

AU - Yang, H.

AU - Zhao, R. C.

C1 - PubMed

DA - May 15

DO - 10.1089/scd.2018.0016

DP - NLM

ET - 2018/04/11

IS - 10

KW - Apoptosis/drug effects

Cell Differentiation/drug effects

Cell Proliferation/drug effects

Cells, Cultured

Endolymph/*cytology/*drug effects

Humans

Mesenchymal Stem Cell Transplantation/methods

Mesenchymal Stem Cells/*drug effects

Potassium/*pharmacology

endolymph

mesenchymal stem cells

potassium

stem cell therapy

survival

LA - eng

N1 - 1557-8534

Gao, Juanjuan

Wang, Shihua

Tang, Qi

Li, Xiaoxia

Zhang, Yongli

Liu, Wenbin

Gao, Zhiqiang

Yang, Hua

Zhao, Robert Chunhua

Journal Article

Research Support, Non-U.S. Gov't

United States

Stem Cells Dev. 2018 May 15;27(10):658-670. doi: 10.1089/scd.2018.0016. Epub 2018 May 7.

PY - 2018

SN - 1547-3287

SP - 658-670

ST - In Vitro Survival of Human Mesenchymal Stem Cells is Enhanced in Artificial Endolymph with Moderately High Concentrations of Potassium

T2 - Stem Cells Dev

TI - In Vitro Survival of Human Mesenchymal Stem Cells is Enhanced in Artificial Endolymph with Moderately High Concentrations of Potassium

VL - 27

ID - 39317

ER -

TY - JOUR

AB - Mammalian inner ear sensory hair cells are highly sensitive to environmental stress and do not regenerate, making hearing loss progressive and permanent. The paucity and extreme inaccessibility of these cells hinder the development of regenerative and otoprotective strategies, Direct lineage reprogramming to generate large quantities of hair cell-like cells in vitro offers a promising approach to overcome these experimental bottlenecks. Previously, we identified four transcription factors-Six1, Atoh1, Pou4f3, and Gfi1 (SAPG)-capable of converting mouse embryonic fibroblasts, adult tail tip fibroblasts, and postnatal mouse supporting cells into induced hair cell-like cells through retroviral or lentiviral transduction (Menendez et al., 2020). Here, we developed a virus-free, inducible system using a stable human induced pluripotent stem (iPS) cell line carrying doxycycline-inducible SAPG. Our inducible system significantly increases reprogramming efficiency compared to retroviral methods, achieving a ~19-fold greater conversion to a hair cell fate in half the time. Immunostaining, Western blot, and single-nucleus RNA-seq analyses confirm the expression of hair cell-specific markers and activation of hair cell gene networks in reprogrammed cells. The reprogrammed hair cells closely resemble developing fetal hair cells, as evidenced by comparison with a human fetal inner ear dataset. Electrophysiological analysis reveals that the induced hair cell-like cells exhibit diverse voltage-dependent ion currents, including robust, quick-activating, slowly inactivating currents characteristic of primary hair cells. This virus-free approach improves scalability, reproducibility, and the modeling of hair cell differentiation, offering significant potential for hair cell regenerative strategies and preclinical drug discovery targeting ototoxicity and otoprotection.

AD - Department of Stem Cell Biology and Regenerative Medicine, Eli and Edythe Broad Center for Regenerative Medicine and Stem Cell Research, Keck School of Medicine of University of Southern California, Los Angeles, California, United States.

Present address: Touro University of California, College of Osteopathic Medicine, Vallejo, California, United States.

Dornsife College of Letters, Arts and Sciences, University of Southern California, Los Angeles, California, United States.

Biomedical Sciences Department, School of Medicine, Creighton University, Omaha, Nebraska, United States.

Present address: Icahn School of Medicine at Mount Sinai, New York, New York, United States.

Present address: Biology and Biological Engineering, California Institute of Technology, Pasadena, California, United States.

Zilkha Neurogenetic Institute, Keck School of Medicine of University of Southern California, Los Angeles, California, United States.

USC Caruso Department of Otolaryngology - Head and Neck Surgery, Keck School of Medicine of University of Southern California, Los Angeles, California, United States.

Department of Molecular and Human Genetics, Baylor College of Medicine, Houston, Texas, United States.

Department of Neuroscience, Baylor College of Medicine, Houston, Texas, United States.

Department of Developmental Biology, Washington University School of Medicine, St. Louis, Missouri, United States.

AN - 40060658

AU - Rainey, R. N.

AU - Houman, S. D.

AU - Menendez, L.

AU - Chang, R.

AU - Tao, L.

AU - Bugacov, H.

AU - McMahon, A. P.

AU - Kalluri, R.

AU - Oghalai, J. S.

AU - Groves, A. K.

AU - Segil, N.

C1 - PubMed

C2 - PMC11888184 advisor to Novartis, eGENESIS, Trestle Biotherapeutics, and IVIVA Medical. These companies have no competing technology and are not involved in this research. The other authors declare no competing interests.

DA - Feb 25

DO - 10.1101/2025.02.20.639352

DP - NLM

ET - 2025/03/10

LA - eng

N1 - 2692-8205

Rainey, Robert N

Orcid: 0000-0003-2732-493x

Houman, Sam D

Menendez, Louise

Orcid: 0000-0001-6830-0358

Chang, Ryan

Tao, Litao

Bugacov, Helena

Orcid: 0000-0002-9575-3333

McMahon, Andrew P

Orcid: 0000-0002-3779-1729

Kalluri, Radha

Orcid: 0000-0002-0360-8965

Oghalai, John S

Groves, Andrew K

Orcid: 0000-0002-0784-7998

Segil, Neil

Orcid: 0000-0002-0441-2067

R01 DC014832/DC/NIDCD NIH HHS/United States

R01 DC015829/DC/NIDCD NIH HHS/United States

T32 HD060549/HD/NICHD NIH HHS/United States

Journal Article

Preprint

United States

bioRxiv [Preprint]. 2025 Feb 25:2025.02.20.639352. doi: 10.1101/2025.02.20.639352.

PY - 2025

SN - 2692-8205

ST - Inducible, virus-free direct lineage reprogramming enhances scalable generation of human inner ear hair cell-like cells

T2 - bioRxiv

TI - Inducible, virus-free direct lineage reprogramming enhances scalable generation of human inner ear hair cell-like cells

ID - 38880

ER -

TY - JOUR

AB - Inner ear hair cells (HCs) function as the primary transducers for perception of sound and balance, while a defect in their formation or their loss results in sensory deficits. In mammals, once HCs are lost, they are not regenerated, and thus, various medical strategies have been proposed for their reproduction. Although embryonic stem (ES) cells are a promising source for cell therapy, little is known about efficient generation of hair cell-like cells (HCLs) from mouse ES cells. In the present protocol, we describe a simple method for obtaining ES-derived murine HCLs (HIST2 method).

AD - Department of Pathogen, Infection and Immunity, Nara Medical University, 840 Shijo-Cho, Kashihara, Nara, 634-8521, Japan. myoshika@naramed-u.ac.jp.

Department of Pathogen, Infection and Immunity, Nara Medical University, 840 Shijo-Cho, Kashihara, Nara, 634-8521, Japan. oujix@naramed-u.ac.jp.

AN - 27032944

AU - Yoshikawa, M.

AU - Ouji, Y.

C1 - PubMed

DO - 10.1007/7651_2016_328

DP - NLM

ET - 2016/04/02

KW - Animals

Cell Culture Techniques/*methods

Cell Differentiation/*genetics

Hair Cells, Auditory, Inner/*cytology

Mice

Mouse Embryonic Stem Cells/*cytology

Regeneration

Differentiation

Embryonic stem cell

HIST2 method

Hair cells

In vitro culture

Inner ear

LA - eng

N1 - 1940-6029

Yoshikawa, Masahide

Ouji, Yukiteru

Journal Article

United States

Methods Mol Biol. 2016;1516:257-267. doi: 10.1007/7651_2016_328.

PY - 2016

SN - 1064-3745

SP - 257-267

ST - Induction of Inner Ear Hair Cells from Mouse Embryonic Stem Cells In Vitro

T2 - Methods Mol Biol

TI - Induction of Inner Ear Hair Cells from Mouse Embryonic Stem Cells In Vitro

VL - 1516

ID - 39096

ER -

TY - JOUR

AB - The vestibular organs of birds are capable of regenerating sensory hair cells after ototoxic injury, but the regenerative ability of the mammalian vestibular organs is much more limited. The factors that inhibit regeneration in the mammalian inner ear are not known, but it has been proposed that the structure of filamentous actin cables at cell-cell junctions within the sensory epithelium may be an important regulatory influence. Junctional actin cables in the chick utricle are relatively thin, while those in mouse utricle are much thicker. These differences result in differing mechanical properties of the avian vs. mammalian inner ear, which may affect the potential for regenerative proliferation. The present study characterized injury-evoked changes in junctional actin cables in the utricles of mice and chicks. We found that the thickness of junctional cables in the chick utricle was not affected by ototoxic injury, but that injury to the mouse utricle led to the formation of many new junctional actin bands whose thickness was comparable to those in the chick utricle. Thicker actin bands persisted after injury, but were not necessarily associated with cellular junctions. In addition, the relative extent of supporting cell expansion in the injured chick utricle was larger than that in the mouse utricle, which may affect activation of Hippo/YAP signaling in both species. Together, these data point to important differences in actin cable plasticity in the avian vs. mammalian utricle that may partially account for their differing regenerative abilities.

AD - Department of Otolaryngology, Washington University School of Medicine St. Louis MO.

AN - 41394702

AU - Warchol, M. E.

C1 - PubMed

C2 - PMC12697542 that are relevant to this study. Data will be made available upon reasonable request.

DA - Nov 26

DO - 10.1101/2025.11.24.682866

DP - NLM

ET - 2025/12/15

LA - eng

N1 - 2692-8205

Warchol, Mark E

Orcid: 0000-0002-0445-6318

R01 DC006283/DC/NIDCD NIH HHS/United States

Journal Article

Preprint

United States

bioRxiv [Preprint]. 2025 Nov 26:2025.11.24.682866. doi: 10.1101/2025.11.24.682866.

PY - 2025

SN - 2692-8205

ST - Injury-Induced Remodeling of Junctional Actin Bands in the Vestibular Maculae of Mice and Chicks: Implications for Sensory Regeneration

T2 - bioRxiv

TI - Injury-Induced Remodeling of Junctional Actin Bands in the Vestibular Maculae of Mice and Chicks: Implications for Sensory Regeneration

ID - 39511

ER -

TY - JOUR

AB - OBJECTIVES: Current treatments for hearing loss offer some functional improvements in hearing, but do not restore normal hearing. The aim of this review is to highlight recent advances in viral and non-viral vectors for gene therapy and to discuss approaches for overcoming barriers inherent to inner ear delivery of gene products. DATA SOURCES: The databases used were Medline, EMBASE, Web of Science, and Google Scholar. Search terms were [("cochlea*" or "inner ear" or "transtympanic" or "intratympanic" or "intracochlear" or "hair cells" or "spiral ganglia" or "Organ of Corti") and ("gene therapy" or "gene delivery")]. The references section of resulting articles was also used to identify relevant studies. RESULTS: Both viral and non-viral vectors play important roles in advancing gene delivery to the inner ear. The round window membrane is one significant barrier to gene delivery that intratympanic delivery methods attempt to overcome through diffusion and intracochlear delivery methods bypass completely. CONCLUSIONS: Gene therapy for hearing loss is a promising treatment for restoring hearing function by addressing innate defects. Recent technological advances in inner ear drug delivery techniques pose exciting opportunities for progress in gene therapy.

AD - Department of Otolaryngology -- Head and Neck Surgery, Columbia University Vagelos College of Physicians and Surgeons, New York, NY.

Department of Mechanical Engineering, School of Engineering, Columbia University, New York, New York.

AN - 33604229

AU - Valentini, C.

AU - Szeto, B.

AU - Kysar, J. W.

AU - Lalwani, A. K.

C1 - PubMed

C2 - PMC7888570

C6 - NIHMS1660101

DO - 10.1080/21695717.2020.1807261

DP - NLM

ET - 2021/02/20

IS - 4

KW - Gene therapy

hearing loss

inner ear delivery

non-viral vector

viral vector

for Advanced Bionics and on the Surgical Advisory Board for MED-EL. For the

remaining authors, no conflicts of interest were declared.

LA - eng

N1 - 2169-5725

Valentini, Chris

Szeto, Betsy

Kysar, Jeffrey W

Lalwani, Anil K

R01 DC014547/DC/NIDCD NIH HHS/United States

Journal Article

India

Hearing Balance Commun. 2020;18(4):278-285. doi: 10.1080/21695717.2020.1807261. Epub 2020 Aug 25.

PY - 2020

SN - 2169-5717 (Print)

2169-5717

SP - 278-285

ST - Inner Ear Gene Delivery: Vectors and Routes

T2 - Hearing Balance Commun

TI - Inner Ear Gene Delivery: Vectors and Routes

VL - 18

ID - 39599

ER -

TY - JOUR

AB - The development of therapeutic interventions for hearing loss requires fundamental knowledge about the signaling pathways controlling tissue development as well as the establishment of human cell-based assays to validate therapeutic strategies ex vivo. Recent advances in the field of stem cell biology and organoid culture systems allow the expansion and differentiation of tissue-specific progenitors and pluripotent stem cells in vitro into functional hair cells and otic-like neurons. We discuss how inner ear organoids have been developed and how they offer for the first time the opportunity to validate drug-based therapies, gene-targeting approaches and cell replacement strategies. © 2019. Published by The Company of Biologists Ltd

AD - Inner Ear Research Laboratory, Department of Biomedical Research (DBMR), University of Bern, Bern, 3008, Switzerland

Department of Otorhinolaryngology, Head and Neck Surgery, Inselspital, Bern University Hospital, University of Bern, Bern, 3010, Switzerland

Department of Otolaryngology, Harvard Medical School, Boston, 02115, MA, United States

Eaton-Peabody Laboratory, Massachusetts Eye and Ear, Boston, 02114, MA, United States

Harvard Stem Cell Institute, Cambridge, 02138, MA, United States

AU - Roccio, M.

AU - Edge, A. S. B.

C1 - Scopus

C2 - 31477580

C7 - dev177188

DB - Scopus

DO - 10.1242/dev.177188

IS - 17

J2 - Development

KW - Hair cells

Inner ear

Organoids

Regeneration

Stem cells

Adult

Animals

Animals, Newborn

Cell Differentiation

Drug Evaluation, Preclinical

Hair Cells, Auditory, Inner

Hearing Loss

Humans

Infant, Newborn

Mammals

Mice

Neural Stem Cells

Pluripotent Stem Cells

cell degeneration

cell maturation

cell regeneration

cochlea

gene targeting

human

in vitro study

neuroepithelium

nonhuman

organogenesis

organoid

pluripotent stem cell

priority journal

Review

three dimensional cell culture

tissue regeneration

animal

cytology

embryology

genetics

growth, development and aging

hearing impairment

inner hair cell

mammal

metabolism

mouse

neural stem cell

newborn

physiology

preclinical study

procedures

LA - English

M3 - Review

N1 - Export Date: 21 May 2026; Cited By: 65; Correspondence Address: M. Roccio; Inner Ear Research Laboratory, Department of Biomedical Research (DBMR), University of Bern, Bern, 3008, Switzerland; email: marta.roccio@dbmr.unibe.ch; CODEN: DEVPE

PY - 2019

SN - 09501991 (ISSN)

ST - Inner ear organoids: New tools to understand neurosensory cell development, degeneration and regeneration

T2 - Development (Cambridge)

TI - Inner ear organoids: New tools to understand neurosensory cell development, degeneration and regeneration

UR - https://www.scopus.com/pages/publications/85071752799?origin=resultslist

VL - 146

ID - 39868

ER -

TY - JOUR

AB - There are three times as many outer hair cells (OHC) as inner hair cells (IHC), yet IHC transmit virtually all acoustic information to the brain as they synapse with 90-95% of type I auditory nerve fibers. Here we review a comprehensive series of experiments aimed at determining how loss of the IHC/type I system affects hearing by selectively destroying these cells in chinchillas using the ototoxic anti-cancer agent carboplatin. Eliminating IHC/type I neurons has no effect on distortion product otoacoustic emission or the cochlear microphonic potential generated by OHC; however, it greatly reduces the summating potential produced by IHC and the compound action potential (CAP) generated by type I neurons. Remarkably, responses from remaining auditory nerve fibers maintain sharp tuning and low thresholds despite innervating regions of the cochlea with ~80% IHC loss. Moreover, chinchillas with large IHC lesions have surprisingly normal thresholds in quiet until IHC losses exceeded 80%, suggesting that only a few IHC are needed to detect sounds in quiet. However, behavioral thresholds in broadband noise are elevated significantly and tone-in-narrow band noise masking patterns exhibit greater remote masking. These results suggest the auditory system is able to compensate for considerable loss of IHC/type I neurons in quiet but not in difficult listening conditions. How does the auditory brain deal with the drastic loss of cochlear input? Recordings from the inferior colliculus found a relatively small decline in sound-evoked activity despite a large decrease in CAP amplitude after IHC lesion. Paradoxically, sound-evoked responses are generally larger than normal in the auditory cortex, indicative of increased central gain. This gain enhancement in the auditory cortex is associated with decreased GABA-mediated inhibition. These results suggest that when the neural output of the cochlea is reduced, the central auditory system compensates by turning up its gain so that weak signals once again become comfortably loud. While this gain enhancement is able to restore normal hearing under quiet conditions, it may not adequately compensate for peripheral dysfunction in more complex sound environments. In addition, excessive gain increases may convert recruitment into the debilitating condition known as hyperacusis.

AD - Center for Hearing and Deafness, University at Buffalo Buffalo, NY, USA.

Callier Center, University of Texas at Dallas Dallas, TX, USA.

School of Human Communication Disorders, Dalhousie University Halifax, NS, Canada.

AN - 28149271

AU - Salvi, R.

AU - Sun, W.

AU - Ding, D.

AU - Chen, G. D.

AU - Lobarinas, E.

AU - Wang, J.

AU - Radziwon, K.

AU - Auerbach, B. D.

C1 - PubMed

C2 - PMC5241314

DO - 10.3389/fnins.2016.00621

DP - NLM

ET - 2017/02/06

KW - auditory cortex

auditory gain

carboplatin

central auditory system

hyperacusis

inner hair cells

tinnitus

LA - eng

N1 - 1662-453x

Salvi, Richard

Sun, Wei

Ding, Dalian

Chen, Guang-Di

Lobarinas, Edward

Wang, Jian

Radziwon, Kelly

Auerbach, Benjamin D

F32 DC015160/DC/NIDCD NIH HHS/United States

R01 DC014693/DC/NIDCD NIH HHS/United States

Journal Article

Review

Switzerland

Front Neurosci. 2017 Jan 18;10:621. doi: 10.3389/fnins.2016.00621. eCollection 2016.

PY - 2016

SN - 1662-4548 (Print)

1662-453x

SP - 621

ST - Inner Hair Cell Loss Disrupts Hearing and Cochlear Function Leading to Sensory Deprivation and Enhanced Central Auditory Gain

T2 - Front Neurosci

TI - Inner Hair Cell Loss Disrupts Hearing and Cochlear Function Leading to Sensory Deprivation and Enhanced Central Auditory Gain

VL - 10

ID - 39415

ER -

TY - JOUR

AB - Most hearing loss often results from permanent damage to cochlear hair cells, and effective treatments remain limited. A reliable, scalable, and physiologically relevant ear model can accelerate the development of hearing-loss protection therapeutics for injury prevention and hearing restoration. The challenge remains on screening delivery systems for regenerative compounds, and no in vitro screening systems exist that capture the complexity of inner ear properties. Here, we present a high-throughput, microphysiological system (MPS) featuring a round window membrane (RWM) model co-cultured with murine auditory hair cells. It is integrated with a transepithelial electrical resistance (TEER) sensor module to monitor epithelial barrier function development in continuous measurements, without sacrificing a sample and thus allowing “real-time” monitoring of the RWM construct progress. The MPS integrates a syringe pump, tissue compartment, multi-channel fluid distributor, and sensors into a microfluidic continuous-flow system, allowing for on-demand sample collections of analytes triggered by the cellular response to the introduced compounds. Drug screening was conducted with protective antibiotic, antioxidant, and anti-inflammatory compounds. RWM cell and hair cell viability, TD50 values, and membrane integrity were measured. In addition, we also designed a graphene field-effect transistor (GFET)-based cytokine sensor to study proinflammatory cytokine release from cells during damaging exposure. The system was employed to assess drug diffusion efficiency, cell viability, and the drug's TD50 and compared to published data from animal studies. Cell membrane integrity was also analyzed, and proinflammatory cytokine release was measured using a GFET sensor. We evaluated and monitored the real-time structural integrity of the RWM epithelial barrier using the integrated TEER sensor in the MPS. The sensor's ability to measure TEER and cytokine levels was validated by comparing its readings to those obtained from commercial TEER signal processing equipment and standard cytokine concentration measurements. This ear-on-a-chip design enables high-throughput screening of investigational new drugs, reducing the need for animal models in complex studies of inner ear damage and regeneration. It allows for the real-time study of drug responses. It facilitates the development and identifying novel agents that protect against hearing loss and the design of delivery methods for hearing regeneration compounds. © 2025 The Royal Society of Chemistry.

AD - Department of Living Systems, Triton Systems, Inc., Chelmsford, MA, United States

AU - Bai, J.

AU - Bolonduro, O.

AU - Gordiichuk, P.

AU - Green, R. M.

AU - Chung, H. H.

AU - Mahmud, K.

AU - Shvartsman, D.

C1 - Scopus

C2 - 40326305

DB - Scopus

DO - 10.1039/d4lc01025f

IS - 11

J2 - Lab Chip

KW - Animals

Cochlea

Coculture Techniques

Drug Evaluation, Preclinical

Hair Cells, Auditory

Mice

Microfluidic Analytical Techniques

Microphysiological Systems

Round Window, Ear

Antibiotics

Controlled drug delivery

Targeted drug delivery

antiinflammatory agent

beta tubulin

beta tubulin iv

cell marker

ciprofloxacin

cytokeratin 14

cytokeratin 5

cytokine

dexamethasone

erythromycin

gentamicin

interleukin 10

interleukin 12p70

interleukin 6

lactate dehydrogenase

mucin 5AC

new drug

protein ZO1

unclassified drug

Cell viability

Cytokines

Drug response

Electrical resistance sensors

Epithelial barrier

Hair cells

Hearing loss

Inner ear

Real- time

Transepithelial electrical resistances

airway epithelium cell

animal cell

animal experiment

animal model

Article

cell adhesion

cell differentiation

cell viability assay

ciliated epithelium cell

cochlea fenestra

cochlear hair cell

controlled study

cytokine release

diffusivity

drug cytotoxicity

drug diffusion

drug penetration

equipment design

ex vivo study

flow rate

goblet cell

HEI-OC1 cell line

high throughput screening

human

human cell

image display

immunofluorescence assay

impedance spectroscopy

in vitro study

kidney proximal tubule

LC50

liquid chromatography-mass spectrometry

mouse

nonhuman

phase contrast imaging

quantitative analysis

round window membrane

shear stress

simulation

skin fibroblast

transepithelial resistance

animal

coculture

cytology

devices

drug effect

microfluidic analysis

organ on a chip

preclinical study

Integrated circuit design

LA - English

M3 - Article

N1 - Export Date: 21 May 2026; Cited By: 2; Correspondence Address: J. Bai; Department of Living Systems, Triton Systems, Inc., Chelmsford, United States; email: jbai@tritonsys.com; CODEN: LCAHA

PY - 2025

SN - 14730197 (ISSN)

SP - 2744-2756

ST - An integrative round window membrane/cochlear microphysiological system with sensing components for the study of real-time drug response

T2 - Lab on a Chip

TI - An integrative round window membrane/cochlear microphysiological system with sensing components for the study of real-time drug response

UR - https://www.scopus.com/pages/publications/105004917342?origin=resultslist

VL - 25

ID - 39864

ER -

TY - JOUR

AB - Mesenchymal stem cells (MSCs) can be isolated from different tissue origins, such as the bone marrow, the placenta, the umbilical cord, adipose tissues, and skin tissues. MSCs can secrete anti-inflammatory molecules and growth factors for tissue repair and remodeling. However, the ability of skin-derived MSCs (SMSCs) to repair cochlear damage and ameliorate hearing loss remains unclear. Cisplatin is a commonly used chemotherapeutic agent that has the side effect of ototoxicity due to inflammation and oxidative stress. This study investigated the effects of SMSCs on cisplatin-induced hearing loss in mice. Two independent experiments were designed for modeling cisplatin-induced hearing loss in mice, one for chronic toxicity (4 mg/kg intraperitoneal [IP] injection once per day for 5 consecutive days) and the other for acute toxicity (25 mg/kg IP injection once on day one). Three days after cisplatin injection, 1 × 10(6) or 3 × 10(6) SMSCs were injected through the tail vein. Data on auditory brain responses suggested that SMSCs could significantly reduce the hearing threshold of cisplatin-injected mice. Furthermore, immunohistochemical staining data suggested that SMSCs could significantly ameliorate the loss of cochlear hair cells, TUNEL-positive cells and cleaved caspase 3-positive cells in cisplatin-injected mice. Neuropathological gene analyses revealed that SMSCs treatment could downregulate the expression of cochlear genes involved in apoptosis, autophagy, chromatin modification, disease association, matrix remodeling, oxidative stress, tissue integrity, transcription, and splicing and unfolded protein responses. Additionally, SMSCs treatment could upregulate the expression of cochlear genes affecting the axon and dendrite structures, cytokines, trophic factors, the neuronal skeleton and those involved in carbohydrate metabolism, growth factor signaling, myelination, neural connectivity, neural transmitter release, neural transmitter response and reuptake, neural transmitter synthesis and storage, and vesicle trafficking. Results from TUNEL and caspase 3 staining further confirmed that cisplatin-induced apoptosis in cochlear tissues of cisplatin-injected mice could be reduced by SMSCs treatment. In conclusion, the evidence of the effects of SMSCs in favor of ameliorating ototoxicity-induced hearing loss suggests a potential clinical application.

AD - Department of Otolaryngology, Tungs' Taichung Metroharbor Hospital, Taichung, Taiwan.

School of Medicine, Chung Shan Medical University, Taichung, Taiwan.

Department of Medical Research, Tungs' Taichung Metroharbor Hospital, Taichung, Taiwan; Graduate Institute of Biomedical Sciences, China Medical University, Taichung, Taiwan; General Education Center, Jen-Teh Junior College of Medicine, Nursing and Management, Miaoli, Taiwan.

Institute of Biomedical Sciences, Mackay Medical College, New Taipei City, Taiwan.

Graduate Institute of Biomedical Sciences, China Medical University, Taichung, Taiwan; Center for Molecular Medicine, China Medical University Hospital, Taichung, Taiwan; Department of Biotechnology, Asia University, Taichung, Taiwan.

Maria Von Med-Biotechnology Co. Ltd., Taipei, Taiwan.

Medical and Pharmaceutical Industry Technology and Development Center, Taipei, Taiwan.

Laboratory Animal Service Center, Office of Research and Development, China Medical University, Taiwan.

Department of Audiology and Speech-Language Pathology, Mackay Medical College, New Taipei City, Taiwan; Department of Otolaryngology, Mackay Memorial Hospital, Taipei, Taiwan.

Institute of Biomedical Sciences, Mackay Medical College, New Taipei City, Taiwan; Department of Audiology and Speech-Language Pathology, Mackay Medical College, New Taipei City, Taiwan. Electronic address: hsuyc@mmc.edu.tw.

AN - 34020824

AU - Tsai, S. C.

AU - Lin, F. C.

AU - Chang, K. H.

AU - Li, M. C.

AU - Chou, R. H.

AU - Huang, M. Y.

AU - Chen, Y. C.

AU - Kao, C. Y.

AU - Cheng, C. C.

AU - Lin, H. C.

AU - Hsu, Y. C.

C1 - PubMed

DA - Jan

DO - 10.1016/j.heares.2021.108254

DP - NLM

ET - 2021/05/23

KW - Administration, Intravenous

Animals

*Antineoplastic Agents/metabolism

Cisplatin/metabolism/toxicity

Cochlea/pathology

Hair Cells, Auditory, Outer/pathology

*Hearing Loss/chemically induced/metabolism/prevention & control

*Mesenchymal Stem Cells/metabolism

Mice

Cell therapy

Cisplatin

Hair cells

Inner ear

Ototoxicity

Skin-derived mesenchymal stem cells

provided a partial grant. The authors independently characterized the SMSCs and

evaluated the effects of SMSCs in cisplatin-induced hearing loss. Stella

Chin-Shaw Tsai has no relation with Maria Von Med- Biotechnology Co., Ltd. Frank

Cheau-Feng Lin has no relation with Maria Von Med- Biotechnology Co., Ltd.

Kuang-Hsi Chang has no relation with Maria Von Med- Biotechnology Co., Ltd.

Min-Chih Li has no relation with Maria Von Med- Biotechnology Co., Ltd.

Ruey-Hwang Chou has no relation with Maria Von Med- Biotechnology Co., Ltd.

Mei-Yue Huang is the founder of Maria Von Med- Biotechnology Co., Ltd. Yen-Chung

Chen was the chief technology officer in Maria Von Med- Biotechnology Co., Ltd.

Chien-Yu Kao has no relation with Maria Von Med- Biotechnology Co., Ltd.

Ching-Chang Cheng has no relation with Maria Von Med- Biotechnology Co., Ltd.

Hung-Ching Lin has no relation with Maria Von Med- Biotechnology Co., Ltd.

Yi-Chao Hsu has no relation with Maria Von Med- Biotechnology Co., Ltd.

LA - eng

N1 - 1878-5891

Tsai, Stella Chin-Shaw

Lin, Frank Cheau-Feng

Chang, Kuang-Hsi

Li, Min-Chih

Chou, Ruey-Hwang

Huang, Mei-Yue

Chen, Yen-Chung

Kao, Chien-Yu

Cheng, Ching-Chang

Lin, Hung-Ching

Hsu, Yi-Chao

Journal Article

Research Support, Non-U.S. Gov't

Netherlands

Hear Res. 2022 Jan;413:108254. doi: 10.1016/j.heares.2021.108254. Epub 2021 May 5.

PY - 2022

SN - 0378-5955

SP - 108254

ST - The intravenous administration of skin-derived mesenchymal stem cells ameliorates hearing loss and preserves cochlear hair cells in cisplatin-injected mice: SMSCs ameliorate hearing loss and preserve outer hair cells in mice

T2 - Hear Res

TI - The intravenous administration of skin-derived mesenchymal stem cells ameliorates hearing loss and preserves cochlear hair cells in cisplatin-injected mice: SMSCs ameliorate hearing loss and preserve outer hair cells in mice

VL - 413

ID - 39090

ER -

TY - JOUR

AB - CABP2 modulates presynaptic Ca(V)1.3 Ca(2+) channel function in inner hair cells (IHCs) and is required for indefatigable synaptic sound encoding. Biallelic variants in CABP2 are associated with non-syndromic hearing loss (DFNB93). Otoacoustic emissions have been observed in an Italian family with a homozygous CABP2 variant, indicating preservation of outer hair cell-mediated cochlear amplification. Hence, DFNB93 belongs to the hearing disorders caused by impairment of IHC synapses, termed auditory synaptopathy. DFNB93 mouse models have recapitulated findings and demonstrated that lack of CaBP2 impairs synaptic sound encoding by enhanced steady-state inactivation of Ca(V)1.3 Ca(2+) channels. Furthermore, preclinical studies have demonstrated feasibility of gene therapy. As growing evidence from OTOF clinical trials confirms synaptopathies as promising therapeutic targets for hearing restoration, CABP2 ranks highly among the candidate genes for virus-mediated gene therapy to restore hearing. This perspective summarizes the preclinical gene replacement studies for hereditary hearing loss and outlines the characteristics that make genetic targets ideal for therapy development. It reviews the current literature on human CABP2 studies, pre-clinical therapy development, and introduces a patient registry that aims to support research involvement with the CABP2 patient community. We conclude with a preview of the next steps toward CABP2 gene therapy clinical trials.

AD - Institute of Human Genetics University Medical Center Göttingen Göttingen Germany.

Institute for Auditory Neuroscience and InnerEarLab University Medical Center Göttingen Göttingen Germany.

Auditory Neuroscience and Optogenetics Laboratory German Primate Center Göttingen Germany.

Collaborative Research Center 1690 (CRC1690) University of Göttingen Göttingen Germany.

Cluster of Excellence "Multiscale Bioimaging: from Molecular Machines to Networks of Excitable Cells" (MBExC) University of Göttingen Göttingen Germany.

Department of Otolaryngology University Medical Center Göttingen Göttingen Germany.

Auditory Neuroscience and Synaptic Nanophysiology Group Max Planck Institute for Multidisciplinary Sciences Göttingen Germany.

AN - 40927552

AU - Vona, B.

AU - Wollnik, B.

AU - Strenzke, N.

AU - Pangršič, T.

AU - Moser, T.

C1 - PubMed

C2 - PMC12415355

DA - Sep

DO - 10.1002/mco2.70363

DP - NLM

ET - 2025/09/10

IS - 9

KW - Dfnb93

cabp2

calcium‐binding protein 2

hearing impairment

patient registry

synaptopathy

journal's review of or decisions related to this manuscript. The other authors

declare no conflicts of interest.

LA - eng

N1 - 2688-2663

Vona, Barbara

Orcid: 0000-0002-6719-3447

Wollnik, Bernd

Strenzke, Nicola

Pangršič, Tina

Moser, Tobias

Orcid: 0000-0001-7145-0533

Journal Article

China

MedComm (2020). 2025 Sep 8;6(9):e70363. doi: 10.1002/mco2.70363. eCollection 2025 Sep.

PY - 2025

SN - 2688-2663

SP - e70363

ST - Is CABP2-Associated Hearing Loss (DFNB93) a Gene Therapy Target? Preclinical Progress and Patient Registry

T2 - MedComm (2020)

TI - Is CABP2-Associated Hearing Loss (DFNB93) a Gene Therapy Target? Preclinical Progress and Patient Registry

VL - 6

ID - 39648

ER -

TY - JOUR

AB - The mammalian inner ear mediates hearing and balance and during development generates both cochleo-vestibular ganglion neurons and sensory epithelial receptor cells, that is, hair cells and support cells. Cell marking experiments have shown that both hair cells and support cells can originate from a common progenitor. Here, we demonstrate the lineage potential of individual otic epithelial cell clones using three cell lines established by a combination of limiting dilution and gene-marking techniques from an embryonic day 12 (E12) rat otocyst. Cell-type specific marker analyses of these clonal lines under proliferation and differentiation culture conditions demonstrate that during differentiation immature cell markers (Nanog and Nestin) were downregulated and hair cell (Myosin VIIa and Math1), support cell (p27(Kip1) and cytokeratin) and neuronal cell (NF-H and NeuroD) markers were upregulated. Our results suggest that the otic epithelium of the E12 mammalian inner ear possess multipotent progenitor cells able to generate cell types of both sensory epithelial and neural cell lineages when cultured under a differentiation culture condition. Understanding the molecular mechanisms of proliferation and differentiation of multipotent otic progenitor cells may provide insights that could contribute to the development of a novel cell therapy with a potential to initiate or stimulate the sensorineural repair of damaged inner ear sensory receptors. Anat Rec, 303:451-460, 2020. © 2019 American Association for Anatomy.

AD - Department of Otolaryngology-Head and Neck Surgery, Graduate School of Medicine, Kyoto University, Kyoto, Japan.

University of Miami Ear Institute, Department of Otolaryngology, University of Miami School of Medicine, Miami, Florida.

Department of Integrative Brain Science, Graduate School of Medicine, Kyoto University, Kyoto, Japan.

Department of Medical Chemistry, Graduate School of Medicine, Kyoto University, Kyoto, Japan.

Center for Molecular Biology and Genetics, Kyoto University, Kyoto, Japan.

BioMedical Special Research Unit, Human Stress Signal Research Center, National Institute of Advanced Industrial Science and Technology, Ikeda, Japan.

Institute of Neuroscience, INSERM U. 583, University of Montpellier I, Montpellier, France.

AN - 31943808

AU - Kojima, K.

AU - Nishida, A. T.

AU - Tashiro, K.

AU - Hirota, K.

AU - Nishio, T.

AU - Murata, M.

AU - Kato, N.

AU - Kawaguchi, S.

AU - Zine, A.

AU - Ito, J.

AU - Van De Water, T. R.

C1 - PubMed

DA - Mar

DO - 10.1002/ar.24335

DP - NLM

ET - 2020/01/17

IS - 3

KW - Animals

Basic Helix-Loop-Helix Proteins/metabolism

Cell Differentiation/*physiology

Cell Lineage/*physiology

Ear, Inner/*cytology/embryology/metabolism

Hair Cells, Auditory/*cytology/metabolism

Myosin VIIa/metabolism

Nanog Homeobox Protein/metabolism

Nestin/metabolism

Neurons/*cytology/metabolism

Rats

Rats, Wistar

Stem Cells/*cytology/metabolism

ganglion cell

inner ear

multipotent progenitor

otocyst

sensory epithelia

LA - eng

N1 - 1932-8494

Kojima, Ken

Nishida, Akiko T

Tashiro, Kei

Hirota, Kiichi

Orcid: 0000-0003-1110-0827

Nishio, Takeshi

Murata, Miyahiko

Kato, Nobuo

Kawaguchi, Saburo

Zine, Azel

Ito, Juichi

Van De Water, Thomas R

Orcid: 0000-0002-5051-9041

Journal Article

Research Support, Non-U.S. Gov't

United States

Anat Rec (Hoboken). 2020 Mar;303(3):451-460. doi: 10.1002/ar.24335. Epub 2020 Jan 13.

PY - 2020

SN - 1932-8486

SP - 451-460

ST - Isolation and Characterization of Mammalian Otic Progenitor Cells that Can Differentiate into Both Sensory Epithelial and Neuronal Cell Lineages

T2 - Anat Rec (Hoboken)

TI - Isolation and Characterization of Mammalian Otic Progenitor Cells that Can Differentiate into Both Sensory Epithelial and Neuronal Cell Lineages

VL - 303

ID - 39758

ER -

TY - JOUR

AB - Mechanosensory hair cells in the inner ear mediate the sensations of hearing and balance, and in a specialize lateral line sensory system of aquatic vertebrates, the sensation of water movement. In mammals, hair cells lack the ability of regenerate following damage, resulting in sensory deficits. In contrast, non-mammalian vertebrates, such zebrafish, can renew hair cells throughout the life of the animal. Wnt signaling is required for development of inner ear and lateral line hair cells and regulates regeneration. Kremen1 inhibits Wnt signaling and hair cell formation, though its role in regeneration has not been established. We use a zebrafish kremen1 mutant line, to show that when Wnt signaling is overactivated in the lateral line, excessive regeneration occurs in the absence of increased proliferation, due to an increase in support cells. This contrasts with the previously described role of Wnt signaling during hair cell regeneration. This work will allow us to understand the biology of mechanosensory hair cells, and how regeneration might be promoted following damage.

AN - 37546780

AU - Megerson, E.

AU - Kuehn, M.

AU - Leifer, B.

AU - Bell, J.

AU - McGraw, H. F.

C1 - PubMed

C2 - PMC10402150

DA - Jul 28

DO - 10.1101/2023.07.27.550825

DP - NLM

ET - 2023/08/07

LA - eng

N1 - 2692-8205

Megerson, Ellen

Kuehn, Michael

Orcid: 0009-0001-0113-4874

Leifer, Ben

Bell, Jon

McGraw, Hillary F

Orcid: 0000-0002-7318-6374

Journal Article

Preprint

United States

bioRxiv [Preprint]. 2023 Jul 28:2023.07.27.550825. doi: 10.1101/2023.07.27.550825.

PY - 2023

SN - 2692-8205

ST - Kremen1 regulates the regenerative capacity of support cells and mechanosensory hair cells in the zebrafish lateral line

T2 - bioRxiv

TI - Kremen1 regulates the regenerative capacity of support cells and mechanosensory hair cells in the zebrafish lateral line

ID - 39058

ER -

TY - JOUR

AB - Presbycusis is a form of age-related hearing loss (AHL). Many studies have shown that the degeneration of various structures in the cochlea of the inner ear is related to AHL, and DNA damage is an important factor leading to the above process. As an E2 ubiquitin-conjugated enzyme, RAD6B plays an important role in DNA damage repair (DDR) through histone ubiquitination. However, the molecular mechanism is still unclear. In this study, we investigated the role of RAD6B in the morphological changes and DDR mechanisms in aging-related degeneration of the cochlea of mice. We observed that the hair cells, stria vascularis and spiral ganglion in the cochlea of the RAD6B knockout mice showed significant degenerative changes and abnormal expression of proteins associated with DDR mechanisms compared with those of the littermate wild-type mice. In conclusion, our results suggest that the deletion of RAD6B may lead to abnormalities in DDR, thereby accelerating the degeneration of various structures in the cochlea and senescence and apoptosis of cochlea cells.

AD - Department of Anatomy and Histology, School of Basic Medical Sciences, Lanzhou University, Lanzhou, China.

Department of Anatomy and Histology, School of Basic Medical Sciences, Lanzhou University, Lanzhou, China. Electronic address: wangdegui@lzu.edu.cn.

AN - 32868078

AU - Ma, Y.

AU - Song, Y.

AU - Shen, R.

AU - Li, P.

AU - Ding, H.

AU - Guo, Z.

AU - Liu, X.

AU - Wang, D.

C1 - PubMed

DA - Oct 20

DO - 10.1016/j.bbrc.2020.08.017

DP - NLM

ET - 2020/09/02

IS - 3

KW - Aging/*pathology

Animals

Apoptosis

Caspase 3/metabolism

Cell Line

Cochlea/*metabolism/*pathology

Cyclin-Dependent Kinase Inhibitor p16/metabolism

Cyclin-Dependent Kinase Inhibitor p21/metabolism

DNA Damage

DNA Repair

Histones/metabolism

Mice, Knockout

Tumor Suppressor Protein p53/metabolism

Ubiquitin-Conjugating Enzymes/*deficiency/metabolism

Cochlea

Degeneration

Rad6b

Ubiquitin

of interest.

LA - eng

N1 - 1090-2104

Ma, Yangping

Song, Yanfeng

Shen, Rong

Li, Panpan

Ding, Han

Guo, Zhao

Liu, Xiangwen

Wang, Degui

Journal Article

Research Support, Non-U.S. Gov't

United States

Biochem Biophys Res Commun. 2020 Oct 20;531(3):402-408. doi: 10.1016/j.bbrc.2020.08.017. Epub 2020 Aug 28.

PY - 2020

SN - 0006-291x

SP - 402-408

ST - Loss of RAD6B induces degeneration of the cochlea in mice

T2 - Biochem Biophys Res Commun

TI - Loss of RAD6B induces degeneration of the cochlea in mice

VL - 531

ID - 39627

ER -

TY - JOUR

AB - We evaluated the role of iron deficiency (ID) without anemia on hearing function and cochlear pathophysiology of young rats before and after noise exposure. We used rats at developmental stages as an animal model to induce ID without anemia by dietary iron restriction. We have established this dietary restriction model in the rat that should enable us to study the effects of iron deficiency in the absence of severe anemia on hearing and ribbon synapses. Hearing function was measured on Postnatal Day (PND) 21 after induction of ID using auditory brainstem response (ABR). Then, the young rats were exposed to loud noise on PND 21. After noise exposure, hearing function was again measured. We observed the morphology of ribbon synapses, hair cells and spiral ganglion cells (SGCs), and assessed the expression of myosin VIIa, vesicular glutamate transporter 3 and prestin in the cochlea. ID without anemia did not elevate ABR threshold shifts, but reduced ABR wave I peak amplitude of young rats. At 70, 80, and 90 dB SPL, amplitudes of wave I (3.11 ± 0.96 µV, 3.52 ± 1.31 µV, and 4.37 ± 1.08 µV, respectively) in pups from the ID group were decreased compared to the control (5.92 ± 1.67 µV, 6.53 ± 1.70 µV, and 6.90 ± 1.76 µV, respectively) (p < 0.05). Moreover, ID without anemia did not impair the morphology hair cells and SGCs, but decreased the number of ribbon synapses. Before noise exposure, the mean number of ribbon synapses per inner hair cell (IHC) was significantly lower in the ID group (8.44 ± 1.21) compared to that seen in the control (13.08 ± 1.36) (p < 0.05). In addition, the numbers of ribbon synapses per IHC of young rats in the control (ID group) were 6.61 ± 1.59, 3.07 ± 0.83, 5.85 ± 1.63 and 12.25 ± 1.97 (3.75 ± 1.45, 2.03 ± 1.08, 3.81 ± 1.70 and 4.01 ± 1.65) at 1, 4, 7 and 14 days after noise exposure, respectively. Moreover, ABR thresholds at 4 and 8 kHz in young rats from the ID group were significantly elevated at 7 and 14 days after noise exposure compared to control (p < 0.05). The average number of young rat SGCs from the ID group were significantly decreased in the basal turn of the cochlea compared to the control (p < 0.05). Therefore, ID without anemia delayed the recovery from noise-induced hearing loss and ribbon synapses damage, increased SGCs loss, and upregulated prestin after noise exposure. Thus, the cochleae in rat pups with ID without anemia were potentially susceptible to loud noise exposure, and this deficit may be attributed to the reduction of ribbon synapses and SGCs.

AD - Department of Nutrition and Food Hygiene, School of Public Health, China Medical University, No. 77 Puhe Road, Shenyang North New Area, Shenyang 110122, China. yufei@mail.cmu.edu.cn.

Department of Otolaryngology, First Affiliated Hospital of China Medical University, No. 155, Nanjing North Street, Heping District, Shenyang 110001, China. kellya@163.com.

Department of Otolaryngology, First Affiliated Hospital of China Medical University, No. 155, Nanjing North Street, Heping District, Shenyang 110001, China. feigo_dl@163.com.

Department of Nutrition and Food Hygiene, School of Public Health, China Medical University, No. 77 Puhe Road, Shenyang North New Area, Shenyang 110122, China. ford_cmu@163.com.

Department of Nutrition and Food Hygiene, School of Public Health, China Medical University, No. 77 Puhe Road, Shenyang North New Area, Shenyang 110122, China. yangjun@mail.cmu.edu.cn.

AN - 27483303

AU - Yu, F.

AU - Hao, S.

AU - Yang, B.

AU - Zhao, Y.

AU - Yang, J.

C1 - PubMed

C2 - PMC4997371

DA - Jul 28

DO - 10.3390/nu8080456

DP - NLM

ET - 2016/08/03

IS - 8

KW - Anemia, Iron-Deficiency/diet therapy/metabolism/pathology/*physiopathology

Animals

Auditory Cortex/metabolism/physiopathology/ultrastructure

Auditory Threshold/radiation effects

Brain Stem/metabolism/physiopathology/ultrastructure

Cochlea/innervation/metabolism/*physiopathology/ultrastructure

Cochlear Nerve/metabolism/*physiopathology/radiation effects/ultrastructure

*Disease Models, Animal

Disease Susceptibility

Female

Gene Expression Regulation, Developmental/radiation effects

Hearing Loss, Noise-Induced/*etiology/prevention & control

Iron, Dietary/therapeutic use

Male

Microscopy, Electron, Scanning

Nerve Tissue Proteins/genetics/metabolism

Noise/adverse effects

*Nutritional Status

Random Allocation

Rats, Sprague-Dawley

Spiral Ganglion/metabolism/physiopathology/ultrastructure

Weaning

iron deficiency without anemia

ribbon synapse

spiral ganglion cell

LA - eng

N1 - 2072-6643

Yu, Fei

Hao, Shuai

Yang, Bo

Zhao, Yue

Yang, Jun

Journal Article

Retracted Publication

Switzerland

Nutrients. 2016 Jul 28;8(8):456. doi: 10.3390/nu8080456.

PY - 2016

SN - 2072-6643

ST - Low Iron Diet Increases Susceptibility to Noise-Induced Hearing Loss in Young Rats

T2 - Nutrients

TI - Low Iron Diet Increases Susceptibility to Noise-Induced Hearing Loss in Young Rats

VL - 8

ID - 39842

ER -

TY - JOUR

AB - Hearing is initiated in hair cells by the mechanical activation of ion channels in the hair bundle. The hair bundle is formed by stereocilia organized into rows of increasing heights interconnected by tip links, which convey sound-induced forces to stereocilia tips. The auditory mechanosensitive channels are complexes containing at least four protein-subunits - TMC1/2, TMIE, CIB2, and LHFPL5(1-16) - and are located at the tips of shorter stereocilia at a yet-undetermined distance from the lower tip link insertion point(17). While multiple auditory channel subunits appear to interact with the tip link, it remains unknown whether their combined interaction alone can resist the high-frequency mechanical stimulations owing to sound. Here we show that an unanticipated additional element, LOXHD1, is indispensable for maintaining the TMC1 pore-forming channel subunits coupled to the tip link. We demonstrate that LOXHD1 is a unique element of the auditory mechanotransduction complex that selectively affects the localization of TMC1, but not its close developmental paralogue TMC2. Taking advantage of our novel immunogold scanning electron microscopy method for submembranous epitopes (SUB-immunogold-SEM), we demonstrate that TMC1 normally concentrates within 100-nm of the tip link insertion point. In LOXHD1's absence, TMC1 is instead mislocalized away from this force transmission site. Supporting this finding, we found that LOXHD1 interacts selectively in vitro with TMC1 but not with TMC2 while also binding to channel subunits CIB2 and LHFPL5 and tip-link protein PCDH15. SUB-immunogold-SEM additionally demonstrates that LOXHD1 and TMC1 are physically connected to the lower tip-link complex in situ. Our results show that the TMC1-driven mature channels require LOXHD1 to stay coupled to the tip link and remain functional, but the TMC2-driven developmental channels do not. As both tip links and TMC1 remain present in hair bundles lacking LOXHD1, it opens the possibility to reconnect them and restore hearing for this form of genetic deafness.

AD - Department of Otolaryngology-Head and Neck Surgery, Stanford University, 240 Pasteur Drive, Stanford, CA, USA.

Pittsburgh Hearing Research Center, Department of Otolaryngology, University of Pittsburgh, Pittsburgh, PA 15213, USA.

Lead contact.

AN - 38260480

AU - Wang, P.

AU - Miller, K. K.

AU - He, E.

AU - Dhawan, S. S.

AU - Cunningham, C. L.

AU - Grillet, N.

C1 - PubMed

C2 - PMC10802736 Declarations: There is NO Competing Interest.

DA - Jan 2

DO - 10.21203/rs.3.rs-3752492/v1

DP - NLM

ET - 2024/01/23

LA - eng

N1 - 2693-5015

Wang, Pei

Miller, Katharine K

He, Enqi

Dhawan, Siddhant S

Cunningham, Christopher L

Grillet, Nicolas

Orcid: 0000-0001-8007-9246

R01 AG081608/AG/NIA NIH HHS/United States

R01 DC016409/DC/NIDCD NIH HHS/United States

R21 DC019457/DC/NIDCD NIH HHS/United States

Journal Article

Preprint

United States

Res Sq [Preprint]. 2024 Jan 2:rs.3.rs-3752492. doi: 10.21203/rs.3.rs-3752492/v1.

PY - 2024

SN - 2693-5015

ST - LOXHD1 is indispensable for coupling auditory mechanosensitive channels to the site of force transmission

T2 - Res Sq

TI - LOXHD1 is indispensable for coupling auditory mechanosensitive channels to the site of force transmission

ID - 39683

ER -

TY - JOUR

AB - Cisplatin is a widely used and highly effective anti-cancer drug with significant side effects including ototoxicity and nephrotoxicity. Macrophages, the major resident immune cells in the cochlea and kidney, are important drivers of both inflammatory and tissue repair responses. To investigate the roles of macrophages in cisplatin-induced ototoxicity and nephrotoxicity, we used PLX3397, an FDA-approved inhibitor of the colony-stimulating factor 1 receptor (CSF1R), to eliminate tissue-resident macrophages during the course of cisplatin administration. Mice treated with cisplatin alone (cisplatin/vehicle) had significant hearing loss (ototoxicity) as well as kidney injury (nephrotoxicity). Macrophage ablation using PLX3397 resulted in significantly reduced hearing loss measured by auditory brainstem responses (ABR) and distortion-product otoacoustic emissions (DPOAE). Sensory hair cells in the cochlea were protected against cisplatin-induced death in mice treated with PLX3397. Macrophage ablation also protected against cisplatin-induced nephrotoxicity, as evidenced by markedly reduced tubular injury and fibrosis as well as reduced plasma blood urea nitrogen (BUN) and neutrophil gelatinase-associated lipocalin (NGAL) levels. Mechanistically, our data suggest that the protective effect of macrophage ablation against cisplatin-induced ototoxicity and nephrotoxicity is mediated by reduced platinum accumulation in both the inner ear and the kidney. Together our data indicate that ablation of tissue-resident macrophages represents a novel strategy for mitigating cisplatin-induced ototoxicity and nephrotoxicity.

AD - Laboratory of Hearing Biology and Therapeutics, National Institute on Deafness and Other Communication Disorders (NIDCD), NIH, Bethesda, Maryland, USA.

Renal Diagnostics and Therapeutics Unit, National Institute of Diabetes and Digestive and Kidney Diseases (NIDDK), NIH, Bethesda, Maryland, USA.

Bioinformatics and Biostatistics Collaboration Core, National Institute on Deafness and Other Communication Disorders (NIDCD), NIH, Bethesda, Maryland, USA.

Washington University, Department of Otolaryngology, School of Medicine, Saint Louis, MO.

AN - 38014097

AU - Sung, C. Y. W.

AU - Hayase, N.

AU - Yuen, P. S. T.

AU - Lee, J.

AU - Fernandez, K.

AU - Hu, X.

AU - Cheng, H.

AU - Star, R. A.

AU - Warchol, M. E.

AU - Cunningham, L. L.

C1 - PubMed

C2 - PMC10680818

DA - Nov 17

DO - 10.1101/2023.11.16.567274

DP - NLM

ET - 2023/11/28

KW - Ablation

Auditory Brainstem Response (ABR)

Csf1r

Cisplatin

Hair cells

Kidney

Macrophages

Nephrotoxicity

Ototoxicity

Plx3397

LA - eng

N1 - 2692-8205

Sung, Cathy Yea Won

Orcid: 0000-0002-1795-2345

Hayase, Naoki

Yuen, Peter S T

Lee, John

Fernandez, Katharine

Hu, Xuzhen

Cheng, Hui

Star, Robert A

Warchol, Mark E

Cunningham, Lisa L

R01 DC006283/DC/NIDCD NIH HHS/United States

ZIA DC000079/ImNIH/Intramural NIH HHS/United States

ZIA DK043403/ImNIH/Intramural NIH HHS/United States

ZIC DC000080/ImNIH/Intramural NIH HHS/United States

Journal Article

Preprint

United States

bioRxiv [Preprint]. 2023 Nov 17:2023.11.16.567274. doi: 10.1101/2023.11.16.567274.

PY - 2023

SN - 2692-8205

ST - Macrophage Depletion Protects Against Cisplatin-Induced Ototoxicity and Nephrotoxicity

T2 - bioRxiv

TI - Macrophage Depletion Protects Against Cisplatin-Induced Ototoxicity and Nephrotoxicity

ID - 39554

ER -

TY - JOUR

AB - Macrophages are abundant in the cochlea; however, their role in hearing loss is not well understood. Insults to the cochlea, such as noise or insertion of a cochlear implant, cause an inflammatory response, which includes activation of tissue-resident macrophages. Activation is characterized by changes in macrophage morphology, mediator expression, and distribution. Evidence from other organs shows activated macrophages can become primed, whereby subsequent insults cause an elevated inflammatory response. Primed macrophages in brain pathologies respond to circulating inflammatory mediators by disproportionate synthesis of inflammatory mediators. This signaling occurs behind an intact blood-brain barrier, similar to the blood-labyrinth barrier in the cochlea. Local tissue damage can occur as the result of mediator release by activated macrophages. Damage is typically localized; however, if it is to structures with limited ability to repair, such as neurons or hair cells within the cochlea, it is feasible that this contributes to the progressive loss of function seen in hearing loss. We propose that macrophages in the cochlea link risk factors and hearing loss. Injury to the cochlea causes local macrophage activation that typically resolves. However, in susceptible individuals, some macrophages enter a primed state. Once primed, these macrophages can be further activated, as a consequence of circulating inflammatory molecules associated with common co-morbidities. Hypothetically, this would lead to further cochlear damage and loss of hearing. We review the evidence for the role of tissue-resident macrophages in the cochlea and propose that cochlear macrophages contribute to the trajectory of hearing loss and warrant further study.

AD - Faculty of Engineering and Physical Sciences, University of Southampton, Southampton, UK.

Faculty of Engineering and Physical Sciences, Auditory Implant Centre, University of Southampton, Southampton, UK.

School of Biochemistry & Immunology, Trinity Biomedical Sciences Institute & Trinity College Institute of Neuroscience (TCIN), Dublin, Ireland.

Clinical and Experimental Sciences, Faculty of Medicine, IfLS, University of Southampton, Southampton, UK.

AN - 34536249

AU - Hough, K.

AU - Verschuur, C. A.

AU - Cunningham, C.

AU - Newman, T. A.

C1 - PubMed

DA - Feb

DO - 10.1002/glia.24095

DP - NLM

ET - 2021/09/19

IS - 2

KW - *Cochlea/metabolism/pathology

*Hearing Loss/metabolism/pathology

Humans

Macrophage Activation

Macrophages/metabolism

Risk Factors

cochlea

hearing loss

immune memory

immune response

inflammation

macrophages

priming

LA - eng

N1 - 1098-1136

Hough, Kate

Orcid: 0000-0002-5160-2517

Verschuur, Carl A

Cunningham, Colm

Newman, Tracey A

Journal Article

Review

United States

Glia. 2022 Feb;70(2):219-238. doi: 10.1002/glia.24095. Epub 2021 Sep 18.

PY - 2022

SN - 0894-1491

SP - 219-238

ST - Macrophages in the cochlea; an immunological link between risk factors and progressive hearing loss

T2 - Glia

TI - Macrophages in the cochlea; an immunological link between risk factors and progressive hearing loss

VL - 70

ID - 39501

ER -

TY - JOUR

AB - The sensory organs of the inner ear contain resident populations of macrophages, which are recruited to sites of cellular injury. Such macrophages are known to phagocytose the debris of dying cells but the full role of macrophages in otic pathology is not understood. Lateral line neuromasts of zebrafish contain hair cells that are nearly identical to those in the inner ear, and the optical clarity of larval zebrafish permits direct imaging of cellular interactions. In this study, we used larval zebrafish to characterize the response of macrophages to ototoxic injury of lateral line hair cells. Macrophages migrated into neuromasts within 20 min of exposure to the ototoxic antibiotic neomycin. The number of macrophages in the near vicinity of injured neuromasts was similar to that observed near uninjured neuromasts, suggesting that this early inflammatory response was mediated by "local" macrophages. Upon entering injured neuromasts, macrophages actively phagocytosed hair cell debris. The injury-evoked migration of macrophages was significantly reduced by inhibition of Src-family kinases. Using chemical-genetic ablation of macrophages before the ototoxic injury, we also examined whether macrophages were essential for the initiation of hair cell regeneration. Results revealed only minor differences in hair cell recovery in macrophage-depleted vs. control fish, suggesting that macrophages are not essential for the regeneration of lateral line hair cells.

AD - Department of Otolaryngology, Washington University School of Medicine, St. Louis, MO, United States.

Department of Neuroscience, Washington University School of Medicine, St. Louis, MO, United States.

Department of Developmental Biology, Washington University School of Medicine, St. Louis, MO, United States.

AN - 33488362

AU - Warchol, M. E.

AU - Schrader, A.

AU - Sheets, L.

C1 - PubMed

C2 - PMC7820375

DO - 10.3389/fncel.2020.613246

DP - NLM

ET - 2021/01/26

KW - hair cell

inner ear

lateral line

macrophage

ototoxicity

regeneration

zebrafish

commercial or financial relationships that could be construed as a potential

conflict of interest.

LA - eng

N1 - 1662-5102

Warchol, Mark E

Schrader, Angela

Sheets, Lavinia

R01 DC006283/DC/NIDCD NIH HHS/United States

Journal Article

Switzerland

Front Cell Neurosci. 2021 Jan 8;14:613246. doi: 10.3389/fncel.2020.613246. eCollection 2020.

PY - 2020

SN - 1662-5102 (Print)

1662-5102

SP - 613246

ST - Macrophages Respond Rapidly to Ototoxic Injury of Lateral Line Hair Cells but Are Not Required for Hair Cell Regeneration

T2 - Front Cell Neurosci

TI - Macrophages Respond Rapidly to Ototoxic Injury of Lateral Line Hair Cells but Are Not Required for Hair Cell Regeneration

VL - 14

ID - 39192

ER -

TY - JOUR

AB - The lateral membrane of mammalian cochlear outer hair cells contains prestin, a protein which can act as a fast voltage-driven actuator responsible for electromotility and enhanced sensitivity to sound. The protein belongs to the SLC26 family of transporters whose members are characterised as able to exchange halides for SO(4)(2-) or HCO(3)(-) yet previous analyses of mammalian prestin have suggested that such exchange functions were minimal. Here anion transport is investigated both in guinea-pig outer hair cells (OHCs) and in an expression system where we employ a sensitive intracellular pH (pH(i)) probe, pHluorin, to report HCO(3)(-) transport and to monitor the small pH(i) changes observable in the cells. In the presence of extracellular HCO(3)(-), pH(i) recovered from an acid load 4 times faster in prestin-transfected cells. The acceleration required a chloride gradient established by reducing extracellular chloride to 2 mm. Similar results were also shown using BCECF as an alternative pH(i) sensor, but with recovery only found in those cells expressing prestin. Simultaneous electrophysiological recording of the transfected cells during bicarbonate exposure produced a shift in the reversal potential to more negative potentials, consistent with electrogenic transport. These data therefore suggest that prestin can act as a weak Cl(-)/HCO(3)(-) antiporter and it is proposed that, in addition to participating in wide band cochlear sound amplification, prestin may also be involved in the slow time scale (>10 s) phenomena where changes in cell stiffness and internal pressure have been implicated. The results show the importance of considering the effects of the endogenous bicarbonate buffering system in evaluating the function of prestin in cochlear outer hair cells.

AD - UCL Ear Institute, 332 Gray’s Inn Road, London WC1E 6BT, UK.

AN - 22890707

AU - Mistrík, P.

AU - Daudet, N.

AU - Morandell, K.

AU - Ashmore, J. F.

C1 - PubMed

C2 - PMC3528980

DA - Nov 15

DO - 10.1113/jphysiol.2012.241448

DP - NLM

ET - 2012/08/15

IS - 22

KW - Action Potentials

Animals

Anion Transport Proteins/genetics/*physiology

Bicarbonates/metabolism

CHO Cells

Chloride-Bicarbonate Antiporters/*physiology

Chlorine/metabolism

Cricetinae

Cricetulus

Green Fluorescent Proteins/genetics/metabolism

Guinea Pigs

Hair Cells, Auditory, Outer/physiology

Hydrogen-Ion Concentration

Rats

Recombinant Fusion Proteins/genetics/metabolism

Sulfate Transporters

LA - eng

N1 - 1469-7793

Mistrík, P

Daudet, N

Morandell, K

Ashmore, J F

BB/F007175/1/BB_/Biotechnology and Biological Sciences Research Council/United Kingdom

Journal Article

Research Support, Non-U.S. Gov't

England

J Physiol. 2012 Nov 15;590(22):5597-610. doi: 10.1113/jphysiol.2012.241448. Epub 2012 Aug 13.

PY - 2012

SN - 0022-3751 (Print)

0022-3751

SP - 5597-610

ST - Mammalian prestin is a weak Cl⁻/HCO₃⁻ electrogenic antiporter

T2 - J Physiol

TI - Mammalian prestin is a weak Cl⁻/HCO₃⁻ electrogenic antiporter

VL - 590

ID - 39770

ER -

TY - JOUR

AB - Hair cells in the inner ear convert mechanical stimuli provided by sound waves and head movements into electrical signal. Several mechanically evoked ionic currents with different properties have been recorded in hair cells. The search for the proteins that form the underlying ion channels is still in progress. The mechanoelectrical transduction (MET) channel near the tips of stereociliary in hair cells, which is responsible for sensory transduction, has been studied most extensively. Several components of the sensory mechanotransduction machinery in stereocilia have been identified, including the multi-transmembrane proteins tetraspan membrane protein in hair cell stereocilia (TMHS)/LHFPL5, transmembrane inner ear (TMIE) and transmembrane channel-like proteins 1 and 2 (TMC1/2). However, there remains considerable uncertainty regarding the molecules that form the channel pore. In addition to the sensory MET channel, hair cells express the mechanically gated ion channel PIEZO2, which is localized near the base of stereocilia and not essential for sensory transduction. The function of PIEZO2 in hair cells is not entirely clear but it might have a role in damage sensing and repair processes. Additional stretch-activated channels of unknown molecular identity and function have been found to localize at the basolateral membrane of hair cells. Here, we review current knowledge regarding the different mechanically gated ion channels in hair cells and discuss open questions concerning their molecular composition and function.

AD - Solomon H. Snyder Department of Neuroscience, Johns Hopkins University School of Medicine, Baltimore, MD, United States.

Department of Cell Biology, Johns Hopkins University School of Medicine, Baltimore, MD, United States.

AN - 29755320

AU - Qiu, X.

AU - Müller, U.

C1 - PubMed

C2 - PMC5932396

DO - 10.3389/fncel.2018.00100

DP - NLM

ET - 2018/05/15

KW - Lhfpl5

Piezo2

Tmc1

Tmie

auditory

hair cell

inner ear

mechanotransduction

LA - eng

N1 - 1662-5102

Qiu, Xufeng

Müller, Ulrich

R01 DC005965/DC/NIDCD NIH HHS/United States

R01 DC014427/DC/NIDCD NIH HHS/United States

R01 DC014713/DC/NIDCD NIH HHS/United States

R01 DC016960/DC/NIDCD NIH HHS/United States

Journal Article

Review

Switzerland

Front Cell Neurosci. 2018 Apr 11;12:100. doi: 10.3389/fncel.2018.00100. eCollection 2018.

PY - 2018

SN - 1662-5102 (Print)

1662-5102

SP - 100

ST - Mechanically Gated Ion Channels in Mammalian Hair Cells

T2 - Front Cell Neurosci

TI - Mechanically Gated Ion Channels in Mammalian Hair Cells

VL - 12

ID - 39352

ER -

TY - JOUR

AB - Mechanotransducer channels at the tips of sensory stereocilia of inner ear hair cells are gated by the tension of 'tip links' interconnecting stereocilia. To ensure maximal sensitivity, tip links are tensioned at rest, resulting in a continuous influx of Ca(2+) into the cell. Here, we show that this constitutive Ca(2+) influx, usually considered as potentially deleterious for hair cells, is in fact essential for stereocilia stability. In the auditory hair cells of young postnatal mice and rats, a reduction in mechanotransducer current, via pharmacological channel blockers or disruption of tip links, leads to stereocilia shape changes and shortening. These effects occur only in stereocilia that harbor mechanotransducer channels, recover upon blocker washout or tip link regeneration and can be replicated by manipulations of extracellular Ca(2+) or intracellular Ca(2+) buffering. Thus, our data provide the first experimental evidence for the dynamic control of stereocilia morphology by the mechanotransduction current.

AD - Department of Physiology, College of Medicine, University of Kentucky, Lexington, United States.

AN - 28350294

AU - Vélez-Ortega, A. C.

AU - Freeman, M. J.

AU - Indzhykulian, A. A.

AU - Grossheim, J. M.

AU - Frolenkov, G. I.

C1 - PubMed

C2 - PMC5407859

DA - Mar 28

DO - 10.7554/eLife.24661

DP - NLM

ET - 2017/03/30

KW - Animals

Animals, Newborn

Calcium/metabolism

Hair Cells, Auditory/*physiology/*ultrastructure

*Mechanotransduction, Cellular

Mice, Inbred C57BL

Microscopy, Electron, Scanning

Rats, Sprague-Dawley

Stereocilia/*physiology/*ultrastructure

actin

calcium

cell biology

deafness

hair cells

mechanotransduction

mouse

neuroscience

rat

stereocilia

LA - eng

N1 - 2050-084x

Vélez-Ortega, A Catalina

Orcid: 0000-0001-9157-8390

Freeman, Mary J

Indzhykulian, Artur A

Grossheim, Jonathan M

Frolenkov, Gregory I

Orcid: 0000-0002-9810-5024

R01 DC008861/DC/NIDCD NIH HHS/United States

R01 DC014658/DC/NIDCD NIH HHS/United States

Journal Article

Research Support, N.I.H., Extramural

Research Support, Non-U.S. Gov't

England

Elife. 2017 Mar 28;6:e24661. doi: 10.7554/eLife.24661.

PY - 2017

SN - 2050-084x

ST - Mechanotransduction current is essential for stability of the transducing stereocilia in mammalian auditory hair cells

T2 - Elife

TI - Mechanotransduction current is essential for stability of the transducing stereocilia in mammalian auditory hair cells

VL - 6

ID - 39199

ER -

TY - JOUR

AB - Mechanosensory hair cells (HCs) residing in the inner ear are critical for hearing and balance. Precise coordination of proliferation, sensory specification, and differentiation during development is essential to ensure the correct patterning of HCs in the cochlear and vestibular epithelium. Recent studies have revealed that FGF20 signaling is vital for proper HC differentiation. However, the mechanisms by which FGF20 signaling promotes HC differentiation remain unknown. Here, we show that mitogen-activated protein 3 kinase 4 (MEKK4) expression is highly regulated during inner ear development and is critical to normal cytoarchitecture and function. Mice homozygous for a kinase-inactive MEKK4 mutation exhibit significant hearing loss. Lack of MEKK4 activity in vivo also leads to a significant reduction in the number of cochlear and vestibular HCs, suggesting that MEKK4 activity is essential for overall development of HCs within the inner ear. Furthermore, we show that loss of FGF20 signaling in vivo inhibits MEKK4 activity, whereas gain of Fgf20 function stimulates MEKK4 expression, suggesting that Fgf20 modulates MEKK4 activity to regulate cellular differentiation. Finally, we demonstrate, for the first time, that MEKK4 acts as a critical node to integrate FGF20-FGFR1 signaling responses to specifically influence HC development and that FGFR1 signaling through activation of MEKK4 is necessary for outer hair cell differentiation. Collectively, this study provides compelling evidence of an essential role for MEKK4 in inner ear morphogenesis and identifies the requirement of MEKK4 expression in regulating the specific response of FGFR1 during HC development and FGF20/FGFR1 signaling activated MEKK4 for normal sensory cell differentiation. SIGNIFICANCE STATEMENT: Sensory hair cells (HCs) are the mechanoreceptors within the inner ear responsible for our sense of hearing. HCs are formed before birth, and mammals lack the ability to restore the sensory deficits associated with their loss. In this study, we show, for the first time, that MEKK4 signaling is essential for the development of normal cytoarchitecture and hearing function as MEKK4 signaling-deficient mice exhibit a significant reduction of HCs and a hearing loss. We also identify MEKK4 as a critical hub kinase for FGF20-FGFR1 signaling to induce HC differentiation in the mammalian cochlea. These results reveal a new paradigm in the regulation of HC differentiation and provide significant new insights into the mechanism of Fgf signaling governing HC formation.

AD - Department of Pathology and Laboratory Medicine, Medical University of South Carolina, Charleston, South Carolina 29425, and.

Department of Otorhinolaryngology Head and Neck Surgery, School of Medicine, University of Maryland, Baltimore, Maryland 21201.

Department of Pathology and Laboratory Medicine, Medical University of South Carolina, Charleston, South Carolina 29425, and puligill@musc.edu.

AN - 26818521

AU - Haque, K.

AU - Pandey, A. K.

AU - Zheng, H. W.

AU - Riazuddin, S.

AU - Sha, S. H.

AU - Puligilla, C.

C1 - PubMed

C2 - PMC4728730

DA - Jan 27

DO - 10.1523/jneurosci.1853-15.2016

DP - NLM

ET - 2016/01/29

IS - 4

KW - Animals

Animals, Newborn

Basic Helix-Loop-Helix Proteins/metabolism

Cell Differentiation/genetics

*Ear, Inner/cytology/enzymology/growth & development

Embryo, Mammalian

Evoked Potentials, Auditory, Brain Stem/genetics

Female

Gene Expression Regulation, Developmental/genetics/*physiology

Hair Cells, Auditory, Inner/physiology

MAP Kinase Kinase Kinase 4/genetics/*metabolism

Male

Mice

Mice, Transgenic

Mutation/genetics

Nerve Tissue Proteins/metabolism

Pregnancy

Repressor Proteins/metabolism

SOXB1 Transcription Factors/metabolism

Sensory Receptor Cells/*physiology

Signal Transduction/genetics/*physiology

Spiral Ganglion/cytology

Tubulin/metabolism

Fgf20

Mekk4

cellular differentiation

cochlea

mouse

LA - eng

N1 - 1529-2401

Haque, Khujista

Pandey, Atul K

Zheng, Hong-Wei

Riazuddin, Saima

Orcid: 0000-0002-8645-4761

Sha, Su-Hua

Puligilla, Chandrakala

Orcid: 0000-0003-4474-1022

C06 RR014516/RR/NCRR NIH HHS/United States

R01 DC011803/DC/NIDCD NIH HHS/United States

Journal Article

Research Support, N.I.H., Extramural

United States

J Neurosci. 2016 Jan 27;36(4):1347-61. doi: 10.1523/JNEUROSCI.1853-15.2016.

PY - 2016

SN - 0270-6474 (Print)

0270-6474

SP - 1347-61

ST - MEKK4 Signaling Regulates Sensory Cell Development and Function in the Mouse Inner Ear

T2 - J Neurosci

TI - MEKK4 Signaling Regulates Sensory Cell Development and Function in the Mouse Inner Ear

VL - 36

ID - 39787

ER -

TY - JOUR

AB - OBJECTIVE: To investigate the delivery efficiency of human umbilical cord mesenchymal stem cell-derived exosomes (hucMSC-EXOs) via intratympanic injection into vestibular end organs, evaluate their protective effects against gentamicin-induced vestibular dysfunction and hearing loss on gentamicin-induced vestibular dysfunction and hearing loss, and explore their regulatory mechanisms on hair cell apoptosis and autophagy. METHODS: Exosome characteristics were identified by transmission electron microscopy, nanoparticle tracking analysis, and Western blot. PKH26 labeling was used to trace their distribution in the vestibule. SD rats were randomly divided into four groups: control group, gentamicin group (GEN group), gentamicin + exosome group (GEN + EXO group), and gentamicin + dexamethasone group (GEN + DEX group). On day 6 after administration, vestibular function was assessed via open-field test and beam balance test. On day 7, high-frequency hearing (32 kHz) was detected by auditory brainstem response (ABR). The quantity and structural changes of hair cells were analyzed by immunofluorescence staining and scanning electron microscopy. Proteomics was used to analyze differentially expressed proteins in vestibular tissues treated with dexamethasone or hucMSC-EXOs. The regulatory effects on Caspase-3 (apoptosis) and LC3 (autophagy) were validated by immunofluorescence. RESULTS: hucMSC-EXOs administered via intratympanic injection were found to target the utricle, saccule, and crista ampullaris. Behavioral studies showed that the GEN + EXO group exhibited significant suppression of gentamicin-induced reduction in total movement distance (p < 0.05) and movement speed (p < 0.05, superior to the GEN + DEX group), with a 60.5% reduction in beam balance test passage time (p < 0.05). ABR results revealed that the auditory threshold at 32 kHz in the GEN + EXO group was 18.3 dB SPL lower than that in the injury group (p < 0.01), with no statistical difference compared to the GEN + DEX group. Hair cell counting showed significant protective effects of exosomes in reducing hair cell loss in the utricular striola (+ 25%), saccular striola (+ 44%), and central crista ampullaris (+ 44%), with partial regions outperforming the GEN + DEX group (e.g., 109% repair rate in the saccular striola). Scanning electron microscopy confirmed that exosomes reduced hair cell cilia loss and structural vacuolization. Proteomics showed significant activation of the complement and coagulation cascades in the GEN group, while the GEN + EXO group enhanced autophagy via enriching the vesicle transport-related SNARE interaction pathway and improved energy metabolism and oxidative stress by regulating metabolic pathways. Immunofluorescence demonstrated that exosomes significantly reduced the expression of the apoptosis marker Caspase-3 (p < 0.01) and enhanced the activity of the autophagy marker LC3 (p < 0.05). CONCLUSIONS: hucMSC-EXOs delivered via intratympanic injection effectively target vestibular sensory epithelia, significantly suppressing gentamicin-induced balance dysfunction and high-frequency hearing loss, with superior efficacy in partial indicators (e.g., movement speed, saccular hair cell repair) compared to dexamethasone. hucMSC-EXOs exert protective effects through a dual mechanism of "anti-apoptosis and pro-autophagy," providing a novel therapeutic strategy for vestibular hair cell injury.

AD - Department of Otorhinolaryngology, The First Hospital of Dalian Medical University, No.222 Zhongshan Road, Dalian, 116011, Liaoning, China.

Department of Otorhinolaryngology, The Sixth Medical Center of Chinese People's Liberation Army General Hospital, Beijing, China.

Department of Otorhinolaryngology, The First Hospital of Dalian Medical University, No.222 Zhongshan Road, Dalian, 116011, Liaoning, China. shilin516@sina.com.

Stem Cell Clinical Research Center, National Joint Engineering Laboratory, Regenerative Medicine Center, The First Affiliated Hospital of Dalian Medical University, Dalian, 116012, Liaoning, China. wangliang@dmu.edu.cn.

AN - 41350742

AU - Han, R.

AU - Yu, N.

AU - Qi, G.

AU - Wang, J.

AU - Wu, Y.

AU - Qin, C.

AU - Shi, L.

AU - Wang, L.

C1 - PubMed

C2 - PMC12797357

DA - Dec 5

DO - 10.1186/s13287-025-04819-z

DP - NLM

ET - 2025/12/06

IS - 1

KW - Animals

*Exosomes/metabolism/transplantation

*Gentamicins/toxicity/adverse effects

*Autophagy/drug effects

*Mesenchymal Stem Cells/metabolism/cytology

Rats

Rats, Sprague-Dawley

*Hair Cells, Vestibular/metabolism/drug effects/pathology

Humans

Male

Apoptosis/drug effects

Apoptosis

Autophagy

Exosomes

Gentamicin ototoxicity

Intratympanic injection

Vestibular dysfunction

cell-derived exosomes used in this study are sourced from a research project that

has obtained ethical approval. The source project (title: Clinical Study on

Umbilical Cord Mesenchymal Stem Cells in the Treatment of Acute Ischemic Stroke)

was reviewed and approved by the Ethics Committee of the Stem Cell Clinical

Research Institution, the First Affiliated Hospital of Dalian Medical University,

with the approval number 2022-01 and the approval date of January 16, 2022. This

study only involves the use of the aforementioned exosomes and does not involve

human samples or human participation, thus there is no need to obtain written

informed consent from patients or their guardians/legally authorized

representatives. Animal ethics statement: The animal study was approved by the

Medical Ethics Committee of PLA General Hospital, with the project title

“Research on Intelligent Recognition of Auditory Brainstem Evoked Potentials

Based on Deep Learning” and approval date on March 2, 2021. The approval number

is S2020-465-01. All procedures were conducted in accordance with relevant

guidelines and regulations. Competing interests: The authors declare no competing

interests.

LA - eng

N1 - 1757-6512

Han, Runnan

Yu, Ning

Qi, GuoWei

Wang, Jing

Wu, Yanan

Qin, Chuan

Shi, Lin

Wang, Liang

JYTMS20230575/the Fund of Liaoning Provincial Education Department/

82072953/National Natural Science Foundation of China/

XLYC1907009/the Top Young Talent Program of Liaoning Provincial Government/

2021RJ12/Dalian Outstanding Young Talents Project/

Journal Article

England

Stem Cell Res Ther. 2025 Dec 5;17(1):20. doi: 10.1186/s13287-025-04819-z.

PY - 2025

SN - 1757-6512

SP - 20

ST - Mesenchymal stem cell-derived exosomes ameliorate gentamicin-induced vestibular hair cell injury by regulating the SNARE pathway and enhancing autophagy

T2 - Stem Cell Res Ther

TI - Mesenchymal stem cell-derived exosomes ameliorate gentamicin-induced vestibular hair cell injury by regulating the SNARE pathway and enhancing autophagy

VL - 17

ID - 39823

ER -

TY - JOUR

AB - The methionine sulfoxide reductase (Msr) family of proteins is a class of repair enzymes that reduce methionine-S (MsrA) or methionine-R (MsrB) sulfoxide to methionine. Recent studies have reported that mutations in the MSRB3 gene cause autosomal recessive hearing loss in humans, and in mice MsrB3 deficiency leads to profound hearing loss due to hair cell apoptosis and stereocilia degeneration. However, apart from MsrB3, studies on Msr proteins in the inner ear have not yet been reported. In this study, we identified and characterized Msr expression in the cochlea and vestibule. First, we confirmed RNA expression levels of Msr family members in the cochlea and vestibule using reverse transcription PCR and detected Msr family members in both tissues. We also conducted immunohistochemical staining to localize Msr family members within the cochlea and vestibule. In the cochlea, MsrA was detected in supporting cells, spiral ligament, spiral limbus, Reissner's membrane and the spiral ganglion. MsrB1 was specifically expressed in hair cells and the spiral ganglion. MsrB2 was noted in the spiral ganglion, tectorial membrane and stria vascularis. In the vestibule, MsrA and MsrB1 were detected in hair cells and the vestibular ganglion, while MsrB2 was restricted to the vestibular ganglion. In this study, we identified distinct distributions of Msr family members in the organ of Corti and hypothesized that MsrA, MsrB1 and MsrB2 protect proteins in the organ of Corti from oxidative stress.

AD - School of Life Sciences, KNU Creative BioResearch Group (BK21 Plus Project), Kyungpook National University, Daegu, South Korea.

AN - 25531578

AU - Kwon, T. J.

AU - Oh, S. K.

AU - Kim, Y. R.

AU - Kim, M. A.

AU - Lee, B.

AU - Choi, K. S.

AU - Lee, J.

AU - Kim, U. K.

AU - Lee, K. Y.

C1 - PubMed

DO - 10.1159/000368893

DP - NLM

ET - 2014/12/23

IS - 4

KW - Animals

Ear, Inner/*pathology

Hearing Loss/*etiology

Humans

Immunohistochemistry

Methionine Sulfoxide Reductases/*therapeutic use

Mice

Oxidative Stress

Reactive Oxygen Species

LA - eng

N1 - 1422-6421

Kwon, Tae-Jun

Oh, Se-Kyung

Kim, Ye-Ri

Kim, Min-A

Lee, Byeonghyeon

Choi, Kwang Shik

Lee, Jaetae

Kim, Un-Kyung

Lee, Kyu-Yup

Journal Article

Research Support, Non-U.S. Gov't

Switzerland

Cells Tissues Organs. 2014;199(4):294-300. doi: 10.1159/000368893. Epub 2014 Dec 13.

PY - 2014

SN - 1422-6405

SP - 294-300

ST - Methionine sulfoxide reductase A, B1 and B2 are likely to be involved in the protection against oxidative stress in the inner ear

T2 - Cells Tissues Organs

TI - Methionine sulfoxide reductase A, B1 and B2 are likely to be involved in the protection against oxidative stress in the inner ear

VL - 199

ID - 39699

ER -

TY - JOUR

AB - Methionine sulfoxide reductase B3 (MsrB3) is a protein repair enzyme that specifically reduces methionine-R-sulfoxide to methionine. A recent genetic study showed that the MSRB3 gene is associated with autosomal recessive hearing loss in human deafness DFNB74. However, the precise role of MSRB3 in the auditory system and the pathogenesis of hearing loss have not yet been determined. This work is the first to generate MsrB3 knockout mice to elucidate the possible pathological mechanisms of hearing loss observed in DFNB74 patients. We found that homozygous MsrB3(-/-) mice were profoundly deaf and had largely unaffected vestibular function, whereas heterozygous MsrB3(+/-) mice exhibited normal hearing similar to that of wild-type mice. The MsrB3 protein is expressed in the sensory epithelia of the cochlear and vestibular tissues, beginning at E15.5 and E13.5, respectively. Interestingly, MsrB3 is densely localized at the base of stereocilia on the apical surface of auditory hair cells. MsrB3 deficiency led to progressive degeneration of stereociliary bundles starting at P8, followed by a loss of hair cells, resulting in profound deafness in MsrB3(-/-) mice. The hair cell loss appeared to be mediated by apoptotic cell death, which was measured using TUNEL and caspase 3 immunocytochemistry. Taken together, our data suggest that MsrB3 plays an essential role in maintaining the integrity of hair cells, possibly explaining the pathogenesis of DFNB74 deafness in humans caused by MSRB3 deficiency.

AD - Department of Biology, College of Natural Sciences.

AN - 24191262

AU - Kwon, T. J.

AU - Cho, H. J.

AU - Kim, U. K.

AU - Lee, E.

AU - Oh, S. K.

AU - Bok, J.

AU - Bae, Y. C.

AU - Yi, J. K.

AU - Lee, J. W.

AU - Ryoo, Z. Y.

AU - Lee, S. H.

AU - Lee, K. Y.

AU - Kim, H. Y.

C1 - PubMed

DA - Mar 15

DO - 10.1093/hmg/ddt549

DP - NLM

ET - 2013/11/06

IS - 6

KW - Animals

Apoptosis

Cochlea/*pathology

Disease Models, Animal

Gene Expression Regulation, Developmental

Hair Cells, Auditory/pathology

Hearing Loss/enzymology/*genetics/*pathology

Humans

Methionine Sulfoxide Reductases/*genetics/metabolism

Mice

Mice, Inbred C57BL

Mice, Knockout

Microscopy, Electron, Scanning

Microscopy, Electron, Transmission

Stereocilia/metabolism/*pathology

LA - eng

N1 - 1460-2083

Kwon, Tae-Jun

Cho, Hyun-Ju

Kim, Un-Kyung

Lee, Eujin

Oh, Se-Kyung

Bok, Jinwoong

Bae, Yong Chul

Yi, Jun-Koo

Lee, Jang Woo

Ryoo, Zae-Young

Lee, Sang Heun

Lee, Kyu-Yup

Kim, Hwa-Young

Journal Article

Research Support, Non-U.S. Gov't

England

Hum Mol Genet. 2014 Mar 15;23(6):1591-601. doi: 10.1093/hmg/ddt549. Epub 2013 Nov 3.

PY - 2014

SN - 0964-6906

SP - 1591-601

ST - Methionine sulfoxide reductase B3 deficiency causes hearing loss due to stereocilia degeneration and apoptotic cell death in cochlear hair cells

T2 - Hum Mol Genet

TI - Methionine sulfoxide reductase B3 deficiency causes hearing loss due to stereocilia degeneration and apoptotic cell death in cochlear hair cells

VL - 23

ID - 39234

ER -

TY - JOUR

AB - miRNAs are essential factors of an extensively conserved post-transcriptional process controlling gene expression at mRNA level. Varoius biological processes such as growth and differentiation are regulated by miRNAs. Web of Science and PubMed databases were searched using the Endnote software for the publications about the role miRNA-183 family in inner ear: hair cell development and deafness published from 2000 to 2016. A triplet of these miRNAs particularly the miR-183 family is highly expressed in vertebrate hair cells, as with some of the peripheral neurosensory cells. Point mutations in one member of this family, miR-96, underlie DFNA50 autosomal deafness in humans and lead to abnormal hair cell development and survival in mice. In zebrafish, overexpression of the miR-183 family induces extra and ectopic hair cells, while knockdown decreases the number of hair cell. The miR-183 family (miR-183, miR-96 and miR-182) is expressed abundantly in some types of sensory cell in the eye, nose and inner ear. In the inner ear, mechanosensory hair cells have a robust expression level. Despite much similarity of these miRs sequences, small differences lead to distinct targeting of messenger RNAs targets. In the near future, miRNAs are likely to be explored as potential therapeutic agents to repair or regenerate hair cells, cell reprogramming and regenerative medicine applications in animal models because they can simultaneously down-regulate dozens or even hundreds of transcripts.

AD - Research Center for Molecular Medicine, Hamadan University of Medical Sciences, Department of Genetics and Molecular Medicine, Hamadan University of Medical Sciences, Hamadan, Iran.

Cellular and Molecular Research Center, Shahrekord University of Medical Sciences, Sharekord, Iran.

AN - 27942598

AU - Mahmoodian Sani, M. R.

AU - Hashemzadeh-Chaleshtori, M.

AU - Saidijam, M.

AU - Jami, M. S.

AU - Ghasemi-Dehkordi, P.

C1 - PubMed

C2 - PMC5144812

DA - Dec

DO - 10.7874/jao.2016.20.3.131

DP - NLM

ET - 2016/12/13

IS - 3

KW - Hair cell

Hearing loss

miR-183 family

miRNA

LA - eng

N1 - 2384-1710

Mahmoodian Sani, Mohammad Reza

Hashemzadeh-Chaleshtori, Morteza

Saidijam, Massoud

Jami, Mohammad-Saeid

Ghasemi-Dehkordi, Payam

Journal Article

Review

Korea (South)

J Audiol Otol. 2016 Dec;20(3):131-138. doi: 10.7874/jao.2016.20.3.131. Epub 2016 Nov 30.

PY - 2016

SN - 2384-1621 (Print)

SP - 131-138

ST - MicroRNA-183 Family in Inner Ear: Hair Cell Development and Deafness

T2 - J Audiol Otol

TI - MicroRNA-183 Family in Inner Ear: Hair Cell Development and Deafness

VL - 20

ID - 38842

ER -

TY - JOUR

AB - Auditory hair cell regeneration following injury is critical to hearing restoration. The Notch signaling pathway participates in the regulation of inner ear development and cell differentiation. Recent evidence suggests that microRNA (miR)‑183 has a similar role in the inner ear. However, it is unclear how Notch signaling functions in hair cell regeneration in mammals and if there is cross‑talk between Notch signaling and miR‑183. The present study used a gentamicin‑induced cochlear injury mouse model. Gentamicin‑induced damage of the hair cells activated the Notch signaling pathway and downregulated miR‑183 expression. Notch signaling inhibition by the γ‑secretase inhibitor, 24‑diamino‑5‑phenylthiazole (DAPT), attenuated gentamicin‑induced hair cell loss and reversed the downregulation of miR‑183 expression. Further investigation revealed that the novel hair cells produced, induced by DAPT, were derived from transdifferentiated supporting cells. Additionally, myosin VI‑positive hair cell numbers were increased by Notch signaling inhibition in in vitro experiments with cultured neonatal mouse inner ear precursor cells. This effect was reversed by miR‑183 inhibition. These findings indicate that the Notch signaling pathway served a repressing role during the regeneration of hair cells. Inhibiting this signal improved hair cell regeneration in the gentamicin‑damaged cochlear model. miR‑183 was demonstrated to be involved in hair cell differentiation and regeneration, and was required for the differentiation of the Notch‑inhibited hair cells.

AD - Department of Otolaryngology, The First Affiliated Hospital and Institute of Otorhinolaryngology, Sun Yat‑sen University, Guangzhou, Guangdong 510080, P.R. China.

AN - 29901127

AU - Zhou, W.

AU - Du, J.

AU - Jiang, D.

AU - Wang, X.

AU - Chen, K.

AU - Tang, H.

AU - Zhang, X.

AU - Cao, H.

AU - Zong, L.

AU - Dong, C.

AU - Jiang, H.

C1 - PubMed

C2 - PMC6072138

DA - Aug

DO - 10.3892/mmr.2018.9127

DP - NLM

ET - 2018/06/15

IS - 2

KW - Animals

*Cell Differentiation

Diamines/pharmacology

Hair Cells, Auditory/*metabolism/pathology

Mice

MicroRNAs/*biosynthesis/genetics

Receptors, Notch/antagonists & inhibitors/genetics/*metabolism

*Regeneration

*Signal Transduction

Thiazoles/pharmacology

LA - eng

N1 - 1791-3004

Zhou, Wei

Du, Jintao

Jiang, Di

Wang, Xianren

Chen, Kaitian

Tang, Haocheng

Zhang, Xuemei

Cao, Hui

Zong, Ling

Dong, Chang

Jiang, Hongyan

Journal Article

Greece

Mol Med Rep. 2018 Aug;18(2):1253-1262. doi: 10.3892/mmr.2018.9127. Epub 2018 Jun 5.

PY - 2018

SN - 1791-2997 (Print)

1791-2997

SP - 1253-1262

ST - microRNA‑183 is involved in the differentiation and regeneration of Notch signaling‑prohibited hair cells from mouse cochlea

T2 - Mol Med Rep

TI - microRNA‑183 is involved in the differentiation and regeneration of Notch signaling‑prohibited hair cells from mouse cochlea

VL - 18

ID - 39290

ER -

TY - JOUR

AB - This review provides an up-to-date source of information on the primary auditory neurons or spiral ganglion neurons in the cochlea. These neurons transmit auditory information in the form of electric signals from sensory hair cells to the first auditory nuclei of the brain stem, the cochlear nuclei. Congenital and acquired neurosensory hearing loss affects millions of people worldwide. An increasing body of evidence suggest that the primary auditory neurons degenerate due to noise exposure and aging more readily than sensory cells, and thus, auditory neurons are a primary target for regenerative therapy. A better understanding of the development and function of these neurons is the ultimate goal for long-term maintenance, regeneration, and stem cell replacement therapy. In this review, we provide an overview of the key molecular factors responsible for the function and neurogenesis of the primary auditory neurons, as well as a brief introduction to stem cell research focused on the replacement and generation of auditory neurons.

AD - BIOCEV, Institute of Biotechnology of the Czech Academy of Sciences, 25250 Vestec, Czech Republic.

AN - 33374462

AU - Pavlinkova, G.

C1 - PubMed

C2 - PMC7796308

DA - Dec 24

DO - 10.3390/ijms22010131

DP - NLM

ET - 2020/12/31

IS - 1

KW - Animals

Base Sequence

Brain Stem

Cochlea/embryology/physiology

Cochlear Nucleus/embryology/physiology

Ear, Inner/embryology/physiology

Evoked Potentials, Auditory, Brain Stem

Hair Cells, Auditory/*physiology

Hearing Loss, Sensorineural/physiopathology

Humans

Induced Pluripotent Stem Cells/cytology

Mice

Mutation

Neurogenesis

Neurons/*physiology

Regenerative Medicine/methods

Spiral Ganglion/embryology/physiology

auditory pathways

cochlea

genetic mutations

single-cell RNAseq

transcription factor

LA - eng

N1 - 1422-0067

Pavlinkova, Gabriela

Orcid: 0000-0002-5689-6577

20-06927S/The Czech Science Foundation/

86652036/the Czech Academy of Sciences RVO/

Journal Article

Review

Switzerland

Int J Mol Sci. 2020 Dec 24;22(1):131. doi: 10.3390/ijms22010131.

PY - 2020

SN - 1422-0067

ST - Molecular Aspects of the Development and Function of Auditory Neurons

T2 - Int J Mol Sci

TI - Molecular Aspects of the Development and Function of Auditory Neurons

VL - 22

ID - 39408

ER -

TY - JOUR

AB - Noise-induced, drug-related, and age-related disabling hearing loss is a major public health problem and affect approximately 466 million people worldwide. In non-mammalian vertebrates, the death of sensory hair cells (HCs) induces the proliferation and transdifferentiation of adjacent supporting cells into new HCs; however, this capacity is lost in juvenile and adult mammalian cochleae leading to permanent hearing loss. At present, cochlear implants and hearing devices are the only available treatments and can help patients to a certain extent; however, no biological approach or FDA-approved drug is effective to treat disabling hearing loss and restore hearing. Recently, regeneration of mammalian cochlear HCs by modulating molecular pathways or transcription factors has offered some promising results, although the immaturity of the regenerated HCs remains the biggest concern. Furthermore, most of the research done is in neonates and not in adults. This review focuses on critically summarizing the studies done in adult mammalian cochleae and discusses various strategies to elucidate novel transcription factors for better therapeutics.

AD - Department of Biomedical Sciences, Creighton University School of Medicine, Omaha, NE 68178, USA.

AN - 35008497

AU - Rai, V.

AU - Tu, S.

AU - Frank, J. R.

AU - Zuo, J.

C1 - PubMed

C2 - PMC8745006

DA - Dec 22

DO - 10.3390/ijms23010066

DP - NLM

ET - 2022/01/12

IS - 1

KW - Animals

Cochlea/*metabolism

Hair Cells, Auditory/*metabolism

Hearing/physiology

Hearing Loss/*metabolism

Humans

Mammals/*metabolism

Regeneration/*physiology

Transcription Factors/metabolism

adult cochlea

bioinformatics

hair cells

regeneration

transcription factor

conflict of interest.

LA - eng

N1 - 1422-0067

Rai, Vikrant

Orcid: 0000-0001-6286-2341

Tu, Shu

Frank, Joseph R

Zuo, Jian

R01DC015010/NH/NIH HHS/United States

R01DC015444/NH/NIH HHS/United States

USAMRMC-RH170030/United States Army Research Office/

LB692/Creighton University/

ONR-N00014-18-1-2507/Office of Naval Research/

Journal Article

Review

Switzerland

Int J Mol Sci. 2021 Dec 22;23(1):66. doi: 10.3390/ijms23010066.

PY - 2021

SN - 1422-0067

ST - Molecular Pathways Modulating Sensory Hair Cell Regeneration in Adult Mammalian Cochleae: Progress and Perspectives

T2 - Int J Mol Sci

TI - Molecular Pathways Modulating Sensory Hair Cell Regeneration in Adult Mammalian Cochleae: Progress and Perspectives

VL - 23

ID - 38863

ER -

TY - JOUR

AB - Loss of auditory sensory hair cells (HCs) is the most common cause of hearing loss. This review addresses the signaling pathways that are involved in the programmed and necrotic cell death of auditory HCs that occur in response to ototoxic and traumatic stressor events. The roles of inflammatory processes, oxidative stress, mitochondrial damage, cell death receptors, members of the mitogen-activated protein kinase (MAPK) signal pathway and pro- and anti-cell death members of the Bcl-2 family are explored. The molecular interaction of these signal pathways that initiates the loss of auditory HCs following acoustic trauma is covered and possible therapeutic interventions that may protect these sensory HCs from loss via apoptotic or non-apoptotic cell death are explored.

AD - University of Miami Ear Institute, University of Miami Miller School of Medicine Miami, FL, USA.

Integrative and Adaptive Neurosciences, Aix-Marseille Université, CNRS, UMR 7260 Marseille, France ; Faculty of Pharmacy, Biophysics Department, University of Montpellier Montpellier, France.

AN - 25873860

AU - Dinh, C. T.

AU - Goncalves, S.

AU - Bas, E.

AU - Van De Water, T. R.

AU - Zine, A.

C1 - PubMed

C2 - PMC4379916

DO - 10.3389/fncel.2015.00096

DP - NLM

ET - 2015/04/16

KW - apoptosis

auditory hair cells

cochlea

inflammation

necrosis

otoprotection

repair

trauma

LA - eng

N1 - 1662-5102

Dinh, Christine T

Goncalves, Stefania

Bas, Esperanza

Van De Water, Thomas R

Zine, Azel

Journal Article

Review

Switzerland

Front Cell Neurosci. 2015 Mar 31;9:96. doi: 10.3389/fncel.2015.00096. eCollection 2015.

PY - 2015

SN - 1662-5102 (Print)

1662-5102

SP - 96

ST - Molecular regulation of auditory hair cell death and approaches to protect sensory receptor cells and/or stimulate repair following acoustic trauma

T2 - Front Cell Neurosci

TI - Molecular regulation of auditory hair cell death and approaches to protect sensory receptor cells and/or stimulate repair following acoustic trauma

VL - 9

ID - 39092

ER -

TY - JOUR

AB - The mechanosensory hair cell of the vertebrate inner ear responds to the mechanical deflections that result from hearing or change in the acceleration due to gravity, to allow us to perceive and interpret sounds, maintain balance and spatial orientation. In mammals, ototoxic compounds, disease, and acoustic trauma can result in damage and extrusion of hair cells, without replacement, resulting in hearing loss. In contrast, non-mammalian vertebrates can regenerate sensory hair cells. Upon damage, hair cells are extruded and an associated cell type, the supporting cell is transformed into a hair cell. The mechanisms that can trigger regeneration are not known. Using mosaic deletion of the hair cell master gene, Atoh1, in the embryonic avian inner ear, we find that despite hair cells depletion at E9, by E12, hair cell number is restored in sensory epithelium. Our study suggests a homeostatic mechanism can restores hair cell number in the basilar papilla, that is activated when juxtracrine signalling is disrupted. Restoration of hair cell numbers during development may mirror regenerative processes, and our work provides insights into the mechanisms that trigger regeneration. © 2024 Elsevier Inc.

AD - National Centre for Biological Sciences, Tata Institute for Fundamental Research, GKVK PO, Bellary Road, Bangalore, 560065, India

The University of Trans-Disciplinary Health Sciences and Technology, Yelahanka, Bangalore, 560064, India

Ashoka University, Haryana, Sonipat, 131029, India

AU - Singh, N.

AU - Kaushik, R.

AU - Prakash, A.

AU - Singh Saini, S.

AU - Garg, S.

AU - Adhikary, A.

AU - Ladher, R. K.

C1 - Scopus

C2 - 39074652

DB - Scopus

DO - 10.1016/j.ydbio.2024.07.017

J2 - Dev. Biol.

KW - Chicken

Hair Cells

Inner Ear

Regeneration

Animals

Basic Helix-Loop-Helix Transcription Factors

Cell Count

Chick Embryo

Chickens

Epithelium

Gene Deletion

Hair Cells, Auditory

Homeostasis

Mosaicism

Organ of Corti

caspase 9

basic helix loop helix transcription factor

animal cell

animal experiment

animal tissue

Article

Atoh1 gene

basilar papilla

cell proliferation

chick

complex formation

controlled study

CRISPR-CAS9 system

embryo

exon

Gallus gallus

gene expression

hair cell (inner ear)

neuroepithelium

nonhuman

protein expression

regulator gene

signal transduction

animal

cochlear hair cell

Corti organ

embryology

genetics

metabolism

physiology

LA - English

M3 - Article

N1 - Export Date: 21 May 2026; Cited By: 1; Correspondence Address: R.K. Ladher; National Centre for Biological Sciences, Tata Institute for Fundamental Research, GKVK PO, Bangalore, Bellary Road, 560065, India; email: rajladher@ncbs.res.in; CODEN: DEBIA

PY - 2024

SN - 00121606 (ISSN)

SP - 35-46

ST - Mosaic Atoh1 deletion in the chick auditory epithelium reveals a homeostatic mechanism to restore hair cell number

T2 - Developmental Biology

TI - Mosaic Atoh1 deletion in the chick auditory epithelium reveals a homeostatic mechanism to restore hair cell number

UR - https://www.scopus.com/pages/publications/85200492893?origin=resultslist

VL - 516

ID - 39867

ER -

TY - JOUR

AB - Hearing loss affects many people worldwide and occurs often as a result of age, ototoxic drugs and/or excessive noise exposure. With a growing number of elderly people, the number of people suffering from hearing loss will also increase in the future. Despite the high number of af-fected people, for most patients there is no curative therapy for hearing loss and hearing aids or cochlea implants remain the only option. Important treatment approaches for hearing loss include the development of regenerative therapies or the inhibition of cell death/promotion of cell survival pathways. The mammalian target of rapamycin (mTOR) pathway is a central regulator of cell growth, is involved in cell survival, and has been shown to be implicated in many age‐related dis-eases. In the inner ear, mTOR signaling has also started to gain attention recently. In this review, we will emphasize recent discoveries of mTOR signaling in the inner ear and discuss implications for possible treatments for hearing restoration. © 2021 by the authors. Licensee MDPI, Basel, Switzer-.

AD - Department of Biomedicine, University of Basel, Hebelstrasse 20, Basel, 4031, Switzerland

Clinic for Otorhinolaryngology, Head and Neck Surgery, University of Basel Hospital, Petersgraben 4, Basel, 4031, Switzerland

AU - Cortada, M.

AU - Levano, S.

AU - Bodmer, D.

C1 - Scopus

C2 - 34198685

C7 - 6368

DB - Scopus

DO - 10.3390/ijms22126368

IS - 12

J2 - Int. J. Mol. Sci.

KW - Cochlea

Hair cells

Hearing loss

Inner ear

MTOR

Protection

Regeneration

Animals

Ear, Inner

Humans

Molecular Targeted Therapy

Signal Transduction

TOR Serine-Threonine Kinases

mammalian target of rapamycin

mammalian target of rapamycin complex 1

mammalian target of rapamycin complex 2

target of rapamycin kinase

cell death

cell growth

cell regeneration

cell survival

feedback system

hair cell (inner ear)

hearing impairment

human

mTOR signaling

nonhuman

oxidative stress

perception deafness

protein function

Review

animal

metabolism

molecularly targeted therapy

pathology

LA - English

M3 - Review

N1 - Export Date: 21 May 2026; Cited By: 18; Correspondence Address: D. Bodmer; Department of Biomedicine, University of Basel, Basel, Hebelstrasse 20, 4031, Switzerland; email: Daniel.Bodmer@usb.ch

PY - 2021

SN - 16616596 (ISSN)

ST - Mtor signaling in the inner ear as potential target to treat hearing loss

T2 - International Journal of Molecular Sciences

TI - Mtor signaling in the inner ear as potential target to treat hearing loss

UR - https://www.scopus.com/pages/publications/85107727678?origin=resultslist

VL - 22

ID - 39865

ER -

TY - JOUR

AB - Cisplatin is a common and effective chemotherapeutic agent, yet it often causes permanent hearing loss as a result of sensory hair cell death. The causes of sensitivity to DNA-damaging agents in nondividing cell populations, such as cochlear hair and supporting cells, are poorly understood, as are the specific DNA repair pathways that protect these cells. Nucleotide excision repair (NER) is a conserved and versatile DNA repair pathway for many DNA-distorting lesions, including cisplatin-DNA adducts. Progressive sensorineural hearing loss is observed in a subset of NER-associated DNA repair disorders including Cockayne syndrome and some forms of xeroderma pigmentosum. We investigated whether either of the two overlapping branches that encompass NER, transcription-coupled repair or global genome repair, which are implicated in Cockayne syndrome and xeroderma pigmentosum group C, respectively, modulates cisplatin-induced hearing loss and cell death in the organ of Corti, the auditory sensory epithelium of mammals. We report that cochlear hair cells and supporting cells in transcription-coupled repair-deficient Cockayne syndrome group A (Csa(-/-)) and group B (Csb(-/-)) mice are hypersensitive to cisplatin, in contrast to global genome repair-deficient Xpc(-/-) mice, both in vitro and in vivo We show that sensory hair cells in Csa(-/-) and Csb(-/-) mice fail to remove cisplatin-DNA adducts efficiently in vitro; and unlike Xpc(-/-) mice, Csa(-/-) and Csb(-/-) mice lose hearing and manifest outer hair cell degeneration after systemic cisplatin treatment. Our results demonstrate that Csa and Csb deficiencies predispose to cisplatin-induced hearing loss and hair/supporting cell damage in the mammalian organ of Corti, and emphasize the importance of transcription-coupled DNA repair in the protection against cisplatin ototoxicity. SIGNIFICANCE STATEMENT: The utility of cisplatin in chemotherapy remains limited due to serious side effects, including sensorineural hearing loss. We show that mouse models of Cockayne syndrome, a progeroid disorder resulting from a defect in the transcription-coupled DNA repair (TCR) branch of nucleotide excision repair, are hypersensitive to cisplatin-induced hearing loss and sensory hair cell death in the organ of Corti, the mammalian auditory sensory epithelium. Our work indicates that Csa and Csb, two genes involved in TCR, are preferentially required to protect against cisplatin ototoxicity, relative to global genome repair-specific elements of nucleotide excision repair, and suggests that TCR is a major force maintaining DNA integrity in the cochlea. The Cockayne syndrome mice thus represent a model for testing the contribution of DNA repair mechanisms to cisplatin ototoxicity.

AD - Department of Stem Cell Biology and Regenerative Medicine, and Caruso Department of Otolaryngology, University of Southern California, Eli and Edythe Broad Center, Los Angeles, California 90033, and.

Department of Genetics, Cancer Genomic Center, Erasmus MC, Rotterdam 3015 CN, The Netherlands.

Department of Stem Cell Biology and Regenerative Medicine, and Caruso Department of Otolaryngology, University of Southern California, Eli and Edythe Broad Center, Los Angeles, California 90033, and nsegil@med.usc.edu.

AN - 27122034

AU - Rainey, R. N.

AU - Ng, S. Y.

AU - Llamas, J.

AU - van der Horst, G. T.

AU - Segil, N.

C1 - PubMed

C2 - PMC4846672

DA - Apr 27

DO - 10.1523/jneurosci.3890-15.2016

DP - NLM

ET - 2016/04/29

IS - 17

KW - Animals

Cell Death

Cisplatin/*toxicity

Cochlea/metabolism

Cockayne Syndrome/*genetics

DNA Adducts

DNA Repair/*genetics

DNA Repair Enzymes/genetics

Disease Models, Animal

Female

Hearing Loss/*chemically induced

Humans

Male

Mice

Mice, Inbred C57BL

Mice, Transgenic

Mutation/*genetics

Xeroderma Pigmentosum/genetics

Cockayne syndrome

DNA repair

cisplatin

hearing loss

ototoxicity

sensory hair cells

LA - eng

N1 - 1529-2401

Rainey, Robert N

Orcid: 0000-0003-2732-493x

Ng, Sum-Yan

Llamas, Juan

van der Horst, Gijsbertus T J

Segil, Neil

F32 DC010125/DC/NIDCD NIH HHS/United States

R01 DC007173/DC/NIDCD NIH HHS/United States

Journal Article

Research Support, N.I.H., Extramural

Research Support, Non-U.S. Gov't

United States

J Neurosci. 2016 Apr 27;36(17):4758-70. doi: 10.1523/JNEUROSCI.3890-15.2016.

PY - 2016

SN - 0270-6474 (Print)

0270-6474

SP - 4758-70

ST - Mutations in Cockayne Syndrome-Associated Genes (Csa and Csb) Predispose to Cisplatin-Induced Hearing Loss in Mice

T2 - J Neurosci

TI - Mutations in Cockayne Syndrome-Associated Genes (Csa and Csb) Predispose to Cisplatin-Induced Hearing Loss in Mice

VL - 36

ID - 39231

ER -

TY - JOUR

AB - Unlike mammals, the non-mammalian vertebrate inner ear can regenerate the sensory cells, hair cells, either spontaneously or through induction after hair cell loss, leading to hearing recovery. The mechanisms underlying the regeneration are poorly understood. By microarray analysis on a chick model, we show that chick hair cell regeneration involves the activation of proliferation genes and downregulation of differentiation genes. Both MYC and FGF are activated in chick hair cell regeneration. Using a zebrafish lateral line neuromast hair cell regeneration model, we show that the specific inhibition of Myc or Fgf suppresses hair cell regeneration, demonstrating that both pathways are essential to the process. Rapid upregulation of Myc and delayed Fgf activation during regeneration suggest a role of Myc in proliferation and Fgf in differentiation. The dorsal-ventral pattern of fgfr1a in the neuromasts overlaps with the distribution of hair cell precursors. By laser ablation, we show that the fgfr1a-positive supporting cells are likely the hair cell precursors that directly give rise to new hair cells; whereas the anterior-posterior fgfr1a-negative supporting cells have heightened proliferation capacity, likely to serve as more primitive progenitor cells to replenish lost precursors after hair cell loss. Thus fgfr1a is likely to mark compartmentalized supporting cell subtypes with different capacities in renewal proliferation and hair cell regeneration. Manipulation of c-MYC and FGF pathways could be explored for mammalian hair cell regeneration. © 2016 Lee et al. This is an open access article distributed under the terms of the Creative Commons Attribution License, which permits unrestricted use, distribution, and reproduction in any medium, provided the original author and source are credited.

AD - Department of Otolaryngology, Harvard Medical School, Boston, MA, United States

Eaton-Peabody Laboratory, Massachusetts Eye and Ear Infirmary, Boston, MA, United States

Department of Systems Biology, Harvard Medical School, Boston, MA, United States

Department of Otorhinolaryngology, Shanghai Eye and ENT Hospital, Shanghai Medical College, Fudan University, Shanghai, China

Department of Otolaryngology, Chinese PLA General Hospital, Beijing, China

Stem Cell Program, Division of Pediatric Hematology/Oncology, Children's Hospital Boston, Dana-Farber Cancer Institute, Boston, MA, United States

Cell and Developmental Biology, University of Massachusetts Medical School, Worcester, MA, United States

AU - Lee, S. G.

AU - Huang, M.

AU - Obholzer, N. D.

AU - Sun, S.

AU - Li, W.

AU - Petrillo, M.

AU - Dai, P.

AU - Zhou, Y.

AU - Cotanche, D. A.

AU - Megason, S. G.

AU - Li, H.

AU - Chen, Z. Y.

C1 - Scopus

C2 - 27351484

C7 - e0157768

DB - Scopus

DO - 10.1371/journal.pone.0157768

IS - 6

J2 - PLoS ONE

KW - Animals

Cell Proliferation

Fibroblast Growth Factors

Lateral Line System

Mast Cells

Neurons, Afferent

Proto-Oncogene Proteins c-myc

Receptors, Fibroblast Growth Factor

Regeneration

Zebrafish

Zebrafish Proteins

fibroblast growth factor

fibroblast growth factor receptor 1

fibroblast growth factor receptor 1a

Myc protein

unclassified drug

fibroblast growth factor receptor

zebrafish protein

adult

animal cell

animal tissue

Article

cell compartmentalization

cell differentiation

cell loss

cell regeneration

cell renewal

cell transdifferentiation

chick

controlled study

down regulation

fgfr1a gne

gene activation

gene expression

growth inhibition

hair cell

microarray analysis

neuromast hair cell

nonhuman

protein analysis

protein function

stem cell

zebra fish

animal

cytology

genetics

mast cell

metabolism

physiology

sensory nerve cell

LA - English

M3 - Article

N1 - Export Date: 21 May 2026; Cited By: 24; CODEN: POLNC

PY - 2016

SN - 19326203 (ISSN)

ST - Myc and Fgf are required for zebrafish neuromast hair cell regeneration

T2 - PLoS ONE

TI - Myc and Fgf are required for zebrafish neuromast hair cell regeneration

UR - https://www.scopus.com/pages/publications/84977505722?origin=resultslist

VL - 11

ID - 39866

ER -

TY - JOUR

AB - The inner ears of adult humans and other mammals possess a limited capacity for regenerating sensory hair cells, which can lead to permanent auditory and vestibular deficits. During development and regeneration, undifferentiated supporting cells within inner ear sensory epithelia can self-renew and give rise to new hair cells; however, these otic progenitors become depleted postnatally. Therefore, reprogramming differentiated supporting cells into otic progenitors is a potential strategy for restoring regenerative potential to the ear. Transient expression of the induced pluripotency transcription factors, Oct3/4, Klf4, Sox2, and c-Myc reprograms fibroblasts into neural progenitors under neural-promoting culture conditions, so as a first step, we explored whether ectopic expression of these factors can reverse supporting cell quiescence in whole organ cultures of adult mouse utricles. Co-infection of utricles with adenoviral vectors separately encoding Oct3/4, Klf4, Sox2, and the degradation-resistant T58A mutant of c-Myc (c-MycT58A) triggered significant levels of supporting cell S-phase entry as assessed by continuous BrdU labeling. Of the four factors, c-MycT58A alone was both necessary and sufficient for the proliferative response. The number of BrdU-labeled cells plateaued between 5-7 days after infection, and then decreased ~60% by 3 weeks, as many cycling cells appeared to enter apoptosis. Switching to differentiation-promoting culture medium at 5 days after ectopic expression of c-MycT58A temporarily attenuated the loss of BrdU-labeled cells and accompanied a very modest but significant expansion of the sensory epithelium. A small number of the proliferating cells in these cultures labeled for the hair cell marker, myosin VIIA, suggesting they had begun differentiating towards a hair cell fate. The results indicate that ectopic expression of c-MycT58A in combination with methods for promoting cell survival and differentiation may restore regenerative potential to supporting cells within the adult mammalian inner ear. © 2012 Burns et al.

AD - Wake Forest Institute for Regenerative Medicine, Wake Forest School of Medicine, Winston-Salem, NC, United States

AU - Burns, J. C.

AU - Yoo, J. J.

AU - Atala, A.

AU - Jackson, J. D.

C1 - Scopus

C2 - 23119091

C7 - e48704

DB - Scopus

DO - 10.1371/journal.pone.0048704

IS - 10

J2 - PLoS ONE

KW - Animals

Cell Differentiation

Cell Proliferation

Cell Survival

Gene Transfer Techniques

Hair Cells, Auditory

Humans

Immunohistochemistry

Kruppel-Like Transcription Factors

Mice

Microscopy, Confocal

Mitosis

Mutation

Myosins

Octamer Transcription Factor-3

Organ Culture Techniques

Proto-Oncogene Proteins c-myc

S Phase

Saccule and Utricle

SOXB1 Transcription Factors

Time Factors

Mammalia

adenovirus vector

kruppel like factor 4

Myc protein

myosin VIIa

octamer transcription factor 4

transcription factor Sox2

adenovirus infection

animal cell

animal experiment

animal model

animal tissue

apoptosis

article

cell cycle S phase

cell fate

cell regeneration

concentration (parameters)

controlled study

embryo

female

genetic transduction

hair cell

immunocytochemistry

in vitro study

male

mouse

nerve cell differentiation

neural stem cell

neuroepithelium

nonhuman

oncogene c myc

protein expression

quantitative analysis

regenerative ability

utricle

viral gene delivery system

viral gene therapy

LA - English

M3 - Article

N1 - Export Date: 21 May 2026; Cited By: 33; Correspondence Address: J. C. Burns; Wake Forest Institute for Regenerative Medicine, Wake Forest School of Medicine, Winston-Salem, NC, United States; email: joburns@wakehealth.edu

PY - 2012

SN - 19326203 (ISSN)

ST - MYC Gene Delivery to Adult Mouse Utricles Stimulates Proliferation of Postmitotic Supporting Cells In Vitro

T2 - PLoS ONE

TI - MYC Gene Delivery to Adult Mouse Utricles Stimulates Proliferation of Postmitotic Supporting Cells In Vitro

UR - https://www.scopus.com/pages/publications/84868130499?origin=resultslist

VL - 7

ID - 39863

ER -

TY - JOUR

AB - Current treatments for hearing loss, the most common neurosensory disorder, do not restore perfect hearing. Regeneration of lost organ of Corti hair cells through forced cell cycle re-entry of supporting cells or through manipulation of stem cells, both avenues towards a permanent cure, require a more complete understanding of normal inner ear development, specifically the balance of proliferation and differentiation required to form and to maintain hair cells. Direct successful alterations to the cell cycle result in cell death whereas regulation of upstream genes is insufficient to permanently alter cell cycle dynamics. The Myc gene family is uniquely situated to synergize upstream pathways into downstream cell cycle control. There are three Mycs that are embedded within the Myc/Max/Mad network to regulate proliferation. The function of the two ear expressed Mycs, N-Myc and L-Myc were unknown less than two years ago and their therapeutic potentials remain speculative. In this review, we discuss the roles the Mycs play in the body and what led us to choose them to be our candidate gene for inner ear therapies. We will summarize the recently published work describing the early and late effects of N-Myc and L-Myc on hair cell formation and maintenance. Lastly, we detail the translational significance of our findings and what future work must be performed to make the ultimate hearing aid: the regeneration of the organ of Corti.

AD - Department of Biology, 143 Biology Building, University of Iowa, Iowa City, IA 52242, USA. benjamin-kopecky@uiowa.edu.

Department of Biology, 143 Biology Building, University of Iowa, Iowa City, IA 52242, USA. bernd-fritzsch@uiowa.edu.

AN - 24710525

AU - Kopecky, B.

AU - Fritzsch, B.

C1 - PubMed

C2 - PMC3901154

DA - Sep 25

DO - 10.3390/cells1040667

DP - NLM

ET - 2012/01/01

IS - 4

LA - eng

N1 - 2073-4409

Kopecky, Benjamin

Fritzsch, Bernd

P30 DC010362/DC/NIDCD NIH HHS/United States

T32 GM007337/GM/NIGMS NIH HHS/United States

UL1 RR024979/RR/NCRR NIH HHS/United States

Journal Article

Switzerland

Cells. 2012 Sep 25;1(4):667-98. doi: 10.3390/cells1040667.

PY - 2012

SN - 2073-4409 (Print)

2073-4409

SP - 667-98

ST - The myc road to hearing restoration

T2 - Cells

TI - The myc road to hearing restoration

VL - 1

ID - 39292

ER -

TY - JOUR

AB - Sensorineural hearing loss results from damage to the hair cells of the organ of Corti and is irreversible in mammals. While hair cell regeneration may prove to be the ideal therapy after hearing loss, prevention of initial hair cell loss could provide even more benefit at a lower cost. Previous studies have shown that the deletion of Atoh1 results in embryonic loss of hair cells while the absence of Barhl1, Gfi1, and Pou4f3 leads to the progressive loss of hair cells in newborn mice. We recently reported that in the early embryonic absence of N-Myc (using Pax2-Cre), hair cells in the organ of Corti develop and remain until at least seven days after birth, with subsequent progressive loss. Thus, N-Myc plays a role in hair cell viability; however, it is unclear if this is due to its early expression in hair cell precursors and throughout the growing otocyst as it functions through proliferation or its late expression exclusively in differentiated hair cells. Furthermore, the related family member L-Myc is mostly co-expressed in the ear, including in differentiated hair cells, but its function has not been studied and could be partially redundant to N-Myc. To test for a long-term function of the Mycs in differentiated hair cells, we generated nine unique genotypes knocking out N-Myc and/or L-Myc after initial formation of hair cells using the well-characterized Atoh1-Cre. We tested functionality of the auditory and vestibular systems at both P21 and four months of age and under the administration of the ototoxic drug cisplatin. We conclude that neither N-Myc nor L-Myc is likely to play important roles in long-term hair cell maintenance. Therefore, it is likely that the late-onset loss of hair cells resulting from early deletion of the Mycs leads to an unsustainable developmental defect. © 2012 Elsevier B.V.

AD - University of Iowa, Department of Biology, Iowa City, IA 52242, United States

University of Iowa, Carver College of Medicine, Medical Scientist Training Program, Iowa City, IA 52242, United States

University of Iowa, Department of Statistics and Actuarial Science, Iowa City, IA 52242, United States

AU - Kopecky, B. J.

AU - Decook, R.

AU - Fritzsch, B.

C1 - Scopus

C2 - 23022312

DB - Scopus

DO - 10.1016/j.brainres.2012.09.027

J2 - Brain Res.

KW - Hair Cell

Hearing

L-Myc

N-Myc

Prevention

Regeneration

Animals

Embryo, Mammalian

Hair Cells, Auditory, Inner

Immunohistochemistry

In Situ Hybridization

Mice

Mice, Knockout

Microscopy, Confocal

Organ of Corti

Proto-Oncogene Proteins c-myc

Mammalia

Mus

cisplatin

article

auditory system

cell differentiation

cell maturation

cell viability

Corti organ

gene deletion

gene expression

gene function

genotype

L Myc gene

oncogene c myb

oncogene myc

priority journal

vestibular system

LA - English

M3 - Article

N1 - Export Date: 21 May 2026; Cited By: 14; Correspondence Address: B.J. Kopecky; University of Iowa, Department of Biology, Iowa City, IA 52242, United States; email: benjamin-kopecky@uiowa.edu; CODEN: BRREA

PY - 2012

SN - 18726240 (ISSN)

SP - 1-14

ST - N-Myc and L-Myc are essential for hair cell formation but not maintenance

T2 - Brain Research

TI - N-Myc and L-Myc are essential for hair cell formation but not maintenance

UR - https://www.scopus.com/pages/publications/84867864586?origin=resultslist

VL - 1484

ID - 39853

ER -

TY - JOUR

AB - BACKGROUND AND OBJECTIVES: Regeneration or replacement of damaged hair cells and neurons in the cochlea might be an ideal treatment for sensorineural hearing loss (SNHL). The purpose of the present study was to investigate whether mesenchymal stem cells (MSCs), derived from the bone marrow of rats, could differentiate into auditory hair cells and neurons. MATERIALS AND METHODS: The centrifuge gradient method was used to isolate MSCs from the bone marrow of rats. To confirm whether bone marrow-derived MSCs can differentiate into neuronal cells, culture medium with glial cell-derived neurotrophic factor, brain-derived neurotrophic factor and neurotrophin-3 for 14 days. In addition, immunofluorescence staining and RT-PCR were performed for characterization of the neurospheres and differentiated cells from 7 and 14 day cultures. RESULTS: The results showed that MSCs could differentiate into neuron-positive and hair cell-positive cells, using different compositions of growth factors. And RT-PCR result was identified high or low of gene expression all these differentiated cells. CONCLUSIONS: Rat bone marrow-derived MSCs differentiated into neuronal progenitor cells. These cells might be useful for the treatment of SNHL.

AD - Department of Otolaryngology-HNS, The Catholic University of Korea College of Medicine, Seoul, Korea.

Department of Surgery, The Catholic University of Korea College of Medicine, Seoul, Korea.

AN - 24653871

AU - Lee, J. H.

AU - Kang, W. K.

AU - Seo, J. H.

AU - Choi, M. Y.

AU - Lee, Y. H.

AU - Kim, H. M.

AU - Park, K. H.

C1 - PubMed

C2 - PMC3936568

DA - Sep

DO - 10.7874/kja.2012.16.2.47

DP - NLM

ET - 2012/09/01

IS - 2

KW - Bone marrow

Differentiation

Hair cells

Mesenchymal stem cells

Neurons

LA - eng

N1 - 2093-3797

Lee, Jae-Hong

Kang, Won Kyung

Seo, Jae-Hyun

Choi, Mi-Yung

Lee, Yang Hyun

Kim, Hyo Min

Park, Kyoung-Ho

Journal Article

Korea (South)

Korean J Audiol. 2012 Sep;16(2):47-53. doi: 10.7874/kja.2012.16.2.47. Epub 2012 Sep 20.

PY - 2012

SN - 2092-9862 (Print)

2092-9862

SP - 47-53

ST - Neural differentiation of bone marrow-derived mesenchymal stem cells: applicability for inner ear therapy

T2 - Korean J Audiol

TI - Neural differentiation of bone marrow-derived mesenchymal stem cells: applicability for inner ear therapy

VL - 16

ID - 39485

ER -

TY - JOUR

AB - Neurotrophin-3 (Ntf3) and brain derived neurotrophic factor (Bdnf) are critical for sensory neuron survival and the establishment of neuronal projections to sensory epithelia in the embryonic inner ear, but their postnatal functions remain poorly understood. Using cell-specific inducible gene recombination in mice we found that, in the postnatal inner ear, Bbnf and Ntf3 are required for the formation and maintenance of hair cell ribbon synapses in the vestibular and cochlear epithelia, respectively. We also show that supporting cells in these epithelia are the key endogenous source of the neurotrophins. Using a new hair cell CreERT 33 line with mosaic expression, we also found that Ntf3’s effect on cochlear synaptogenesis is highly localized. Moreover, supporting cell-derived Ntf3, but not Bbnf, promoted recovery of cochlear function and ribbon synapse regeneration after acoustic trauma. These results indicate that glial-derived neurotrophins play critical roles in inner ear synapse density and synaptic regeneration after injury. © 2014, eLife Sciences Publications Ltd. All rights reserved.

AD - F.M. Kirby Neurobiology Center, Boston Children’s Hospital, Boston, 02115, MA, United States

Department of Neurology, Harvard Medical School, Boston, 02115, MA, United States

Kresge Hearing Research Institute, Department of Otolaryngology – Head and Neck Surgery, University of Michigan, Ann Arbor, 48109, MI, United States

Department of Otology and Laryngology, Harvard Medical School, Boston, 02115, MA, United States

Eaton Peabody Laboratories, Massachusetts Eye and Ear Infirmary, Boston, 02114, MA, United States

AU - Wan, G.

AU - Gómez-Casati, M. E.

AU - Gigliello, A. R.

AU - Charles Liberman, M.

AU - Corfas, G.

C1 - Scopus

C2 - 25329343

C7 - e03564

DB - Scopus

DO - 10.7554/eLife.03564

J2 - eLife

KW - Animals

Animals, Newborn

Brain-Derived Neurotrophic Factor

Gene Expression Regulation

Hair Cells, Auditory

Hair Cells, Vestibular

Hearing Loss, Noise-Induced

Mice

Mice, Transgenic

Neurogenesis

Neuroglia

Neurotrophin 3

Regeneration

Synapses

Synaptic Transmission

Mus

brain derived neurotrophic factor

tamoxifen

animal experiment

animal model

animal tissue

Article

auditory response

auditory threshold

cochlea

controlled study

frequency analysis

gene expression

immunohistochemistry

inner ear

mouse

newborn

noise injury

nonhuman

reverse transcription polymerase chain reaction

RNA isolation

synapse

synaptogenesis

animal

genetics

glia

hair cell

metabolism

nervous system development

pathology

transgenic mouse

LA - English

M3 - Article

N1 - Export Date: 21 May 2026; Cited By: 224; Correspondence Address: G. Corfas; Kresge Hearing Research Institute, University of Michigan, Ann Arbor, Medical Sciences I Building, 1150 West Medical Center Drive, 48109-5616, United States; email: corfas@med.umich.edu

PY - 2014

SN - 2050084X (ISSN)

SP - 1-35

ST - Neurotrophin-3 regulates ribbon synapse density in the cochlea and induces synapse regeneration after acoustic trauma

T2 - eLife

TI - Neurotrophin-3 regulates ribbon synapse density in the cochlea and induces synapse regeneration after acoustic trauma

UR - https://www.scopus.com/pages/publications/84930654678?origin=resultslist

VL - 2014-October

ID - 39859

ER -

TY - JOUR

AB - Noise exposure is a major cause of hearing loss. Classical methods of studying protein involvement have provided a basis for understanding signaling pathways that mediate hearing loss and damage repair but do not lend themselves to studying large networks of proteins that are likely to increase or decrease during noise trauma. To address this issue, antibody microarrays were used to quantify the very early changes in protein expression in three distinct regions of the chinchilla cochlea 2h after exposure to a 0.5-8 kHz band of noise for 2h at 112 dB SPL. The noise exposure caused significant functional impairment 2h post-exposure which only partially recovered. Distortion product otoacoustic emissions were abolished 2h after the exposure, but at 4 weeks post-exposure, otoacoustic emissions were present, but still greatly depressed. Cochleograms obtained 4 weeks post-exposure demonstrated significant loss of outer hair cells in the basal 60% of the cochlea corresponding to frequencies in the noise spectrum. A comparative analysis of the very early (2h post-exposure) noise-induced proteomic changes indicated that the sensory epithelium, lateral wall and modiolus differ in their biological response to noise. Bioinformatic analysis of the cochlear protein profile using "The Database for Annotation, Visualization and Integrated Discovery 2008" (DAVID - http://david.abcc. ncifcrf.gov) revealed the initiation of the cell death process in sensory epithelium and modiolus. An increase in Fas and phosphorylation of FAK and p38/MAPK in the sensory epithelium suggest that noise-induced stress signals at the cell membrane are transmitted to the nucleus by Fas and focal adhesion signaling through the p38/MAPK signaling pathway. Up-regulation of downstream nuclear proteins E2F3 and WSTF in immunoblots and microarrays along with their immunolocalization in the outer hair cells supported the pivotal role of p38/MAPK signaling in the mechanism underlying noise-induced hearing loss.

AD - Center for Hearing and Deafness, University at Buffalo, the State University of New York, Buffalo, NY 14214, USA.

AN - 21871588

AU - Jamesdaniel, S.

AU - Hu, B.

AU - Kermany, M. H.

AU - Jiang, H.

AU - Ding, D.

AU - Coling, D.

AU - Salvi, R.

C1 - PubMed

C2 - PMC3225708

C6 - NIHMS319665

DA - Dec 21

DO - 10.1016/j.jprot.2011.08.007

DP - NLM

ET - 2011/08/30

IS - 2

KW - Animals

Chinchilla

Cochlea/injuries/*physiopathology

Focal Adhesion Protein-Tyrosine Kinases/metabolism

Hair Cells, Auditory, Outer/*metabolism/pathology

Hearing Loss, Noise-Induced/*physiopathology

Noise

Otoacoustic Emissions, Spontaneous/physiology

Proteomics

Signal Transduction/*physiology

fas Receptor/biosynthesis

p38 Mitogen-Activated Protein Kinases/*biosynthesis

LA - eng

N1 - 1876-7737

Jamesdaniel, Samson

Hu, Bohua

Kermany, Mohammad Habiby

Jiang, Haiyan

Ding, Dalian

Coling, Donald

Salvi, Richard

R03 DC010225/DC/NIDCD NIH HHS/United States

R01 DC010154/DC/NIDCD NIH HHS/United States

R01 DC009091/DC/NIDCD NIH HHS/United States

R01DC009091/DC/NIDCD NIH HHS/United States

R01 DC009219/DC/NIDCD NIH HHS/United States

R03DC010225/DC/NIDCD NIH HHS/United States

1R01DC009219/DC/NIDCD NIH HHS/United States

Journal Article

Research Support, N.I.H., Extramural

Netherlands

J Proteomics. 2011 Dec 21;75(2):410-24. doi: 10.1016/j.jprot.2011.08.007. Epub 2011 Aug 16.

PY - 2011

SN - 1874-3919 (Print)

1874-3919

SP - 410-24

ST - Noise induced changes in the expression of p38/MAPK signaling proteins in the sensory epithelium of the inner ear

T2 - J Proteomics

TI - Noise induced changes in the expression of p38/MAPK signaling proteins in the sensory epithelium of the inner ear

VL - 75

ID - 39821

ER -

TY - JOUR

AB - The synapse between the inner hair cells (IHCs) and the spiral ganglion neurons (SGNs) in mammalian cochleae is characterized as having presynaptic ribbons and therefore is called ribbon synapse. The special molecular organization is reviewed in this chapter in association with the functional feature of this synapse in signal processing. This is followed by the review on noise-induced damage to this synapse with a focus on recent reports in animal models in which the effect of brief noise exposures is observed without causing significant permanent threshold shift (PTS). In this regard, the potential mechanism of the synaptic damage by noise and the impact of this damage on hearing are summarized to clarify the concept of noise-induced hidden hearing loss, which is defined as the functional deficits in hearing without threshold elevation. A controversial issue is addressed in this review as whether the disrupted synapses can be regenerated. Moreover, the review summarizes the work of therapeutic research to protect the synapses or to promote the regeneration of the synapse after initial disruption. Lastly, several unresolved issues are raised for investigation in the future.

AD - School of Communication Science and Disorders, Dalhousie University, Halifax, NS, Canada. jian.wang@dal.ca.

Otolaryngology Research Institute, 6th Affiliated Hospital, Shanghai Jiao-Tong University, Shanghai, China.

Department of Physiology, Medical College of Southeast University, Nanjing, China.

AN - 30915700

AU - Wang, J.

AU - Yin, S.

AU - Chen, H.

AU - Shi, L.

C1 - PubMed

DO - 10.1007/978-981-13-6123-4_3

DP - NLM

ET - 2019/03/28

KW - Animals

Cochlea/*pathology

*Hearing Loss, Noise-Induced

Noise

Regeneration

Synapses/*pathology

Neurotrophins

Noise-induced hidden hearing loss

Ribbon synapses

Synapse regeneration

LA - eng

N1 - Wang, Jian

Yin, Shankai

Chen, Hengchao

Shi, Lijuan

Journal Article

Review

United States

Adv Exp Med Biol. 2019;1130:37-57. doi: 10.1007/978-981-13-6123-4_3.

PY - 2019

SN - 0065-2598 (Print)

0065-2598

SP - 37-57

ST - Noise-Induced Cochlear Synaptopathy and Ribbon Synapse Regeneration: Repair Process and Therapeutic Target

T2 - Adv Exp Med Biol

TI - Noise-Induced Cochlear Synaptopathy and Ribbon Synapse Regeneration: Repair Process and Therapeutic Target

VL - 1130

ID - 38873

ER -

TY - JOUR

AB - PURPOSE OF THE REVIEW: This article presents research findings from two invertebrate model systems with potential to advance both the understanding of noise-induced hearing loss mechanisms and the development of putative therapies to reduce human noise damage. RECENT FINDINGS: Work on sea anemone hair bundles, which resemble auditory hair cells, has revealed secretions that exhibit astonishing healing properties not only for damaged hair bundles, but also for vertebrate lateral line neuromasts. We present progress on identifying functional components of the secretions, and their mechanisms of repair. The second model, the Johnston's organ in Drosophila, is also genetically homologous to hair cells and shows noise-induced hearing loss similar to vertebrates. Drosophila offers genetic and molecular insight into noise sensitivity and pathways that can be manipulated to reduce stress and damage from noise. SUMMARY: Using the comparative approach is a productive avenue to understanding basic mechanisms, in this case cellular responses to noise trauma. Expanding study of these systems may accelerate identification of strategies to reduce or prevent noise damage in the human ear.

AD - Department of Biology, The University of Iowa, Iowa City, Iowa, USA.

AN - 25111054

AU - Christie, K. W.

AU - Eberl, D. F.

C1 - PubMed

C2 - PMC4289668

C6 - NIHMS628892

DA - Oct

DO - 10.1097/moo.0000000000000086

DP - NLM

ET - 2014/08/12

IS - 5

KW - Anatomy, Comparative

Animals

Disease Models, Animal

Drosophila

Hair Cells, Auditory/physiology

*Hearing Loss, Noise-Induced/physiopathology

Mechanoreceptors/physiology

*Models, Animal

Sea Anemones

LA - eng

N1 - 1531-6998

Christie, Kevin W

Eberl, Daniel F

P30 DC010362/DC/NIDCD NIH HHS/United States

R21 DC011397/DC/NIDCD NIH HHS/United States

Journal Article

Research Support, N.I.H., Extramural

Research Support, Non-U.S. Gov't

Review

United States

Curr Opin Otolaryngol Head Neck Surg. 2014 Oct;22(5):374-83. doi: 10.1097/MOO.0000000000000086.

PY - 2014

SN - 1068-9508 (Print)

1068-9508

SP - 374-83

ST - Noise-induced hearing loss: new animal models

T2 - Curr Opin Otolaryngol Head Neck Surg

TI - Noise-induced hearing loss: new animal models

VL - 22

ID - 39430

ER -

TY - JOUR

AB - Noise-induced hearing loss is one of the most common forms of hearing loss in adults and also one of the most common occupational diseases. Extensive previous work has shown that the highly sensitive synapses of the inner hair cells (IHCs) may be the first target for irreparable damage and permanent loss in the noise-exposed cochlea, more precisely in the cochlear base. However, how such synaptic loss affects the synaptic physiology of the IHCs in this particularly vulnerable part of the cochlea has not yet been investigated. To address this question, we exposed 3-4-week-old C57BL/6J mice to 8-16 kHz noise for 2 h under isoflurane anesthesia. We then employed hearing measurements, immunohistochemistry and patch-clamp to assess IHC synaptic function. Two noise sound pressure levels (SPLs) were used to evoke acute hearing threshold elevations with different levels of recovery 2 weeks post-exposure. Regardless of noise intensity, the exposure resulted in a loss of approximately 25-36% of ribbon synapses in the basal portions of the cochlea that persisted 2 weeks after exposure. Perforated patch-clamp recordings were made in the IHCs of the basal regions of the cochlea where the greatest synaptic losses were observed. Depolarization-evoked calcium currents in IHCs 2 weeks after exposure were slightly but not significantly smaller as compared to controls from age-matched non-exposed animals. Exocytic changes monitored as changes in membrane capacitance did not follow that trend and remained similar to controls despite significant loss of ribbons, likely reflecting increased exocytosis at the remaining synapses. Additionally, we report for the first time that acute application of isoflurane reduces IHC calcium currents, which may have implications for noise-induced IHC synaptic loss.

AD - Experimental Otology Group, InnerEarLab, Department of Otolaryngology, University Medical Center Göttingen, Göttingen, Germany.

Auditory Neuroscience Group, Max Planck Institute for Multidisciplinary Sciences, Göttingen, Germany.

Auditory Systems Physiology Group, Institute for Auditory Neuroscience, InnerEarLab, University Medical Center Göttingen, Göttingen, Germany.

Collaborative Research Center 889, University of Göttingen, Göttingen, Germany.

AN - 39839350

AU - Oestreicher, D.

AU - Malpede, A. M.

AU - Reitmeier, A.

AU - Bräuer, C. P.

AU - Schoch, L.

AU - Strenzke, N.

AU - Pangrsic, T.

C1 - PubMed

C2 - PMC11747652

DO - 10.3389/fncel.2024.1523978

DP - NLM

ET - 2025/01/22

KW - calcium channel

inner hair cell

isoflurane

noise trauma

ribbon synapse

commercial or financial relationships that could be construed as a potential

conflict of interest.

LA - eng

N1 - 1662-5102

Oestreicher, David

Malpede, Alfonso Mauro

Reitmeier, Annalena

Bräuer, Carolin Paula

Schoch, Laura

Strenzke, Nicola

Pangrsic, Tina

Journal Article

Switzerland

Front Cell Neurosci. 2025 Jan 7;18:1523978. doi: 10.3389/fncel.2024.1523978. eCollection 2024.

PY - 2024

SN - 1662-5102 (Print)

1662-5102

SP - 1523978

ST - Noise-induced ribbon synapse loss in the mouse basal cochlear region does not reduce inner hair cell exocytosis

T2 - Front Cell Neurosci

TI - Noise-induced ribbon synapse loss in the mouse basal cochlear region does not reduce inner hair cell exocytosis

VL - 18

ID - 39826

ER -

TY - JOUR

AB - In the inner ear, Notch signaling has been shown to have two key developmental roles. The first occurs early in otic development and defines the prosensory domains that will develop into the six sensory organs of the inner ear. The second role occurs later in development and establishes the mosaic-like pattern of the mechanosensory hair cells and their surrounding support cells through the more well-characterized process of lateral inhibition. These dual developmental roles have inspired several different strategies to regenerate hair cells in the mature inner ear organs. These strategies include (1) modulation of Notch signaling in inner ear stem cells in order to increase hair cell yield, (2) activation of Notch signaling in order to promote the formation of ectopic sensory regions in normally non-sensory regions within the inner ear, and (3) inhibition of Notch signaling to disrupt lateral inhibition and allow support cells to transdifferentiate into hair cells. In this review, we summarize some of the promising studies that have used these various strategies for hair cell regeneration through modulation of Notch signaling and some of the challenges that remain in developing therapies based on hair cell regeneration.

AD - Department of Biological Structure, University of Washington, Seattle, Washington 98195 ; Graduate Program in Neurobiology and Behavior, University of Washington, Seattle, Washington 98195.

Department of Biological Structure, University of Washington, Seattle, Washington 98195 ; Institute for Stem Cells and Regenerative Medicine, University of Washington, Seattle, Washington 98109, USA.

AN - 25328289

AU - Slowik, A. D.

AU - Bermingham-McDonogh, O.

C1 - PubMed

C2 - PMC4199338

C6 - NIHMS599613

DP - NLM

ET - 2013/01/01

KW - Delta

Jagged

balance

cochlea

hearing

inner ear

sensory

vestibular

γ-secretase

interests.

LA - eng

N1 - Slowik, Amber D

Bermingham-McDonogh, Olivia

R01 DC009991/DC/NIDCD NIH HHS/United States

R21 DC010862/DC/NIDCD NIH HHS/United States

T32 GM007108/GM/NIGMS NIH HHS/United States

T32 GM007270/GM/NIGMS NIH HHS/United States

Journal Article

India

Trends Dev Biol. 2013;7:73-89.

PY - 2013

SN - 0972-8422 (Print)

0972-8422

SP - 73-89

ST - Notch signaling in mammalian hair cell regeneration

T2 - Trends Dev Biol

TI - Notch signaling in mammalian hair cell regeneration

VL - 7

ID - 39094

ER -

TY - JOUR

AB - The study of an adult mammalian auditory system, such as regeneration, has been hampered by the lack of an in vitro system in which hypotheses can be tested efficiently. This is primarily due to the fact that the adult inner ear is encased in the toughest bone of the body, whereas its removal leads to the death of the sensory epithelium in culture. We hypothesized that we could take advantage of the integral cochlear structure to maintain the overall inner ear architecture and improve sensory epithelium survival in culture. We showed that by culturing adult mouse cochlea with the (surrounding) bone intact, the supporting cells (SCs) survived and almost all hair cells (HCs) degenerated. To evaluate the utility of the explant culture system, we demonstrated that the overexpression of Atoh1, an HC fate-determining factor, is sufficient to induce transdifferentiation of adult SCs to HC-like cells (HCLCs). Transdifferentiation-derived HCLCs resemble developmentally young HCs and are able to attract adult ganglion neurites. Furthermore, using a damage model, we showed that degenerated adult ganglions respond to regenerated HCLCs by directional neurite outgrowth that leads to HCLC-neuron contacts, strongly supporting the intrinsic properties of the HCLCs in establishing HCLC-neuron connections. The adult whole cochlear explant culture is suitable for diverse studies of the adult inner ear including regeneration, HC-neuron pathways, and inner ear drug screening.

AD - Department of Otolaryngology-Head and Neck Surgery, Graduate Program in Speech and Hearing Bioscience and Technology and Program in Neuroscience, Harvard Medical School, Boston, MA, United States.

Eaton-Peabody Laboratory, Massachusetts Eye and Ear Infirmary, Boston, MA, United States.

ENT Institute and Otorhinolaryngology Department of Eye & ENT Hospital, State Key Laboratory of Medical Neurobiology and MOE Frontiers Center for Brain Science, Fudan University, Shanghai, China.

Institutes of Biomedical Sciences, Fudan University, Shanghai, China.

AN - 35082601

AU - Li, W.

AU - Quan, Y.

AU - Huang, M.

AU - Wei, W.

AU - Shu, Y.

AU - Li, H.

AU - Chen, Z. Y.

C1 - PubMed

C2 - PMC8785685

DO - 10.3389/fnmol.2021.757831

DP - NLM

ET - 2022/01/28

KW - adult

cochlea

hair cell

novel model

regeneration

developing treatments for hearing loss including genome editing, inner ear

regeneration, novel delivery, and gene therapy. The remaining authors declare

that the research was conducted in the absence of any commercial or financial

relationships that could be construed as a potential conflict of interest.

LA - eng

N1 - 1662-5099

Li, Wenyan

Quan, Yizhou

Huang, Mingqian

Wei, Wei

Shu, Yilai

Li, Huawei

Chen, Zheng-Yi

Journal Article

Switzerland

Front Mol Neurosci. 2022 Jan 10;14:757831. doi: 10.3389/fnmol.2021.757831. eCollection 2021.

PY - 2021

SN - 1662-5099 (Print)

1662-5099

SP - 757831

ST - A Novel in vitro Model Delineating Hair Cell Regeneration and Neural Reinnervation in Adult Mouse Cochlea

T2 - Front Mol Neurosci

TI - A Novel in vitro Model Delineating Hair Cell Regeneration and Neural Reinnervation in Adult Mouse Cochlea

VL - 14

ID - 39065

ER -

TY - JOUR

AB - Sensorineural hearing loss (SNHL), a leading cause of disability worldwide, arises from damage to hair cells (HCs) or spiral ganglion neurons (SGNs) within the cochlea. Among its etiologies, auditory neuropathy (AN) is characterized by disrupted signal transmission due to SGN damage. Traditional interventions, such as hearing aids and cochlear implants, provide limited benefit in cases of AN, where neuronal damage impairs signal transduction to the brain. Emerging regenerative therapies, including cell replacement and gene delivery, hold potential to restore SGN function, but their application is limited by challenges in delivering therapeutic agents to cochlear targets. In this study, we developed a novel stereotaxic approach for minimally invasive, precise delivery of therapeutic agents to murine SGNs. Utilizing pre-determined coordinates, we successfully accessed the cochlea and SGNs. Immunohistochemistry confirmed accurate delivery and integration of therapeutic agents. Functional hearing assessments showed that the approach preserved HC function and demonstrated minimal adverse effects. This technique offers a scalable platform for advancing cell and gene therapies aimed at restoring auditory function in AN and other forms of SNHL.

AD - The Eaton-Peabody Laboratories, The Massachusetts Eye and Ear Department of Otolaryngology-Head and Neck Surgery, Boston, MA, United States.

University of Massachusetts Chan Medical School, Worcester, MA, United States.

Harvard Medical School Department of Otolaryngology-Head and Neck Surgery, Boston, MA, United States.

Department of Otolaryngology, UMass Memorial Medical Center, Worcester, MA, United States.

AN - 41561328

AU - Al-Asad, R. K.

AU - Luu, N. C.

AU - Edge, A. S.

AU - Kempfle, J. S.

C1 - PubMed

C2 - PMC12812712

DO - 10.3389/fneur.2025.1696492

DP - NLM

ET - 2026/01/21

KW - auditory neuropathy

hearing regeneration

sensorineural hearing loss

spiral ganglion neurons

stereotaxic surgery

commercial or financial relationships that could be construed as a potential

conflict of interest.

LA - eng

N1 - 1664-2295

Al-Asad, Reef K

Luu, Ngoc-Nhi C

Edge, Albert S

Kempfle, Judith S

Journal Article

Switzerland

Front Neurol. 2026 Jan 5;16:1696492. doi: 10.3389/fneur.2025.1696492. eCollection 2025.

PY - 2025

SN - 1664-2295 (Print)

1664-2295

SP - 1696492

ST - A novel minimally invasive stereotaxic technique to target inner ear neurons in the mouse

T2 - Front Neurol

TI - A novel minimally invasive stereotaxic technique to target inner ear neurons in the mouse

VL - 16

ID - 39307

ER -

TY - JOUR

AB - Different mutations of the OTOF gene, encoding for otoferlin protein expressed in the cochlear inner hair cells, induces a form of deafness that is the major cause of nonsyndromic recessive auditory neuropathy spectrum disorder in humans. We report the generation of the first large animal model of OTOF mutations using the CRISPR system associated with different Cas9 components (mRNA or protein) assisted by single strand oligodeoxynucleotides (ssODN) to induce homology-directed repair (HDR). Zygote microinjection was performed with two sgRNA targeting exon 5 and 6 associated to Cas9 mRNA or protein (RNP) at different concentrations in a mix with an ssODN template targeting HDR in exon 5 containing two STOP sequences. A total of 73 lambs were born, 13 showing indel mutations (17.8%), 8 of which (61.5%) had knock-in mutations by HDR. Higher concentrations of Cas9-RNP induced targeted mutations more effectively, but negatively affected embryo survival and pregnancy rate. This study reports by the first time the generation of OTOF disrupted sheep, which may allow better understanding and development of new therapies for human deafness related to genetic disorders. These results support the use of CRISPR/Cas system assisted by ssODN as an effective tool for gene editing in livestock.

AD - Instituto de Reproducción Animal Uruguay, Fundación IRAUy, Montevideo, Uruguay. menchaca.alejo@gmail.com.

Instituto de Reproducción Animal Uruguay, Fundación IRAUy, Montevideo, Uruguay.

Unidad de Animales Transgénicos y de Experimentación (UATE), Institut Pasteur de Montevideo, Montevideo, Uruguay.

Inserm, Centre de Recherche en Transplantation et Immunologie, UMR 1064, F-44000, Nantes, France.

Transgenesis Rat ImmunoPhenomic facility (TRIP), F-44000, Nantes, France.

GenoCellEdit facility, F-44000, Nantes, France.

Inserm, Centre de Recherche en Transplantation et Immunologie, UMR 1064, F-44000, Nantes, France. ianegon@nantes.inserm.fr.

Transgenesis Rat ImmunoPhenomic facility (TRIP), F-44000, Nantes, France. ianegon@nantes.inserm.fr.

GenoCellEdit facility, F-44000, Nantes, France. ianegon@nantes.inserm.fr.

Unidad de Animales Transgénicos y de Experimentación (UATE), Institut Pasteur de Montevideo, Montevideo, Uruguay. crispo@pasteur.edu.uy.

AN - 32265471

AU - Menchaca, A.

AU - Dos Santos-Neto, P. C.

AU - Souza-Neves, M.

AU - Cuadro, F.

AU - Mulet, A. P.

AU - Tesson, L.

AU - Chenouard, V.

AU - Guiffès, A.

AU - Heslan, J. M.

AU - Gantier, M.

AU - Anegón, I.

AU - Crispo, M.

C1 - PubMed

C2 - PMC7138848

DA - Apr 7

DO - 10.1038/s41598-020-62879-y

DP - NLM

ET - 2020/04/09

IS - 1

KW - Animals

*CRISPR-Cas Systems

Clustered Regularly Interspaced Short Palindromic Repeats

Female

Gene Editing/*methods

Male

Membrane Proteins/*genetics

Microinjections

Mutation

Oligodeoxyribonucleotides/*genetics

Recombinational DNA Repair

Sheep/embryology/*genetics

LA - eng

N1 - 2045-2322

Menchaca, A

Orcid: 0000-0002-2494-9574

Dos Santos-Neto, P C

Souza-Neves, M

Cuadro, F

Mulet, A P

Tesson, L

Chenouard, V

Guiffès, A

Heslan, J M

Gantier, M

Anegón, I

Crispo, M

Journal Article

Research Support, Non-U.S. Gov't

England

Sci Rep. 2020 Apr 7;10(1):5995. doi: 10.1038/s41598-020-62879-y.

PY - 2020

SN - 2045-2322

SP - 5995

ST - Otoferlin gene editing in sheep via CRISPR-assisted ssODN-mediated Homology Directed Repair

T2 - Sci Rep

TI - Otoferlin gene editing in sheep via CRISPR-assisted ssODN-mediated Homology Directed Repair

VL - 10

ID - 39630

ER -

TY - JOUR

AB - Sensorineural hearing loss is a caused by the loss of the cochlear hair cells with the consequent deafferentation of spiral ganglion neurons. Humans do not show endogenous cellular regeneration in the inner ear and there is no exogenous therapy that allows the replacement of the damaged hair cells. Currently, treatment is based on the use of hearing aids and cochlear implants that present different outcomes, some difficulties in auditory discrimination and a limited useful life. More advanced technology is hindered by the functional capacity of the remaining spiral ganglion neurons. The latest advances with stem cell therapy and cellular reprogramming have developed several possibilities to induce endogenous regeneration or stem cell transplantation to replace damaged inner ear hair cells and restore hearing function. With further knowledge of the cellular and molecular biology of the inner ear and its embryonic development, it will be possible to use induced stem cells as in vitro models of disease and as replacement cellular therapy. Investigation in this area is focused on generating cellular therapy with clinical use for the treatment of profound sensorineural hearing loss.

AD - Grupo de Otología y Otoneurología CTS495, Área de Medicina Genómica-Centro de Genómica e Investigación Oncológica-Pfizer/Universidad de Granada/Junta de Andalucía (GENYO), Granada, España; Servicio de Otorrinolaringología, Hospital Universitario Granada, Granada, España.

Grupo de Otología y Otoneurología CTS495, Área de Medicina Genómica-Centro de Genómica e Investigación Oncológica-Pfizer/Universidad de Granada/Junta de Andalucía (GENYO), Granada, España.

Grupo de Otología y Otoneurología CTS495, Área de Medicina Genómica-Centro de Genómica e Investigación Oncológica-Pfizer/Universidad de Granada/Junta de Andalucía (GENYO), Granada, España; Departamento de Otorrinolaringología, Hospital de Poniente, El Ejido, Almería, España. Electronic address: antonio.lopezescamez@genyo.es.

AN - 25459416

AU - Almeida-Branco, M. S.

AU - Cabrera, S.

AU - Lopez-Escamez, J. A.

C1 - PubMed

DA - Sep-Oct

DO - 10.1016/j.otorri.2014.07.009

DP - NLM

ET - 2014/12/03

IS - 5

KW - Cell Self Renewal

*Cellular Reprogramming Techniques

Electric Stimulation Therapy

Hair Cells, Auditory, Inner/pathology

Hearing Loss, Sensorineural/*surgery

Humans

Nerve Growth Factors/physiology

Neural Stem Cells/cytology

Organ of Corti/physiology

Regeneration

Spiral Ganglion/pathology

Stem Cell Transplantation/*methods

Células madre

Gene therapy

Hipoacusia neurosensorial

Inner ear regeneration

Neuronas del ganglio espiral

Regeneración oído interno

Sensorineural hearing loss

Spinal ganglion neurons

Stem cells

Terapia génica

LA - eng

spa

N1 - 1988-3013

Almeida-Branco, Mario S

Cabrera, Sonia

Lopez-Escamez, Jose A

Journal Article

Research Support, Non-U.S. Gov't

Review

Spain

Acta Otorrinolaringol Esp. 2015 Sep-Oct;66(5):286-95. doi: 10.1016/j.otorri.2014.07.009. Epub 2014 Nov 20.

PY - 2015

SN - 0001-6519

SP - 286-95

ST - Perspectives for the treatment of sensorineural hearing loss by cellular regeneration of the inner ear

T2 - Acta Otorrinolaringol Esp

TI - Perspectives for the treatment of sensorineural hearing loss by cellular regeneration of the inner ear

VL - 66

ID - 38921

ER -

TY - JOUR

AB - In a previous study, we observed spontaneous restoration of vestibular function in young adult rodents following excitotoxic injury of the neuronal connections within vestibular endorgans. The functional restoration was supported by a repair of synaptic contacts between hair cells and primary vestibular neurons. This process was observed in 2/3 of the animals studied and occurred within 5 days following the synaptic damage. To assess whether repair capacity is a fundamental trait of vestibular endorgans and to decipher the cellular mechanisms supporting such a repair process, we studied the neuronal regeneration and synaptogenesis in co-cultures of vestibular epithelia and Scarpa's ganglion from young and adult rodents. We demonstrate that, under specific culture conditions, primary vestibular neurons from young mice or rats exhibit robust ability to regenerate nervous processes. When co-cultured with vestibular epithelia, primary vestibular neurons were able to establish de novo contacts with hair cells. Under the present paradigm, these contacts displayed morphological features of immature synaptic contacts. Preliminary observations using co-cultures of adult rodents suggest that this reparative capacity remained in older mice although to a lesser extent. Identifying the basic mechanisms underlying the repair process may provide a basis for novel therapeutic strategies to restore mature and functional vestibular synaptic contacts following damage or loss.

AD - INSERM U1051, Institute for Neurosciences Montpellier, France.

AN - 22685444

AU - Travo, C.

AU - Gaboyard-Niay, S.

AU - Chabbert, C.

C1 - PubMed

C2 - PMC3368229

DO - 10.3389/fneur.2012.00091

DP - NLM

ET - 2012/06/12

KW - plasticity

primary neurons

repair

sensory epithelia

synapse

vestibule

LA - eng

N1 - 1664-2295

Travo, Cécile

Gaboyard-Niay, Sophie

Chabbert, Christian

Journal Article

Switzerland

Front Neurol. 2012 Jun 6;3:91. doi: 10.3389/fneur.2012.00091. eCollection 2012.

PY - 2012

SN - 1664-2295

SP - 91

ST - Plasticity of Scarpa's Ganglion Neurons as a Possible Basis for Functional Restoration within Vestibular Endorgans

T2 - Front Neurol

TI - Plasticity of Scarpa's Ganglion Neurons as a Possible Basis for Functional Restoration within Vestibular Endorgans

VL - 3

ID - 39252

ER -

TY - JOUR

AB - miRNA-183 family, in normal biology, is expressed in a harmonious and stable manner in the neurosensory organs and cells. Studies have also shown that miRNA-183 family, in different pathways, affects the neurosensory development, maintenance, survival and function. In addition, it has potential neuroprotective effects in response to neurosensory destructive stimulations. miRNA-96 mutation causes hereditary deafness in humans and mice, and therefore affects the inner ear activity and its maintenance. Certain roles have been identified for miR-96 in the maintenance and function of the inner ear. The comparison of the target genes of family-183 in transcriptomes of newborn and adult hair cells shows that hundreds of target genes in this family may affect development and maintenance of the ears. Identifying the genes that are regulated by miRNA-183 family provides researchers with important information about the complex development and environmental regulation of the inner ear, and can offer new approaches to the maintenance and regeneration of hair cells and auditory nerve.

AD - Research Center for Molecular Medicine, Hamadan University of Medical Sciences, Hamadan, Iran.

Dept. of Genetics and Molecular Medicine, Hamadan University of Medical Sciences, Hamadan, Iran.

Cellular and Molecular Research Center, Shahrekord University of Medical Sciences, Sharekord, Iran.

AN - 29937838

AU - Mahmoodian-Sani, M. R.

AU - Mehri-Ghahfarrokhi, A.

C1 - PubMed

C2 - PMC5963458

DA - Jun

DO - 10.1016/j.joto.2017.03.003

DP - NLM

ET - 2018/06/26

IS - 2

KW - Hair cell

Hearing loss

Inner ear

miRNA

miRNA-183 family

LA - eng

N1 - 2524-1753

Mahmoodian-Sani, Mohammad-Reza

Mehri-Ghahfarrokhi, Ameneh

Journal Article

Review

China

J Otol. 2017 Jun;12(2):55-61. doi: 10.1016/j.joto.2017.03.003. Epub 2017 May 3.

PY - 2017

SN - 1672-2930 (Print)

1672-2930

SP - 55-61

ST - The potential of miR-183 family expression in inner ear for regeneration, treatment, diagnosis and prognosis of hearing loss

T2 - J Otol

TI - The potential of miR-183 family expression in inner ear for regeneration, treatment, diagnosis and prognosis of hearing loss

VL - 12

ID - 39308

ER -

TY - JOUR

AB - Hearing loss is a serious occupational health problem worldwide. Noise, aminoglycoside antibiotics and chemotherapeutic drugs induce hearing loss through changes in metabolic functions resulting in sensory cell death in the cochlea. Metabolic sequelae from noise exposure increase production of nitric oxide (NO) and Reactive Oxygen Species (ROS) contributing to higher levels of oxidative stress beyond the physiologic threshold levels of intracellular repair. Photobiomodulation (PBM) therapy is a light treatment involving endogenous chromophores commonly used to reduce inflammation and promote tissue repair. Near infrared light (NIR) from Light Emitting Diodes (LED) at 810 nm wavelength were used as a biochemical modulator of cytokine response in cultured HEI-OC1 auditory cells placed under oxidative stress. Results reported here show that NIR PBM at 810 nm, 30 mW/cm(2) , 100 seconds, 1.0 J, 3 J/cm(2) altered mitochondrial metabolism and oxidative stress response for up to 24 hours post treatment. We report a decrease of inflammatory cytokines and stress levels resulting from NIR applied to HEI-OC1 auditory cells before treatment with gentamicin or lipopolysaccharide. These results show that cells pretreated with NIR exhibit reduction of proinflammatory markers that correlate with inhibition of mitochondrial superoxide, ROS and NO in response to continuous oxidative stress challenges. Non-invasive biomolecular down regulation of proinflammatory intracellular metabolic pathways and suppression of oxidative stress via NIR may have the potential to develop novel therapeutic approaches to address noise exposure and ototoxic compounds associated with hearing loss.

AD - Harvard University - Harvard T.H. Chan School of Public Health, Molecular and Integrative Physiological Sciences - Department of Environmental Health, Building 1, 665 Huntington Ave, Boston, MA, 02115, USA.

THOR Photomedicine Ltd, Chesham, HP5 1LF, United Kingdom.

AN - 26790619

AU - Bartos, A.

AU - Grondin, Y.

AU - Bortoni, M. E.

AU - Ghelfi, E.

AU - Sepulveda, R.

AU - Carroll, J.

AU - Rogers, R. A.

C1 - PubMed

DA - Dec

DO - 10.1002/jbio.201500209

DP - NLM

ET - 2016/01/23

IS - 11-12

KW - Animals

Cell Death

Cell Line

Cytokines/*metabolism

Hair Cells, Auditory/metabolism/*radiation effects

Hearing Loss, Noise-Induced

Humans

Inflammation/metabolism

*Infrared Rays

Mice

Nitric Oxide/metabolism

*Oxidative Stress

Reactive Oxygen Species/metabolism

Hearing loss

Lllt

Low Level Laser Therapy

auditory cells

inflammation

near infrared photobiomodulation

near-infrared

oxidative stress

LA - eng

N1 - 1864-0648

Bartos, Adam

Grondin, Yohann

Bortoni, Magda E

Ghelfi, Elisa

Sepulveda, Rosalinda

Carroll, James

Rogers, Rick A

Journal Article

Germany

J Biophotonics. 2016 Dec;9(11-12):1125-1135. doi: 10.1002/jbio.201500209. Epub 2016 Jan 21.

PY - 2016

SN - 1864-063x

SP - 1125-1135

ST - Pre-conditioning with near infrared photobiomodulation reduces inflammatory cytokines and markers of oxidative stress in cochlear hair cells

T2 - J Biophotonics

TI - Pre-conditioning with near infrared photobiomodulation reduces inflammatory cytokines and markers of oxidative stress in cochlear hair cells

VL - 9

ID - 39310

ER -

TY - JOUR

AB - Noise exposures causing only transient threshold shifts can destroy auditory-nerve synapses without damaging hair cells. Here, we asked whether virally mediated neurotrophin3 (NT3) overexpression can repair this damage. CBA/CaJ mice at 6 wks were injected unilaterally with adeno-associated virus (AAV) containing either NT3 or GFP genes, via the posterior semicircular canal, 3 wks prior to, or 5 hrs after, noise exposure. Controls included exposed animals receiving vehicle only, and unexposed animals receiving virus. Thresholds were measured 2 wks post-exposure, just before cochleas were harvested for histological analysis. In separate virus-injected animals, unexposed cochleas were extracted for qRT-PCR. The GFP reporter showed that inner hair cells (IHCs) were transfected throughout the cochlea, and outer hair cells mainly in the apex. qRT-PCR showed 4- to 10-fold overexpression of NT3 from 1-21 days post-injection, and 1.7-fold overexpression at 40 days. AAV-NT3 delivered prior to noise exposure produced a dose-dependent reduction of synaptopathy, with nearly complete rescue at some cochlear locations. In unexposed ears, NT3 overexpression did not affect thresholds, however GFP overexpression caused IHC loss. In exposed ears, NT3 overexpression increased permanent threshold shifts. Thus, although NT3 overexpression can minimize noise-induced synaptic damage, the forced overexpression may be harmful to hair cells themselves during cochlear overstimulation.

AD - Department of Otolaryngology-Head and Neck Surgery, Harvard Medical School, Boston, MA, USA.

Eaton-Peabody Laboratories, Massachusetts Eye & Ear, Boston, MA, USA.

Department of Otorhinolaryngology-Head and Neck Surgery, Tohoku University Graduate School of Medicine, Sendai, Miyagi, Japan.

Department of Otolaryngology-Head and Neck Surgery, Harvard Medical School, Boston, MA, USA. Tyler_Hickman@meei.harvard.edu.

Eaton-Peabody Laboratories, Massachusetts Eye & Ear, Boston, MA, USA. Tyler_Hickman@meei.harvard.edu.

Kresge Hearing Research Institute, University of Michigan, Ann Arbor, MI, USA.

Department of Otolaryngology-Head and Neck Surgery, University of Michigan, Ann Arbor, MI, USA.

AN - 31653916

AU - Hashimoto, K.

AU - Hickman, T. T.

AU - Suzuki, J.

AU - Ji, L.

AU - Kohrman, D. C.

AU - Corfas, G.

AU - Liberman, M. C.

C1 - PubMed

C2 - PMC6814859 equity interest in and have received compensation for consulting, but the company was not involved in this study.

DA - Oct 25

DO - 10.1038/s41598-019-51724-6

DP - NLM

ET - 2019/10/28

IS - 1

KW - Animals

Auditory Threshold

Cochlea/*pathology/physiopathology

Dependovirus/*metabolism

Evoked Potentials, Auditory, Brain Stem

Green Fluorescent Proteins/metabolism

Hair Cells, Auditory, Inner/metabolism/pathology

Hair Cells, Auditory, Outer/metabolism/pathology

Male

Mice, Inbred C57BL

Mice, Inbred CBA

Neurotrophin 3/genetics/*metabolism

*Noise

Otoacoustic Emissions, Spontaneous

RNA, Messenger/genetics/metabolism

Synapses/metabolism/*pathology

LA - eng

N1 - 2045-2322

Hashimoto, Ken

Hickman, Tyler T

Orcid: 0000-0003-2623-3251

Suzuki, Jun

Orcid: 0000-0002-9164-6389

Ji, Lingchao

Kohrman, David C

Corfas, Gabriel

Orcid: 0000-0001-5412-9473

Liberman, M Charles

R01 DC000188/DC/NIDCD NIH HHS/United States

R01 DC004820/DC/NIDCD NIH HHS/United States

Journal Article

Research Support, N.I.H., Extramural

Research Support, Non-U.S. Gov't

England

Sci Rep. 2019 Oct 25;9(1):15362. doi: 10.1038/s41598-019-51724-6.

PY - 2019

SN - 2045-2322

SP - 15362

ST - Protection from noise-induced cochlear synaptopathy by virally mediated overexpression of NT3

T2 - Sci Rep

TI - Protection from noise-induced cochlear synaptopathy by virally mediated overexpression of NT3

VL - 9

ID - 39700

ER -

TY - JOUR

AB - Hair cells are specialized sensory epithelia cells that receive mechanical sound waves and convert them into neural signals for hearing, and these cells can be killed or damaged by ototoxic drugs, including many aminoglycoside antibiotics, platinum-based anticancer agents, and loop diuretics, leading to drug-induced hearing loss. Studies of therapeutic approaches to drug-induced hearing loss have been hampered by the limited understanding of the biological mechanisms that protect and regenerate hair cells. This review briefly discusses some of the most common ototoxic drugs and describes recent research concerning the mechanisms of ototoxic drug-induced hearing loss. It also highlights current developments in potential therapies and explores current clinical treatments for patients with hearing impairments.

AD - Key Laboratory of Hearing Medicine of NHFPC, ENT Institute and Otorhinolaryngology Department, Shanghai Engineering Research Centre of Cochlear Implant, Affiliated Eye and ENT Hospital, State Key Laboratory of Medical Neurobiology, Fudan University, Shanghai, China.

MOE Key Laboratory for Developmental Genes and Human Disease, Institute of Life Sciences, Jiangsu Province High-Tech Key Laboratory for Bio-Medical Research, Southeast University, Nanjing, China.

Key Laboratory of Hearing Medicine of NHFPC, ENT Institute and Otorhinolaryngology Department, Shanghai Engineering Research Centre of Cochlear Implant, Affiliated Eye and ENT Hospital, State Key Laboratory of Medical Neurobiology, Fudan University, Shanghai, China. shansun@fudan.edu.cn.

AN - 30915699

AU - Guo, J.

AU - Chai, R.

AU - Li, H.

AU - Sun, S.

C1 - PubMed

DO - 10.1007/978-981-13-6123-4_2

DP - NLM

ET - 2019/03/28

KW - Aminoglycosides/toxicity

Antineoplastic Agents/toxicity

Diuretics/toxicity

Hair Cells, Auditory/*drug effects

Hearing Loss/*chemically induced/*prevention & control

Humans

Platinum/toxicity

Aminoglycosides

Hair cell

Mitochondrial DNA

Ototoxicity

Reactive oxygen species

LA - eng

N1 - Guo, Jin

Chai, Renjie

Li, Huawei

Sun, Shan

Journal Article

Review

United States

Adv Exp Med Biol. 2019;1130:17-36. doi: 10.1007/978-981-13-6123-4_2.

PY - 2019

SN - 0065-2598 (Print)

0065-2598

SP - 17-36

ST - Protection of Hair Cells from Ototoxic Drug-Induced Hearing Loss

T2 - Adv Exp Med Biol

TI - Protection of Hair Cells from Ototoxic Drug-Induced Hearing Loss

VL - 1130

ID - 38839

ER -

TY - JOUR

AB - In the auditory system, the primary sensory neurons, spiral ganglion neurons (SGNs), transmit complex acoustic information from hair cells to the second-order sensory neurons in the cochlear nucleus for sound processing, thus building the initial bridge between the physical world of sound and the perception of that sound. Cochlear SGN loss causes irreversible hearing impairment because this type of neural cell cannot regenerate. A better understanding of the molecular mechanisms of formation, structure, degeneration, and protection of SGNs will help to design potential therapeutic strategies for preservation and replacement of them in the cochlear implant recipient. In this review, we described and summarized the following about SGNs: (1) their cell biology and their peripheral and central connections, (2) mechanisms of their neuronal damage and their protection, and (3) the neural and synaptic mechanism of auditory neuropathy and current options for hearing rehabilitation from auditory neuropathy. The updates of the research progress and the significant issues on these topics were discussed.

AD - Otolaryngology-Head and Neck Surgery, Shandong Provincial ENT Hospital, Shandong Provincial ENT Hospital Affiliated to Shandong University, Jinan, China.

Shandong Provincial Key Laboratory of Otology, Jinan, China.

Otolaryngology-Head and Neck Surgery, Shandong Provincial ENT Hospital, Shandong Provincial ENT Hospital Affiliated to Shandong University, Jinan, China. whboto11@163.com.

Shandong Provincial Key Laboratory of Otology, Jinan, China. whboto11@163.com.

AN - 30915703

AU - Liu, W.

AU - Wang, X.

AU - Wang, M.

AU - Wang, H.

C1 - PubMed

DO - 10.1007/978-981-13-6123-4_6

DP - NLM

ET - 2019/03/28

KW - Hair Cells, Auditory/*cytology

Hearing Loss, Central/*prevention & control

Humans

Neurons/*cytology

Spiral Ganglion/*physiopathology

Auditory neuropathy

Cochlea implantation

Neuronal damage

Spiral ganglion neuron

Synapse

LA - eng

N1 - Liu, Wenwen

Wang, Xue

Wang, Man

Wang, Haibo

Journal Article

Review

United States

Adv Exp Med Biol. 2019;1130:93-107. doi: 10.1007/978-981-13-6123-4_6.

PY - 2019

SN - 0065-2598 (Print)

0065-2598

SP - 93-107

ST - Protection of Spiral Ganglion Neurons and Prevention of Auditory Neuropathy

T2 - Adv Exp Med Biol

TI - Protection of Spiral Ganglion Neurons and Prevention of Auditory Neuropathy

VL - 1130

ID - 38899

ER -

TY - JOUR

AB - Cisplatin-induced ototoxicity leads to hearing impairment, possibly through reactive oxygen species (ROS) production and DNA damage in cochlear hair cells (HC), although the exact mechanism is unknown. Avenanthramide-C (AVN-C), a natural, potent antioxidant, was evaluated in three study groups of normal adult C57Bl/6 mice (control, cisplatin, and AVN-C+cisplatin) for the prevention of cisplatin-induced hearing loss. Auditory brainstem responses and immunohistochemistry of outer hair cells (OHCs) were ascertained. Cell survival, ROS production, Phospho-H2AX-enabled tracking of DNA damage-repair kinetics, and expression levels of inflammatory cytokines (TNF-α, IL-1β, IL6, iNOS, and COX2) were assessed using House Ear Institute-Organ of Corti 1 (HEI-OC1 Cells). In the in vivo mouse model, following cisplatin-induced damage, AVN-C decreased the hearing thresholds and sheltered all cochlear turns' OHCs. In HEI-OC1 cells, AVN-C preserved cell viability and decreased ROS production, whereas cisplatin enhanced both ROS levels and cell viability. In HEI-OC1 cells, AVN-C downregulated IL6, IL-1β, TNF-α, iNOS, and COX2 production that was upregulated by cisplatin treatment. AVN-C attenuated the cisplatin-enhanced nuclear H2AX activation. AVN-C had a strong protective effect against cisplatin-induced ototoxicity through inhibition of ROS and inflammatory cytokine production and DNA damage and is thus a promising candidate for preventing cisplatin-induced sensorineural hearing loss.

AD - Department of Otolaryngology-Head and Neck Surgery, Chonnam National University Hospital, Chonnam National University Medical School, Gwangju 61469, Republic of Korea.

Department of Biomedical Science, College of Medicine, Chonnam National University Graduate School, Gwangju 61469, Republic of Korea.

BK21 PLUS Center for Creative Biomedical Scientists at Chonnam National University, Gwangju 61469, Republic of Korea.

Department of Medicinal Biotechnology, College of Health Sciences, Dong-A University, Busan 49315, Republic of Korea.

Department of Translational Biomedical Sciences, Graduate School of Dong-A University, Busan 49201, Republic of Korea.

AN - 36769271

AU - Umugire, A.

AU - Nam, Y. S.

AU - Nam, Y. E.

AU - Choi, Y. M.

AU - Choi, S. M.

AU - Lee, S.

AU - Cho, J. H.

AU - Cho, H. H.

C1 - PubMed

C2 - PMC9918115

DA - Feb 2

DO - 10.3390/ijms24032947

DP - NLM

ET - 2023/02/12

IS - 3

KW - Mice

Animals

Cisplatin/toxicity/metabolism

Cytokines/metabolism

*Antineoplastic Agents/pharmacology

Reactive Oxygen Species/metabolism

*Ototoxicity/etiology/metabolism

Interleukin-6/metabolism

Tumor Necrosis Factor-alpha/metabolism

Cyclooxygenase 2/metabolism

Cell Line

Apoptosis

Hair Cells, Auditory/metabolism

Oxidative Stress

*Hearing Loss/chemically induced/prevention & control/metabolism

DNA Damage

ortho-Aminobenzoates

Ros

avenanthramide-C

cisplatin

hair cell loss

hearing loss

LA - eng

N1 - 1422-0067

Umugire, Alphonse

Orcid: 0000-0001-6515-5466

Nam, Yoon Seok

Orcid: 0000-0003-1432-3321

Nam, Ye Eun

Orcid: 0000-0002-9110-3309

Choi, Young Mi

Choi, Se Myeong

Orcid: 0000-0003-0431-9185

Lee, Sungsu

Cho, Jong Hyun

Orcid: 0000-0002-0995-6952

Cho, Hyong-Ho

Orcid: 0000-0002-1331-4039

2022C100/Commercialization Promotion Agency for R&D Outcomes (COMPA) through a grant that was funded by the Korean government/

CRI18035-1/Chonnam National University Hospital Biomedical Research Institute/

Journal Article

Switzerland

Int J Mol Sci. 2023 Feb 2;24(3):2947. doi: 10.3390/ijms24032947.

PY - 2023

SN - 1422-0067

ST - Protective Effect of Avenanthramide-C on Auditory Hair Cells against Oxidative Stress, Inflammatory Cytokines, and DNA Damage in Cisplatin-Induced Ototoxicity

T2 - Int J Mol Sci

TI - Protective Effect of Avenanthramide-C on Auditory Hair Cells against Oxidative Stress, Inflammatory Cytokines, and DNA Damage in Cisplatin-Induced Ototoxicity

VL - 24

ID - 39086

ER -

TY - JOUR

AB - Sea anemones have an extraordinary capability to repair damaged hair bundles, even after severe trauma. A group of secreted proteins, named repair proteins (RPs), found in mucus covering sea anemones significantly assists the repair of damaged hair bundle mechanoreceptors both in the sea anemone Haliplanella luciae and the blind cavefish Astyanax hubbsi. The polypeptide constituents of RPs must be identified in order to gain insight into the molecular mechanisms by which repair of hair bundles is accomplished. In this study, several polypeptides of RPs were isolated from mucus using blue native PAGE and then sequenced using LC-MS/MS. Thirty-seven known polypeptides were identified, including Hsp70s, as well as many polypeptide subunits of the 20S proteasome. Other identified polypeptides included those involved in cellular stress responses, protein folding, and protein degradation. Specific inhibitors of Hsp70s and the 20S proteasome were employed in experiments to test their involvement in hair bundle repair. The results of those experiments suggested that repair requires biologically active Hsp70s and 20S proteasomes. A model is proposed that considers the function of extracellular Hsp70s and 20S proteasomes in the repair of damaged hair cells.

AD - Department of Biology, University of Louisiana Lafayette, USA.

Department of Biology, University of Louisiana Lafayette, USA. Electronic address: gmw5722@louisiana.edu.

AN - 26183436

AU - Tang, P. C.

AU - Watson, G. M.

C1 - PubMed

DA - Sep

DO - 10.1016/j.heares.2015.07.005

DP - NLM

ET - 2015/07/18

KW - Animals

Chromatography, Liquid

HSP70 Heat-Shock Proteins/metabolism

Hair Cells, Auditory/*metabolism/pathology

Proteasome Endopeptidase Complex/metabolism

Proteins/*metabolism

*Proteomics/methods

Sea Anemones/*metabolism

Tandem Mass Spectrometry

20S proteasome

Hair bundle

Hsp70s

Repair proteins

LA - eng

N1 - 1878-5891

Tang, Pei-Ciao

Watson, Glen M

Journal Article

Netherlands

Hear Res. 2015 Sep;327:245-56. doi: 10.1016/j.heares.2015.07.005. Epub 2015 Jul 14.

PY - 2015

SN - 0378-5955

SP - 245-56

ST - Proteomic identification of hair cell repair proteins in the model sea anemone Nematostella vectensis

T2 - Hear Res

TI - Proteomic identification of hair cell repair proteins in the model sea anemone Nematostella vectensis

VL - 327

ID - 39395

ER -

TY - JOUR

AB - Cochlear hair cell loss is a leading cause of deafness in humans. Neighboring supporting cells have some capacity to regenerate hair cells. However, their regenerative potential sharply declines as supporting cells undergo maturation (postnatal day 5 in mice). We recently reported that reactivation of the RNA-binding protein LIN28B restores the hair cell-regenerative potential of P5 cochlear supporting cells. Here, we identify the LIN28B target Trim71 as a novel and equally potent enhancer of supporting cell plasticity. TRIM71 is a critical regulator of stem cell behavior and cell reprogramming, however, its role in cell regeneration is poorly understood. Employing an organoid-based assay, we show that TRIM71 reactivation increases the mitotic and hair cell-forming potential of P5 cochlear supporting cells by facilitating their de-differentiation into progenitor-like cells. Our mechanistic work indicates that TRIM71â€™s RNA-binding activity is essential for such ability, and our transcriptomic analysis identifies gene modules that are linked to TRIM71 and LIN28B-mediated supporting cell reprogramming. Furthermore, our study uncovers that the TRIM71-LIN28B target Hmga2 is essential for supporting cell self-renewal and hair cell formation.

AN - 36711735

AU - Li, X. J.

AU - Morgan, C.

AU - Nadar-Ponniah, P. T.

AU - Kolanus, W.

AU - Doetzlhofer, A.

C1 - PubMed

C2 - PMC9882147

DA - Jan 13

DO - 10.1101/2023.01.12.523802

DP - NLM

ET - 2023/01/31

LA - eng

N1 - 2692-8205

Li, Xiao-Jun

Morgan, Charles

Nadar-Ponniah, Prathamesh T

Kolanus, Waldemar

Doetzlhofer, Angelika

Orcid: 0000-0002-7424-2112

Journal Article

Preprint

United States

bioRxiv [Preprint]. 2023 Jan 13:2023.01.12.523802. doi: 10.1101/2023.01.12.523802.

PY - 2023

SN - 2692-8205

ST - Reactivation of the progenitor gene Trim71 enhances the mitotic and hair cell-forming potential of cochlear supporting cells

T2 - bioRxiv

TI - Reactivation of the progenitor gene Trim71 enhances the mitotic and hair cell-forming potential of cochlear supporting cells

ID - 39076

ER -

TY - JOUR

AB - Sensorineural hearing loss is caused by damage to sensory hair cells and/or spiral ganglion neurons. In non-mammalian species, hair cell regeneration after damage is observed, even in adulthood. Although the neonatal mammalian cochlea carries regenerative potential, the adult cochlea cannot regenerate lost hair cells. The survival of supporting cells with regenerative potential after cochlear trauma in adults is promising for promoting hair cell regeneration through therapeutic approaches. Targeting these cells by manipulating key signaling pathways that control mammalian cochlear development and non-mammalian hair cell regeneration could lead to regeneration of hair cells in the mammalian cochlea. This review discusses the pathways involved in the development of the cochlea and the impact that trauma has on the regenerative capacity of the endogenous progenitor cells. Furthermore, it discusses the effects of manipulating key signaling pathways targeting supporting cells with progenitor potential to promote hair cell regeneration and translates these findings to the human situation. To improve hearing recovery after hearing loss in adults, we propose a combined approach targeting (1) the endogenous progenitor cells by manipulating signaling pathways (Wnt, Notch, Shh, FGF and BMP/TGFβ signaling pathways), (2) by manipulating epigenetic control, and (3) by applying neurotrophic treatments to promote reinnervation. © 2023 by the authors.

AD - Department of Otorhinolaryngology and Head & Neck Surgery, University Medical Center Utrecht, Utrecht University, Heidelberglaan 100, Utrecht, 3584 CX, Netherlands

UMC Utrecht Brain Center, Utrecht University, Universiteitsweg 100, Utrecht, 3584 CG, Netherlands

AU - Smith-Cortinez, N.

AU - Tan, A. K.

AU - Stokroos, R. J.

AU - Versnel, H.

AU - Straatman, L. V.

C1 - Scopus

C2 - 37175547

C7 - 7840

DB - Scopus

DO - 10.3390/ijms24097840

IS - 9

J2 - Int. J. Mol. Sci.

KW - endogenous progenitor cells

inner ear regeneration

re-innervation

Adult

Cochlea

Hair Cells, Auditory

Humans

Infant, Newborn

Neurogenesis

Signal Transduction

Spiral Ganglion

bone morphogenetic protein

fibroblast growth factor

Notch receptor

sonic hedgehog protein

transforming growth factor beta

Wnt protein

BMP signaling

cell regeneration

cell survival

clinical trial (topic)

epigenetics

hair cell (inner ear)

hearing impairment

human

mammal

nonhuman

Notch signaling

pathophysiology

reinnervation

Review

stem cell

target cell

TGF beta signaling

Wnt signaling

cochlear hair cell

metabolism

nervous system development

newborn

LA - English

M3 - Review

N1 - Export Date: 21 May 2026; Cited By: 8; Correspondence Address: L.V. Straatman; Department of Otorhinolaryngology and Head & Neck Surgery, University Medical Center Utrecht, Utrecht University, Utrecht, Heidelberglaan 100, 3584 CX, Netherlands; email: l.v.straatman@umcutrecht.nl

PY - 2023

SN - 16616596 (ISSN)

ST - Regeneration of Hair Cells from Endogenous Otic Progenitors in the Adult Mammalian Cochlea: Understanding Its Origins and Future Directions

T2 - International Journal of Molecular Sciences

TI - Regeneration of Hair Cells from Endogenous Otic Progenitors in the Adult Mammalian Cochlea: Understanding Its Origins and Future Directions

UR - https://www.scopus.com/pages/publications/85159375810?origin=resultslist

VL - 24

ID - 39858

ER -

TY - JOUR

AB - Hearing loss affects hundreds of millions of people worldwide by dampening or cutting off their auditory connection to the world. Current treatments for sensorineural hearing loss (SNHL) with cochlear implants are not perfect, leaving regenerative medicine as the logical avenue to a perfect cure. Multiple routes to regeneration of damaged hair cells have been proposed and are actively pursued. Each route not only requires a keen understanding of the molecular basis of ear development but also faces the practical limitations of stem cell regulation in the delicate inner ear where topology of cell distribution is essential. Improvements in our molecular understanding of the minimal essential genes necessary for hair cell formation and recent advances in stem cell manipulation, such as seen with inducible pluripotent stem cells (iPSCs) and epidermal neural crest stem cells (EPI-NCSCs), have opened new possibilities to advance research in translational stem cell therapies for individuals with hearing loss. Despite this, more detailed network maps of gene expression are needed, including an appreciation for the roles of microRNAs (miRs), key regulators of transcriptional gene networks. To harness the true potential of stem cells for hair cell regeneration, basic science and clinical medicine must work together to expedite the transition from bench to bedside by elucidating the full mechanisms of inner ear hair cell development, including a focus on the role of miRs, and adapting this knowledge safely and efficiently to stem cell technologies.

AD - Department of Biology, University of Iowa, Iowa City, IA, 52242, USA.

AN - 21966254

AU - Kopecky, B.

AU - Fritzsch, B.

C1 - PubMed

C2 - PMC3180915

C6 - NIHMS324994

DA - Jun 1

DO - 10.3390/ph4060848

DP - NLM

ET - 2011/10/04

IS - 6

LA - eng

N1 - Kopecky, Benjamin

Fritzsch, Bernd

P30 DC010362/DC/NIDCD NIH HHS/United States

R01 DC005590/DC/NIDCD NIH HHS/United States

T32 GM007337/GM/NIGMS NIH HHS/United States

Journal Article

Switzerland

Pharmaceuticals (Basel). 2011 Jun 1;4(6):848-879. doi: 10.3390/ph4060848.

PY - 2011

SN - 1424-8247 (Print)

1424-8247

SP - 848-879

ST - Regeneration of Hair Cells: Making Sense of All the Noise

T2 - Pharmaceuticals (Basel)

TI - Regeneration of Hair Cells: Making Sense of All the Noise

VL - 4

ID - 39045

ER -

TY - JOUR

AB - Hearing loss and balance dysfunction are commonly caused by the loss of sensory hair cells, the cells which detect sound waves in the auditory organs and head movements in the vestibule. Replacement and regeneration of damaged hair cells occurs naturally in non-mammalian vertebrates such as birds and fish. While a small amount of hair cell regeneration occurs in the vestibular organs of mammals even at adult ages, this process only happens in the cochlea during the first days after birth and does not, in either system, result in recovery of function. Here, we review what is known about the natural capacity for hair cell regeneration in mammals, comparing auditory and vestibular organs. We also discuss strategies to induce the formation of new hair cells in the adult inner ear such as reprogramming the remaining supporting cells with genes to drive a hair cell fate or induce proliferation. Finally, we present a roadmap for what is needed to restore auditory and vestibular function and discuss the challenges that remain.

AD - Department of Otolaryngology/Head and Neck Surgery, University of Mississippi, Jackson, MS, United States.

Departments of Pharmacology and Otolaryngology, Southern Illinois University School of Medicine, Springfield, IL, United States.

Department of Otolaryngology/Head and Neck Surgery, University of Washington School of Medicine, Seattle, WA, United States. Electronic address: stoner@uw.edu.

AN - 40973232

AU - Walters, B.

AU - Cox, B.

AU - Stone, J.

C1 - PubMed

DO - 10.1016/bs.ctdb.2025.04.002

DP - NLM

ET - 2025/09/20

KW - Animals

*Hair Cells, Auditory/physiology/cytology

*Regeneration/physiology

*Mammals/physiology

Humans

Direct transdifferentiation

Hair cell

Mitotic regeneration

Proliferation

Reprogramming

Supporting cell

LA - eng

N1 - 1557-8933

Walters, Bradley

Cox, Brandon

Stone, Jennifer

Journal Article

Review

United States

Curr Top Dev Biol. 2025;165:307-351. doi: 10.1016/bs.ctdb.2025.04.002. Epub 2025 Apr 29.

PY - 2025

SN - 0070-2153

SP - 307-351

ST - Regeneration of sensory hair cells in mature mammals

T2 - Curr Top Dev Biol

TI - Regeneration of sensory hair cells in mature mammals

VL - 165

ID - 38797

ER -

TY - JOUR

AB - Using adult zebrafish inner ears as a model for sensorineural regeneration, we ablated the mechanosensory receptors and characterized the single-cell epigenome and transcriptome at consecutive time points during hair cell regeneration. We utilized deep learning on the regeneration-induced open chromatin sequences and identified cell-specific transcription factor (TF) motif patterns. Enhancer activity correlated with gene expression and identified potential gene regulatory networks. A pattern of overlapping Sox- and Six-family TF gene expression and binding motifs was detected, suggesting a combinatorial program of TFs driving regeneration and cell identity. Pseudotime analysis of single-cell transcriptomic data suggested that support cells within the sensory epithelium changed cell identity to a "progenitor" cell population that could differentiate into hair cells. We identified a 2.6 kb DNA enhancer upstream of the sox2 promoter that, when deleted, showed a dominant phenotype that resulted in a hair-cell-regeneration-specific deficit in both the lateral line and adult inner ear.

AD - Translational and Functional Genomics Branch, National Human Genome Research Institute, Bethesda, MD, USA.

Computational Biology Branch, National Center for Biotechnology Information, National Library of Medicine, National Institutes of Health, Bethesda, MD 20894, USA.

CAS Key Laboratory of Tropical Marine Bio-Resources and Ecology, South China Sea Institute of Oceanology, Chinese Academy of Sciences, Guangzhou, China.

Southern Marine Science and Engineering Guangdong Laboratory, Guangzhou, China.

Vaccine Immunology Program, Vaccine Research Center, National Institute of Allergy and Infectious Diseases, Bethesda, MD, USA.

Cancer Genetics and Comparative Genomics Branch, National Human Genome Research Institute, Bethesda, MD, USA.

Lead contact.

AN - 36212030

AU - Jimenez, E.

AU - Slevin, C. C.

AU - Song, W.

AU - Chen, Z.

AU - Frederickson, S. C.

AU - Gildea, D.

AU - Wu, W.

AU - Elkahloun, A. G.

AU - Ovcharenko, I.

AU - Burgess, S. M.

C1 - PubMed

C2 - PMC9540346

C6 - NIHMS1837489

DA - Sep 14

DO - 10.1016/j.xgen.2022.100170

DP - NLM

ET - 2022/10/11

IS - 9

LA - eng

N1 - 2666-979x

Jimenez, Erin

Slevin, Claire C

Song, Wei

Chen, Zelin

Frederickson, Stephen C

Gildea, Derek

Wu, Weiwei

Elkahloun, Abdel G

Ovcharenko, Ivan

Burgess, Shawn M

ZIA HG200386/ImNIH/Intramural NIH HHS/United States

Journal Article

United States

Cell Genom. 2022 Sep 14;2(9):100170. doi: 10.1016/j.xgen.2022.100170. Epub 2022 Aug 22.

PY - 2022

SN - 2666-979x

ST - A regulatory network of Sox and Six transcription factors initiate a cell fate transformation during hearing regeneration in adult zebrafish

T2 - Cell Genom

TI - A regulatory network of Sox and Six transcription factors initiate a cell fate transformation during hearing regeneration in adult zebrafish

VL - 2

ID - 39353

ER -

TY - JOUR

AB - Exposure to intense sound or ototoxic chemicals can damage the auditory hair cells of vertebrates, resulting in hearing loss. Although the relationship between such hair cell damage and auditory function is fairly established for terrestrial vertebrates, there are limited data available to understand this relationship in fishes. Although investigators have measured either the morphological damage of the inner ear or the functional deficits in the hearing of fishes, very few have directly measured both in an attempt to find a relationship between the two. Those studies that have examined both auditory hair cell damage in the inner ear and the resulting hearing loss in fishes are reviewed here. In general, there is a significant linear relationship between the number of hair cells lost and the severity of hearing threshold shifts, although this varies between species and different hair cell-damaging stimuli. After trauma to the fish ear, auditory hair cells are able to regenerate to control level densities. With this regeneration also comes a restoration of hearing. Thus there is also a significant relationship between hair cell recovery and hearing recovery in fishes.

AD - Department of Biology, Western Kentucky University, Bowling Green, KY, 42101, USA. michael.smith1@wku.edu.

AN - 26611069

AU - Smith, M. E.

C1 - PubMed

DO - 10.1007/978-1-4939-2981-8_132

DP - NLM

ET - 2015/11/28

KW - Animals

Auditory Threshold

Environmental Exposure

Fishes/*physiology

Hair Cells, Auditory/*pathology

Hearing Loss, Noise-Induced/*pathology/physiopathology

Saccule and Utricle/pathology

Acoustic exposure

Auditory evoked potential

Regeneration

Regression

Temporary threshold shift

LA - eng

N1 - Smith, Michael E

8 P20 GM103436-12/GM/NIGMS NIH HHS/United States

Journal Article

Research Support, N.I.H., Extramural

United States

Adv Exp Med Biol. 2016;875:1067-74. doi: 10.1007/978-1-4939-2981-8_132.

PY - 2016

SN - 0065-2598 (Print)

0065-2598

SP - 1067-74

ST - Relationship Between Hair Cell Loss and Hearing Loss in Fishes

T2 - Adv Exp Med Biol

TI - Relationship Between Hair Cell Loss and Hearing Loss in Fishes

VL - 875

ID - 38928

ER -

TY - JOUR

AB - Cochlear hair cells can be killed by loud noises, ototoxic drugs, and natural aging. Once lost, mammalian hair cells do not naturally regenerate, leading to permanent hearing loss. Since the mammalian cochlea lacks any intrinsic ability to regenerate, genetic reprogramming of cochlear supporting cells that lie adjacent to hair cells is a potential option for hearing restoration therapies. We targeted cochlear supporting cells with three hair cell transcription factors: Atoh1, or Atoh1 + Gfi1, or Atoh1 + Gfi1 + Pou4f3 and found that 1- and 2-factor reprogramming is not sufficient to reprogram adult supporting cells into hair cells. However, activation of all three hair cell transcription factors reprogrammed some adult supporting cells into hair cell-like cells. We found that killing endogenous hair cells significantly improved the ability of supporting cells to be reprogrammed and regenerated numerous hair cell-like cells throughout the length of the cochlea. These regenerated hair cell-like cells expressed myosin VIIa and parvalbumin, as well as the mature outer hair cell protein prestin, were innervated, expressed proteins associated with ribbon synapses, and formed rudimentary stereociliary bundles. Finally, we demonstrate that supporting cells remained responsive to transcription factor reprogramming for at least 6 weeks after hair cell damage, suggesting that hair cell reprogramming may be effective in the chronically deafened cochlea.

AD - Department of Neuroscience, Baylor College of Medicine, Houston, TX 77030, USA.

Department of Otolaryngology-Head and Neck Surgery, University of Mississippi Medical Center, Jackson, MS 39216, USA.

Department of Molecular and Human Genetics, Baylor College of Medicine, Houston, TX 77030, USA.

Program in Genetics and Genomics, Baylor College of Medicine, Houston, TX 77030, USA.

AN - 39411090

AU - McGovern, M. M.

AU - Ghosh, S.

AU - Dupuis, C.

AU - Walters, B. J.

AU - Groves, A. K.

C1 - PubMed

C2 - PMC11477985

DA - Oct

DO - 10.1093/pnasnexus/pgae445

DP - NLM

ET - 2024/10/16

IS - 10

KW - hair cells

inner ear

reprogramming

LA - eng

N1 - 2752-6542

McGovern, Melissa M

Orcid: 0000-0003-2254-8782

Ghosh, Sumana

Dupuis, Colleen

Walters, Bradley J

Groves, Andrew K

Orcid: 0000-0002-0784-7998

R01 DC014832/DC/NIDCD NIH HHS/United States

Journal Article

England

PNAS Nexus. 2024 Oct 4;3(10):pgae445. doi: 10.1093/pnasnexus/pgae445. eCollection 2024 Oct.

PY - 2024

SN - 2752-6542

SP - pgae445

ST - Reprogramming with Atoh1, Gfi1, and Pou4f3 promotes hair cell regeneration in the adult organ of Corti

T2 - PNAS Nexus

TI - Reprogramming with Atoh1, Gfi1, and Pou4f3 promotes hair cell regeneration in the adult organ of Corti

VL - 3

ID - 38793

ER -

TY - JOUR

AB - INTRODUCTION: Inner ear progenitor cells have the potential for multi-directional differentiation. Retinoic acid is an important requirement for the development of the inner ear. Blocking the Curtyr's retinoic acid signaling pathway can significantly reduce the number of hair cells. Therefore, we believe that retinoic acid may induce the regeneration of inner ear hair cells. OBJECTIVE: To investigate whether the cochlear neural progenitor cells maintain the characteristics of stem cells during recovery and subculture, whether retinoic acid can induce cochlear neural progenitor cells into hair cells in vitro, and whether retinoic acid promotes or inhibits the proliferation of cochlear neural progenitor cells during differentiation. METHODS: Cochlear neural progenitor cells were cultured and induced in DMEM/F12+RA (10(-6)M) and then detected the expressions of hair cell markers (Math1 and MyosinVIIa) by immunofluorescence cytochemistry and realtime-polymerase chain reaction, and the proliferation of cochlear neural progenitor cells was detected by Brdu. RESULTS: The nestin of cochlear neural progenitor cells was positively expressed. The ratios of Math1-positive cells in the control group and experimental group were 1.5% and 63%, respectively; the ratios of MyosinVIIa-positive cells in the control group and experimental group were 0.96% and 56%, respectively (p<0.05). The ratios of Brdu(+)-labeled cells in retinoic acid group, group PBS, and group FBS were 20.6%, 29.9%, and 54.3%, respectively; however, the proliferation rate in the experimental group decreased. CONCLUSION: Retinoic acid can promote cochlear neural progenitor cells to differentiate into the hair cells.

AD - The Second Affiliated Hospital of Fujian Medical University, Department of Otolaryngology, Fujian, China.

Fujian Medical University Union Hospital, Department of Otolaryngology, Fujian, China. Electronic address: myjmcn@163.com.

AN - 33707121

AU - Chen, M.

AU - Huang, J.

C1 - PubMed

C2 - PMC9615533

DA - Nov-Dec

DO - 10.1016/j.bjorl.2021.01.005

DP - NLM

ET - 2021/03/13

IS - 6

KW - Humans

*Tretinoin/pharmacology

Bromodeoxyuridine

Cells, Cultured

*Neural Stem Cells

Cell Differentiation

Cochlear neural progenitor cells

Math1

MyosinVIIa

Retinoic acid

LA - eng

N1 - 1808-8686

Chen, Minyun

Huang, Jianmin

Journal Article

Brazil

Braz J Otorhinolaryngol. 2022 Nov-Dec;88(6):962-967. doi: 10.1016/j.bjorl.2021.01.005. Epub 2021 Feb 21.

PY - 2022

SN - 1808-8694 (Print)

1808-8686

SP - 962-967

ST - Retinoic acid induces differentiation of cochlear neural progenitor cells into hair cells

T2 - Braz J Otorhinolaryngol

TI - Retinoic acid induces differentiation of cochlear neural progenitor cells into hair cells

VL - 88

ID - 38925

ER -

TY - JOUR

AB - Sensory neural hearing loss and vestibular dysfunction have become the most common forms of sensory defects, affecting millions of people worldwide. Developing effective therapies to restore hearing loss is challenging, owing to the limited regenerative capacity of the inner ear hair cells. With recent advances in understanding the developmental biology of mammalian and non-mammalian hair cells a variety of strategies have emerged to restore lost hair cells are being developed. Two predominant strategies have developed to restore hair cells: transfer of genes responsible for hair cell genesis and replacement of missing cells via transfer of stem cells. In this review article, we evaluate the use of several genes involved in hair cell regeneration, the advantages and disadvantages of the different viral vectors employed in inner ear gene delivery and the insights gained from the use of embryonic, adult and induced pluripotent stem cells in generating inner ear hair cells. Understanding the role of genes, vectors and stem cells in therapeutic strategies led us to explore potential solutions to overcome the limitations associated with their use in hair cell regeneration.

AD - Bioengineering Program, University of Kansas, Lawrence, KS 66045, USA. keerthanad@gmail.com.

Department of Otolaryngology Head and Neck Surgery, University of Kansas School of Medicine, Kansas City, KS 66160, USA. hstaecker@kumc.edu.

Bioengineering Program, University of Kansas, Lawrence, KS 66045, USA. detamore@ku.edu.

AN - 24956306

AU - Devarajan, K.

AU - Staecker, H.

AU - Detamore, M. S.

C1 - PubMed

C2 - PMC4030941

DA - Sep 13

DO - 10.3390/jfb2030249

DP - NLM

ET - 2011/01/01

IS - 3

LA - eng

N1 - 2079-4983

Devarajan, Keerthana

Staecker, Hinrich

Detamore, Michael S

Journal Article

Switzerland

J Funct Biomater. 2011 Sep 13;2(3):249-70. doi: 10.3390/jfb2030249.

PY - 2011

SN - 2079-4983 (Print)

2079-4983

SP - 249-70

ST - A review of gene delivery and stem cell based therapies for regenerating inner ear hair cells

T2 - J Funct Biomater

TI - A review of gene delivery and stem cell based therapies for regenerating inner ear hair cells

VL - 2

ID - 39241

ER -

TY - JOUR

AB - Insulin-like growth factor-I (IGF-I) belongs to the family of polypeptides of insulin, which play a central role in embryonic development and adult nervous system homeostasis by endocrine, autocrine, and paracrine mechanisms. IGF-I is fundamental for the regulation of cochlear development, growth, and differentiation, and its mutations are associated with hearing loss in mice and men. Low levels of IGF-I have been shown to correlate with different human syndromes showing hearing loss and with presbyacusis. Animal models are fundamental to understand the genetic, epigenetic, and environmental factors that contribute to human hearing loss. In the mouse, IGF-I serum levels decrease with aging and there is a concomitant hearing loss and retinal degeneration. In the Igf1(-/-) null mouse, hearing loss is due to neuronal loss, poor innervation of the sensory hair cells, and age-related stria vascularis alterations. In the inner ear, IGF-I actions are mediated by intracellular signaling networks, RAF, AKT, and p38 MAPK protein kinases modulate the expression and activity of transcription factors, as AP1, MEF2, FoxM1, and FoxP3, leading to the regulation of cell cycle and metabolism. Therapy with rhIGF-I has been approved in humans for the treatment of poor linear growth and certain neurodegenerative diseases. This review will discuss these findings and their implications in new IGF-I-based treatments for the protection or repair of hearing loss.

AD - Servicio de Evaluación Neurofuncional no Invasiva, Instituto de Investigaciones Biomédicas Alberto Sols, Consejo Superior de Investigaciones Científicas-Universidad Autónoma de Madrid Madrid, Spain.

AN - 21845174

AU - Murillo-Cuesta, S.

AU - Rodríguez-de la Rosa, L.

AU - Cediel, R.

AU - Lassaletta, L.

AU - Varela-Nieto, I.

C1 - PubMed

C2 - PMC3146045

DO - 10.3389/fnmol.2011.00011

DP - NLM

ET - 2011/08/17

KW - IGF1R signaling

animal models

deafness

human genetics

insulin-like factors

organ of Corti

LA - eng

N1 - 1662-5099

Murillo-Cuesta, Silvia

Rodríguez-de la Rosa, Lourdes

Cediel, Rafael

Lassaletta, Luis

Varela-Nieto, Isabel

Journal Article

Switzerland

Front Mol Neurosci. 2011 Jul 25;4:11. doi: 10.3389/fnmol.2011.00011. eCollection 2011.

PY - 2011

SN - 1662-5099

SP - 11

ST - The role of insulin-like growth factor-I in the physiopathology of hearing

T2 - Front Mol Neurosci

TI - The role of insulin-like growth factor-I in the physiopathology of hearing

VL - 4

ID - 39759

ER -

TY - JOUR

AB - Deafness is one of the major global health problems that seriously affects the quality of human life. At present, there are no successful treatments for deafness caused by cochlear hair cell (HC) damage. The irreversibility of mammalian hearing impairment is that the inner ear's sensory epithelium cannot repair lost hair cells and neurons through spontaneous regeneration. The goal of stem cell therapy for sensorineural hearing loss is to reconstruct the damaged inner ear structure and achieve functional repair. microRNA (miRNA), as a class of highly conserved endogenous non-coding small RNAs, plays an important role in the development of cochlea and HCs. miRNA also participates in the regulation of stem cell proliferation and differentiation, and plays an important role in the process of regeneration of inner ear HCs, miRNA has a broad application prospect of clinical treatment of hearing loss, which is conducive to solving the medical problem of inner ear HC regeneration.

AD - Department of Otorhinolaryngology, Union Hospital, Tongji Medical College, Huazhong University of Science and Technology Wuhan 430022, P. R. China.

Otorhinolaryngology Department, Sun Yat-sen Memorial Hospital, Sun Yat-Sen University 107 West Yan Jiang Road, Guangzhou 510120, P. R. China.

AN - 32419976

AU - Wu, X.

AU - Zou, S.

AU - Wu, F.

AU - He, Z.

AU - Kong, W.

C1 - PubMed

C2 - PMC7218733

DP - NLM

ET - 2020/05/19

IS - 2

KW - Hair cell

hearing loss

inner ear

miRNA

stem cell

LA - eng

N1 - 2160-4150

Wu, Xia

Zou, Shengyu

Wu, Fan

He, Zuhong

Kong, Weijia

Journal Article

Review

United States

Am J Stem Cells. 2020 Apr 25;9(2):16-24. eCollection 2020.

PY - 2020

SN - 2160-4150 (Print)

2160-4150

SP - 16-24

ST - Role of microRNA in inner ear stem cells and related research progress

T2 - Am J Stem Cells

TI - Role of microRNA in inner ear stem cells and related research progress

VL - 9

ID - 38846

ER -

TY - JOUR

AB - Gene expression changes contribute greatly to phenotypic variations in nature. Studying patterns of regulators of gene expression is important to fully understand the molecular mechanism underlying phenotypic variations. In horseshoe bats, the cochleae are finely tuned to echoes of call frequency. Here, using 2 recently diverged subspecies of the intermediate horseshoe bat (Rhinolophus affinis hainanus and R. a. himalayanus) with great acoustic variations as the system, we aim to explore relative roles of different regulators of gene expression (differential gene expression, alternative splicing (AS) and long non-coding RNAs (lncRNAs)) in phenotypic variation with a combination of Illumina short-read and Nanopore long-read RNA-seq data from the cochlea. Compared to R. a. hainanus, R. a. himalayanus exhibited much more upregulated differentially expressed genes (DEGs) and multiple of them may play important roles in the maintenance and damage repair of auditory hair cells. We identified 411 differentially expressed lncRNAs and their target DEGs upregulated in R. a. himalayanus were also mainly involved in a protective mechanism for auditory hair cells. Using 3 different methods of AS analysis, we identified several candidate alternatively spliced genes (ASGs) that expressed different isoforms which may be associated with acoustic divergence of the 2 subspecies. We observed significantly less overlap than expected between DEGs and ASGs, supporting complementary roles of differential gene expression and AS in generating phenotypic variations. Overall, our study highlights the importance of a combination of short-read and long-read RNA-seq data in examining the regulation of gene expression changes responsible for phenotypic variations.

AD - School of Ecological and Environmental Sciences, East China Normal University, Shanghai 200062, China.

AN - 39463690

AU - Li, Q.

AU - Wu, J.

AU - Mao, X.

C1 - PubMed

C2 - PMC11502156

DA - Oct

DO - 10.1093/cz/zoad045

DP - NLM

ET - 2024/10/28

IS - 5

KW - alternative splicing

lncRNAs

noise-induced hearing loss

phenotypic variation

repair of hair cell

LA - eng

N1 - 2396-9814

Li, Qianqian

Orcid: 0000-0001-9280-0563

Wu, Jianyu

Mao, Xiuguang

Journal Article

England

Curr Zool. 2023 Sep 30;70(5):575-588. doi: 10.1093/cz/zoad045. eCollection 2024 Oct.

PY - 2024

SN - 1674-5507 (Print)

1674-5507

SP - 575-588

ST - The roles of different gene expression regulators in acoustic variation in the intermediate horseshoe bat revealed by long-read and short-read RNA sequencing data

T2 - Curr Zool

TI - The roles of different gene expression regulators in acoustic variation in the intermediate horseshoe bat revealed by long-read and short-read RNA sequencing data

VL - 70

ID - 39707

ER -

TY - JOUR

AB - The inner ear sensory epithelium consists of two major types of cells: hair cells (HCs) and supporting cells (SCs). Critical functions of HCs in the perception of mechanical stimulation and mechanosensory transduction have long been elucidated. SCs are indispensable components of the sensory epithelia, and they maintain the structural integrity and ionic environment of the inner ear. Once delicate inner ear epithelia sustain injuries (for example, due to ototoxic drugs or noise exposure), SCs respond immediately to serve as repairers of the epithelium and as adapters to become HC progenitors, aiming at morphological and functional recovery of the inner ear. This regenerative process is extensive in non-mammals, but is limited in the mammalian inner ear, especially in the mature cochlea. This review aimed to discuss the important roles of SCs in the repair of the mammalian inner ear.

AD - Department of Otolaryngology-Head and Neck Surgery, Beijing Friendship Hospital, Capital Medical University, Beijing, China.

Clinical Center for Hearing Loss, Capital Medical University, Beijing, China.

AN - 39776546

AU - Guo, J. Y.

AU - Xu, J. Y.

AU - Gong, S. S.

AU - Wang, G. P.

C1 - PubMed

C2 - PMC11701326

DA - Oct

DO - 10.1016/j.joto.2024.07.007

DP - NLM

ET - 2025/01/08

IS - 4

LA - eng

N1 - 2524-1753

Guo, Jing-Ying

Xu, Jun-Yi

Gong, Shu-Sheng

Wang, Guo-Peng

Journal Article

Review

China

J Otol. 2024 Oct;19(4):234-240. doi: 10.1016/j.joto.2024.07.007. Epub 2024 Oct 19.

PY - 2024

SN - 1672-2930 (Print)

1672-2930

SP - 234-240

ST - Roles of supporting cells in the maintenance and regeneration of the damaged inner ear: A literature review

T2 - J Otol

TI - Roles of supporting cells in the maintenance and regeneration of the damaged inner ear: A literature review

VL - 19

ID - 38922

ER -

TY - JOUR

AB - Sensorineural hearing loss a result from hair cell damage, which is irreversible in mammals owing to the lack of hair cell regeneration, but recent researches have shown that Lgr5+ supporting cells are progenitors capable of regenerating hair cells. RPS14 (ribosomal protein S14) is a 40S ribosomal subunit component and is associated with erythrocyte differentiation, and in this study, we used a novel adeno-associated virus-inner ear system to upregulate Rps14 expression in cultured hair cell progenitors and observed an enhancement on their ability to proliferate and differentiate into hair cells. Similarly, Rps14 overexpression in the mice cochlea could promote supporting cells proliferation by activating the Wnt signalling pathway. In addition, over-expressing Rps14 induced hair cells regeneration in the organ of Corti, and lineage tracing showed that the new hair cells had transformed from Lgr5+ progenitors. In conclusion, our analysis reveals the potential role of Rps14 in driving hair cell regeneration in mammalian. © 2023 The Authors. Cell Proliferation published by Beijing Institute for Stem Cell and Regenerative Medicine and John Wiley & Sons Ltd.

AD - Health Management Center, Sichuan Provincial People's Hospital, University of Electronic Science and Technology of China, Chengdu, China

Sichuan Provincial Key Laboratory for Human Disease Gene Study and Department of Laboratory Medicine, Sichuan Provincial People's Hospital, University of Electronic Science and Technology of China, Chengdu, China

Research Unit for Blindness Prevention of the Chinese Academy of Medical Sciences (2019RU026), Sichuan Academy of Medical Sciences and Sichuan Provincial People's Hospital, Sichuan, Chengdu, China

State Key Laboratory of Bioelectronics, Department of Otolaryngology Head and Neck Surgery, Zhongda Hospital, School of Life Sciences and Technology, Advanced Institute for Life and Health, Jiangsu Province High-Tech Key Laboratory for Bio-Medical Research, Southeast University, Nanjing, 210096, China

Department of Plastic and Reconstruction surgery, Zhongda Hospital, Southeast University, 87 Dingjiaqiao Street, Jiangsu Province, Nanjing, China

Department of Otolaryngology, Guangdong Provincial People's Hospital (Guangdong Academy of Medical Sciences), Southern Medical University, Guangzhou, 510080, China

School of Medicine, South China University of Technology, Guangzhou, 510006, China

Department of Otolaryngology Head and Neck Surgery, Sichuan Provincial People's Hospital, University of Electronic Science and Technology of China, Chengdu, China

Co-Innovation Center of Neuroregeneration, Nantong University, Nantong, 226001, China

Institute for Stem Cell and Regeneration, Chinese Academy of Science, Beijing, China

Beijing Key Laboratory of Neural Regeneration and Repair, Capital Medical University, 100069 Beijing, China

AU - Xu, C.

AU - Qi, J.

AU - Hu, X.

AU - Zhang, L.

AU - Sun, Q.

AU - Li, N.

AU - Chen, X.

AU - Guo, F.

AU - Wu, P.

AU - Shi, Y.

AU - Chai, R.

C1 - Scopus

C2 - 36977657

C7 - e13458

DB - Scopus

DO - 10.1111/cpr.13458

IS - 5

J2 - Cell Prolif.

KW - Animals

Animals, Newborn

Cell Differentiation

Cell Proliferation

Ear, Inner

Hair Cells, Auditory

Mammals

Mice

Receptors, G-Protein-Coupled

Up-Regulation

beta catenin

enhanced green fluorescent protein

ribosomal protein s14

ribosome protein

RNA

unclassified drug

Wnt protein

G protein coupled receptor

animal cell

Article

basilar membrane

cell count

cell density

cell regeneration

cell structure

cellular distribution

cochlea

cochlea fenestra

comparative study

controlled study

Corti organ

cytoplasm

Deiters cell

differentiation

expression vector

fluorescence

fluorescence activated cell sorting

fluorescence intensity

genetic transfection

hair cell (inner ear)

immunofluorescence assay

inner ear

inner pillar cell

mouse

newborn

nonhuman

nuclear localization signal

stem cell culture

upregulation

Western blotting

Wnt signaling

animal

cochlear hair cell

mammal

metabolism

LA - English

M3 - Article

N1 - Export Date: 21 May 2026; Cited By: 18; Correspondence Address: Y. Shi; Health Management Center, Sichuan Provincial People's Hospital, University of Electronic Science and Technology of China, Chengdu, China; email: shiyi1614@126.com; J. Qi; State Key Laboratory of Bioelectronics, Department of Otolaryngology Head and Neck Surgery, Zhongda Hospital, School of Life Sciences and Technology, Advanced Institute for Life and Health, Jiangsu Province High-Tech Key Laboratory for Bio-Medical Research, Southeast University, Nanjing, 210096, China; email: jieyuqi@seu.edu.cn; R. Chai; State Key Laboratory of Bioelectronics, Department of Otolaryngology Head and Neck Surgery, Zhongda Hospital, School of Life Sciences and Technology, Advanced Institute for Life and Health, Jiangsu Province High-Tech Key Laboratory for Bio-Medical Research, Southeast University, Nanjing, 210096, China; email: renjiec@seu.edu.cn; P. Wu; Department of Otolaryngology, Guangdong Provincial People's Hospital, Guangdong Academy of Medical Sciences, Southern Medical University, Guangzhou, 510080, China; email: linwupeina@hotmail.com; CODEN: CPROE

PY - 2023

SN - 09607722 (ISSN)

ST - Rps14 upregulation promotes inner ear progenitor proliferation and hair cell regeneration in the neonatal mouse cochlea

T2 - Cell Proliferation

TI - Rps14 upregulation promotes inner ear progenitor proliferation and hair cell regeneration in the neonatal mouse cochlea

UR - https://www.scopus.com/pages/publications/85151990157?origin=resultslist

VL - 56

ID - 39847

ER -

TY - JOUR

AB - BACKGROUND AND OBJECTIVES: Sensorineural hearing loss (SNHL) in children is associated with neurocognitive morbidity. The cause of SNHL is a loss of hair cells in the organ of Corti. There are currently no reparative treatments for SNHL. Numerous studies suggest that cord blood mononuclear cells (human umbilical cord blood, hUCB) allow at least partial restoration of SNHL by enabling repair of a damaged organ of Corti. Our objective is to determine if hUCB is a safe treatment for moderate to severe acquired SNHL in children. Subjects and. METHODS: Eleven children aged 6 months to 6 years with moderate to severe acquired SNHL were treated with intravenous autologous hUCB. The cell dose ranged from 8 to 30 million cells/kg body weight. Safety was assessed by measuring systemic hemodynamics during hUCB infusion. Infusion-related toxicity was evaluated by measuring neurologic, hepatic, renal and pulmonary function before and after infusion. Auditory function, auditory verbal language assessments and MRI with diffusion tensor imaging (DTI) were obtained before and after treatment. RESULTS: All patients survived, and there were no adverse events. No infusionrelated changes in hemodynamics occurred. No infusion-related toxicity was recorded. Five subjects experienced a reduction in auditory brainstem response (ABR) thresholds. Four of those 5 subjects also experienced an improvement in cochlear nerve latencies. Comparison of MRI with DTI sequences obtained before and after treatment revealed increased fractional anisotropy in the primary auditory cortex in three of five subjects with reduced ABR thresholds. Statistically significant (p<0.05) reductions in ABR thresholds were identified. CONCLUSIONS: TIntravenous hUCB is feasible and safe in children with SNHL.

AD - Little HEARoes & Clarke Schools for Hearing and Speech, Orlando, FL, USA.

Department of Audiology and Speech-Language Pathology, University of North Texas, Denton, TA, USA.

Florida Hospital for Children, Orlando, FL, USA.

Florida Radiology Institute, Orlando, FL, USA.

Little HEARoes, LLC, Winston Salem, NC, USA.

Department of Biostatistics, School of Public Health, University of North Texas, Fort Worth, TA, USA.

Florida Hospital Celebration, Celebration, FL, USA.

Shriner's Hospital for Children, Houston, TA, USA.

Department of Neurosurgery, University of Central Florida College of Medicine, Orlando, FL, USA.

AN - 30126263

AU - Baumgartner, L. S.

AU - Moore, E.

AU - Shook, D.

AU - Messina, S.

AU - Day, M. C.

AU - Green, J.

AU - Nandy, R.

AU - Seidman, M.

AU - Baumgartner, J. E.

C1 - PubMed

C2 - PMC6233943

DA - Oct

DO - 10.7874/jao.2018.00115

DP - NLM

ET - 2018/08/22

IS - 4

KW - Auditory brainstem response

Diffusion tensor imaging

Human umbilical cord blood

Mesenchymal stem cells

Mononuclear fraction

Otoacoustic emission

Sensorineural hearing loss

LA - eng

N1 - 2384-1710

Baumgartner, Linda S

Moore, Ernest

Shook, David

Messina, Steven

Day, Mary Clare

Green, Jennifer

Nandy, Rajesh

Seidman, Michael

Baumgartner, James E

Cord Blood Registry®/

Wayne Densch Charities/

Journal Article

Korea (South)

J Audiol Otol. 2018 Oct;22(4):209-222. doi: 10.7874/jao.2018.00115. Epub 2018 Aug 22.

PY - 2018

SN - 2384-1621 (Print)

SP - 209-222

ST - Safety of Autologous Umbilical Cord Blood Therapy for Acquired Sensorineural Hearing Loss in Children

T2 - J Audiol Otol

TI - Safety of Autologous Umbilical Cord Blood Therapy for Acquired Sensorineural Hearing Loss in Children

VL - 22

ID - 39820

ER -

TY - JOUR

AB - Sensory hair cells are specialized mechanotransductive receptors required for hearing and vestibular function. Loss of hair cells in humans and other mammals is permanent and causes reduced hearing and balance. In the early 1980’s, it was shown that hair cells continue to be added to the inner ear sensory epithelia in cartilaginous and bony fishes. Soon thereafter, hair cell regeneration was documented in the chick cochlea following acoustic trauma. Since then, research using chick and other avian models has led to great insights into hair cell death and regeneration. However, with the rise of the zebrafish as a model organism for studying disease and developmental processes, there has been an increased interest in studying sensory hair cell death and regeneration in its lateral line and inner ears. Advances derived from studies in zebrafish and other fish species include understanding the effect of ototoxins on hair cells and finding otoprotectants to mitigate ototoxin damage, the role of cellular proliferation vs. direct transdifferentiation during hair cell regeneration, and elucidating cellular pathways involved in the regeneration process. This review will summarize research on hair cell death and regeneration using fish models, indicate the potential strengths and weaknesses of these models, and discuss several emerging areas of future studies. © 2015, Frontiers Research Foundation. All rights reserved.

AD - Department of Biology, Western Kentucky University, Bowling Green, KY, United States

Department of Genetics and Genome Sciences, University of Connecticut Health Center, Farmington, CT, United States

AU - Monroe, J. D.

AU - Rajadinakaran, G.

AU - Smith, M. E.

C1 - Scopus

DB - Scopus

DO - 10.3389/fncel.2015.00131

IS - APR

J2 - Front. Cell. Neurosci.

KW - Acoustic trauma

Cell death

Hair cell

Lateral line

Ototoxicity

Regeneration

Teleost

Zebrafish

aminoglycoside

aminoglycoside derivative

antibiotic agent

antineoplastic agent

cisplatin

epicatechin

gentamicin

kanamycin

neomycin

Notch receptor

quinoline derivative

transcription factor Sox2

Wnt protein

Article

cell cycle

cell damage

cell differentiation

cell function

cell proliferation

cell regeneration

cell structure

cell transdifferentiation

gene expression

inner ear

lateral line system

major histocompatibility complex

noise injury

nonhuman

reinnervation

TUNEL assay

LA - English

M3 - Article

N1 - Export Date: 21 May 2026; Cited By: 67

PY - 2015

SN - 16625102 (ISSN)

ST - Sensory hair cell death and regeneration in fishes

T2 - Frontiers in Cellular Neuroscience

TI - Sensory hair cell death and regeneration in fishes

UR - https://www.scopus.com/pages/publications/84929589988?origin=resultslist

VL - 9

ID - 39862

ER -

TY - JOUR

AB - Sensory hair cells are mechanoreceptors of the auditory and vestibular systems and are crucial for hearing and balance. In adult mammals, auditory hair cells are unable to regenerate, and damage to these cells results in permanent hearing loss. By contrast, hair cells in the chick cochlea and the zebrafish lateral line are able to regenerate, prompting studies into the signaling pathways, morphogen gradients and transcription factors that regulate hair cell development and regeneration in various species. Here, we review these findings and discuss how various signaling pathways and factors function to modulate sensory hair cell development and regeneration. By comparing and contrasting development and regeneration, we also highlight the utility and limitations of using defined developmental cues to drive mammalian hair cell regeneration. © 2015. Published by The Company of Biologists Ltd.

AD - Department of Otolaryngology – Head and Neck Surgery, Stanford University School of Medicine, Stanford, 94305, CA, United States

AU - Atkinson, P. J.

AU - Najarro, E. H.

AU - Sayyid, Z. N.

AU - Cheng, A. G.

C1 - Scopus

C2 - 25922522

DB - Scopus

DO - 10.1242/dev.114926

IS - 9

J2 - Development

KW - Atoh1

Cdkn1b

FGF

Notch

P27<sup>kip1</sup>

Shh

Wnt

Age Factors

Animals

Basic Helix-Loop-Helix Transcription Factors

Cell Cycle

Cell Differentiation

Cell Lineage

Chick Embryo

Ear, Inner

Hair Cells, Ampulla

Lateral Line System

Mechanoreceptors

Mice

Models, Biological

Morphogenesis

Regeneration

Species Specificity

Zebrafish

Danio rerio

Mammalia

fibroblast growth factor

morphogen

Notch receptor

sonic hedgehog protein

transcription factor

transcription factor Atoh1

unclassified drug

Wnt protein

Atoh1 protein, mouse

basic helix loop helix transcription factor

cell communication

cell maturation

cell proliferation

cell regeneration

chick

clinical trial (topic)

cochlea

developmental stage

hair cell

hearing

hearing impairment

human

mitosis

nonhuman

nuclear reprogramming

organogenesis

priority journal

regenerative medicine

regulatory mechanism

Review

sense organ

sensory nerve cell

signal transduction

Wnt signaling pathway

zebra fish

age

animal

biological model

cytology

embryology

inner ear

mechanoreceptor

mouse

physiology

species difference

LA - English

M3 - Review

N1 - Export Date: 21 May 2026; Cited By: 132; CODEN: DEVPE

PY - 2015

SN - 09501991 (ISSN)

SP - 1561-1571

ST - Sensory hair cell development and regeneration: Similarities and differences

T2 - Development (Cambridge)

TI - Sensory hair cell development and regeneration: Similarities and differences

UR - https://www.scopus.com/pages/publications/84928603760?origin=resultslist

VL - 142

ID - 39848

ER -

TY - JOUR

AB - A hallmark of the nervous system is the presence of precise patterns of connections between different types of neurons. Many mechanisms can be used to establish specificity, including homophilic adhesion and synaptic refinement, but the range of strategies used across the nervous system remains unclear. To broaden the understanding of how neurons find their targets, we studied the developing murine cochlea, where two classes of spiral ganglion neurons (SGNs), type I and type II, navigate together to the sensory epithelium and then diverge to contact inner hair cells (IHCs) or outer hair cells (OHCs), respectively. Neurons with type I and type II morphologies are apparent before birth, suggesting that target selection might be accomplished by excluding type I processes from the OHC region. However, because type I processes appear to overshoot into type II territory postnatally, specificity may also depend on elimination of inappropriate synapses. To resolve these differences, we analyzed the morphology and dynamic behaviors of individual fibers and their branches as they interact with potential partners. We found that SGN processes continue to be segregated anatomically in the postnatal cochlea. Although type I-like fibers branched locally, few branches contacted OHCs, arguing against synaptic elimination. Instead, time-lapse imaging studies suggest a prominent role for retraction, first positioning processes to the appropriate region and then corralling branches during a subsequent period of exuberant growth and refinement. Thus, sequential stages of retraction can help to achieve target specificity, adding to the list of mechanisms available for sculpting neural circuits. SIGNIFICANCE STATEMENT: During development, different types of neurons must form connections with specific synaptic targets, thereby creating the precise wiring diagram necessary for adult function. Although studies have revealed multiple mechanisms for target selection, we still know little about how different strategies are used to produce each circuit's unique pattern of connectivity. Here we combined neurite-tracing and time-lapse imaging to define the events that lead to the simple binary wiring specificity of the cochlea. A better understanding of how the cochlea is innervated will broaden our knowledge of target selection across the nervous system, offer new insights into the developmental origins of deafness, and guide efforts to restore connectivity in the damaged cochlea.

AD - Department of Neurobiology, Harvard Medical School, Boston, Massachusetts 02115.

Department of Neurobiology, Harvard Medical School, Boston, Massachusetts 02115 Lisa_Goodrich@hms.harvard.edu.

AN - 26658872

AU - Druckenbrod, N. R.

AU - Goodrich, L. V.

C1 - PubMed

C2 - PMC4682787

DA - Dec 9

DO - 10.1523/jneurosci.2236-15.2015

DP - NLM

ET - 2015/12/15

IS - 49

KW - Age Factors

Analysis of Variance

Animals

Animals, Newborn

Basic Helix-Loop-Helix Proteins/genetics/metabolism

Cochlea/*anatomy & histology/*innervation

Embryo, Mammalian

Hair Cells, Auditory, Inner/*physiology

Hair Cells, Auditory, Outer/*physiology

Imaging, Three-Dimensional

In Vitro Techniques

Luminescent Proteins/genetics/metabolism

Mice

Mice, Transgenic

Models, Biological

Nerve Tissue Proteins/genetics/metabolism

Neural Pathways/*physiology

Organ Culture Techniques

Spiral Ganglion/*cytology

Time-Lapse Imaging

auditory

cochlea

cochlear development

refinement

spiral ganglion neuron

target selection

LA - eng

N1 - 1529-2401

Druckenbrod, Noah R

Goodrich, Lisa V

R01 DC009223/DC/NIDCD NIH HHS/United States

T32 NS007484/NS/NINDS NIH HHS/United States

Journal Article

Research Support, N.I.H., Extramural

Research Support, Non-U.S. Gov't

Video-Audio Media

United States

J Neurosci. 2015 Dec 9;35(49):16221-35. doi: 10.1523/JNEUROSCI.2236-15.2015.

PY - 2015

SN - 0270-6474 (Print)

0270-6474

SP - 16221-35

ST - Sequential Retraction Segregates SGN Processes during Target Selection in the Cochlea

T2 - J Neurosci

TI - Sequential Retraction Segregates SGN Processes during Target Selection in the Cochlea

VL - 35

ID - 39762

ER -

TY - JOUR

AB - Loss of hair cells from vestibular epithelium results in balance dysfunction. The current therapeutic regimen for vestibular diseases is limited. Upon injury or Atoh1 overexpression, hair cell replacement occurs rapidly in the mammalian utricle, suggesting a promising approach to induce vestibular hair cell regeneration. In this study, we applied simultaneous gentamicin-mediated hair cell ablation and Atoh1 overexpression to induce neonatal utricular hair cell formation in vitro. We confirmed that type I hair cells were the primary targets of gentamicin. Furthermore, injury and Atoh1 overexpression promoted hair cell regeneration in a timely and efficient manner through robust viral transfection. Hair cells regenerated with type II characteristics in the striola and type I/II characteristics in non-sensory regions. Rare EdU+/myosin7a+ cells in sensory regions and robust EdU+/myosin7a+ signals in ectopic regions indicate that transdifferentiation of supporting cells in situ, and mitosis and differentiation of non-sensory epithelial cells in ectopic regions, are sources of regenerative hair cells. Distinct regeneration patterns in in situ and ectopic regions suggested robust plasticity of vestibular non-sensory epithelium, generating more developed hair cell subtypes and thus providing a promising stem cell-like source of hair cells. These findings suggest that simultaneously causing injury and overexpressing Atoh1 promotes hair cell regeneration efficacy and maturity, thus expanding the understanding of ectopic plasticity in neonatal vestibular organs. © 2020 The Author(s)

AD - ENT Institute and Department of Otorhinolaryngology, Eye & ENT Hospital, Fudan University, Shanghai, 200031, China

NHC Key Laboratory of Hearing Medicine (Fudan University), Shanghai, 200031, China

AU - Qian, X.

AU - Ma, R.

AU - Wang, X.

AU - Xu, X.

AU - Yang, J.

AU - Chi, F.

AU - Ren, D.

C1 - Scopus

C2 - 33279477

C7 - 112395

DB - Scopus

DO - 10.1016/j.yexcr.2020.112395

IS - 1

J2 - Exp. Cell Res.

KW - Atoh1

Gentamicin

Hair cell

Regeneration

Utricle

Animals

Basic Helix-Loop-Helix Transcription Factors

Gentamicins

Hair Cells, Vestibular

Mice

Mice, Inbred C57BL

Saccule and Utricle

atonal homolog 1

basic helix loop helix transcription factor

myosin VIIa

unclassified drug

Atoh1 protein, mouse

animal cell

animal experiment

animal tissue

Article

cell differentiation

cell regeneration

cell transdifferentiation

controlled study

epithelium cell

hair cell (inner ear)

immunocytochemistry

in vitro study

marker gene

mitosis

mouse

neuroepithelium

newborn

nonhuman

priority journal

quantitative analysis

sustentacular cell

upregulation

viral gene delivery system

animal

C57BL mouse

drug effects

genetics

metabolism

pathology

vestibular hair cell

vestibule

LA - English

M3 - Article

N1 - Export Date: 21 May 2026; Cited By: 9; Correspondence Address: D. Ren; Department of Otorhinolaryngology, Eye & ENT Hospital, Fudan University, Shanghai, 83 Fenyang Road, Xuhui, 200031, China; email: dongdongren@fudan.edu.cn; F. Chi; Department of Otorhinolaryngology, Eye & ENT Hospital, Fudan University, Shanghai, 83 Fenyang Road, Xuhui, 200031, China; email: Chifanglu@126.com; J. Yang; Department of Otorhinolaryngology, Eye & ENT Hospital, Fudan University, Shanghai, 83 Fenyang Road, Xuhui, 200031, China; email: yangjuanmei1982@126.com; CODEN: ECREA

PY - 2021

SN - 00144827 (ISSN)

ST - Simultaneous gentamicin-mediated damage and Atoh1 overexpression promotes hair cell regeneration in the neonatal mouse utricle

T2 - Experimental Cell Research

TI - Simultaneous gentamicin-mediated damage and Atoh1 overexpression promotes hair cell regeneration in the neonatal mouse utricle

UR - https://www.scopus.com/pages/publications/85097449796?origin=resultslist

VL - 398

ID - 39849

ER -

TY - JOUR

AB - Hearing loss caused by the death of cochlear hair cells (HCs) might be restored through regeneration from supporting cells (SCs) via dedifferentiation and proliferation, as observed in birds. In a previous report, ERBB2 activation in a subset of cochlear SCs promoted widespread down-regulation of SOX2 in neighboring cells, proliferation, and the differentiation of HC-like cells. Here we analyze single cell transcriptomes from neonatal mouse cochlear SCs with activated ERBB2, with the goal of identifying potential secreted effectors. ERBB2 induction in vivo generated a new population of cells with de novo expression of a gene network. Called small integrin-binding ligand n-linked glycoproteins (SIBLINGs), these ligands and their regulators can alter NOTCH signaling and promote cell survival, proliferation, and differentiation in other systems. We validated mRNA expression of network members, and then extended our analysis to older stages. ERBB2 signaling in young adult SCs also promoted protein expression of gene network members. Furthermore, we found proliferating cochlear cell aggregates in the organ of Corti. Our results suggest that ectopic activation of ERBB2 signaling in cochlear SCs can alter the microenvironment, promoting proliferation and cell rearrangements. Together these results suggest a novel mechanism for inducing stem cell-like activity in the adult mammalian cochlea.

AD - Department of Neuroscience, University of Rochester School of Medicine and Dentistry, Rochester, NY, United States.

Department of Biology, University of Rochester, Rochester, NY, United States.

Genomic Research Center, University of Rochester School of Medicine and Dentistry, Rochester, NY, United States.

AN - 36687526

AU - Piekna-Przybylska, D.

AU - Na, D.

AU - Zhang, J.

AU - Baker, C.

AU - Ashton, J. M.

AU - White, P. M.

C1 - PubMed

C2 - PMC9853549

DO - 10.3389/fncel.2022.1096872

DP - NLM

ET - 2023/01/24

KW - Cd44

Erbb2

Sibling

candidate pathway

cochlear regeneration

single cell sequencing

commercial or financial relationships that could be construed as a potential

conflict of interest.

LA - eng

N1 - 1662-5102

Piekna-Przybylska, Dorota

Na, Daxiang

Zhang, Jingyuan

Baker, Cameron

Ashton, John M

White, Patricia M

R01 DC014261/DC/NIDCD NIH HHS/United States

Journal Article

Switzerland

Front Cell Neurosci. 2023 Jan 6;16:1096872. doi: 10.3389/fncel.2022.1096872. eCollection 2022.

PY - 2022

SN - 1662-5102 (Print)

1662-5102

SP - 1096872

ST - Single cell RNA sequencing analysis of mouse cochlear supporting cell transcriptomes with activated ERBB2 receptor indicates a cell-specific response that promotes CD44 activation

T2 - Front Cell Neurosci

TI - Single cell RNA sequencing analysis of mouse cochlear supporting cell transcriptomes with activated ERBB2 receptor indicates a cell-specific response that promotes CD44 activation

VL - 16

ID - 39083

ER -

TY - JOUR

AB - Notch signaling is active during the development of mosaic epithelial sheets and during their turnover and regeneration. After the loss of hair cells in the mosaic sheet of the vestibular sensory epithelium, new hair cells can be spontaneously generated by transdifferentiation of supporting cells. This regenerative process involves downregulation of the Hes5 gene and is known to be limited and incomplete, especially when the lesion is severe. Here, we test whether further downregulation of Hes5 gene accomplished by the use of siRNA after a severe lesion induced by an aminoglycoside in the mouse utricle can enhance the transdifferentiation of supporting cells and lead to the increased production of new hair cells. We demonstrate that Hes5 levels in the utricle decreased after the application of siRNA and that the number of hair cells in these utricles was significantly larger than following control treatment. The data suggest that siRNA technology may be useful for inducing repair and regeneration in the inner ear and that the Notch signaling pathway is a potentially useful target for specific gene expression inhibition. © The American Society of Gene & Cell Therapy.

AD - KHRI, Otolaryngology, Head and Neck Surgery, University of Michigan, Ann Arbor, MI 48109-5648, United States

Department of ORL-HNS, College of Medicine, Dankook University, Cheonan, South Korea

Oregon Hearing Research Center, Oregon Health and Science University, Portland, OR, United States

Quark Pharmaceuticals, Inc., Fremont, CA, United States

AU - Jung, J. Y.

AU - Avenarius, M. R.

AU - Adamsky, S.

AU - Alpert, E.

AU - Feinstein, E.

AU - Raphael, Y.

C1 - Scopus

DB - Scopus

DO - 10.1038/mt.2013.18

IS - 4

J2 - Mol. Ther.

KW - aminoglycoside

Notch receptor

small interfering RNA

transcription factor HES 5

animal cell

animal experiment

animal tissue

article

cell regeneration

cell transdifferentiation

controlled study

down regulation

female

gene expression

gene targeting

hair cell

inner ear

mouse

neuroepithelium

nonhuman

utricle

vestibular disorder

vestibular system

LA - English

M3 - Article

N1 - Export Date: 21 May 2026; Cited By: 33; Correspondence Address: Y. Raphael; KHRI, Otolaryngology, Head and Neck Surgery, University of Michigan, Ann Arbor, MI 48109-5648, United States; email: yoash@umich.edu; CODEN: MTOHC

PY - 2013

SN - 15250016 (ISSN)

SP - 834-841

ST - SiRNA targeting Hes5 augments hair cell regeneration in aminoglycoside-damaged mouse utricle

T2 - Molecular Therapy

TI - SiRNA targeting Hes5 augments hair cell regeneration in aminoglycoside-damaged mouse utricle

UR - https://www.scopus.com/pages/publications/84878572295?origin=resultslist

VL - 21

ID - 39857

ER -

TY - JOUR

AB - Chick hair cells display calcium (Ca(2+))-sensitive spontaneous action potentials during development and regeneration. The role of this activity is unclear but thought to be involved in establishing proper synaptic connections and tonotopic maps, both of which are instrumental to normal hearing. Using an electrophysiological approach, this work investigated the functional expression of Ca(2+)-sensitive potassium [I(K(Ca))] currents and their role in spontaneous electrical activity in the developing and regenerating hair cells (HCs) in the chick basilar papilla. The main I(K(Ca)) in developing and regenerating chick HCs is an SK current, based on its sensitivity to apamin. Analysis of the functional expression of SK current showed that most dramatic changes occurred between E8 and E16. Specifically, there is a developmental downregulation of the SK current after E16. The SK current gating was very sensitive to the availability of intracellular Ca(2+) but showed very little sensitivity to T-type voltage-gated Ca(2+) channels, which are one of the hallmarks of developing and regenerating hair cells. Additionally, apamin reduced the frequency of spontaneous electrical activity in HCs, suggesting that SK current participates in patterning the spontaneous electrical activity of HCs.

AD - Center for Neuroscience, University of California, Davis, Davis, CA, United States.

Sensory Neuroscience Research Group, School of Pharmacy and Biomolecular Sciences, University of Brighton, Brighton, United Kingdom.

Brighton and Sussex Medical School, University of Sussex, Brighton, United Kingdom.

AN - 35069114

AU - Levic, S.

C1 - PubMed

C2 - PMC8770932

DO - 10.3389/fncel.2021.766264

DP - NLM

ET - 2022/01/25

KW - SK currents

T-type calcium channels

basilar papilla

chick

development

hair cells

intracellular calcium

regeneration

commercial or financial relationships that could be construed as a potential

conflict of interest.

LA - eng

N1 - 1662-5102

Levic, Snezana

MR/N004299/1/MRC_/Medical Research Council/United Kingdom

MR/W028956/1/MRC_/Medical Research Council/United Kingdom

Journal Article

Switzerland

Front Cell Neurosci. 2022 Jan 6;15:766264. doi: 10.3389/fncel.2021.766264. eCollection 2021.

PY - 2021

SN - 1662-5102 (Print)

1662-5102

SP - 766264

ST - SK Current, Expressed During the Development and Regeneration of Chick Hair Cells, Contributes to the Patterning of Spontaneous Action Potentials

T2 - Front Cell Neurosci

TI - SK Current, Expressed During the Development and Regeneration of Chick Hair Cells, Contributes to the Patterning of Spontaneous Action Potentials

VL - 15

ID - 39191

ER -

TY - JOUR

AB - POU4 homologs are involved in the development of sensory cell types across diverse species, including cnidarians, ascidians, and mammals. Whether these developmental regulators are redeployed during adult tissue maintenance and regeneration remains an open question in regenerative biology. Here, we investigated the role of the Schmidtea mediterranea BRN3/POU4 homolog, Smed-pou4-2 (pou4-2), in the regeneration of mechanosensory neurons. We found that pou4-2 is regulated by the SoxB1 homolog soxB1-2 and is expressed in a distinct population of ciliated sensory cells that detect water flow. Transcriptomic analysis of pou4-2-deficient planarians revealed enrichment for conserved genes associated with human auditory and vestibular function, suggesting that planarian rheosensory neurons share molecular features with mammalian inner ear hair cells. Expression of these conserved genes was significantly reduced following RNAi-mediated knockdown of pou4-2. To determine whether these transcriptional changes had functional consequences, we assessed the impact of pou4-2 knockdown on sensory function. pou4-2 RNAi resulted in impaired mechanosensation in both uninjured and regenerating planarians. Together with the loss of terminal differentiation markers in mechanosensory neurons, these findings identify Smed-pou4-2 as a key regulator of mechanosensory neuron identity in planarians and support the idea that conserved sensory specification programs are redeployed during adult tissue regeneration.

AD - Department of Biology, San Diego State University, San Diego, United States.

Center for Computational Biology and Bioinformatics, University of California, San Diego, San Diego, United States.

AN - 41252183

AU - McCubbin, R. A.

AU - Auwal, M. A.

AU - Wang, S.

AU - Alvarez Zepeda, S.

AU - Sasik, R.

AU - Zeller, R. W.

AU - Ross, K. G.

AU - Zayas, R. M.

C1 - PubMed

C2 - PMC12626421

DA - Nov 18

DO - 10.7554/eLife.107718

DP - NLM

ET - 2025/11/18

KW - Animals

*Planarians/physiology/genetics

*Regeneration

*Mechanoreceptors/physiology

POU4 transcription factor

ciliated sensory neurons

developmental biology

gene regulatory networks

mechanosensation

planarian

regenerative medicine

stem cells

terminal selector

LA - eng

N1 - 2050-084x

McCubbin, Ryan A

Auwal, Mohammad A

Wang, Shengzhou

Alvarez Zepeda, Sarai

Orcid: 0009-0003-8740-6280

Sasik, Roman

Zeller, Robert W

Ross, Kelly G

Orcid: 0000-0002-5940-8778

Zayas, Ricardo M

Orcid: 0000-0002-6272-0519

EDUC4-12813/California Institute for Regenerative Medicine/

NIH R01GM135657/NH/NIH HHS/United States

R01 GM135657/GM/NIGMS NIH HHS/United States

IOS 557448/U.S. National Science Foundation/

IOS 1938531/U.S. National Science Foundation/

Journal Article

England

Elife. 2025 Nov 18;14:RP107718. doi: 10.7554/eLife.107718.

PY - 2025

SN - 2050-084x

ST - Smed-pou4-2 regulates mechanosensory neuron regeneration and function in planarians

T2 - Elife

TI - Smed-pou4-2 regulates mechanosensory neuron regeneration and function in planarians

VL - 14

ID - 38885

ER -

TY - JOUR

AB - Studies have showed that transplanted stem cells in the inner ear won't regenerate to replace the damaged sensory hair cells. They can spontaneously differentiate into mesenchymal cells and fibrocytes in the damaged inner ear. Only mature sensory cells of MSCs-derived possess the great potency for cell transplantation in the treatment of sensorineural hearing loss. So, we try to establish an efficient generation of the glutamatergic sensory neural phenotype for the cell transplantation of the hearing loss. We isolated MSCs from femoral and tibial bones according to their adherence to culture dishes. After purification, proliferation, and passaged, cells became homogeneous in appearance, showing more uniformity and grew in a monolayer with a typical spindle-shape morphology. The cell surface markers were assessed using FACS to characterize the isolated cells. For neural induction to harvest the glutamatergic sensory neurons, passage 3 MSCs were incubated with preinduced medium for 24 hr, and neural-induced medium for an additional 14 days. The cells exhibit a typical neural shape. RT-PCR analysis indicated that the mRNA levels of the neural cell marker nestin, Tau, MAP-2, β-tubulin III, GluR-3, and GluR-4 were higher compared with primary MSCs. Immunohistochemistry and western-blotting proofed that nestin, MAP-2, β-tubulin III, and GluR-4 proteins indeed exhibit their expression difference in the induced cells compared to the MSCs. We show an efficient protocol by the combined applications of Sonic Hedgehog (Shh) and Retinoic Acid (RA) to induce MSCs to differentiate into the glutamatergic sensory neuron which were identified from the morphological, biochemical, and molecular characteristics.

AD - a Department of Human Anatomy, College of Basic Medical Sciences , Second Military Medical University , Shanghai , PR China.

AN - 24547891

AU - Yu, Z.

AU - Wu, S.

AU - Liu, Z.

AU - Lin, H.

AU - Chen, L.

AU - Yuan, X.

AU - Zhang, Z.

AU - Liu, F.

AU - Zhang, C.

C1 - PubMed

DO - 10.1080/15321819.2014.889025

DP - NLM

ET - 2014/02/20

IS - 1

KW - Animals

Cell Differentiation/drug effects/immunology

Hedgehog Proteins/*immunology

Male

Mesenchymal Stem Cells/cytology/*drug effects/immunology

Neural Stem Cells/cytology/*drug effects/immunology

Rats

Rats, Sprague-Dawley

Structure-Activity Relationship

Tretinoin/*pharmacology

Retinoic Acid

Sonic Hedgehog

glutamatergic sensory neurons

induced

mesenchymal stem cell

LA - eng

N1 - 1532-4230

Yu, Zhenhai

Wu, Shixing

Liu, Zhen

Lin, Haiyan

Chen, Lei

Yuan, Xinli

Zhang, Zhiying

Liu, Fang

Zhang, Chuansen

Journal Article

Research Support, Non-U.S. Gov't

England

J Immunoassay Immunochem. 2015;36(1):1-15. doi: 10.1080/15321819.2014.889025.

PY - 2015

SN - 1532-1819

SP - 1-15

ST - Sonic hedgehog and retinoic Acid induce bone marrow-derived stem cells to differentiate into glutamatergic neural cells

T2 - J Immunoassay Immunochem

TI - Sonic hedgehog and retinoic Acid induce bone marrow-derived stem cells to differentiate into glutamatergic neural cells

VL - 36

ID - 39635

ER -

TY - JOUR

AB - Gene therapy for genetic hearing loss is a nascent field with just a handful of studies published to date that demonstrate proof‐of‐concept recovery of auditory function (reviewed in Ahmed et al, 2017; Lustig & Akil, 2018). One challenge that faces the inner ear field, as well as the broader gene therapy field, is the need to deliver large gene sequences despite the limited genetic capacity (~4.5 kB) of delivery vehicles such as adeno‐associated viral vectors (AAV). In this issue, Al‐Moyed et al have overcome this conundrum by using two AAV vectors to deliver the coding sequence for otoferlin, which is ~6 kB. With dual‐AAV delivery of split otoferlin and a trans‐splicing approach, they demonstrate recombination of full‐length otoferlin in sensory hair cells of the inner ear, enabling partial restoration of auditory function in deaf, otoferlin‐null mice.

AD - Department of Otolaryngology, Boston Children's Hospital and Harvard Medical School, Boston, MA, USA.

AN - 30573543

AU - Holt, J. R.

AU - Geleoc, G. S.

C1 - PubMed

C2 - PMC6328905

DA - Jan

DO - 10.15252/emmm.201809995

DP - NLM

ET - 2018/12/24

IS - 1

KW - Animals

*Exocytosis

*Hearing

Membrane Proteins

Mice

Mice, Knockout

LA - eng

N1 - 1757-4684

Holt, Jeffrey R

Orcid: 0000-0002-7182-8011

Geleoc, Gwenaelle Sg

Orcid: 0000-0002-2825-6521

Comment

Journal Article

Germany

EMBO Mol Med. 2019 Jan;11(1):e9995. doi: 10.15252/emmm.201809995.

PY - 2019

SN - 1757-4676 (Print)

1757-4676

ST - Split otoferlins reunited

T2 - EMBO Mol Med

TI - Split otoferlins reunited

VL - 11

ID - 39589

ER -

TY - JOUR

AB - Relationships between novel phenotypic behaviors and specific genetic alterations are often discovered using target-specific, directed mutagenesis or phenotypic selection following chemical mutagenesis. An alternative approach is to exploit deficiencies in DNA repair pathways that maintain genetic integrity in response to spontaneously induced damage. Mice deficient in the DNA glycosylase NEIL1 show elevated spontaneous mutations, which arise from translesion DNA synthesis past oxidatively induced base damage. Several litters of Neil1 knockout mice included animals that were distinguished by their backwards-walking behavior in open-field environments, while maintaining frantic forward movements in their home cage environment. Other phenotypic manifestations included swim test failures, head tilting and circling. Mapping of the mutation that conferred these behaviors showed the introduction of a stop codon at amino acid 4 of the Ush1g gene. Ush1g(bw/bw) null mice displayed auditory and vestibular defects that are commonly seen with mutations affecting inner-ear hair-cell function, including a complete lack of auditory brainstem responses and vestibular-evoked potentials. As in other Usher syndrome type I mutant mouse lines, hair cell phenotypes included disorganized and split hair bundles, as well as altered distribution of proteins for stereocilia that localize to the tips of row 1 or row 2. Disruption to the bundle and kinocilium displacement suggested that USH1G is essential for forming the hair cell's kinocilial links. Consistent with other Usher type 1 models, Ush1g(bw/bw) mice had no substantial retinal degeneration compared with Ush1g(bw) /+ controls. In contrast to previously described Ush1g alleles, this new allele provides the first knockout model for this gene.

AD - Oregon Institute of Occupational Health Sciences, Oregon Health & Science University, Portland, Oregon, USA.

Oregon Hearing Research Center and Vollum Institute, Oregon Health & Science University, Portland, Oregon, USA.

Department of Ophthalmology, Casey Eye Institute, Oregon Health & Science University, Portland, Oregon, USA.

Oregon Institute of Occupational Health Sciences and School of Public Health, Oregon Health & Science University-Portland State University, Portland, Oregon, USA.

Department of Special Education and Communication Disorders, University of Nebraska-Lincoln, Lincoln, Nebraska, USA.

Department of Nutritional Sciences and New Jersey Institute for Food, Nutrition, and Health, Rutgers University, New Brunswick, New Jersey, USA.

Oregon Institute of Occupational Health Sciences and Department of Behavioral Neuroscience, Oregon Health & Science University, Portland, Oregon, USA.

Department of Molecular and Medical Genetics, Oregon Health & Science University, Portland, Oregon, USA.

AN - 37328946

AU - Vartanian, V.

AU - Krey, J. F.

AU - Chatterjee, P.

AU - Curtis, A.

AU - Six, M.

AU - Rice, S. P. M.

AU - Jones, S. M.

AU - Sampath, H.

AU - Allen, C. N.

AU - Ryals, R. C.

AU - Lloyd, R. S.

AU - Barr-Gillespie, P. G.

C1 - PubMed

C2 - PMC10393423

DA - Aug

DO - 10.1111/gbb.12849

DP - NLM

ET - 2023/06/17

IS - 4

KW - Mice

Animals

Alleles

*Usher Syndromes/genetics

Mutation

Phenotype

*DNA Glycosylases/genetics

DNA repair

backward walking mouse

cochlea

hair cell

hyperactivity

retina

stereocilia

LA - eng

N1 - 1601-183x

Vartanian, Vladimir

Krey, Jocelyn F

Chatterjee, Paroma

Curtis, Allison

Six, Makayla

Rice, Sean P M

Jones, Sherri M

Sampath, Harini

Allen, Charles N

Ryals, Renee C

Lloyd, R Stephen

Barr-Gillespie, Peter G

Orcid: 0000-0002-9787-5860

P30 EY010572/EY/NEI NIH HHS/United States

R01 DC002368/DC/NIDCD NIH HHS/United States

R01 ES031086/ES/NIEHS NIH HHS/United States

Journal Article

Research Support, N.I.H., Extramural

Research Support, Non-U.S. Gov't

England

Genes Brain Behav. 2023 Aug;22(4):e12849. doi: 10.1111/gbb.12849. Epub 2023 Jun 16.

PY - 2023

SN - 1601-1848 (Print)

1601-183x

SP - e12849

ST - Spontaneous allelic variant in deafness-blindness gene Ush1g resulting in an expanded phenotype

T2 - Genes Brain Behav

TI - Spontaneous allelic variant in deafness-blindness gene Ush1g resulting in an expanded phenotype

VL - 22

ID - 38979

ER -

TY - JOUR

AB - Stereocilia are mechanosensitive protrusions on the surfaces of sensory hair cells in the inner ear that detect sound, gravity, and head movement. Their cores are composed of parallel actin filaments that are cross-linked and stabilized by several actin-binding proteins, including fascin-2, plastin-1, espin, and XIRP2. The actin filaments are the most stable known, with actin turnover primarily occurring at the stereocilia tips. While stereocilia actin dynamics has been well studied, little is known about the behavior of the actin cross-linking proteins, which are the most abundant type of protein in stereocilia after actin and are critical for stereocilia morphogenesis and maintenance. Here, we developed a novel transgenic mouse to monitor EGFP-fascin-2 incorporation . In contrast to actin, EGFP-fascin-2 readily enters the stereocilia core. We also compared the effect of EGFP-fascin-2 expression on developing and mature stereocilia. When it was induced during hair cell development, we observed increases in both stereocilia length and width. Interestingly, stereocilia size was not affected when EGFP-fascin-2 was induced in adult stereocilia. Regardless of the time of induction, EGFP-fascin-2 displaced both espin and plastin-1 from stereocilia. Altering the actin cross-linker composition, even as the actin filaments exhibit little to no turnover, provides a mechanism for ongoing remodeling and repair important for stereocilia homeostasis.

AD - Department of Biology, Indiana University-Purdue University Indianapolis, Indianapolis, IN 46202.

AN - 29874122

AU - Roy, P.

AU - Perrin, B. J.

C1 - PubMed

C2 - PMC6085822

DA - Aug 1

DO - 10.1091/mbc.E18-03-0196

DP - NLM

ET - 2018/06/07

IS - 15

KW - Actins/*metabolism

Animals

Carrier Proteins/metabolism

Cross-Linking Reagents/*metabolism

Fluorescence Recovery After Photobleaching

Green Fluorescent Proteins/metabolism

Hair Cells, Auditory/metabolism

*Mechanotransduction, Cellular

Mice, Inbred C57BL

Mice, Transgenic

Microfilament Proteins/metabolism

Stereocilia/*metabolism/ultrastructure

LA - eng

N1 - 1939-4586

Roy, Pallabi

Perrin, Benjamin J

R01 DC015495/DC/NIDCD NIH HHS/United States

Journal Article

Research Support, N.I.H., Extramural

United States

Mol Biol Cell. 2018 Aug 1;29(15):1856-1865. doi: 10.1091/mbc.E18-03-0196. Epub 2018 Jun 6.

PY - 2018

SN - 1059-1524 (Print)

1059-1524

SP - 1856-1865

ST - The stable actin core of mechanosensory stereocilia features continuous turnover of actin cross-linkers

T2 - Mol Biol Cell

TI - The stable actin core of mechanosensory stereocilia features continuous turnover of actin cross-linkers

VL - 29

ID - 39702

ER -

TY - JOUR

AB - All nonmammalian vertebrates studied can regenerate inner ear mechanosensory receptors (i.e., hair cells) (Corwin and Cotanche, 1988; Lombarte et al., 1993; Baird et al., 1996), but mammals possess only a very limited capacity for regeneration after birth (Roberson and Rubel, 1994). As a result, mammals experience permanent deficiencies in hearing and balance once their inner ear hair cells are lost. The mechanisms of hair cell regeneration are poorly understood. Because the inner ear sensory epithelium is highly conserved in all vertebrates (Fritzsch et al., 2007), we chose to study hair cell regeneration mechanism in adult zebrafish, hoping the results would be transferrable to inducing hair cell regeneration in mammals. We defined the comprehensive network of genes involved in hair cell regeneration in the inner ear of adult zebrafish with the powerful transcriptional profiling technique digital gene expression, which leverages the power of next-generation sequencing ('t Hoen et al., 2008). We also identified a key pathway, stat3/socs3, and demonstrated its role in promoting hair cell regeneration through stem cell activation, cell division, and differentiation. In addition, transient pharmacological inhibition of stat3 signaling accelerated hair cell regeneration without overproducing cells. Taking other published datasets into account (Sano et al., 1999; Schebesta et al., 2006; Dierssen et al., 2008; Riehle et al., 2008; Zhu et al., 2008; Qin et al., 2009), we propose that the stat3/socs3 pathway is a key response in all tissue regeneration and thus an important therapeutic target for a broad application in tissue repair and injury healing.

AD - Genome Technology Branch, and Inherited Disease Research Branch, National Human Genome Research Institute, Bethesda, Maryland 20892, USA.

AN - 22855815

AU - Liang, J.

AU - Wang, D.

AU - Renaud, G.

AU - Wolfsberg, T. G.

AU - Wilson, A. F.

AU - Burgess, S. M.

C1 - PubMed

C2 - PMC3427933

C6 - NIHMS399750

DA - Aug 1

DO - 10.1523/jneurosci.5785-10.2012

DP - NLM

ET - 2012/08/03

IS - 31

KW - Analysis of Variance

Animals

Bromodeoxyuridine/metabolism

Cell Count

Cell Death/drug effects

Cell Differentiation/physiology

Cell Proliferation

Chi-Square Distribution

Copper Sulfate/pharmacology

Female

Gene Expression Regulation, Developmental/drug effects/*physiology

Green Fluorescent Proteins/genetics/metabolism

Hair Cells, Auditory/drug effects/*physiology

Larva

Lateral Line System/cytology/drug effects/growth & development

Male

Morpholinos/pharmacology

Neural Inhibition/drug effects/genetics

Noise/adverse effects

RNA, Messenger/administration & dosage

Regeneration/drug effects/*physiology

STAT3 Transcription Factor/genetics/*metabolism

Signal Transduction/drug effects/*physiology

Suppressor of Cytokine Signaling 3 Protein

Suppressor of Cytokine Signaling Proteins/genetics/*metabolism

Zebrafish

Zebrafish Proteins/genetics/*metabolism

LA - eng

N1 - 1529-2401

Liang, Jin

Wang, Dongmei

Renaud, Gabriel

Wolfsberg, Tyra G

Wilson, Alexander F

Burgess, Shawn M

ZIA HG000183/ImNIH/Intramural NIH HHS/United States

Journal Article

Research Support, N.I.H., Intramural

United States

J Neurosci. 2012 Aug 1;32(31):10662-73. doi: 10.1523/JNEUROSCI.5785-10.2012.

PY - 2012

SN - 0270-6474

SP - 10662-73

ST - The stat3/socs3a pathway is a key regulator of hair cell regeneration in zebrafish. [corrected]

T2 - J Neurosci

TI - The stat3/socs3a pathway is a key regulator of hair cell regeneration in zebrafish. [corrected]

VL - 32

ID - 39144

ER -

TY - JOUR

AB - Regeneration and homeostatic turnover of solid tissues depend on the proliferation of symmetrically dividing adult stem cells, which either remain stem cells or differentiate based on their niche position. Here we demonstrate that in zebrafish lateral line sensory organs, stem and progenitor cell proliferation are independently regulated by two cyclinD genes. Loss of ccnd2a impairs stem cell proliferation during development, while loss of ccndx disrupts hair cell progenitor proliferation but allows normal differentiation. Notably, ccnd2a can functionally replace ccndx, indicating that the respective effects of these Cyclins on proliferation are due to cell type-specific expression. However, even though hair cell progenitors differentiate normally in ccndx mutants, they are mispolarized due to hes2 and Emx2 downregulation. Thus, regulated proliferation ensures that equal numbers of hair cells are polarized in opposite directions. Our study reveals cell type-specific roles for cyclinD genes in regulating the different populations of symmetrically dividing cells governing organ development and regeneration, with implications for regenerative medicine and disease.

AD - Stowers Institute for Medical Research, Kansas City, MO. USA.

Lead contact.

AN - 39484411

AU - Lush, M. E.

AU - Tsai, Y. Y.

AU - Chen, S.

AU - Münch, D.

AU - Peloggia, J.

AU - Sandler, J. E.

AU - Piotrowski, T.

C1 - PubMed

C2 - PMC11526906

DA - Oct 22

DO - 10.1101/2024.10.21.619490

DP - NLM

ET - 2024/11/04

KW - Crispr

Notch

cell cycle

enhancer

live imaging

mitosis

stem cell activation

LA - eng

N1 - 2692-8205

Lush, Mark E

Tsai, Ya-Yin

Chen, Shiyuan

Münch, Daniela

Peloggia, Julia

Sandler, Jeremy E

Piotrowski, Tatjana

R01 DC015488/DC/NIDCD NIH HHS/United States

Journal Article

Preprint

United States

bioRxiv [Preprint]. 2024 Oct 22:2024.10.21.619490. doi: 10.1101/2024.10.21.619490.

PY - 2024

SN - 2692-8205

ST - Stem and progenitor cell proliferation are independently regulated by cell type-specific cyclinD genes

T2 - bioRxiv

TI - Stem and progenitor cell proliferation are independently regulated by cell type-specific cyclinD genes

ID - 39444

ER -

TY - JOUR

AB - Regeneration and homeostatic turnover of solid tissues depend on the proliferation of symmetrically dividing adult stem cells, which either remain stem cells or differentiate based on their niche position. Here we demonstrate that in zebrafish lateral line sensory organs, stem and progenitor cell proliferation are independently regulated by two cyclinD genes. Loss of ccnd2a impairs stem cell proliferation during development, while loss of ccndx disrupts hair cell progenitor proliferation but allows normal differentiation. Notably, ccnd2a can functionally replace ccndx, indicating that the respective effects of these Cyclins on proliferation are due to cell type-specific expression. However, even though hair cell progenitors differentiate normally in ccndx mutants, they are mispolarized due to hes2 and Emx2 downregulation. Thus, regulated proliferation ensures that equal numbers of hair cells are polarized in opposite directions. Our study reveals cell type-specific roles for cyclinD genes in regulating the different populations of symmetrically dividing cells governing organ development and regeneration, with implications for regenerative medicine and disease. © The Author(s) 2025.

AD - Stowers Institute for Medical Research, Kansas City, MO, United States

Department of Biological Chemistry, David Geffen School of Medicine, University of California, Los Angeles (UCLA), Los Angeles, CA, United States

AU - Lush, M. E.

AU - Tsai, Y. Y.

AU - Chen, S.

AU - Münch, D.

AU - Peloggia, J.

AU - Sandler, J. E.

AU - Piotrowski, T.

C1 - Scopus

C2 - 40659618

C7 - 5913

DB - Scopus

DO - 10.1038/s41467-025-60251-0

IS - 1

J2 - Nat. Commun.

KW - Animals

Cell Differentiation

Cell Proliferation

Cyclin D

Cyclin D2

Gene Expression Regulation, Developmental

Hair Cells, Auditory

Stem Cells

Zebrafish

Zebrafish Proteins

cyclin D2 alpha

cyclin DX

genomic DNA

messenger RNA

transcription factor

transcription factor atoh1a

transcription factor EMX2

transcription factor HES2

unclassified drug

zebrafish protein

biochemistry

cell

cyprinid

gene expression

mutation

animal cell

animal experiment

Article

cell cycle

cell division

cell population

cell regeneration

cell specificity

cells by body anatomy

controlled study

down regulation

hair cell (inner ear)

homeostasis

lateral line system

mRNA expression level

neuromast

nonhuman

primordium

protein expression

protein structure

regenerative medicine

sense organ

single cell RNA seq

stem cell

stop codon

time lapse imaging

zebra fish

animal

cochlear hair cell

cytology

gene expression regulation

genetics

metabolism

LA - English

M3 - Article

N1 - Export Date: 21 May 2026; Cited By: 2; Correspondence Address: T. Piotrowski; Stowers Institute for Medical Research, Kansas City, United States; email: pio@stowers.org

PY - 2025

SN - 20411723 (ISSN)

ST - Stem and progenitor cell proliferation are independently regulated by cell type-specific cyclinD genes

T2 - Nature Communications

TI - Stem and progenitor cell proliferation are independently regulated by cell type-specific cyclinD genes

UR - https://www.scopus.com/pages/publications/105010625261?origin=resultslist

VL - 16

ID - 39854

ER -

TY - JOUR

AB - The incidence and prevalence of hearing loss is increasing globally at an accelerated pace. Hair cells represent the sensory receptors of auditory and vestibular systems. Hair cell absence, loss or degeneration due to congenital diseases, trauma, toxicity, infection or advancing age, results in disabling hearing loss. Regenerative medicine approaches consisting in stem cell-based hair cell rescue or regeneration, gene therapy, as well as cell and tissue engineering are expected to dramatically improve the therapeutic arsenal available for addressing hearing loss. Current strategies that are using different stem cell types to rescue or to induce hair cell proliferation and regeneration are presented. Gene and cell therapy methods that modulates transdifferentiation of surrounding cell types into hair cells are presented, together with their specific advantages and limitations. Several modalities for improving therapeutic targeting to the inner ear such as nanoparticle-mediated cell and gene delivery are introduced. Further steps in building more relevant high-throughput models for testing novel drugs and advanced therapies are proposed as a modality to accelerate translation to clinical settings. © 2024 Mary Ann Liebert Inc.. All rights reserved.

AD - Doctoral School, ''Grigore T. Popa'' University of Medicine and Pharmacy, Iasi, Romania

Orthopedics and Traumatology Clinic, Emergency Hospital Saint Spiridon, Iasi, Romania

Magnetic Materials and Sensors, National Institute of Research and Development in Technical Physics, Iasi, Romania

ENT Clinic Department, ''Grigore T. Popa'' University of Medicine and Pharmacy, Iasi, Romania

AU - Cumpata, A. J.

AU - Labusca, L.

AU - Radulescu, L. M.

C1 - Scopus

C2 - 37440318

DB - Scopus

DO - 10.1089/ten.teb.2023.0084

IS - 1

J2 - Tissue Eng. Part B. Rev.

KW - auditory hair cells

extracellular vesicles

gene therapy

hearing loss

stem cell therapy

tissue engineering

Deafness

Hair Cells, Auditory

Humans

Regeneration

Stem Cells

Audition

Cell engineering

Cell proliferation

Controlled drug delivery

Gene transfer

Tissue

Tissue regeneration

Auditory hair cell

Cell types

Cell-based therapy

Extracellular

Extracellular vesicle

Hair cells

Stem-cell

Tissues engineerings

antiinflammatory activity

cell differentiation

cell regeneration

cell transdifferentiation

clinical practice

cochlear hair cell

Deiters cell

gene targeting

hearing impairment

high throughput analysis

human

mesenchymal stem cell

nonhuman

prophylaxis

Review

stem cell transplantation

metabolism

stem cell

LA - English

M3 - Review

N1 - Export Date: 21 May 2026; Cited By: 7; Correspondence Address: L. Labusca; Magnetic Materials and sensors, National Institute of Research and Development in Technical Physics, Iasi, Bulevard Prof Dr Dimitrie Mangeron Nr 47, 7000000, Romania; email: drlluminita@yahoo.com

PY - 2024

SN - 19373368 (ISSN)

SP - 15-28

ST - Stem Cell-Based Therapies for Auditory Hair Cell Regeneration in the Treatment of Hearing Loss

T2 - Tissue Engineering - Part B: Reviews

TI - Stem Cell-Based Therapies for Auditory Hair Cell Regeneration in the Treatment of Hearing Loss

UR - https://www.scopus.com/pages/publications/85168733873?origin=resultslist

VL - 30

ID - 39856

ER -

TY - JOUR

AB - Permanent hearing loss was considered which cannot be cured since cochlear hair cells and primary afferent neurons cannot be regenerated. In recent years, due to the in-depth study of stem cell and its therapeutic potential, regenerating auditory sensory cells is made possible. By using two strategies of endogenous stem cell activation and exogenous stem cell transplantation, researchers hope to find methods to restore hearing function. However, there are complex factors that need to be considered in the in vivo application of stem cell therapy, such as stem cell-type choice, signaling pathway regulations, transplantation approaches, internal environment of the cochlea, and external stimulation. After years of investigations, some theoretic progress has been made in the treatment of hearing loss using stem cells, but there are also many problems which limited its application that need to be solved. Understanding the future perspective of stem cell therapy in hearing loss, solving the encountered problems, and promoting its development are the common goals of audiological researchers. In this review, we present critical experimental findings of stem cell therapy on treatment of hearing loss and intend to bring hope to researchers and patients.

AD - Department of Otolaryngology Head and Neck Surgery, Xijing Hospital, Air Force Military Medical University, Xi'an, Shaanxi, People's Republic of China.

Institutes of Biomedical Sciences, Fudan University, Shanghai, People's Republic of China.

ENT Institute and Otorhinolaryngology Department, Affiliated Eye and ENT Hospital, State Key Laboratory of Medical Neurobiology, Fudan University, Shanghai, People's Republic of China.

Department of Otolaryngology Head and Neck Surgery, Xijing Hospital, Air Force Military Medical University, Xi'an, Shaanxi, People's Republic of China. qiujh@fmmu.edu.cn.

AN - 30915707

AU - Qiu, Y.

AU - Qiu, J.

C1 - PubMed

DO - 10.1007/978-981-13-6123-4_10

DP - NLM

ET - 2019/03/28

KW - Cochlea

Deafness/*urine

Hair Cells, Auditory

Hearing Loss/*therapy

Humans

Neurons, Afferent

*Stem Cell Transplantation

Stem Cells/*cytology

Cell transplantation

Hair cell regeneration

Hearing loss

Stem cells

LA - eng

N1 - Qiu, Yang

Qiu, Jianhua

Journal Article

Review

United States

Adv Exp Med Biol. 2019;1130:165-180. doi: 10.1007/978-981-13-6123-4_10.

PY - 2019

SN - 0065-2598 (Print)

0065-2598

SP - 165-180

ST - Stem Cells: A New Hope for Hearing Loss Therapy

T2 - Adv Exp Med Biol

TI - Stem Cells: A New Hope for Hearing Loss Therapy

VL - 1130

ID - 38862

ER -

TY - JOUR

AB - In contrast to mammals, the avian cochlea, specifically the basilar papilla, can regenerate sensory hair cells, which involves fate conversion of supporting cells to hair cells. To determine the mechanisms for converting supporting cells to hair cells, we used single-cell RNA sequencing during hair cell regeneration in explant cultures of chick basilar papillae. We identified dynamic changes in the gene expression of supporting cells, and the pseudotime trajectory analysis demonstrated the stepwise fate conversion from supporting cells to hair cells. Initially, supporting cell identity was erased and transition to the precursor state occurred. A subsequent gain in hair cell identity progressed together with downregulation of precursor-state genes. Transforming growth factor β receptor 1-mediated signaling was involved in induction of the initial step, and its inhibition resulted in suppression of hair cell regeneration. Our data provide new insights for understanding fate conversion from supporting cells to hair cells in avian basilar papillae.

AD - Department of Otolaryngology, Head and Neck Surgery, Graduate School of Medicine, Kyoto University, Kyoto 606-8507, Japan.

AN - 36818302

AU - Matsunaga, M.

AU - Yamamoto, R.

AU - Kita, T.

AU - Ohnishi, H.

AU - Yamamoto, N.

AU - Okano, T.

AU - Omori, K.

AU - Nakagawa, T.

C1 - PubMed

C2 - PMC9932131

DA - Feb 17

DO - 10.1016/j.isci.2023.106046

DP - NLM

ET - 2023/02/24

IS - 2

KW - Cell biology

Sensory neuroscience

Transcriptomics

LA - eng

N1 - 2589-0042

Matsunaga, Mami

Yamamoto, Ryosuke

Kita, Tomoko

Ohnishi, Hiroe

Yamamoto, Norio

Okano, Takayuki

Omori, Koichi

Nakagawa, Takayuki

Journal Article

United States

iScience. 2023 Jan 25;26(2):106046. doi: 10.1016/j.isci.2023.106046. eCollection 2023 Feb 17.

PY - 2023

SN - 2589-0042

SP - 106046

ST - Stepwise fate conversion of supporting cells to sensory hair cells in the chick auditory epithelium

T2 - iScience

TI - Stepwise fate conversion of supporting cells to sensory hair cells in the chick auditory epithelium

VL - 26

ID - 38957

ER -

TY - JOUR

AB - The nitrile 3,3'-iminodipropionitrile (IDPN) causes a loss of hair cells in the vestibular epithelium of the inner ear in several species of both mammals and nonmammals. It is of interest as a model compound in ototoxicity and vestibular regeneration research, but its effects on the mouse, including the potential relevance of strain and sex differences for susceptibility, have not yet been thoroughly characterized. In this study, we compared the vestibular toxicity of IDPN in dose-response studies (0, 8, 12, 16, and 24 mmol/kg IDPN p.o.) in males and females of 2 different mouse strains (RjOrl:Swiss/CD-1 and 129S1/SvImJ). 3,3'-Iminodipropionitrile caused a dose-dependent loss of vestibular function in all sex and strain groups, as assessed by a specific battery of behavioral tests. However, large differences in systemic toxicity were recorded, with high systemic toxicity in 129S1 mice of both sexes compared to limited effects on the Swiss mice. Both male and female Swiss mice showed a marked increase of hindlimb stride width after exposure. The Swiss, but not the 129S1, mice treated with IDPN showed hyperactivity in the open field. The dose-response relationships in the behavioral effects were matched by the extent of hair cell loss assessed by scanning electron microscopy. Altogether, the data demonstrated prominent strain-dependent differences in the systemic toxicity of IDPN between 129S1 and Swiss mice, in contrast to no differences between the strains and small differences between the sexes in its vestibular toxicity. These results support the use of Swiss mice exposed to IDPN as a mouse lesion model for research in vestibular therapy and regeneration.

AD - Grup de Recerca d'Anatomia Clínica, Embriologia i Neurociència (NEOMA), Departament de Ciències Mèdiques, Facultat de Medicina, Universitat de Girona, 17003 Girona, Catalunya, Spain.

Departament de Ciències Fisiològiques and Institut de Neurociències, Universitat de Barcelona, 08907 Hospitalet de Llobregat, Catalunya, Spain.

Institut d'Investigació Biomèdica de Bellvitge (IDIBELL), 08907 Hospitalet de Llobregat, Catalunya, Spain.

AN - 28013217

AU - Boadas-Vaello, P.

AU - Sedó-Cabezón, L.

AU - Verdú, E.

AU - Llorens, J.

C1 - PubMed

DA - Mar 1

DO - 10.1093/toxsci/kfw238

DP - NLM

ET - 2016/12/26

IS - 1

KW - Administration, Oral

Akathisia, Drug-Induced/*pathology/physiopathology

Animals

Animals, Outbred Strains

Behavior, Animal/drug effects

Bilateral Vestibulopathy/*chemically induced/pathology/physiopathology

Dose-Response Relationship, Drug

*Drug Resistance

Exploratory Behavior/drug effects

Female

Gait/drug effects

Hair Cells, Vestibular/*drug effects/pathology/ultrastructure

Male

Mice

Mice, 129 Strain

Microscopy, Electron, Scanning

Nitriles/administration & dosage/*toxicity

Sex Characteristics

Species Specificity

Toxicity Tests, Acute

Weight Loss/drug effects

Xenobiotics/administration & dosage/*toxicity

Idpn

hair cell

hyperactivity

mouse.

vestibular function

vestibular toxicity

LA - eng

N1 - 1096-0929

Boadas-Vaello, Pere

Sedó-Cabezón, Lara

Verdú, Enrique

Llorens, Jordi

Comparative Study

Journal Article

Research Support, Non-U.S. Gov't

United States

Toxicol Sci. 2017 Mar 1;156(1):109-122. doi: 10.1093/toxsci/kfw238.

PY - 2017

SN - 1096-0929

SP - 109-122

ST - Strain and Sex Differences in the Vestibular and Systemic Toxicity of 3,3'-Iminodipropionitrile in Mice

T2 - Toxicol Sci

TI - Strain and Sex Differences in the Vestibular and Systemic Toxicity of 3,3'-Iminodipropionitrile in Mice

VL - 156

ID - 39623

ER -

TY - JOUR

AB - Despite impressive technical progress in the field of conventional hearing aids and implantable hearing systems, the hopes for the treatment of inner ear diseases such as hearing loss and tinnitus have become increasingly directed toward regenerative therapeutic approaches. This review discusses the currently most promising strategies for hair cell regeneration in the inner ear to treat hearing loss, including stem cell-based, gene transfer-based, and pharmacological interventions. Furthermore, previous milestones and ground-breaking work in this scientific field are identified. After many years of basic research, the first clinical trials with a regenerative therapeutic approach for hearing-impaired patients were recently initiated. Although there is still a long and bumpy road ahead until a true breakthrough is achieved, it seems more realistic than ever that regenerative therapies for the inner ear will find their way into clinical practice. © 2018, Springer Medizin Verlag GmbH, ein Teil von Springer Nature.

AD - Department of Otorhinolaryngology, Head and Neck Surgery, University Hospital Frankfurt, Theodor-Stern-Kai 7, Frankfurt/M, 60590, Germany

AU - Diensthuber, M.

AU - Stöver, T.

C1 - Scopus

C2 - 29318324

DB - Scopus

DO - 10.1007/s00106-017-0467-0

J2 - HNO

KW - Gene therapy

Hearing loss

Inner ear

Regeneration

Stem cells

Deafness

Ear, Inner

Hair Cells, Auditory

Humans

cochlear hair cell

hearing impairment

human

LA - English

M3 - Review

N1 - Export Date: 21 May 2026; Cited By: 13; Correspondence Address: M. Diensthuber; Department of Otorhinolaryngology, Head and Neck Surgery, University Hospital Frankfurt, Frankfurt/M, Theodor-Stern-Kai 7, 60590, Germany; email: marc.diensthuber@kgu.de; CODEN: HBZHA

PY - 2018

SN - 00176192 (ISSN)

SP - 39-46

ST - Strategien für eine regenerative Therapie der Schwerhörigkeit

T2 - HNO

TI - Strategies for a regenerative therapy of hearing loss

UR - https://www.scopus.com/pages/publications/85040222032?origin=resultslist

VL - 66

ID - 39869

ER -

TY - JOUR

AB - The lipid phosphatase synaptojanin 1 (synj1) is required for the disassembly of clathrin coats on endocytic compartments. In neurons such activity is necessary for the recycling of endocytosed membrane into synaptic vesicles. Mutations in zebrafish synj1 have been shown to disrupt the activity of ribbon synapses in sensory hair cells. After prolonged mechanical stimulation of hair cells, both phase locking of afferent nerve activity and the recovery of spontaneous release of synaptic vesicles are diminished in synj1 mutants. Presumably as a behavioral consequence of these synaptic deficits, synj1 mutants are unable to maintain an upright posture. To probe vestibular function with respect to postural control in synj1 mutants, we developed a method for assessing the vestibulospinal reflex (VSR) in larvae. We elicited the VSR by rotating the head and recorded tail movements. As expected, the VSR is completely absent in pcdh15a and lhfpl5a mutants that lack inner ear function. Conversely, lhfpl5b mutants, which have a selective loss of function of the lateral line organ, have normal VSRs, suggesting that the hair cells of this organ do not contribute to this reflex. In contrast to mechanotransduction mutants, the synj1 mutant produces normal tail movements during the initial cycles of rotation of the head. Both the amplitude and temporal aspects of the response are unchanged. However, after several rotations, the VSR in synj1 mutants was strongly diminished or absent. Mutant synj1 larvae are able to recover, but the time required for the reappearance of the VSR after prolonged stimulation is dramatically increased in synj1 mutants. Collectively, the data demonstrate a behavioral correlate of the synaptic defects caused by the loss of synj1 function. Our results suggest that defects in synaptic vesicle recycling give rise to fatigue of ribbons synapses and possibly other synapses of the VS circuit, leading to the loss of postural control.

AD - Department of Otolaryngology, Stanford University, Stanford, CA, United States.

AN - 33584199

AU - Gao, Y.

AU - Nicolson, T.

C1 - PubMed

C2 - PMC7874208

DO - 10.3389/fnmol.2020.604189

DP - NLM

ET - 2021/02/16

KW - hair cell

ribbon synapse

synaptic vesicle (SV)

synaptojanin

vestibulospinal reflex

zebrafish

commercial or financial relationships that could be construed as a potential

conflict of interest.

LA - eng

N1 - 1662-5099

Gao, Yan

Nicolson, Teresa

R01 DC017046/DC/NIDCD NIH HHS/United States

Journal Article

Switzerland

Front Mol Neurosci. 2021 Jan 18;13:604189. doi: 10.3389/fnmol.2020.604189. eCollection 2020.

PY - 2020

SN - 1662-5099 (Print)

1662-5099

SP - 604189

ST - Temporal Vestibular Deficits in synaptojanin 1 (synj1) Mutants

T2 - Front Mol Neurosci

TI - Temporal Vestibular Deficits in synaptojanin 1 (synj1) Mutants

VL - 13

ID - 39775

ER -

TY - JOUR

AB - CONCLUSION: rAAV-NT4-ADNF-9 could ameliorate the damage to auditory function and repair previous impairment of cochlear hair cell loss induced by kanamycin. OBJECTIVE: To investigate the therapeutic effect of ADNF-9 on cochlear hair cells using the recombinant adeno-associated virus (AAV) carrying fusion gene NT4-ADNF-9 and the kanamycin-deafened guinea pig model. METHODS: Forty white guinea pigs with normal auricle reflex and normal auditory brainstem responses (ABRs) were randomly divided into four groups. Kanamycin was administered to the animals in groups A, B, and C to establish the deafened guinea pig model. rAAV-NT4-ADNF-9, vector only, and artificial perilymph were then delivered to the cochlear tissue of animals in groups A, B, and C, respectively, through the round window membrane. Animals in group D did not receive any treatment and acted as normal controls. The hearing thresholds on the surgery side were recorded before and after the transfection treatment. Fourteen days after treatment, cochleae were removed for paraffin slide preparation and cochlear surface preparation. A phase contrast microscope was used to observe the protective effect of ADNF-9 on hair cells. RESULTS: Significant reduction of the ABR threshold was observed after rAAV-NT4-ADNF-9 treatment (p < 0.05). After 14 days of treatment, the ABR threshold was also significantly different between the rAAV-NT4-ADNF-9-infected group and the non-infected group. Moreover, phase contrast microscopy showed significantly less hair cell damage or hair cell loss in the group treated with rAAV-NT4-ADNF-9 than in the groups treated with vector only or artificial perilymph (p < 0.05).

AD - Department of Otorhinolaryngology, Second Hospital of Xi`an jiaotong University , Xi`an.

AN - 24032567

AU - Zheng, G.

AU - Zhu, Z.

AU - Zhu, K.

AU - Wei, J.

AU - Jing, Y.

AU - Duan, M.

C1 - PubMed

DA - Oct

DO - 10.3109/00016489.2013.799777

DP - NLM

ET - 2013/09/17

IS - 10

KW - Animals

Cells, Cultured

Cochlea/*drug effects/pathology

Deafness/chemically induced/metabolism/*therapy

Dependovirus/*genetics

Disease Models, Animal

Evoked Potentials, Auditory, Brain Stem/drug effects

Genetic Vectors

Guinea Pigs

Hair Cells, Auditory/drug effects/metabolism

Kanamycin/toxicity

Oligopeptides/*biosynthesis

Transfection

LA - eng

N1 - 1651-2251

Zheng, Guoxi

Zhu, Zhu

Zhu, Kang

Wei, Junrong

Jing, Yang

Duan, Maoli

Comparative Study

Journal Article

Research Support, Non-U.S. Gov't

England

Acta Otolaryngol. 2013 Oct;133(10):1022-9. doi: 10.3109/00016489.2013.799777.

PY - 2013

SN - 0001-6489

SP - 1022-9

ST - Therapeutic effect of adeno-associated virus (AAV)-mediated ADNF-9 expression on cochlea of kanamycin-deafened guinea pigs

T2 - Acta Otolaryngol

TI - Therapeutic effect of adeno-associated virus (AAV)-mediated ADNF-9 expression on cochlea of kanamycin-deafened guinea pigs

VL - 133

ID - 39340

ER -

TY - JOUR

AB - Regenerative capacity varies significantly among species and across different organs. In mammals, the inner ear exhibits a limited ability to regenerate hair cells, whereas the lateral line sensory organ of zebrafish demonstrates apparently limitless capability to spontaneously regenerate hair cells following injury. However, the specific cell populations representing the stem cells in the neuromast and the mechanism of stem cell self-renewal remain unclear. In this study, we show that a mutation in tnfrsfa led to the depletion of neuromast sensory organs and impairment of hair cell regeneration in zebrafish. The decrease of cells peripheral to the neuromasts in tnfrsfa mutants indicates that these peripheral cells, particularly cells called "mantle cells", serve as the stem cells within the neuromast. Furthermore, our findings indicate that the ligands Tnfsf10 and Tnfsf10l3 (TRAIL) act through Tnfrsfa activating the NF-κB signaling pathway in the mantle cells in a non-cell-autonomous manner. NF-κB signaling is crucial for maintaining the stem cell identity of the mantle cells through activation or maintenance of sox2 expression. These findings demonstrate a critical role for TNF superfamily signaling in stem cell maintenance.

AD - Key Laboratory of Exploration and Utilization of Aquatic Genetic Resources, Ministry of Education, Shanghai Ocean University, Shanghai, China.

International Research Center for Marine Biosciences, Ministry of Science and Technology, Shanghai Ocean University, Shanghai, China.

Translational and Functional Genomics Branch, National Human Genome Research Institute, Bethesda, MD, USA.

AN - 40568085

AU - Fan, C.

AU - Yuan, X.

AU - Wang, J.

AU - Pei, W.

AU - Zheng, H.

AU - Zhang, X.

AU - Burgess, S. M.

C1 - PubMed

C2 - PMC12190914

DA - Apr 19

DO - 10.1101/2025.04.18.649014

DP - NLM

ET - 2025/06/26

KW - Nf-κb

Sox2

Trail

Tnfrsfa

Tnfsf10

hair cell regeneration

lateral line

stem cell niche

zebrafish

LA - eng

N1 - 2692-8205

Fan, Chunxin

Orcid: 0000-0002-8673-4595

Yuan, Xiaoyi

Wang, Jian

Pei, Wuhong

Zheng, Hongfei

Zhang, Xinyu

Burgess, Shawn M

Orcid: 0000-0003-1147-0596

ZIA HG200386/ImNIH/Intramural NIH HHS/United States

Journal Article

Preprint

United States

bioRxiv [Preprint]. 2025 Apr 19:2025.04.18.649014. doi: 10.1101/2025.04.18.649014.

PY - 2025

SN - 2692-8205

ST - Tnfrsf10 Signaling is Required to Maintain the Stem Cell Niche in the Zebrafish Lateral Line

T2 - bioRxiv

TI - Tnfrsf10 Signaling is Required to Maintain the Stem Cell Niche in the Zebrafish Lateral Line

ID - 39239

ER -

TY - JOUR

AB - Hearing loss affects ∼10% of adults worldwide. Most sensorineural hearing loss is caused by the progressive loss of mechanosensitive hair cells (HCs) in the cochlea. The molecular mechanisms underlying HC maintenance and loss remain poorly understood. LBH, a transcription co-factor implicated in development, is abundantly expressed in outer hair cells (OHCs). We used Lbh-null mice to identify its role in HCs. Surprisingly, Lbh deletion did not affect differentiation and the early development of HCs, as nascent HCs in Lbh knockout mice had normal looking stereocilia. The stereocilia bundle was mechanosensitive and OHCs exhibited the characteristic electromotility. However, Lbh-null mice displayed progressive hearing loss, with stereocilia bundle degeneration and OHC loss as early as postnatal day 12. RNA-seq analysis showed significant gene enrichment of biological processes related to transcriptional regulation, cell cycle, DNA damage/repair and autophagy in Lbh-null OHCs. In addition, Wnt and Notch pathway-related genes were found to be dysregulated in Lbh-deficient OHCs. Our study implicates, for the first time, loss of LBH function in progressive hearing loss, and demonstrates a critical requirement of LBH in promoting HC survival in adult mice.

AD - Department of Biomedical Sciences, Creighton University School of Medicine, Omaha, NE 68178, USA.

Department of Otorhinolaryngology-Head and Neck Surgery, University of Miami Miller School of Medicine, Miami, FL 33136, USA.

Department of Otorhinolaryngology-Head and Neck Surgery, Beijing Tongren Hospital, Capital Medical University, 100730 Beijing, China.

Department of Surgery, University of Miami Miller School of Medicine, Miami, FL 33136, USA.

AN - 33674448

AU - Liu, H.

AU - Giffen, K. P.

AU - Grati, M.

AU - Morrill, S. W.

AU - Li, Y.

AU - Liu, X.

AU - Briegel, K. J.

AU - He, D. Z.

C1 - PubMed

C2 - PMC8077238

DA - Apr 1

DO - 10.1242/jcs.254458

DP - NLM

ET - 2021/03/07

IS - 7

KW - Animals

Cochlea

Hair Cells, Auditory, Outer

*Hearing Loss

Mice

Stereocilia

*Transcription Factors

Hair cells

Hearing loss

Lbh

Mechanotransduction

RNA-seq

LA - eng

N1 - 1477-9137

Liu, Huizhan

Giffen, Kimberlee P

Grati, M'Hamed

Morrill, Seth W

Li, Yi

Liu, Xuezhong

Briegel, Karoline J

He, David Z

P20 RR018788/RR/NCRR NIH HHS/United States

R01 DC005575/DC/NIDCD NIH HHS/United States

R01 DC016807/DC/NIDCD NIH HHS/United States

R01 GM113256/GM/NIGMS NIH HHS/United States

Journal Article

Research Support, N.I.H., Extramural

Research Support, Non-U.S. Gov't

England

J Cell Sci. 2021 Apr 1;134(7):jcs254458. doi: 10.1242/jcs.254458. Epub 2021 Apr 13.

PY - 2021

SN - 0021-9533 (Print)

0021-9533

ST - Transcription co-factor LBH is necessary for the survival of cochlear hair cells

T2 - J Cell Sci

TI - Transcription co-factor LBH is necessary for the survival of cochlear hair cells

VL - 134

ID - 39044

ER -

TY - JOUR

AB - Currently, adult cochlear hair cells (HCs) lack the capacity to regenerate, particularly the hearing damage caused by the HC damage are hard to recover. Remarkably, Lgr5+ inner ear progenitor cells can be activated to proliferate and regenerate hair cells (HCs) in response to injury, but the epigenetic regulatory roles in HC regeneration from Lgr5+ progenitor cells remain unresolved to date. We here investigate the possible roles of H3K4me3 modification in Lgr5+ progenitor cell proliferation and HC regeneration, and identify these differentially expressed genes associated with different binding regions between untreated Lgr5+ progenitor cells (ULPs) and neomycin-treated Lgr5+ progenitor cells (NLPs). Especially, H3K4me3 modification drives 12 genes involved in regulating proliferation and HC regeneration. Interestingly, we find that transcription factors Zeb1, Fev and Prdm5 are enriched in distinct peaks, implying their probable important roles in modulating neomycin-induced Lgr5+ progenitor cell proliferation and HC regeneration. Overall, our study demonstrates the underlying roles of H3k4me3 modification in Lgr5+ progenitor cell proliferation and HCs regeneration, and provides candidate H3K4me3 modification targets and regulators for subsequent studies.

AD - State Key Laboratory of Bioelectronics, Department of Otolaryngology Head and Neck Surgery, School of Life Sciences and Technology, Zhongda Hospital, Advanced Institute for Life and Health, Jiangsu Province High-Tech Key Laboratory for Bio-Medical Research, Southeast University, Nanjing, 210096, China.

Laboratory for Comparative Genomics and Bioinformatics, College of Life Science, Nanjing Normal University, Nanjing, 210046, China.

Department of Otorhinolaryngology, Head and Neck Surgery, The Second Hospital of Anhui Medical University, Hefei, 230601, Anhui, China.

Department of Otorhinolaryngology, Head and Neck Surgery, The Second Hospital of Anhui Medical University, Hefei, 230601, Anhui, China. jmyang88@163.com.

Laboratory for Comparative Genomics and Bioinformatics, College of Life Science, Nanjing Normal University, Nanjing, 210046, China. mafei01@tsinghua.org.cn.

State Key Laboratory of Bioelectronics, Department of Otolaryngology Head and Neck Surgery, School of Life Sciences and Technology, Zhongda Hospital, Advanced Institute for Life and Health, Jiangsu Province High-Tech Key Laboratory for Bio-Medical Research, Southeast University, Nanjing, 210096, China. renjiec@seu.edu.cn.

Department of Otolaryngology Head and Neck Surgery, Sichuan Provincial People's Hospital, University of Electronic Science and Technology of China, Chengdu, China. renjiec@seu.edu.cn.

Co-Innovation Center of Neuroregeneration, Nantong University, Nantong, 226001, China. renjiec@seu.edu.cn.

Institute for Stem Cell and Regeneration, Chinese Academy of Science, Beijing, China. renjiec@seu.edu.cn.

Beijing Key Laboratory of Neural Regeneration and Repair, Capital Medical University, Beijing, 100069, China. renjiec@seu.edu.cn.

AN - 35668241

AU - Ma, X.

AU - Zhang, S.

AU - Qin, S.

AU - Guo, J.

AU - Yuan, J.

AU - Qiang, R.

AU - Zhou, S.

AU - Cao, W.

AU - Yang, J.

AU - Ma, F.

AU - Chai, R.

C1 - PubMed

DA - Jul

DO - 10.1007/s13577-022-00727-z

DP - NLM

ET - 2022/06/07

IS - 4

KW - Cell Proliferation/genetics

Epigenomics

Hair Cells, Auditory/metabolism

Histones

*Neomycin/adverse effects/metabolism

Stem Cells/metabolism

*Transcriptome/genetics

Differentiation

H3K4me3

Hair cell regeneration

Neomycin

Proliferation

LA - eng

N1 - 1749-0774

Ma, Xiangyu

Zhang, Shasha

Qin, Shijie

Guo, Jiamin

Yuan, Jia

Qiang, Ruiying

Zhou, Shan

Cao, Wei

Yang, Jianming

Ma, Fei

Chai, Renjie

2017YFA0103903/National Key R&D Program of China/

XDA16010303/Strategic Priority Research Program of the Chinese Academy of Science/

No. 81970882/National Natural Science Foundation of China/

81970892/National Natural Science Foundation of China/

BE2019711/Natural Science Foundation from Jiangsu Province/

BK20190062/Natural Science Foundation from Jiangsu Province/

JCYJ20190814093401920/Shenzhen Fundamental Research Program/

No. SKLGE1809/Open Research Fund of State Key Laboratory of Genetic Engineering, Fudan University/

2242020R40137/the Fundamental Research Funds for the Central Universities for the Support Program of Zhishan Youth Scholars of Southeast University/

Journal Article

Japan

Hum Cell. 2022 Jul;35(4):1030-1044. doi: 10.1007/s13577-022-00727-z. Epub 2022 Jun 6.

PY - 2022

SN - 0914-7470

SP - 1030-1044

ST - Transcriptomic and epigenomic analyses explore the potential role of H3K4me3 in neomycin-induced cochlear Lgr5+ progenitor cell regeneration of hair cells

T2 - Hum Cell

TI - Transcriptomic and epigenomic analyses explore the potential role of H3K4me3 in neomycin-induced cochlear Lgr5+ progenitor cell regeneration of hair cells

VL - 35

ID - 39104

ER -

TY - JOUR

AB - Mature mammals exhibit very limited capacity for regeneration of auditory hair cells, while all non-mammalian vertebrates examined can regenerate them. In an effort to find therapeutic targets for deafness and balance disorders, scientists have examined gene expression patterns in auditory tissues under different developmental and experimental conditions. Microarray technology has allowed the large-scale study of gene expression profiles (transcriptomics) at whole-genome levels, but since mRNA expression does not necessarily correlate with protein expression, other methods, such as microRNA analysis and proteomics, are needed to better understand the process of hair cell regeneration. These technologies and some of the results of them are discussed in this review. Although there is a considerable amount of variability found between studies owing to different species, tissues and treatments, there is some concordance between cellular pathways important for hair cell regeneration. Since gene expression and proteomics data is now commonly submitted to centralized online databases, meta-analyses of these data may provide a better picture of pathways that are common to the process of hair cell regeneration and lead to potential therapeutics. Indeed, some of the proteins found to be regulated in the inner ear of animal models (e.g., IGF-1) have now gone through human clinical trials.

AD - Bioinformatics and Information Science Center, Department of Biology, Western Kentucky University, Bowling Green, KY 42101, USA.

Department of Genetics and Developmental Biology, University of Connecticut Health Center, 263 Farmington Avenue, Farmington, CT 06030, USA; rajadinakaran@student.uchc.edu.

AN - 24416530

AU - Smith, M. E.

AU - Rajadinakaran, G.

C1 - PubMed

C2 - PMC3886832

C6 - NIHMS539703

DA - Jul 25

DO - 10.3390/microarrays2030186

DP - NLM

ET - 2014/01/15

IS - 3

KW - gene expression

growth factors

hair cell

inner ear

microRNA

microarray

proteomics

regeneration

transcriptomics

LA - eng

N1 - Smith, Michael E

Rajadinakaran, Gopinath

P20 GM103436/GM/NIGMS NIH HHS/United States

Journal Article

Switzerland

Microarrays (Basel). 2013 Jul 25;2(3). doi: 10.3390/microarrays2030186.

PY - 2013

SN - 2076-3905 (Print)

2076-3905

ST - The Transcriptomics to Proteomics of Hair Cell Regeneration: Looking for a Hair Cell in a Haystack

T2 - Microarrays (Basel)

TI - The Transcriptomics to Proteomics of Hair Cell Regeneration: Looking for a Hair Cell in a Haystack

VL - 2

ID - 39215

ER -

TY - JOUR

AB - Death of mechanosensory hair cells in the inner ear is a common cause of auditory and vestibular impairment in mammals, which have a limited ability to regrow these cells after damage. In contrast, non-mammalian vertebrates including zebrafish can robustly regenerate hair cells following severe organ damage. The zebrafish inner ear provides an understudied model system for understanding hair cell regeneration in organs that are highly conserved with their mammalian counterparts. Here we quantitatively examine hair cell addition during growth and regeneration of the larval zebrafish inner ear. We used a genetically encoded ablation method to induce hair cell death and observed gradual regeneration with correct spatial patterning over two weeks following ablation. Supporting cells, which surround and are a source of new hair cells, divide in response to hair cell ablation, expanding the possible progenitor pool. In parallel, nascent hair cells arise from direct transdifferentiation of progenitor pool cells uncoupled from progenitor division. These findings reveal a previously unrecognized mechanism of hair cell regeneration with implications for how hair cells may be encouraged to regenerate in the mammalian ear.

AD - Molecular and Cellular Biology Graduate Program, Seattle, WA.

Virginia Merrill Bloedel Hearing Research Center, Department of Otolaryngology - Head and Neck Surgery, Seattle, WA.

Neuroscience Graduate Program, Seattle, WA.

Department of Biological Structure University of Washington, Seattle, WA.

AN - 38645220

AU - Beaulieu, M. O.

AU - Thomas, E. D.

AU - Raible, D. W.

C1 - PubMed

C2 - PMC11030336

DA - Apr 11

DO - 10.1101/2024.04.09.588777

DP - NLM

ET - 2024/04/22

KW - hair cell

inner ear

regeneration

transdifferentiation

zebrafish

LA - eng

N1 - 2692-8205

Beaulieu, Marielle O

Orcid: 0000-0002-9819-2658

Thomas, Eric D

Orcid: 0000-0003-4300-2893

Raible, David W

Orcid: 0000-0002-5342-5841

R21 DC015110/DC/NIDCD NIH HHS/United States

T32 DC005361/DC/NIDCD NIH HHS/United States

F31 DC020898/DC/NIDCD NIH HHS/United States

R21 DC019948/DC/NIDCD NIH HHS/United States

T32 GM007270/GM/NIGMS NIH HHS/United States

Journal Article

Preprint

United States

bioRxiv [Preprint]. 2024 Apr 11:2024.04.09.588777. doi: 10.1101/2024.04.09.588777.

PY - 2024

SN - 2692-8205

ST - Transdifferentiation is uncoupled from progenitor pool expansion during hair cell regeneration in the zebrafish inner ear

T2 - bioRxiv

TI - Transdifferentiation is uncoupled from progenitor pool expansion during hair cell regeneration in the zebrafish inner ear

ID - 39050

ER -

TY - JOUR

AB - Sensorineural hearing loss is the most common form of hearing loss in humans, and results from either dysfunction in hair cells, the sensory receptors of sound, or the neurons that innervate hair cells. A specific type of sensorineural hearing loss, referred to as auditory synaptopathy, occurs when hair cells are able to detect sound but fail to transmit sound stimuli at the hair-cell synapse. Auditory synaptopathy can originate from genetic alterations that specifically disrupt hair-cell synapse function. Additionally, environmental factors such as noise exposure can leave hair cells intact but result in loss of hair-cell synapses, and represent an acquired form of auditory synaptopathy. The zebrafish model has emerged as a valuable system for studies of hair-cell function, and specifically hair-cell synaptopathy. In this review, we describe the experimental tools that have been developed to study hair-cell synapses in zebrafish. We discuss how zebrafish genetics has helped identify and define the roles of hair-cell synaptic proteins crucial for hearing in humans, and highlight how studies in zebrafish have contributed to our understanding of hair-cell synapse formation and function. In addition, we also discuss work that has used noise exposure or pharmacological mimic of noise-induced excitotoxicity in zebrafish to define cellular mechanisms underlying noise-induced hair-cell damage and synapse loss. Lastly, we highlight how future studies in zebrafish could enhance our understanding of the pathological processes underlying synapse loss in both genetic and acquired auditory synaptopathy. This knowledge is critical in order to develop therapies that protect or repair auditory synaptic contacts.

AD - Section on Sensory Cell Development and Function, NIDCD/National Institutes of Health, Bethesda, MD, United States.

Department of Otolaryngology, Washington University School of Medicine, St. Louis, MO, United States.

AN - 30258843

AU - Kindt, K. S.

AU - Sheets, L.

C1 - PubMed

C2 - PMC6143809

DO - 10.3389/fcell.2018.00114

DP - NLM

ET - 2018/09/28

KW - deafness/hearing loss

hair cells (HCs)

ribbon synapse

synaptic transmission

zebrafish model system

LA - eng

N1 - 2296-634x

Kindt, Katie S

Sheets, Lavinia

R01 DC016066/DC/NIDCD NIH HHS/United States

ZIA DC000085/ImNIH/Intramural NIH HHS/United States

Journal Article

Review

Switzerland

Front Cell Dev Biol. 2018 Sep 11;6:114. doi: 10.3389/fcell.2018.00114. eCollection 2018.

PY - 2018

SN - 2296-634X (Print)

2296-634x

SP - 114

ST - Transmission Disrupted: Modeling Auditory Synaptopathy in Zebrafish

T2 - Front Cell Dev Biol

TI - Transmission Disrupted: Modeling Auditory Synaptopathy in Zebrafish

VL - 6

ID - 39351

ER -

TY - JOUR

AB - The mammalian hearing organ is a stereotyped cellular assembly with orderly innervation: two types of spiral ganglion neurons (SGNs) innervate two types of differentially distributed hair cells (HCs). HCs and SGNs evolved from single neurosensory cells through gene multiplication and diversification. Independent regulation of HCs and neuronal differentiation through expression of basic helix-loop-helix transcription factors (bHLH TFs: Atoh1, Neurog1, Neurod1) led to the evolution of vestibular HC assembly and their unique type of innervation. In ancestral mammals, a vestibular organ was transformed into the organ of Corti (OC) containing a single row of inner HC (IHC), three rows of outer HCs (OHCs), several unique supporting cell types, and a peculiar innervation distribution. Restoring the OC following long-term hearing loss is complicated by the fact that the entire organ is replaced by a flat epithelium and requires reconstructing the organ from uniform undifferentiated cell types, recapitulating both evolution and development. Finding the right sequence of gene activation during development that is useful for regeneration could benefit from an understanding of the OC evolution. Toward this end, we report on Foxg1 and Lmx1a mutants that radically alter the OC cell assembly and its innervation when mutated and may have driven the evolutionary reorganization of the basilar papilla into an OC in ancestral Therapsids. Furthermore, genetically manipulating the level of bHLH TFs changes HC type and distribution and allows inference how transformation of HCs might have happened evolutionarily. We report on how bHLH TFs regulate OHC/IHC and how misexpression (Atoh1-Cre; Atoh1f/kiNeurog1) alters HC fate and supporting cell development. Using mice with altered HC types and distribution, we demonstrate innervation changes driven by HC patterning. Using these insights, we speculate on necessary steps needed to convert a random mixture of post-mitotic precursors into the orderly OC through spatially and temporally regulated critical bHLH genes in the context of other TFs to restore normal innervation patterns.

AD - Department of Biology, University of Iowa, 129 East Jefferson, Iowa City, IA 52242, USA.

AN - 29718413

AU - Jahan, I.

AU - Elliott, K. L.

AU - Fritzsch, B.

C1 - PubMed

C2 - PMC6104702

DA - Aug 1

DO - 10.1093/icb/icy019

DP - NLM

ET - 2018/05/03

IS - 2

KW - Animals

Cell Differentiation

Correction of Hearing Impairment/*methods

*Evolution, Molecular

Hearing Loss/*therapy

Humans

Mammals/*physiology

Organ of Corti/*growth & development/*metabolism

LA - eng

N1 - 1557-7023

Jahan, Israt

Elliott, Karen L

Fritzsch, Bernd

R03 DC013655/DC/NIDCD NIH HHS/United States

Journal Article

Research Support, N.I.H., Extramural

England

Integr Comp Biol. 2018 Aug 1;58(2):351-365. doi: 10.1093/icb/icy019.

PY - 2018

SN - 1540-7063 (Print)

1540-7063

SP - 351-365

ST - Understanding Molecular Evolution and Development of the Organ of Corti Can Provide Clues for Hearing Restoration

T2 - Integr Comp Biol

TI - Understanding Molecular Evolution and Development of the Organ of Corti Can Provide Clues for Hearing Restoration

VL - 58

ID - 39477

ER -

TY - JOUR

AB - Tissue engineering focuses on three primary components: stem cells, biomaterials, and growth factors. Together, the combination of these components is used to regrow and repair damaged tissues that normally do not regenerate easily on their own. Much attention has been focused on the use of embryonic stem cells (ESCs) and induced pluripotent stem cells (iPSCs), due to their broad differentiation potential. However, ESCs and iPSCs require very detailed protocols to differentiate into target tissues, which are not always successful. Furthermore, procurement of ESCs is considered ethically controversial in some regions and procurement of iPSCs requires laborious transformation of adult tissues and characterization. However, mesenchymal stem cells are an adult stem cell population that are not ethically controversial and are readily available for procurement. Furthermore, mesenchymal stem cells exhibit the ability to differentiate into a variety of cell types arising from the mesoderm. In particular, human Wharton's jelly cells (hWJCs) are mesenchymal-type stem cells found in umbilical cords that possess remarkable differentiation potential. hWJCs are a highly desirable stem cell population due to their abundance in supply, high proliferation rates, and ability to differentiate into multiple cell types arising from all three germ layers. hWJCs are used to generate several neurological phenotypes arising from the ectoderm and are considered for engineering mechanosensory hair cells found in the auditory complex. Here, we report the methods for isolating hWJCs from human umbilical cords and non-virally transfected for use in cochlear tissue engineering studies.

AD - Department of Plastic Surgery, University of Kansas Medical Center, MS 3015, 3901 Rainbow Blvd., Kansas City, KS, 66160, USA.

Department of Chemical and Petroleum Engineering, University of Kansasr, 4149B Learned Hall, 1530 W. 15th St., Lawrence, KS, 66045, USA.

Department of Otolaryngology, Head and Neck Surgery, University of Kansas Medical Center, MS 3010, 3901 Rainbow Blvd., Kansas City, KS, 66160, USA. hstaecker@kumc.edu.

AN - 27259936

AU - Mellott, A. J.

AU - Detamore, M. S.

AU - Staecker, H.

C1 - PubMed

DO - 10.1007/978-1-4939-3615-1_19

DP - NLM

ET - 2016/06/05

KW - Cell Differentiation

Cells, Cultured

Cochlea/*cytology

Humans

Tissue Engineering/*methods

Transfection

Wharton Jelly/*cytology

Atoh1

Characterization

Human Wharton’s jelly cells

Mesenchymal stem cells

Non-viral gene delivery

Nucleofection

LA - eng

N1 - 1940-6029

Mellott, Adam J

Detamore, Michael S

Staecker, Hinrich

R01 AR056347/AR/NIAMS NIH HHS/United States

Journal Article

Research Support, N.I.H., Extramural

Research Support, Non-U.S. Gov't

United States

Methods Mol Biol. 2016;1427:319-45. doi: 10.1007/978-1-4939-3615-1_19.

PY - 2016

SN - 1064-3745

SP - 319-45

ST - The Use of Human Wharton's Jelly Cells for Cochlear Tissue Engineering

T2 - Methods Mol Biol

TI - The Use of Human Wharton's Jelly Cells for Cochlear Tissue Engineering

VL - 1427

ID - 39374

ER -

TY - JOUR

AB - Therapies aimed at the protection and/or regeneration of inner ear hair cells are of great interest, given the significant monetary and quality of life impact of balance disorders. Different viral vectors have been shown to transfect various cell types in the inner ear. The past decade has provided tremendous advances in the use of adenoviral vectors to achieve targeted treatment delivery. Several routes of delivery have been identified to introduce vectors into the inner ear while minimizing injury to surrounding structures. Recently, the transcription factor Atoh1 was determined to play a critical role in hair cell differentiation. Adenoviral-mediated overexpression of Atoh1 in culture and in vivo has demonstrated the ability to regenerate vestibular hair cells by causing transdifferentiation of neighbouring epithelial-supporting cells. Functional recovery of the vestibular system has also been documented following adenoviral-induced Atoh1 overexpression. Experiments demonstrating gene transfer in human vestibular epithelial cells reveal that the human inner ear is a suitable target for gene therapy.

AD - Second Department of Otolaryngology, University of Medicine and Pharmacy Cluj-Napoca, Cluj-Napoca, Romania. silviualbu63@gmail.com

AN - 22303988

AU - Albu, S.

AU - Muresanu, D. F.

C1 - PubMed

C2 - PMC3822967

DA - Sep

DO - 10.1111/j.1582-4934.2012.01540.x

DP - NLM

ET - 2012/02/07

IS - 9

KW - Adenoviridae/genetics

Animals

Cell Differentiation

Cell Transdifferentiation

Disease Models, Animal

Ear, Inner/*cytology/pathology

Genetic Therapy/methods

Genetic Vectors

Hair Cells, Vestibular/cytology/metabolism

Humans

*Regeneration

Transcription Factors/genetics/metabolism

Vestibular Diseases/therapy

Vestibule, Labyrinth/*cytology/pathology

LA - eng

N1 - 1582-4934

Albu, Silviu

Muresanu, Dafin F

Journal Article

Review

England

J Cell Mol Med. 2012 Sep;16(9):1970-7. doi: 10.1111/j.1582-4934.2012.01540.x.

PY - 2012

SN - 1582-1838 (Print)

1582-1838

SP - 1970-7

ST - Vestibular regeneration--experimental models and clinical implications

T2 - J Cell Mol Med

TI - Vestibular regeneration--experimental models and clinical implications

VL - 16

ID - 38970

ER -

TY - JOUR

AB - Noise-induced hearing loss (NIHL) poses an emerging global health problem with only ear protection or sound avoidance as preventive strategies. In addition, however, the cochlea receives some protection from medial olivocochlear (MOC) efferent neurons, providing a potential target for therapeutic enhancement. Cholinergic efferents release ACh (Acetylycholine) to hyperpolarize and shunt the outer hair cells (OHCs), reducing sound-evoked activation. The (α9)(2)(α10)(3) nicotinic ACh receptor (nAChR) on the OHCs mediates this effect. Transgenic knock-in mice with a gain-of-function nAChR (α9L9'T) suffer less NIHL. α9 knockout mice are more vulnerable to NIHL but can be rescued by viral transduction of the α9L9'T subunit. In this study, an HA-tagged gain-of-function α9 isoform was expressed in wildtype mice in an attempt to reduce NIHL. Synaptic integration of the virally-expressed nAChR subunit was confirmed by HA-immunopuncta in the postsynaptic membrane of OHCs. After noise exposure, α9L9'T-HA injected mice had less hearing loss (auditory brainstem response (ABR) thresholds and threshold shifts) than did control mice. ABRs of α9L9'T-HA injected mice also had larger wave1 amplitudes and better recovery of wave one amplitudes post noise exposure. Thus, virally-expressed α9L9'T combines effectively with native α9 and α10 subunits to mitigate NIHL in wildtype cochleas.

AD - Department of Otolaryngology-Head and Neck Surgery, Johns Hopkins University School of Medicine; Baltimore, MD, 21205, USA.

AN - 39314296

AU - Slika, E.

AU - Fuchs, P. A.

AU - Wood, M. B.

C1 - PubMed

C2 - PMC11419007

DA - Sep 15

DO - 10.1101/2024.09.12.612688

DP - NLM

ET - 2024/09/24

LA - eng

N1 - 2692-8205

Slika, Eleftheria

Orcid: 0000-0001-9128-5125

Fuchs, Paul A

Wood, Megan Beers

R01 DC001508/DC/NIDCD NIH HHS/United States

R01 DC016559/DC/NIDCD NIH HHS/United States

Journal Article

Preprint

United States

bioRxiv [Preprint]. 2024 Sep 15:2024.09.12.612688. doi: 10.1101/2024.09.12.612688.

PY - 2024

SN - 2692-8205

ST - Virally-Mediated Enhancement of Efferent Inhibition Reduces Acoustic Trauma in Wild Type Murine Cochleas

T2 - bioRxiv

TI - Virally-Mediated Enhancement of Efferent Inhibition Reduces Acoustic Trauma in Wild Type Murine Cochleas

ID - 39733

ER -

TY - JOUR

AB - INTRODUCTION: Hearing loss is a major health problem, impacting education, communication, interpersonal relationships, and mental health. Drugs that prevent or restore hearing are lacking and hence novel drug targets are sought. There is the possibility of targeting the α9α10 nicotinic acetylcholine receptor (nAChR) in the prevention of noise-induced, hidden hearing loss and presbycusis. This receptor mediates synaptic transmission between medial olivocochlear efferent fibers and cochlear outer hair cells. This target is key since enhanced olivocochlear activity prevents noise-induced hearing loss and delays presbycusis. AREAS COVERED: The work examines the α9α10 nicotinic acetylcholine receptor (nAChR), its role in noise-induced, hidden hearing loss and presbycusis and the possibility of targeting. Data has been searched in Pubmed, the World Report on Hearing from the World Health Organization and the Global Burden of Disease Study 2019. EXPERT OPINION: The design of positive allosteric modulators of α9α10 nAChRs is proposed because of the advantage of reinforcing the medial olivocochlear (MOC)-hair cell endogenous neurotransmission without directly stimulating the target receptors, therefore avoiding receptor desensitization and reduced efficacy. The time is right for the discovery and development of α9α10 nAChRs targeting agents and high throughput screening assays will support this.

AD - Laboratorio de Fisiología y Genética de la Audición, Instituto de Investigaciones en Ingeniería Genética y Biología Molecular "Dr. Héctor N. Torres" (INGEBI), Consejo Nacional de Investigaciones Científicas y Técnicas (CONICET), Buenos Aires, Argentina.

AN - 35225139

AU - Elgoyhen, A. B.

C1 - PubMed

C2 - PMC9007918

C6 - NIHMS1789231

DA - Mar

DO - 10.1080/14728222.2022.2047931

DP - NLM

ET - 2022/03/01

IS - 3

KW - Animals

Humans

Molecular Targeted Therapy

*Presbycusis/drug therapy/metabolism

*Receptors, Nicotinic/metabolism

Synaptic Transmission

hearing loss

positive allosteric modulators

presbycusis

tinnitus

Α9α10 nicotinic receptors

involvement with any organization or entity with a financial interest in or

financial conflict with the subject matter or materials discussed in the

manuscript. This includes employment, consultancies, honoraria, stock ownership

or options, expert testimony, grants or patents received or pending, or

royalties.

LA - eng

N1 - 1744-7631

Elgoyhen, Ana Belén

R01 DC001508/DC/NIDCD NIH HHS/United States

Journal Article

Research Support, N.I.H., Extramural

Review

England

Expert Opin Ther Targets. 2022 Mar;26(3):291-302. doi: 10.1080/14728222.2022.2047931. Epub 2022 Mar 7.

PY - 2022

SN - 1472-8222 (Print)

1472-8222

SP - 291-302

ST - The α9α10 nicotinic acetylcholine receptor: a compelling drug target for hearing loss?

T2 - Expert Opin Ther Targets

TI - The α9α10 nicotinic acetylcholine receptor: a compelling drug target for hearing loss?

VL - 26

ID - 39431

ER -
