# Supplementary material for: From cellular regeneration toward functional integration: a 15-year bibliometric analysis of inner ear hair cell regeneration (2011–2025)
Source: Front Cell Neurosci. 2026 Aug 18;20:1888383. doi: 10.3389/fncel.2026.1888383 (PMC13529467; doi:10.3389/fncel.2026.1888383)
Supplement: Supplementary file 4 [file Supplementary_file_1.doc]

1. Web of science

<https://lib1.cdutcm.edu.cn:7001/https/vpn/65/P7TXE55GPNSXT3LPMNTT6Z5MMF3GT7UBPSTT6Z5P/wos/woscc/summary/e2f0d0e6-698d-4260-84e1-75ba4281122f-019c7f240c/relevance/1>

(TS=("hair cells" OR "inner ear hair cell" OR "inner ear hair cells" OR "cochlea inner ear hair cells")) AND TS=(regeneration OR regenerated OR regenerate OR recycling OR recycle OR rebirth OR regeneratively OR recuperation OR reproduction OR recovery OR regrowth OR reuse OR restore OR regenerating OR renewable OR regenerative OR recyclable OR reclaiming OR revivify OR reborn OR rehabilitation OR reusability OR rejuvenation OR revive OR renew OR regenesis OR renovation OR reviviscence OR revivifying OR retroaction OR retroact OR resurgence OR restoring OR rendition OR renascence OR recuperate OR recreate OR reconstitution OR rewrite OR renaturation OR resurgent OR restoration OR resurrection OR revitalization OR shoot regeneration OR refresh OR reproducibility OR regeneration system OR reprocess OR reutilization)

1667 (2026.02.06)

2. Pubmed

https://pubmed.ncbi.nlm.nih.gov/?term=%28%22hair+cells%22%5BTitle%2FAbstract%5D+OR+%22inner+ear+hair+cell%22%5BTitle%2FAbstract%5D+OR+%22inner+ear+hair+cells%22%5BTitle%2FAbstract%5D+OR+%22cochlear+hair+cells%22%5BTitle%2FAbstract%5D%29+AND+%28regeneration%5BTitle%2FAbstract%5D+OR+regenerated%5BTitle%2FAbstract%5D+OR+regenerate%5BTitle%2FAbstract%5D+OR+regrowth%5BTitle%2FAbstract%5D+OR+restore%5BTitle%2FAbstract%5D+OR+recovery%5BTitle%2FAbstract%5D+OR+rejuvenation%5BTitle%2FAbstract%5D+OR+revitalization%5BTitle%2FAbstract%5D+OR+repair%5BTitle%2FAbstract%5D%29+AND+%28%22Journal+Article%22%5Bpt%5D+OR+%22Review%22%5Bpt%5D%29+AND+english%5Blang%5D&filter=years.2011-2025

("hair cells"[Title/Abstract] OR "inner ear hair cell"[Title/Abstract] OR "inner ear hair cells"[Title/Abstract] OR "cochlear hair cells"[Title/Abstract]) AND (regeneration[Title/Abstract] OR regenerated[Title/Abstract] OR regenerate[Title/Abstract] OR regrowth[Title/Abstract] OR restore[Title/Abstract] OR recovery[Title/Abstract] OR rejuvenation[Title/Abstract] OR revitalization[Title/Abstract] OR repair[Title/Abstract]) AND ("Journal Article"[pt] OR "Review"[pt]) AND english[lang]

1078 (2026.05.21)

3. Scopus

<https://www-scopus-com.auac.edu.kg/pages/search/publications?searchId=9ff8fce3-4e10-4054-bc23-9146b7ff055b>

( TITLE-ABS-KEY ( hair AND cells OR inner AND ear AND hair AND cell OR inner AND ear AND hair AND cells OR cochlea AND inner AND ear AND hair AND cells ) AND TITLE-ABS-KEY ( regeneration OR regenerated OR regenerate OR recycling OR recycle OR rebirth OR regeneratively OR recuperation OR reproduction OR recovery OR regrowth OR reuse OR restore OR regenerating OR renewable OR regenerative OR recyclable OR reclaiming OR revivify OR reborn OR rehabilitation OR reusability OR rejuvenation OR revive OR renew OR regenesis OR renovation OR reviviscence OR revivifying OR retroaction OR retroact OR resurgence OR restoring OR rendition OR renascence OR recuperate OR recreate OR reconstitution OR rewrite OR renaturation OR resurgent OR restoration OR resurrection OR revitalization OR shoot AND regeneration OR refresh OR reproducibility OR regeneration AND system OR reprocess OR reutilization ) ) AND PUBYEAR > 2010 AND PUBYEAR < 2026 AND ( LIMIT-TO ( DOCTYPE , "ar" ) OR LIMIT-TO ( DOCTYPE , "re" ) ) AND ( LIMIT-TO ( LANGUAGE , "English" ) )

232 (2026.05.21)
